# Synthesis and Electrochemical and Spectroscopic Characterization of 4,7-diamino-1,10-phenanthrolines and Their Precursors

**DOI:** 10.3390/molecules24224102

**Published:** 2019-11-13

**Authors:** Jacek E. Nycz, Jakub Wantulok, Romana Sokolova, Lukasz Pajchel, Marek Stankevič, Marcin Szala, Jan Grzegorz Malecki, Daniel Swoboda

**Affiliations:** 1 Institute of Chemistry, University of Silesia in Katowice, ul. Szkolna 9; PL-40007 Katowice, Poland; jakub.wantulok1@gmail.com (J.W.); jan.malecki@us.edu.pl (J.G.M.); daniel.swoboda96@gmail.com (D.S.); 2 J. Heyrovský Institute of Physical Chemistry of the Czech Academy of Sciences, Dolejškova 3, 18223 Prague, Czech Republic; romana.sokolova@jh-inst.cas.cz; 3 Department of Analytical Chemistry and Biomaterials, Faculty of Pharmacy, Medical University of Warsaw, Banacha 1, 02-097 Warsaw, Poland; lpajchel@wum.edu.pl; 4 Department of Organic Chemistry, Marie Curie-Sklodowska University, 33 Gliniana St, PL-20614 Lublin, Poland; marek.stankevic@poczta.umcs.lublin.pl; 5 Institute of Polymer and Dye Technology, Lodz University of Technology, Stefanowskiego 12/16, 90-924 Lodz, Poland; marcin.szala@p.lodz.pl

**Keywords:** phenanthroline, amination, cyclic voltammetry, ^15^N-NMR, cyclization, heterocyclic, DFt, oxidative nucleophilic substitutions of hydrogen

## Abstract

New approaches to the synthesis of 4,7-dichloro-1,10-phenanthrolines and their corresponding 9*H*-carbazol-9-yl-, 10*H*-phenothiazin-10-yl- and pyrrolidin-1-yl derivatives were developed. Their properties have been characterized by a combination of several techniques: MS, HRMS, GC-MS, electronic absorption spectroscopy and multinuclear NMR in both solution and solid state including ^15^N CP/MAS NMR. The structures of 5-fluoro-2,9-dimethyl-4,7-di(pyrrolidin-1-yl)-1,10-phenanthroline (**5d**), 4,7-di(9*H*-carbazol-9-yl)-9-oxo-9,10-dihydro-1,10-phenanthroline-5-carbonitrile (**6a**) and 4,7-di(10*H*-phenothiazin-10-yl)-1,10-phenanthroline-5-carbonitrile (**6b**) were determined by single-crystal X-ray diffraction measurements. The nucleophilic substitutions of hydrogen followed by oxidation produced compounds **6a** and **6b**. The electrochemical properties of selected 1,10-phenanthrolines were investigated using cyclic voltammetry and compared with commercially available reference 1,10-phenanthrolin-5-amine (**5l**). The spatial distribution of frontier molecular orbitals of the selected compounds has been calculated by density functional theory (DFT). It was shown that potentials of reduction and oxidation were in consistence with the level of HOMO and LUMO energies.

## 1. Introduction

1,10-Phenanthrolines are well known bidentate nitrogen ligands used in coordination chemistry for the analysis of transition metal ions and homogenous catalysis [1,2,3,4]. Their coordination abilities are the result of the contribution of lone pairs of electrons on both nitrogen atoms in the characteristic shape of heterotricyclic systems. They have been found applications in supramolecular chemistry, luminescent sensors, and photosensitizers for solar cells [5,6,7,8,9,10,11,12,13,14,15]. 1,10-Phenanthrolines have also received much attention as potential targets for anticancer drug development [16,17,18,19,20,21]. Mainly there are two methods for the synthesis of compounds containing 1,10-phenanthroline core. The first one is the classical Skraup and Friedlander transformations. The second method is used in the synthesis of 4,7-dichloro-1,10-phenanthroline derivatives through the condensation of Meldrum’s acid, orthoesters, and *ortho*-phenylenediamine derivatives, followed by sequential thermal cyclization, decarboxylation and treatment with refluxing phosphoryl chloride [22,23]. The synthesis of 4,7-dichloro-1,10-phenanthrolines offered a possibility of further transformation through replacing halogen atoms in C4 and C7 positions. In the literature there are few methods describing their modification into amino derivatives [24,25]. In this paper, we report new approaches to the synthesis of the aforementioned compounds with in-depth spectroscopic and electrochemical characterization. In order to improve the understanding of the chemical behavior of novel 4,7-dichloro-1,10-phenanthrolines and their amino derivatives, a ^15^N CP/MAS NMR technique was employed to differentiate and characterize the nitrogen atoms presented in their structures, and to show the influence of substituents on their chemical shifts. Additionally, computation and X-ray studies have been carried out of on the novel 4,7-disubstituted-1,10-phenanthrolines. The investigation of the electrochemical properties of these newly designed compounds may provide a promising tool to demonstrate their stability towards oxidative and reductive conditions for applications, such as advanced synthesis and catalysis.

## 2. Results

### 2.1. Synthesis of 4,7-dichloro-1,10-phenanthrolines

**Scheme 1:**
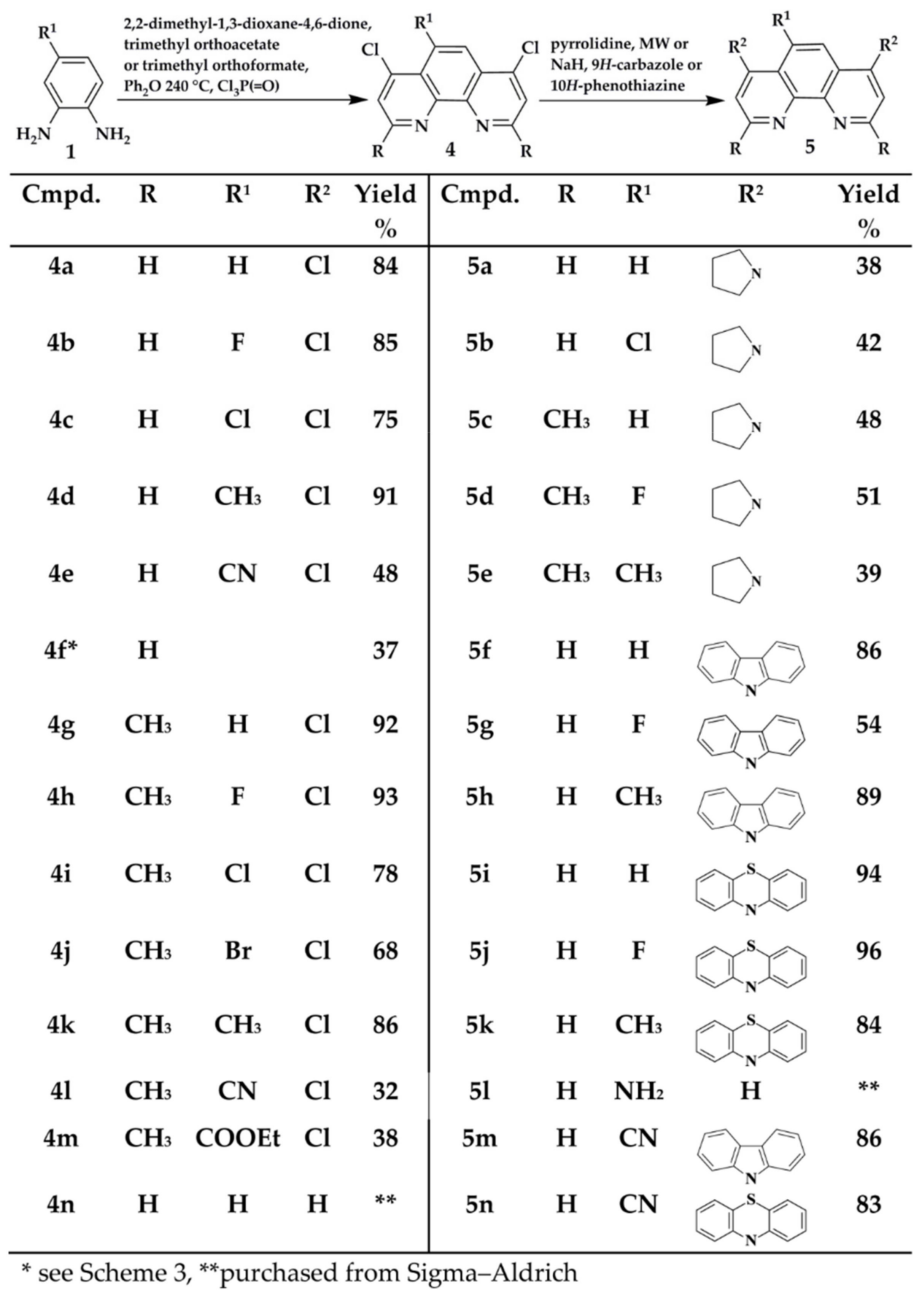
Synthesis route of compounds **4** and **5**.

**Scheme 2:**
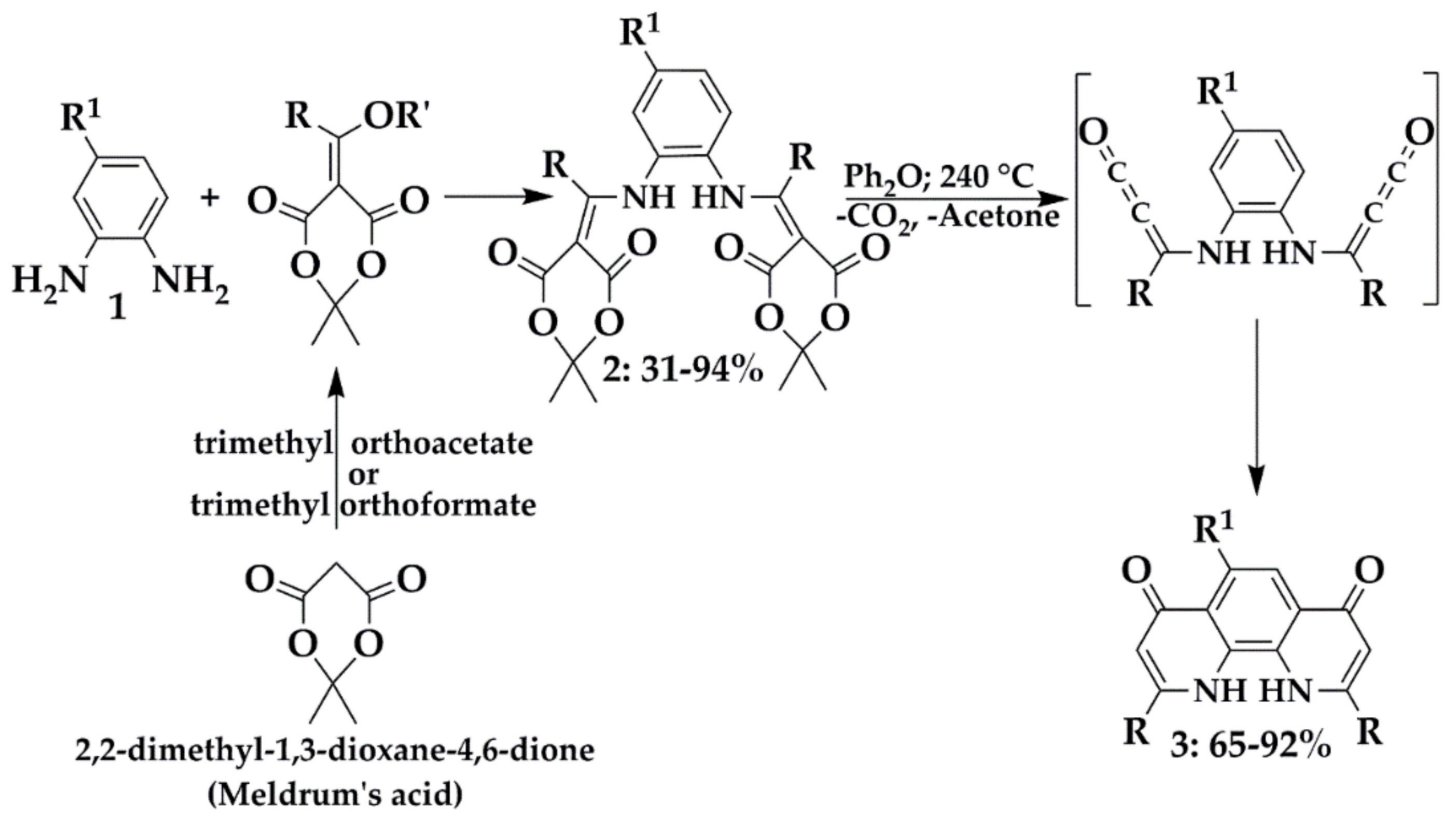
Synthesis route of the compounds **3**. **R** = Me, Et. **R^1^** = H, F, Cl, Br, Me, CN.

In the current study, the functionalization of selected 1,10-phenanthroline derivatives at **R**, **R^1^** and **R^2^** positions was the focus, which have not been fully exploited and may serve as interesting building blocks. The classical Skraup-Doebner-Miller reaction offers the simplest and fastest transformation. To prepare 1,10-phenanthrolines we first attempted to adopt a one-step Skraup-Doebner-Miller cyclocondensation of *ortho*-phenylenediamines **1** with unsaturated carbonyl compounds. However, like in previous work by other authors, this procedure failed, possibly due to an intermolecular condensation [26]. Instead, the synthesis of 4,7-dichloro-1,10-phenanthroline derivatives **4** has been carried out using a three-step condensation of 2,2-dimethyl-1,3-dioxane-4,6-dione (Meldrum’s acid), orthoesters, and *ortho*-phenylenediamines **1** (Scheme 1) [22,23]. Compounds **4** were prepared with high yield, even on the scale of several grams. The first yield-determining step depends on the double nucleophilic addition of *ortho*-phenylenediamines (**1**) to the vinyl group of an intermediate obtained from the condensation of Meldrum’s acid and an orthoester (Scheme 2).

**Scheme 3:**
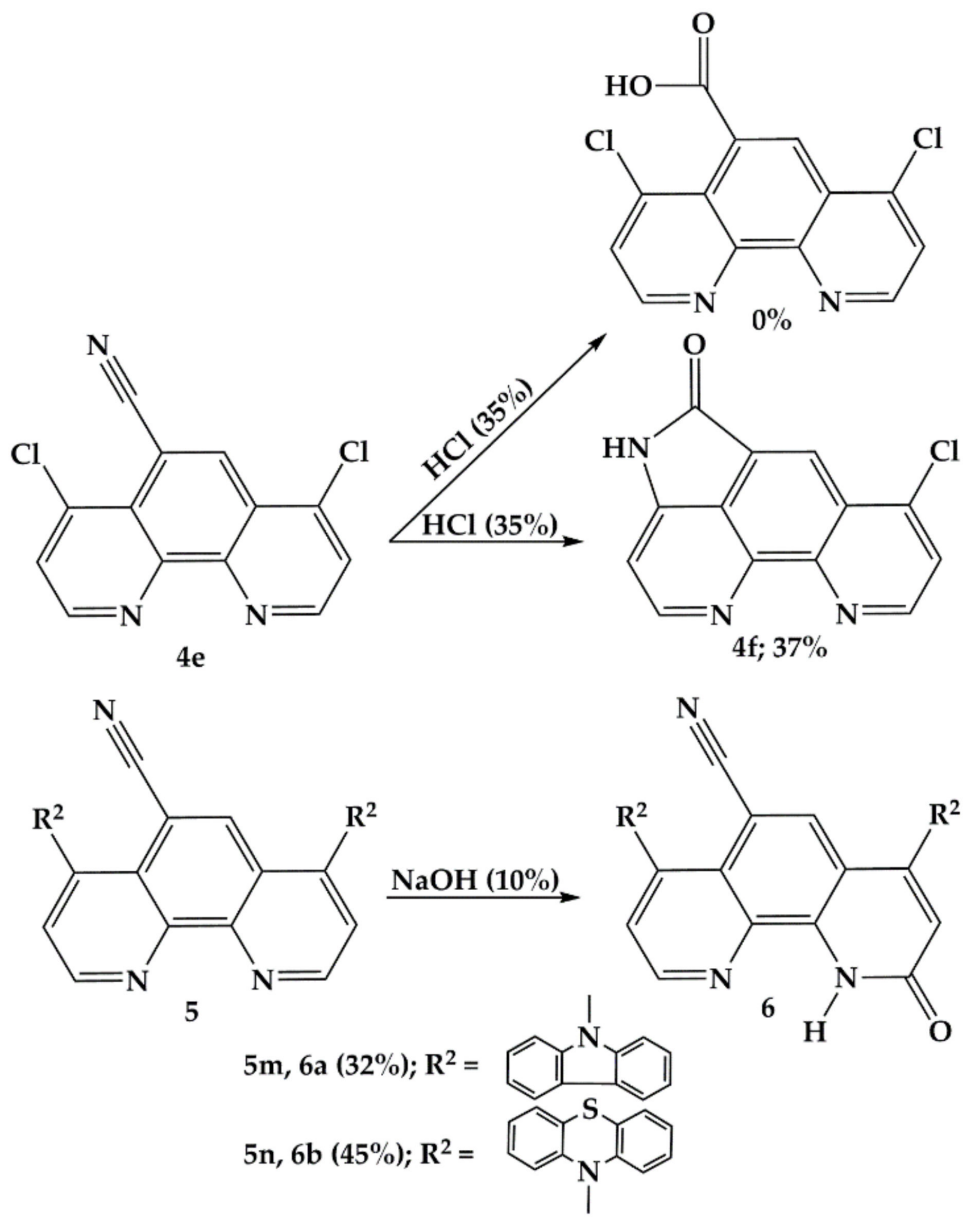
Complexity of the hydrolysis reactions of the CN group.

Anilines are poor *N*-nucleophiles. Their reactivity strongly depends on the solvents used, and substituents in their structures. In a series of experiments it was noticed that a higher isolation yield (up to 94%) was obtained for molecule **2d** which possesses a methyl group as **R^1^** substituent and hydrogen atoms as **R** (Scheme 2). Replacing the methyl group by a halogen atom (F, Cl, Br) or an electron-withdrawing group like CN or COOH in aniline decreases the isolated yield. Additionally, it is observed that the yield of products was dependent on the orthoester used. The use of trimethyl orthoformate produced the highest yields of compounds **2a**, **2b**, **2c**, **2d** and **2g**, followed by a lower yield using triethyl orthoacetate, and the lowest was triethyl orthoformate of molecules **2f**, **2h**, **2i**, **2j** and **2l**. This reactivity could be explained by increased steric interactions between Meldrum’s acid and the orthoesters, which hampers the transformation (Scheme 2). In the syntheses of heterocycles **2,** appropriate esters were produced depending on the ortho esters used as reactants. The hydrogen atom in carboxyl group was replaced by methyl or ethyl fragment giving the compounds **2m** and **2n**, respectively. It is of importance that the synthesis of products **2** was accompanied by side reactions which led to benzo[*d*]imidazole type molecules, for example during the synthesis of compound **2f** 1*H*-benzo[*d*]imidazole-6-carboxylic acid was isolated. During the next step, diketene was generated in situ from molecule **2**. This process relies on a thermally induced decarboxylation and acetone elimination. Highly reactive ketene underwent a cyclocondensation with the aryl substituent leading to compound **3** (Scheme 2). It is noticed that the rate of this reaction was not very sensitive to the presence of substituents at the C5 position (**R^1^**). This method is suitable for a variety of useful functional groups, including ester, CN, alkyl substituents and halogen atoms, with the exception however of COOH, which reacted with electrophilic ketene through disturbing cyclocondensation to give very complicated reaction mixture. Carbonyl groups at C4 and C7 positions in heterocycle **3** can be replaced by chlorine atoms to prepare 4,7-dichloro-1,10-phenanthrolines **4**. In a series of experiments, molecule **3** was treated with phosphoryl chloride giving quantitatively products **4** (Scheme 1) [22,23]. 4,7-Dichloro-1,10-phenanthrolines were obtained in high yields (ca. 68–93%) with the exception of compounds **4e** (48%), **4l** (32%) and **4m** (38%). The lower yields of these molecules could be explained by the hydrolysis of their **R^1^** substituents, i.e., the CN, COOMe and COOEt groups and formation of carboxylic acid groups [27]. To avoid the hydrolysis of CN and COOAlk groups, and a reverse reaction of the 4,7-dichloro-1,10-phenanthrolines **4** to form the 1,10-dihydro-1,10-phenanthroline-4,7-diones **3**, the excess of phosphoryl chloride should be evaporated under reduced pressure. This strategy should facilitate hydrolysis of the excess of phosphoryl chloride. It is noticed that the use of basic solutions during the hydrolysis procedure causes the competitive reversion of product **4** to molecule **3**. Hydroxide ion can substitute for chloride atom at the C4/C7 positions. Surprisingly during the isolation of compound **4e** an unexpected product **4f** was obtained, which is explained as a hydrolysis of the heterocycle **4e**. It is worth noting that during this chemical transformation 4,7-dichloro-1,10-phenanthroline-5-carboxylic acid was not isolated or identified (Scheme 3).

### 2.2. Synthesis of 4,7-diamino-1,10-phenanthrolines

The introduction of substituents **R^2^**, such as a chlorine atom in the 1,10-phenanthroline skeleton, opened opportunities for the further functionalization. Five 4,7-dipyrrolidinyl-1,10-phenanthrolines **5** with four novel structures were synthesized from heterocycles **4** by microwave-assisted nucleophilic aromatic substitution with a 10-fold excess of pyrrolidine. The nitrogen **R^2^** substituents should increase the electron density on nitrogen atoms in 1,10-phenanthrolines (Scheme 1) [24]. Reactions were carried out in a sealed vial in a microwave reactor at 130 °C (Scheme 1). MW irradiation improves the reactivity of the substitutions, shortening the reaction time to give the substitution products in better yields. In the absence of MW irradiation, the substitution reaction failed to initiate. The yields of products depend on the substituent **R^1^**, which may influence the outcome of the reaction through steric and/or electronic effect. Unexpectedly **R^1^** fluorine or chlorine atoms failed to undergo substitution. This phenomenon can be explained by a steric hindrance attributed to the already introduced pyrrolidinyl rings and too high an electron density on the carbon atom at C5 position. Substituents **R^2^** in the pyridine rings in 1,10-phenanthrolines **4** tended to undergo more S_N_Ar type substitutions due to the presence of nitrogen atoms which are able to accommodate a negative charge in the intermediate state.

Four 4,7-di(9*H*-carbazol-9-yl)-1,10-phenanthrolines (**5f**, **5g, 5h** and **5m**) and four 4,7-di(10*H*-phenothiazin-10-yl)-1,10-phenanthroline derivatives (**5i**, **5j**, **5k** and **5n**) with seven novel structures were synthesized from molecules **4** by nucleophilic aromatic substitution with 9*H*-carbazole and 10*H*-phenothiazine, respectively (Scheme 1). Reagents were stirred for sixteen hours under reflux. The chemistry was based on inexpensive, commercially available reagents and easily synthesized heterocycles **4** which possess methyl, CN, fluorine or hydrogen atom as **R^1^**. The yields of 9*H*-carbazole derivatives **5** were lower than for 10*H*-phenothiazine derivatives. In all cases the purification of 9*H*-carbazole derivatives **5** required chromatographic methods to receive pure products. However, the purification of 10*H*-phenothiazine could be carried out by crystallization from the mixture of CH_2_Cl_2_ (or THF) and hexane to afford products even on a multigram scale. The highest yield and less complex reaction mixture of 10*H*-phenothiazine derivatives can be attributed to the better nucleophilic properties of 10*H*-phenothiazine anion than 9*H*-carbazole. Wu et al. obtained the X-ray structure of 4,7-di(10*H*-phenoxazin-10-yl)-1,10-phenanthroline [28], which was cocrystallized with THF as a guest molecule located in the crystalline frameworks. The THF molecule is surrounded by two 10*H*-phenoxazine rings and 1,10-phenanthroline core. ^1^H-NMR and ^13^C-NMR studies showed that the presence of signals from THF for 10*H*-phenothiazine derivatives **5i**, **5j** and **5n** (Supporting Information). It is worth noting that in the case of molecule **5k**, with a methyl group as **R^1^**, no residual THF signals have been detected. 9*H*-Carbazole derivatives have shown similar supramolecular inclusion processes to compounds **5i**, **5j** and **5n**, in which the solvent is located in a cage made by 1,10-phenanthroline derivatives, however, with smaller intensity.

Yields of selected 1,10-phenanthrolines **4** and **5** are summarized in Scheme 1. 4,7-Di(10*H*-phenothiazin-10-yl)-1,10-phenanthroline derivatives were obtained with yields in the range 84-96%. 4,7-Disubstituted-1,10-phenanthrolines with a carboxylic acid group as **R^1^** substituent should be readily soluble in waters. These bifunctional compounds should mimic amino acids or nicotinic acid. Therefore, in order to synthesize appropriate carboxylic acids we carried out the hydrolysis of the CN group in molecule **4e**, using an excess of acid (ca. 50.0 equiv. HCl) or excess of base (ca. 50.0 equiv. NaOH) in the case of 4,7-di(9*H*-carbazol-9-yl)-1,10-phenanthroline-5-carbonitrile (**5m**) and 4,7-di(10*H*-phenothiazin-10-yl)-1,10-phenanthroline-5-carbonitrile (**5n**) under the applied conditions. It is shown in Scheme 3 that in both cases the reactions does not lead to the formation of the expected 4,7-dichloro-1,10-phenanthroline-5-carboxylic acid or related molecules as was observed for the synthesis of 1,10-phenanthroline-2-carboxylic acid [27]. We isolated unexpected product **4f**. The hydrolysis of heterocycles **5m** and **5n** lead to other surprising compounds, namely 4,7-di(9*H*-carbazol-9-yl)-9-oxo-9,10-dihydro-1,10-phenanthroline-5-carbonitrile (**6a**) and 4,7-di(10*H*-pheno- thiazin-10-yl)-1,10-phenanthroline-5-carbonitrile (**6b**). Both molecules were possibly obtained through a reaction of oxidative nucleophilic substitutions of hydrogen (ONSH) of the type S_N_Ar^H^, with hydroxide ion as a nucleophile and in the presence of air as oxidized reagent [29,30]. In both cases nucleophile attacks were selective only at C2 rather than at the C9 position. The natural atomic charges show that both heterocycles possess greater electron density on the C2 (0.040 for compound **5m**, and 0.038 for compound **5n**) than on the C9 position (0.046 for compound **5m** and 0.044 for compound **5n**) (Figures S1 and S2; Supporting Information). Both compounds **6a** and **6b** were isolated in a crystalline form, and their structures were characterized by X-ray crystallography. The prepared 4,7-dichloro-1,10-phenanthrolines **4** tend to undergo a fragmentation with the initial loss of chloride, followed by the loss of a second chloride and further decomposition, with the exception of molecule **4j**, which first eliminates bromide.

### 2.3. X-Ray Studies

Crystals of compounds **5d**, **6a** and **6b** were mounted in turn on a Gemini A Ultra Oxford Diffraction automatic diffractometer equipped with a CCD detector used for data collection. X-ray intensity data were collected with graphite monochromated Mo*K*_α_ radiation at room temperature, with *ω* scan mode. Details concerning crystal data and refinement are gathered in Table 1 in ESI. Lorentz, polarization and empirical absorption correction using spherical harmonics implemented in SCALE3 ABSPACK scaling algorithm were applied [31]. The structures were solved by a direct method and subsequently completed by a difference Fourier recycling. All the non-hydrogen atoms were refined anisotropically using full-matrix, least-squares techniques. The Olex2 [32] and SHELXS, SHELXL [33] programs were used for all the calculations. Atomic scattering factors were incorporated in the computer programs.

**Figure 1:**
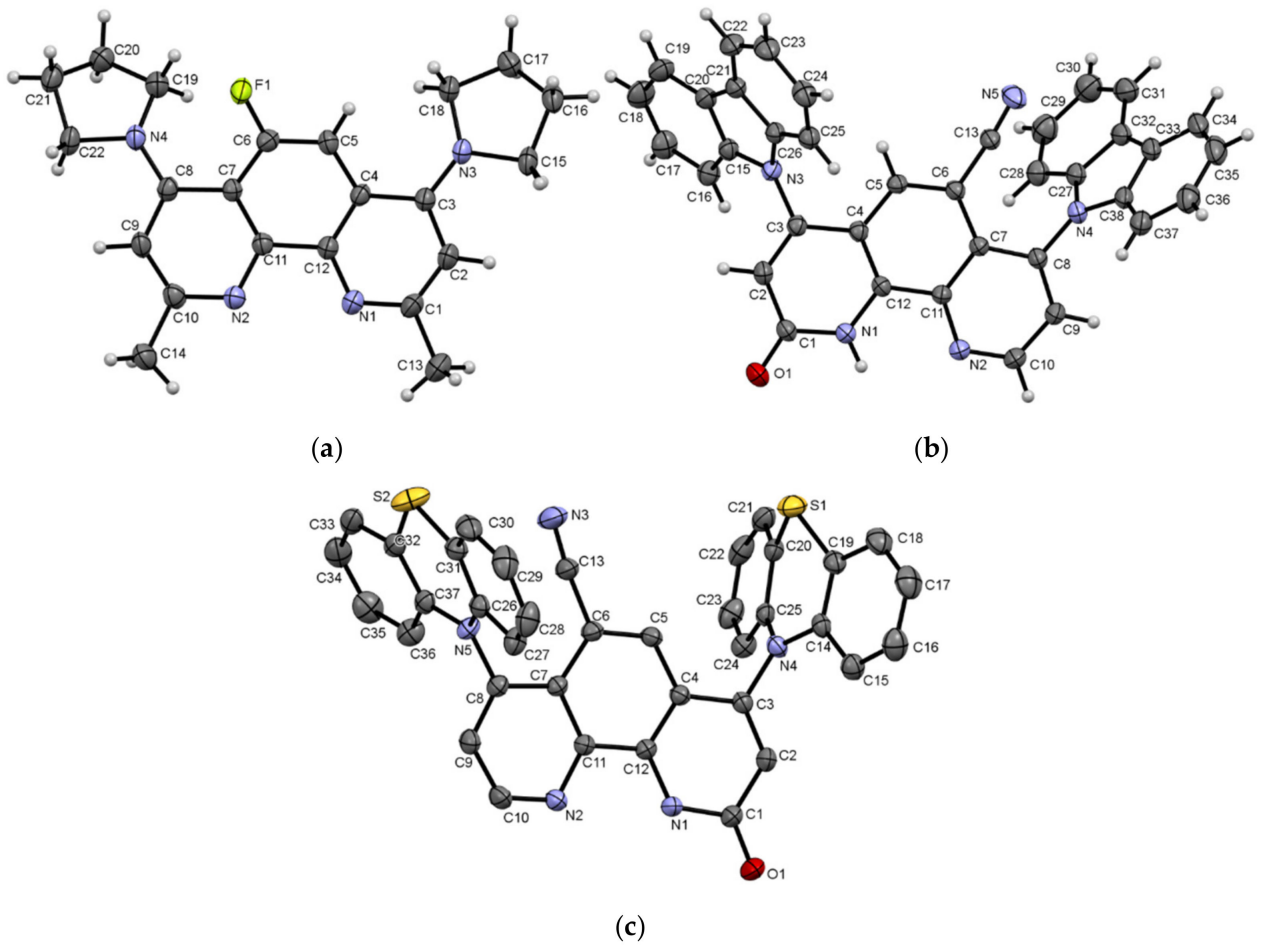
(**a**) ORTEP drawings of compound **5d** with 30% probability. The solvent molecules and fluorine disorder were omitted for clarity. (**b**) ORTEP drawings of compound **6a** with 30% probability. The solvent molecules were omitted for clarity. (**c**) ORTEP drawings of compound **6b** with 30% probability. Hydrogen atoms and solvent molecules are omitted for clarity.

The performed investigations on the X-ray studies were inspired by the work of G. Zucchi et al. and K.J. Shaffer et al. [25,28]. Presented here compounds crystalize in the triclinic *P*–1 space group as a solvate with two CHCl_3_ molecules **5d** and two THF and one methanol molecules in the case of compounds **6a** and **6b** crystalizes in monoclinic *P*2_1_/c space group with two THF molecules. The molecular structures are displayed as ORTEP representation in Figure 1a–c.

The intra- and intermolecular hydrogen bonds in the structures of compounds **5d** and **6a**, **6b** are listed in Table S2 in the Supporting Information. The 1,10-pPhenanthroline moiety is in a planar orientation with a ring puckering amplitude of 0.105 in molecule **5d** and 0.05 and 0.07 in the cases of compounds **6a** and **6b**, respectively. In the structure of heterocycle **5d** C-Cl…π interactions are visible, while in molecules **6a** and **6b** π…π stacking occurs between phenanthroline rings with centroid-centroid distances of 3.89 Å and 3.78 Å and shift distances of 1.91 Å and 1.07 Å, respectively (Table S3; Supporting Information). The interaction between the nitrile C(13)-N(3) group causes flattening of 10*H*-phenothiazine ring in the position C7. The 10*H*-phenothiazine ring in position C4 shows a characteristic, for this type of heterocyclic compounds, deviation of the sulfur atom position from the plane delimited by the benzene rings. The structures of compounds **5d**, **6a** and **6b** are constitutionally related to 4,7-di(10*H*-phenoxazin-10-yl)-1,10-phenanthroline and 4,7-di(9*H*-carbazol-9-yl)-1,10-phenanthroline [25,28]. Similarly to two other compounds which are composed of a 1,10-phenanthroline core and two peripheral amino substituents, the 9*H*-carbazol-9-yl and 10*H*-phenoxazin-10-yl substituents are strongly twisted in relation to the 1,10-phenanthroline rings with a larger angle (~87°) between 1,10-phenanthroline and substituent planes in the position C7 than C4 (~83°). It is noticed that bond lengths between carbon atoms at C4/3 and C7/8 positions and pyrrolidinyl, 9*H* carbazol-9-yl and 10*H*-phenothiazin-10-yl groups 1.360 Å, 1.420 Å and 1.434(3) Å, respectively, are comparable to the values of 1.42 Å in 4,7-di(9*H*-carbazol-9-yl)-1,10-phenanthroline or 1.43 Å for 4,7-di(10*H*-phenoxazin-10-yl)-1,10-phenanthroline. The π-conjugated system derived from 1,10-phenanthroline core and two peripheral 9*H*-carbazole or 10*H*-phenoxazine rings showed less extended delocalization than that of pyrrolidinyl group over the entire π-conjugated systems, because the lone pair of nitrogen atom and the 1,10-phenanthroline core are not in the same plane. 9*H*-carbazole and 10*H*-phenothiazine rings are perpendicular to the 1,10-phenanthroline moiety [25,28].

### 2.4. NMR Studies

^13^C CP/MAS NMR spectra of compounds **4** and **5** displayed readily discernible aliphatic carbon signals such as methyl, and distinguishable resonance lines attributed to aromatic carbons. In general, the signals in the solution and in the solid state are in good agreement (Tables S5 and S6; Supporting Information). To investigate the character of nitrogen atoms which are responsible for their bidentate coordination abilities, studies using the ^15^N CP/MAS NMR technique were carried out, which is a highly sensitive probe and allows being monitored even in subtle changes in molecular constitution and electronic structures. Due to the symmetry of **4e**, **5c** and **5f** molecules only one or two lines are presence in ^15^N CP/MAS NMR spectrum, respectively (Table S7; Supporting Information). Comparing the experimental CP/MAS ^15^N chemical shifts of selected heterocyles **4** and **5**, the influence of substituents originated from chlorine atoms, methyl group and *N*-donor fragments i.e., NH_2_, pyrrolidine, 9*H*-carbazole and 10*H*-phenothiazine could be observed. It is worth noting that the ^15^N chemical shift of a free 1,10-phenanthroline (or 2,9-dimethyl-1,10-phenanthroline) molecule is equal to −74.4 (or −80.2) ppm in the absolute scale [34,35]. A significant impact on the ^15^N chemical shifts of pyridine nitrogen has been seen with pyrrolidine and 10*H*-phenothiazine substituents (Table S7; Supporting Information). For pyridine rings, the dominant contribution to nitrogen shielding is associated with a n → π* excitation, and low transition energies correlate with large deshieldings. Interestingly, 9*H*-carbazole and 10*H*-phenothiazine fragments have higher impact on one of the two pyridine nitrogen atoms. One is significantly deshielded (downfield effect, larger δ) with 11.14 ppm of difference for molecule **5k** or 23.78 ppm of difference for compound **5h** (Table S7). In contrast, *N*-donor substituents i.e., NH_2_, pyrrolidine, 9*H*-carbazole and 10*H*-phenothiazine are characterized by the more negative value of ^15^N chemical shift (−249.48 to −317.08 ppm).

### 2.5. Cyclic Voltammetry

**Figure 2:**
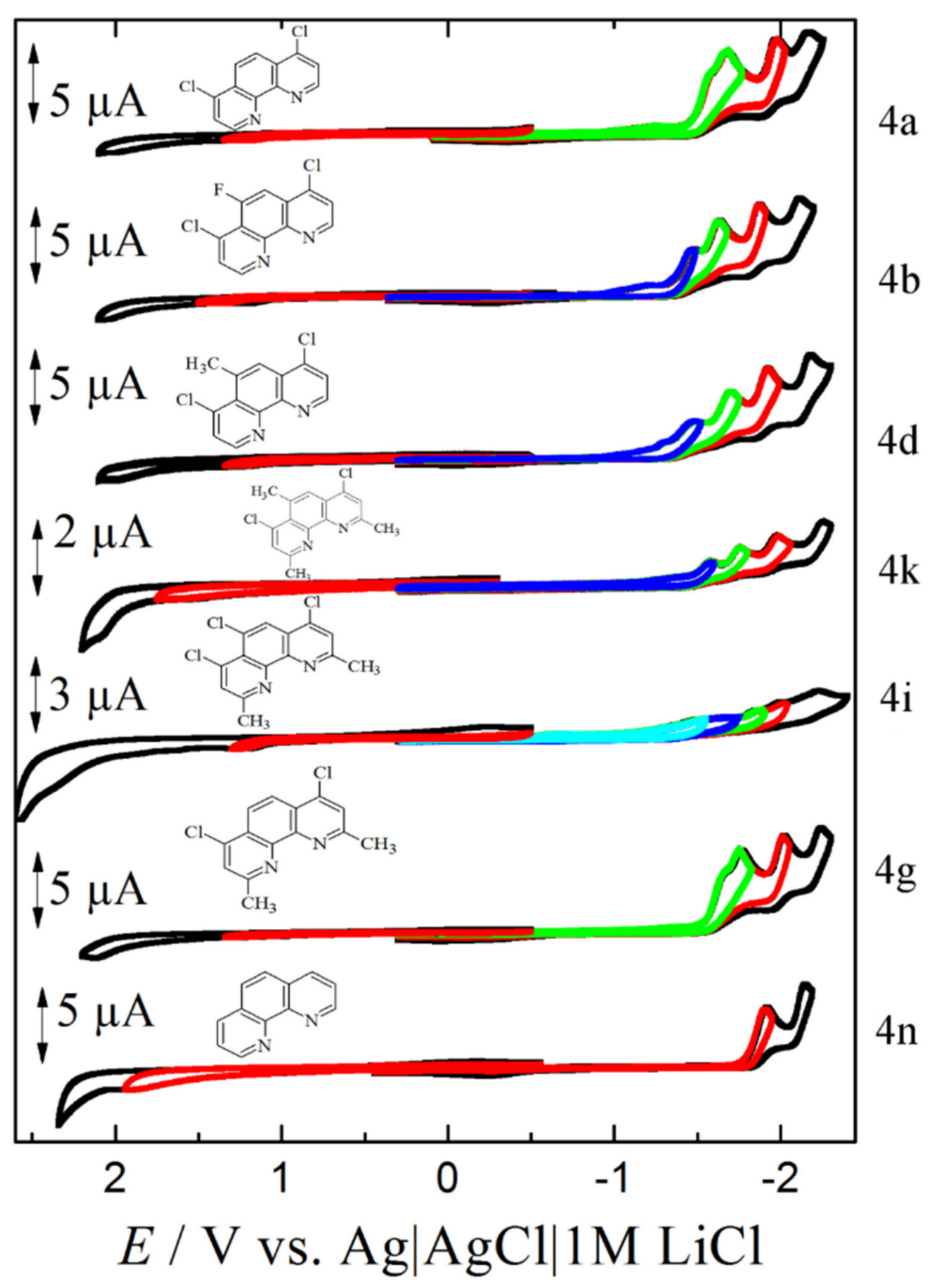
Cyclic voltammograms of 0.60 mM **4a**, **4g**, **4i**, **4n** and 0.50 mM **4b**, **4d** and 0.12 mM **4k** in acetonitrile at 0.1 V/s (0.1 M TBAPF_6_).

**Figure 3:**
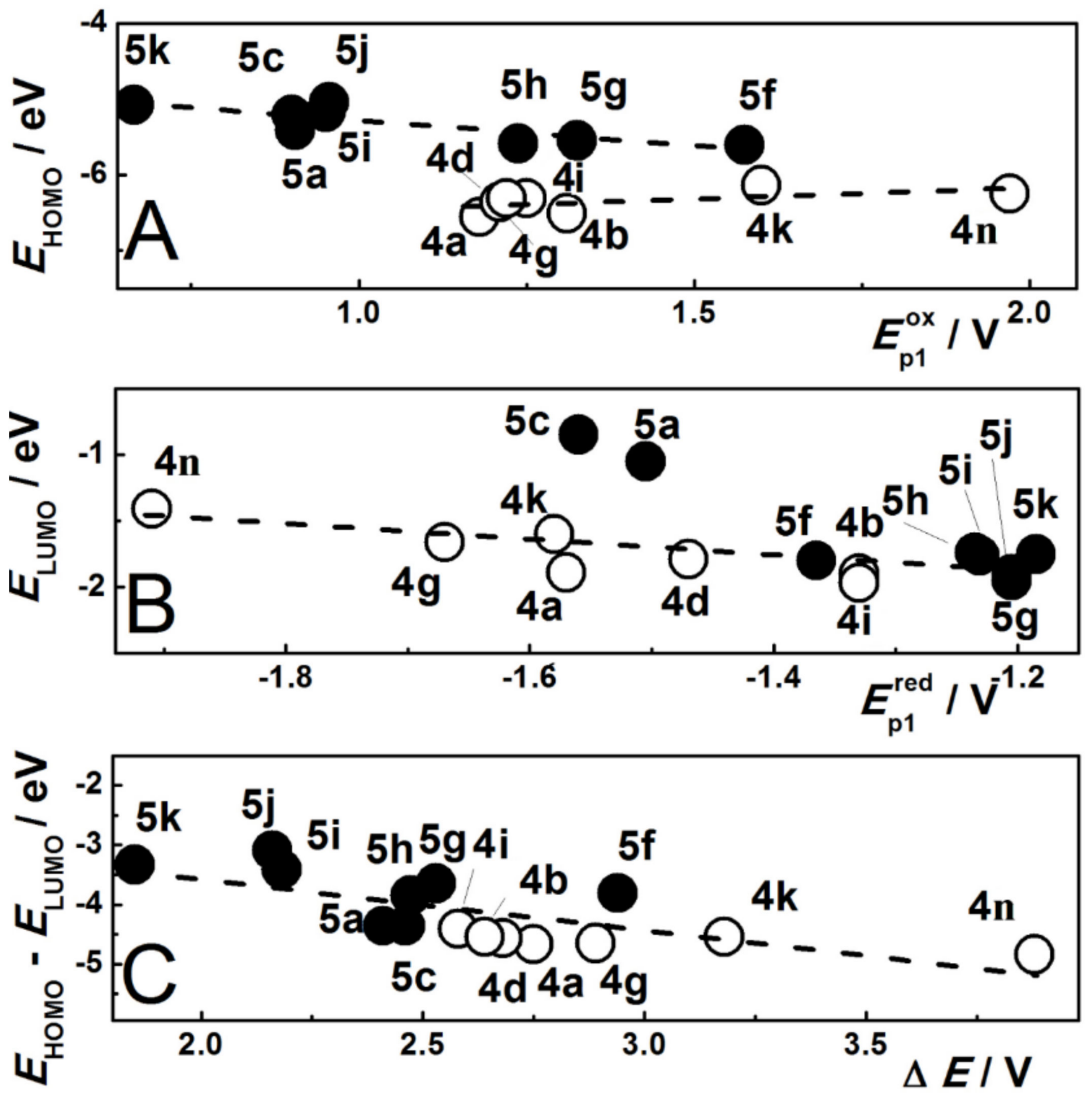
Dependence of HOMO (**A**) and LUMO (**B**) energies on the experimental oxidation and reduction potentials, respectively. Panel (**C**) shows a dependence of HOMO and LUMO energies difference on electrochemical gap, *E*_ox_ and *E*_red_.

The electrochemical behaviour of selected substituted 1,10-phenanthroline derivatives in acetonitrile was studied by cyclic voltammetry on a glassy carbon electrode. Compounds **4** substituted by chlorine atoms (**R^2^** substituent) yield rather complex cyclic voltammograms mainly in the negative potential regions (Figure 2). In the case of all compounds **4** the first reduction wave corresponds to the formation of anion radical and according to literature the subsequent cleavage of chlorine from position **R^2^** in the overall ECE process follows [36,37]. The compounds **4b** and **4i** were found to be the most easily reduced because of the presence of chlorine and fluorine atoms as **R^1^**. In the case of compound **4i** the first reduction step may be attributed to the cleavage of chlorine (**R^1^**) from primarily formed radical anion. Calculated HOMO and LUMO spatial distributions of sterically symmetrical compounds are shown in Table 1 and results for other electrochemically investigated compounds are summarized in Tables S8 and S9 in the Supporting Information. A linear relationship of LUMO energy on reduction potential was observed for compounds from series **4** and **5** (except compounds **5a** and **5c**) showing that the first reduction step proceeds on 1,10-phenanthroline rings and an anion radical is formed (Figure 3). Compounds **5a** and **5c** are substituted with pyrrolidine and their reduction may follow another mechanism. These two compounds differ from the others also by high values of their dipole moments as indicated in Table 2. The substituent effect on the reduction and oxidation potentials of selected compounds was summarized in Table 2. Methyl group as **R** substituent significantly influenced reduction potential of compounds towards more negative values (compounds **4g**, **4k**, **5c** compare to **4a**, **4d**, **5a**, respectively). The oxidation of all compounds **4** may lead to the formation of cation radicals [38]. The dependences of the highest occupied molecular orbital energy on the first oxidation potential were linear for both series of compounds, substituents **R^2^** with chlorine atoms **4** and *N*-heterocycles **5**. Their independence is related to their different oxidation mechanism, resulting from the different electroactive site visible from the spatial distribution of HOMO orbitals (Table 1). Oxidation of phenothiazine is known in the literature [39,40]. Detailed investigation of reduction and oxidation mechanism of both series will be further plan of our research. Figure 3C shows a linear relationship between the energy difference of HOMO and LUMO orbitals and the potential gap Δ*E* obtained experimentally.

## 3. Materials and Methods

### 3.1. Materials

All experiments were carried out in an atmosphere of dry argon and flasks were flame dried. Solvents were dried by usual methods (diphenyl ether, diethyl ether and THF over benzophenone ketyl, CHCl_3_ and CH_2_Cl_2_ over P_4_O_10_, hexane over sodium-potassium alloy) and distilled. Chromatographic purification was carried out on silica gel 60 (0.15–0.3 mm, Macherey-Nagel GmbH & Co. KG, Düren, Germany). Sodium hydride (dry, 95%), trimethyl orthoformate, triethyl orthoformate, triethyl orthoacetate, trimethyl orthoacetate, 1,10-phenanthroline (**4n**), 1,10-phenanthrolin-5-amine (**5l**), 9*H*-carbazole, pyrrolidine, 10*H*-phenothiazine, Meldrum’s acid, benzene-1,2-diamine, 4-fluorobenzene-1,2-diamine, 4-chlorobenzene-1,2-diamine, 4-bromobenzene-1,2-diamine, 4-methylbenzene-1,2-diamine, 3,4-diaminobenzonitrile and 3,4-diaminobenzoic acid were purchased from Sigma–Aldrich (Poznan, Poland), and were used without further purification.

### 3.2. Instrumentation

NMR spectra were obtained with Avance 400, 500 and 600 spectrometers (Bruker, Billerica, MA, USA) operating at 600.2, 500.2 or 400.1 MHz (^1^H), 150, 125.78 or 100.5 MHz (^13^C) and 470.5 MHz (^19^F) at 21 °C, and a Bruker Avance 400WB; 100.65 MHz for ^13^C, 40.55 MHz for ^15^N. Chemical shifts referenced to ext. TMS (^1^H, ^13^C), CFCl_3_ (^19^F) and ext. DSS (^1^H, ^13^C), or using the residual CHCl_3_ signal (δ_H_ 7.26 ppm) and CDCl_3_ (δ_C_ 77.1 ppm) as internal references for ^1^H and ^13^C NMR, respectively. Coupling constants are given in Hz. High-resolution solid-state Cross-Polarization (CP) ^13^C- and ^15^N-NMR experiments were made with magic-angle spinning (MAS) at 5 and 7 kHz. Experiments were done with a 4000 scans, a contact time of 2 ms, a repetition time of 25 s, using 4 and 7 mm Bruker probes. For GC-MS a 7890A gas chromatograph (Agilent Technologies, Wilmington, DE, USA) equipped with a MS (70 eV) 5975 EI/CI MSD, and a 7693 autosampler with an Agilent HP-5MS capillary column (30 m × 250 μm × 0.25 μm)-press. 127.5 kPa, total flow 19 mL/min, col. flow 2 mL/min, split - 7:1, temp. prog. (70 °C - hold 0.5 min, 70–290 °C/25 °C/min., 290 °C - hold 6 min) was used. The LCMS-IT-TOF analysis was performed on an Agilent 1200 Series binary LC system coupled to a micrOTOF-Q system mass spectrometer (Bruker Daltonics, Brema, Germany). High-resolution mass spectrometry (HRMS) measurements were performed using a Synapt G2-Si mass spectrometer (Waters, New Castle, DE USA) equipped with an ESI source and quadrupole-time-of-flight mass analyser. To ensure accurate mass measurements, data were collected in centroid mode and mass was corrected during acquisition using leucine enkephalin solution as an external reference (Lock-Spray^TM^). The results of the measurements were processed using the MassLynx 4.1 software (Waters) incorporated within the instrument. A Nicolet iS50 FTIR spectrometer was used for recording spectra in the IR range 4000–400 cm^−1^. FTIR spectra were recorded on a Perkin Elmer (Schwerzenbach, Switzerland) spectrophotometer in the spectral range 4000–450 cm^−1^ with the samples in the form of KBr pellets. Elementary analysis was performed using Vario EL III apparatus (Elementar, Langenselbold, Germany). Melting points were determined on MPA100 OptiMelt melting point apparatus (Stanford Research Systems, Sunnyvale, CA USA) and are uncorrected.

### 3.3. General Procedures for Synthesis of 4,7-dichloro-1,10-phenanthrolines 

These syntheses were based on procedures described in the literature [22,23]. 

#### 3.3.1. Step A

Triethyl orthoformate or trimethyl orthoformate were used in the synthesis of **2a**, **2b**, **2c**, **2d**, **2e**, **2f** and **2g** and triethyl orthoacetate or trimethyl orthoacetate for **2h**, **2i**, **2j**, **2k**, **2l**, **2m**, **2n** and **2o**. The orthoester (triethyl orthoformate, triethyl orthoacetate, trimethyl orthoformate or trimethyl orthoacetate, 330.0 mmol) and Meldrum’s acid (1.7 g, 12.0 mmol) was brought to a gentle reflux for 15 min. The resulting greenish solution was cooled to 80 °C and the appropriate benzene-1,2-diamine **1** (5.5 mmol) was added portionwise (caution: exothermic reaction). The resulting mixture was stirring up to reflux for 2 h, and left under r.t. for 16 h. Subsequently, diethyl ether or hexane was added and the solution was cooled to −35 °C where a precipitate formed. The precipitate was filtered off, washed with diethyl ether (4 × 100 mL) and dried to afford a solid:

*1,2-Bis-[(2,2-dimethyl-4,6-dioxo-1,3-dioxan-5-ylidenemethyl)amino]benzene* (**2a**) [23,41]; white solid 2.0 g (4.7 mmol, 86%); m.p_dec_. = 207.6 °C.

*5,5′-(((4-Fluoro-1,2-phenylene)bis(azanediyl))bis(methaneylylidene))bis(2,2-dimethyl-1,3-dioxane-4,6-dione)* (**2b**); white; 2.0 g (4.6 mmol, 83%); m.p_dec_. = 216 °C; ^1^H-NMR (CDCl_3_; 400.2 MHz) δ = 1.73 (s, 6H, 2CH_3_), 1.74 (s, 6H, 2CH_3_), 7.06–7.12 (dt, ^3^*J*_H,H_ = 8.1 Hz, ^4^*J*_F,H_ = 2.6 Hz, 1H, aromatic), 7.20 (dd, ^3^*J*_F,H_ = 8.7 Hz, ^4^*J*_H,H_ = 2.5 Hz, 1H, aromatic), 7.38 (dd, ^3^*J*_F,H_ = 8.9 Hz, ^3^*J*_H,H_ = 5.1 Hz, aromatic), 8.38 (d, ^3^*J*_H,H_ = 13.5 Hz, 1H, vinyl), 8.52 (d, ^3^*J*_H,H_ = 13.4 Hz, 1H, vinyl), 11.15 (d, ^3^*J*_H,H_ = 13.5 Hz, 1H, NH), 11.42 (d, ^3^*J*_H,H_ = 13.3 Hz, 1H, NH); ^13^C{^1^H}-NMR (CDCl_3_; 125.78 MHz) δ = 27.2, 27.3, 89.6, 90.3, 105.8 (d, ^3^*J*_C,F_ = 14.3 Hz), 107.4 (d, ^2^*J*_C,F_ = 26.8 Hz), 115.0 (d, ^2^*J*_C,F_ = 22.9 Hz), 124.9 (d, ^4^*J*_C,F_ = 9.5 Hz), 126.9, 133.3 (d, ^3^*J*_C,F_ = 10.1 Hz), 161.1, 155.1 (d, ^1^*J*_C,F_ = 342.9 Hz), 162.9, 163.2, 165.4, 165.5; ^19^F{^1^H}-NMR (CDCl_3_; 470.5 MHz) δ = −108.94; ^19^F- NMR (CDCl_3_; 470.5 MHz) δ = −108.94 (dd, ^3^*J*_F,H_ = 12.9 Hz, ^3^*J*_F,H_ = 7.8 Hz); UV-Vis (methanol; λ [nm] (logε)): 329 (4.48), 292 (4.56), 212 (4.43). 

*5,5′-(((4-Chloro-1,2-phenylene)bis(azanediyl))bis(methaneylylidene))bis(2,2-dimethyl-1,3-dioxane-4,6-dione)* (**2c**) [23]; white; 2.1 g (4.6 mmol, 84%); m.p_dec_. = 202.7 °C; UV-Vis (methanol; λ [nm] (logε)): 332 (4.59), 296 (4.75), 223 (4.47), 206 (4.46). 

*5,5′-(((4-Methyl-1,2-phenylene)bis(azanediyl))bis(methaneylylidene))bis(2,2-dimethyl-1,3-dioxane-4,6-dione)* (**2d**); white; 2.2 g (5.2 mmol, 94%) [41]; m.p_dec_. = 212 °C; UV-Vis (methanol; λ [nm] (logε)): 330 (4.85), 296 (4.93), 221 (4.83). 

*3,4-Bis(((2,2-dimethyl-4,6-dioxo-1,3-dioxan-5-ylidene)methyl)amino)benzonitrile* (**2e**); beige, 1.7 g (3.8 mmol, 69%); m.p_dec_. = 250 °C; ^1^H-NMR (DMSO-*d6*; 400.2 MHz) δ = 1.68 (s, 6H, 2CH_3_), 1.68 (s, 6H, 2CH_3_), 7.81-7.84 (m, 2H, aromatic), 8.16 (dd, ^3^*J*_H,H_ = 4.3 Hz, ^4^*J*_H,H_ = 0.8 Hz, 1H, aromatic), 8.33 (d, ^3^*J*_H,H_ = 14.1 Hz, 1H, vinyl), 8.41 (d, ^3^*J*_H,H_ = 13.9 Hz, 1H, vinyl), 11.33 (d, ^3^*J*_H,H_ = 14.1 Hz, 1H, NH), 11.43 (d, ^3^*J*_H,H_ = 14.0 Hz, 1H, NH); ^13^C{^1^H}-NMR (DMSO-*d6*; 125.78 MHz) δ = 26.5, 26.6, 88.3, 88.8, 104.1, 104.3, 110.0, 117.4, 122.7, 128.0, 132.0, 133.6, 137.5, 155.6, 157.4, 162.3, 162.5, 163.5, 163.6; UV-Vis (methanol; λ [nm] (logε)): 342 (4.11), 296 (4.19), 219 (4.00). 

*3,4-Bis(((2,2-dimethyl-4,6-dioxo-1,3-dioxan-5-ylidene)methyl)amino)benzoic acid* (**2f**); beige, 0.8 g (1.8 mmol, 32%); m.p_dec_. = 225 °C; ^1^H NMR (CDCl_3_; 400.2 MHz) δ = 1.77 (s, 6H, 2CH_3_), 1.79 (s, 6H, 2CH_3_), 7.50 (d, ^3^*J*_H,H_ = 8.5 Hz, 1H, aromatic), 8.14 (d, ^3^*J*_H,H_ = 8.9 Hz, 1H, aromatic), 8.50 (s, 1H, aromatic), 8.59 (d, ^3^*J*_H,H_ = 13.1 Hz, 1H, vinyl), 8.99 (d, ^3^*J*_H,H_ = 12.7 Hz, 1H, vinyl), 11.51 (d, ^3^*J*_H,H_ = 12.6 Hz, 1H, NH), 11.66 (d, ^3^*J*_H,H_ = 12.9 Hz, 1H, NH); ^1^H-NMR (DMSO-*d6*; 500.2 MHz) δ = 1.68 (s, 6H, 2CH_3_), 1.68 (s, 6H, 2CH_3_), 7.74 (d, ^3^*J*_H,H_ = 8.4 Hz, 1H, aromatic), 7.96 (dd, ^3^*J*_H,H_ = 8.3 Hz, ^4^*J*_H,H_ = 1.7 Hz, 1H, aromatic), 8.03 (d, ^4^*J*_H,H_ = 1.7 Hz, 1H, aromatic), 8.24 (d, ^3^*J*_H,H_ = 14.2 Hz, 1H, vinyl), 8.40 (d, ^3^*J*_H,H_ = 14.1 Hz, 1H, vinyl), 11.35 (d, ^3^*J*_H,H_ = 14.3 Hz, 1H, NH), 11.44 (d, ^3^*J*_H,H_ = 14.1 Hz, 1H, NH); ^13^C{^1^H}-NMR (DMSO-*d6*; 125.78 MHz) δ = 26.50, 26.52, 87.8, 88.2, 104.0, 104.1, 121.9, 125.1, 129.3, 130.0, 133.1, 137.2, 155.7, 157.5, 162.4, 162.7, 163.4, 163.6, 165.9, 168.2; UV-Vis (methanol; λ [nm] (logε)): 340 (3.96),303 (3.87) 274 (3.82), 220 (4.00).

*Methyl 3,4-bis(((2,2-dimethyl-4,6-dioxo-1,3-dioxan-5-ylidene)methyl)amino)benzoate* (**2g**); beige, 1.6 g (3.5 mmol, 63%); m.p_dec_. = 225 °C; ^1^H-NMR (DMSO-*d6*; 500.2 MHz) δ = 1.68 (s, 6H, 2CH_3_), 1.69 (s, 6H, 2CH_3_), 4.00 (s, 3H, OCH_3_), 7.39 (d, ^3^*J*_H,H_ = 8.3 Hz, 1H, aromatic), 7.76 (dd, ^3^*J*_H,H_ = 8.2 Hz, ^4^*J*_H,H_ = 1.7 Hz, 1H, aromatic), 8.18 (d, ^4^*J*_H,H_ = 1.6 Hz, 1H, aromatic), 8.24 (d, ^3^*J*_H,H_ = 14.5 Hz, 1H, vinyl), 8.81 (d, ^3^*J*_H,H_ = 14.7 Hz, 1H, vinyl), 11.40 (d, ^3^*J*_H,H_ = 14.1 Hz, 2H, NH), 11.84 (d, ^3^*J*_H,H_ = 14.8 Hz, 2H, NH); ^13^C{^1^H}-NMR (DMSO-*d6*; 125.78 MHz) δ = 26.47, 26.52, 54.2, 87.2, 87.7, 104.39, 104.38, 116.0, 119.9, 127.2, 128.0, 131.3, 140.1, 151.3, 159.3, 162.5, 164.7, 166.6, 172.6; UV-Vis (methanol; λ [nm] (logε)): 340 (3.96),300 (3.87) 275 (3.82), 220 (4.00). 

*5,5′-((1,2-Phenylenebis(azanediyl))bis(ethan-1-yl-1-ylidene))bis(2,2-dimethyl-1,3-dioxane-4,6-dione)* (**2h**) [22,23]; white; 1.2 g (2.8 mmol, 51%); m.p_dec_. = 189.6 °C. 

*5,5′-(((4-Fluoro-1,2-phenylene)bis(azanediyl))bis(ethan-1-yl-1-ylidene))bis(2,2-dimethyl-1,3-dioxane-4,6-dione)* (**2i**); white, 0.9 g (2.0 mmol, 36%); m.p_dec_._._ = 218.0 °C; ^1^H-NMR (CDCl_3_; 400.2 MHz) δ = 1.70 (s, 6H, 2CH_3_), 1.71 (s, 6H, 2CH_3_), 2.50 (s, 3H, CH_3_), 2.58 (s, 3H, CH_3_), 7.12 (dd, ^3^*J*_H,F_ = 8.2 Hz, ^4^*J*_H,H_ = 2.3 Hz, 1H, aromatic), 7.18-7.20 (m, 1H, aromatic), 7.35 (dd, ^3^*J*_H,F_ = 8.7 Hz, ^3^*J*_H,H_ = 5.4 Hz, 1H, aromatic), 12.68 (s, 1H, NH), 12.85 (s, 1H, NH); ^13^C{^1^H}-NMR (CDCl_3_; 125.78 MHz) δ = 19.6, 19.7, 26.7, 26.7, 87.6, 88.0, 103.4, 103.5, 115.4 (d, ^2^*J*_C,F_ = 24.8 Hz), 116.4 (d, ^2^*J*_C,F_ = 22.4 Hz), 128.5 (d, ^4^*J*_C,F_ = 3.7 Hz), 129.7 (d, ^3^*J*_C,F_ = 9.5 Hz), 134.1 (d, ^3^*J*_C,F_ = 10.3 Hz), 161.8 (d, ^1^*J*_C,F_ = 252.9 Hz), 162.3, 162.5, 167.7, 167.6, 172.8, 173.5; ^19^F{^1^H}-NMR (CDCl_3_; 470.5 MHz) δ = −108.53; ^19^F-NMR (CDCl_3_; 470.5 MHz) δ = −108.53 (dd, ^3^*J*_F,H_ = 13.3 Hz, ^4^*J*_F,H_ = 7.7 Hz); UV-Vis (methanol; λ [nm] (logε)): 311 (4.30), 283 (4.66), 226 (4.38), 201 (4.27). 

*5,5′-(((4-Chloro-1,2-phenylene)bis(azanediyl))bis(ethan-1-yl-1-ylidene))bis(2,2-dimethyl-1,3-dioxane-4,6-dione)* (**2j**); white, 1.4 g (2.9 mmol, 52%); mp_dec._ = 203.8 °C; ^1^H-NMR (CDCl_3_; 400.2 MHz) δ = 1.71 (s, 12H, 4CH_3_), 2.53 (s, 3H, CH_3_), 2.56 (s, 3H, CH_3_), 7.30 (d, ^3^*J*_H,H_ = 8.5 Hz, 1H, aromatic), 7.38 (d, ^4^*J*_H,H_ = 1.6 Hz, 1H, aromatic), 7.48 (dd, ^3^*J*_H,H_ = 8.5 Hz, ^4^*J*_H,H_ = 1.8 Hz, 1H, aromatic), 12.76 (s, 1H, NH), 12.81 (s, 1H, NH); ^13^C{^1^H}-NMR (CDCl_3_; 100.5 MHz) δ = 19.7, 19.7, 26.7, 87.9, 88.0, 103.5, 103.5, 128.1, 129.0, 129.5, 131.1, 133.6, 135.0, 162.3, 162.4, 167.7, 167.7, 173.0, 173.1; UV-Vis (methanol; λ [nm] (logε)): 315 (4.36), 285 (4.74), 226 (4.46), 202 (4.38).

*5,5′-(((4-Bromo-1,2-phenylene)bis(azanediyl))bis(ethan-1-yl-1-ylidene))bis(2,2-dimethyl-1,3-dioxane-4,6-dione)* (**2k**); white, 0.9 g (1.8 mmol, 32%); m.p_dec_. = 204.6 °C; ^1^H-NMR (CDCl_3_; 400.2 MHz) δ = 1.71 (s, 12H, 4CH_3_), 2.53 (s, 3H, CH_3_), 2.56 (s, 3H, CH_3_), 7.23 (d, ^3^*J*_H,H_ = 8.5 Hz, 1H, aromatic), 7.53 (d, ^4^*J*_H,H_ = 1.5 Hz, 1H, aromatic), 7.63 (dd, ^3^*J*_H,H_ = 8.5 Hz, ^4^*J*_H,H_ = 1.7 Hz, 1H, aromatic), 12.76 (s, 1H, NH), 12.81 (s, 1H, NH); ^13^C{^1^H}-NMR (CDCl_3_; 125.78 MHz) δ = 19.6, 19.7, 26.6, 87.7, 87.8, 103.4, 103.4, 122.3, 129.1, 131.0, 131.6, 132.4, 133.7, 162.2, 162.3, 167.5, 172.9; UV-Vis (methanol; λ [nm] (logε)): 313 (4.29), 284 (4.65), 226 (4.38), 201 (4.37).

*5,5′-(((4-Methyl-1,2-phenylene)bis(azanediyl))bis(ethan-1-yl-1-ylidene))bis(2,2-dimethyl-1,3-dioxane-4,6-dione)* (**2l**); white, 1.3 g (2.9 mmol, 52%); m.p_dec_. = 204.2 °C; ^1^H-NMR (CDCl_3_; 400.2 MHz) δ = 1.70 (s, 12H, 4CH_3_), 2.45 (s, 3H, CH_3_), 2.51 (s, 3H, CH_3_), 2.52 (s, 3H, CH_3_), 7.17 (s, 1H, aromatic), 7.23 (d, ^3^*J*_H,H_ = 8.1 Hz, 1H, aromatic), 7.30 (d, ^3^*J*_H,H_ = 8.3 Hz, 1H, aromatic), 12.68 (s, 1H, NH), 12.71 (s, 1H, NH); ^13^C{^1^H}-NMR (CDCl_3_; 125.78 MHz) δ = 19.6, 19.6, 21.2, 26.6, 87.1, 87.2, 103.2, 127.7, 128.5, 129.8, 130.1, 132.2, 140.1, 162.5, 162.5, 167.6, 173.2, 173.4; UV-Vis (methanol; λ [nm] (logε)): 319 (4.12), 283 (4.60), 227 (4.38), 203 (4.23).

*3,4-Bis((1-(2,2-dimethyl-4,6-dioxo-1,3-dioxan-5-ylidene)ethyl)amino)benzonitrile* (**2m**); white, 1.0 g (2.2 mmol, 40%); m.p_dec_. = 205.6 °C; ^1^H-NMR (CDCl_3_; 400.2 MHz) δ = 1.72 (bs, 12H, 4CH_3_), 2.55 (s, 3H, CH_3_), 2.61 (s, 3H, CH_3_), 7.49 (d, ^3^*J*_H,H_ = 8.2 Hz, 1H, aromatic), 7.68 (s, 1H, aromatic), 7.78 (d, ^3^*J*_H,H_ = 8.2 Hz, 1H, aromatic), 12.87 (s, 1H, NH), 13.04 (s, 1H, NH); ^13^C{^1^H}-NMR (CDCl_3_; 125.78 MHz) δ = 19.7, 19.9, 26.8, 26.8, 88.6, 89.0, 103.7, 103.7, 112.7, 116.7, 128.2, 131.6, 132.6, 133.1, 136.9, 162.1, 162.2, 167.7, 167.7, 172.2, 172.9; UV-Vis (methanol; λ [nm] (logε)): 324 (4.27), 289 (4.58), 224 (4.40), 204 (4.40). 

*Methyl 3,4-bis((1-(2,2-dimethyl-4,6-dioxo-1,3-dioxan-5-ylidene)ethyl)amino)benzoate* (**2n**); white, 1.2 g (2.3 mmol, 42%); m.p_dec_. = 184.8 °C; ^1^H-NMR (CDCl_3_; 400.2 MHz) δ = 1.72 (s, 6H, 2CH_3_), 1.73 (s, 6H, 2CH_3_), 2.55 (s, 3H, CH_3_), 2.60 (s, 3H, CH_3_), 3.98 (s, 3H, OCH_3_), 7.44 (d, ^3^*J*_H,H_ = 8.3 Hz, 1H, aromatic), 8.03 (s, 1H, aromatic), 8.15 (d, ^3^*J*_H,H_ = 8.2 Hz, 1H, aromatic), 12.84 (s, 1H, NH), 12.97 (s, 1H, NH); ^13^C{^1^H}-NMR (CDCl_3_; 125.78 MHz) δ = 19.7, 19.9, 26.7, 26.8, 53.0, 88.0, 88.4, 103.5, 103.6, 127.5, 129.2, 130.2, 130.8, 132.3, 136.4, 162.3, 162.4, 165.0, 167.7, 167.7, 172.6; UV-Vis (methanol; λ [nm] (logε)): 323 (4.61), 289 (4.96), 226 (4.75), 203 (4.65). 

*Ethyl 3,4-bis((1-(2,2-dimethyl-4,6-dioxo-1,3-dioxan-5-ylidene)ethyl)amino)benzoate* (**2o**); white, 0.9 g (1.7 mmol, 31%); m.p_dec_. = 171.4 °C; ^1^H-NMR (CDCl_3_; 400.2 MHz) δ = 1.42 (t, ^3^*J*_H,H_ = 7.0 Hz, 3H, CH_3_), 1.71 (s, 6H, 2CH_3_), 1.72 (s, 6H, 2CH_3_), 2.54 (s, 3H, CH_3_), 2.59 (s, 3H, CH_3_), 4.43 (q, ^3^*J*_H,H_ = 7.1 Hz, 2H, OCH_2_), 7.43 (d, ^3^*J*_H,H_ = 8.2 Hz, 1H, aromatic), 8.02 (s, 1H, aromatic), 8.15 (d, ^3^*J*_H,H_ = 8.1 Hz, 1H, aromatic), 12.82 (s, 1H, NH), 12.95 (s, 1H, NH); ^13^C{^1^H}-NMR (CDCl_3_; 125.78 MHz) δ = 14.4, 19.7, 19.9, 26.7, 26.7, 62.1, 87.9, 88.3, 103.5, 103.5, 127.5, 129.2, 130.2, 131.2, 132.2, 136.3, 162.3, 162.4, 164.4, 167.7, 167.7, 172.6, 173.3; UV-Vis (methanol; λ [nm] (logε)): 321 (4.25), 287 (4.63), 225 (4.44), 204 (4.35). 

*1H-benzo[d]imidazole-6-carboxylic acid*; m.p_dec_. = 278.0-280.0 °C; ^1^H-NMR (DMSO-*d6*/KOD; 500.2 MHz) δ = 7.74 (d, ^3^*J*_H,H_ = 8.7 Hz, 1H, aromatic), 7.96 (d, ^3^*J*_H,H_ = 8.7 Hz, ^4^*J*_H,H_ = 1.4 Hz, 1H, aromatic), 8.23 (s, 1H, aromatic), 9.08 (s, 1H, aromatic); ^1^H-NMR (D_2_O/KOD; 500.18 MHz) δ = 7.66 (d, ^3^*J*_H,H_ = 8.5 Hz, 1H, aromatic), 7.81 (dd, ^3^*J*_H,H_ = 8.5 Hz, ^4^*J*_H,H_ = 1.5 Hz, 1H, aromatic), 8.21 (s, 1H, aromatic), 8.36 (s, 1H, aromatic); ^13^C{^1^H}-NMR (DMSO-*d6*/KOD; 125.78 MHz) δ = 116.0, 117.9, 128.4, 130.4, 132.1, 135.0, 143.1, 170.8; ^13^C{^1^H}-NMR (D_2_O/KOD; 125.78 MHz) δ = 115.3 (bs), 118.3 (bs), 123.8, 124.8, 144.7, 168.4; HRMS (IT TOF): m/z Calcd for C_8_H_7_N_2_O_2_ [M + H]^+^= 163.0508, Found 163.0509.

#### 3.3.2. Step B 

Modifications to existing procedures described in the literature [22,23] rely on purifications. The crude products were extracted with hexane or toluene at Soxhlet apparatus. Into freshly distillated diphenyl ether (50 mL) at 240 °C was added compound **2** (10.0 mmol) in small portions, resulting in vigorous gas evolution. The resulting orange solution was brought to reflux for 30 min, and was then allowed to cool to 70 °C. The acetone or hexane (25 mL) was added and a dark-brown solid precipitated was filtered, washed with acetone (2 × 10 mL). The crude product was extracted with hexane or toluene at Soxhlet apparatus to yield solid as follows:

*1,10-Dihydro-1,10-phenanthroline-4,7-dione* (**3a**); [41] brown; 1.4 g (6.8 mmol, 68%); m.p_dec_. > 350 °C; ^1^H- NMR (CD_3_OD/KOD/D_2_O; 400.2 MHz) δ = 6.62 (d, ^3^*J*_H,H_ = 5.6 Hz, 2H, aromatic), 8.08 (s, 2H, aromatic), 8.36 (d, ^3^*J*_H,H_ = 5.6 Hz, 2H, aromatic); ^13^C{^1^H}-NMR (CD_3_OD/KOD/D_2_O; 100.5 MHz) δ = 111.6, 118.8, 127.0, 148.8, 150.5, 174.7. 

*5-Fluoro-1,10-dihydro-1,10-phenanthroline-4,7-dione* (**3b**); brown; 1.8 g (7.7 mmol, 77%); m.p_dec_. >350.0 °C; ^1^H-NMR (CD_3_OD/KOD/D_2_O; 400.2 MHz) δ = 6.45 (d, ^3^*J*_H,H_ = 6.5 Hz, 1H, aromatic), 6.50 (d, ^3^*J*_H,H_ = 6.2 Hz, 1H, aromatic), 7.31 (d, ^3^*J*_H,F_ = 14.1 Hz, 1H, aromatic), 8.01 (d, ^3^*J*_H,H_ = 6.4 Hz, 1H, aromatic), 8.10 (d, ^3^*J*_H,H_ = 6.1 Hz, 1H, aromatic); ^13^C{^1^H}-NMR (CD_3_OD/KOD/D_2_O; 100.5 MHz) δ = 101.1 (d, ^2^*J*_C,F_ = 24.1 Hz), 113.0 (d, ^1^*J*_C,F_ = 307.7 Hz), 117.6 (d, ^2^*J*_C,F_ = 11.3 Hz), 125.0 (d, ^3^*J*_C,F_ = 9.6 Hz), 130.7, 136.4, 142.9, 143.4, 147.9, 156.4, 158.9, 175.7, 176.9; GC-MS: t_r_ = 5.1 min, (EI) M^+^ = 230 (< 1%); ^19^F{^1^H}-NMR (CD_3_OD/KOD/D_2_O; 470.5 MHz) δ = −119.68; ^19^F-NMR (CD_3_OD/KOD/D_2_O; 470.5 MHz) δ = −119.68 (d, ^3^*J*_F,H_ = 13.8 Hz); UV-Vis (methanol; λ [nm] (logε)): 352 (3.40), 329 (3.61), 308 (3.55), 273 (3.56), 251 (3.63), 224 (3.91), 208 (3.96). 

*5-Chloro-1,10-dihydro-1,10-phenanthroline-4,7-dione* (**3c**); brown; [23]; 1.5 g (6.1 mmol, 61%); m.p_dec_. = 272.8-273.6 °C; ^1^H-NMR (CD_3_OD/KOD/D_2_O; 400.2 MHz) δ = 6.62 (d, ^3^*J*_H,H_ = 6.9 Hz, 1H, aromatic), 6.64 (d, ^3^*J*_H,H_ = 6.4 Hz, 1H, aromatic), 8.27 (d, ^3^*J*_H,H_ = 5.9 Hz, 1H, aromatic), 8.31 (d, ^3^*J*_H,H_ = 5.8 Hz, 1H, aromatic); ^13^C{^1^H}-NMR (CD_3_OD/KOD/D_2_O; 100.5 MHz) δ = 112.0, 114.5, 120.3, 122.5, 125.4, 126.1, 144.0, 147.1, 148.6, 149.4, 169.3, 173.7, 173.8, 176.0, 176.1; GC-MS: t_r_ = 11.5 min, (EI) M^+^ = 246 (100%), 248 (32%), [M + H]^+^ = 247 (8%), [M-CO]^+^ = 218 (90%); UV-Vis (methanol; λ [nm] (logε)): 356 (3.83), 338 (4.06), 324 (4.06), 285 (3.97), 273 (4.03), 249 (4.42), 231 (4.28), 215 (4.42). 

*5-Methyl-1,10-dihydro-1,10-phenanthroline-4,7-dione* (**3d**); brown; 1.9 g (8.6 mmol, 86%); m.p_dec_. > 350.0 °C; ^1^H-NMR (CD_3_OD/KOD/D_2_O; 500.2 MHz) δ = 2.73 (d, ^4^*J*_H,H_ = 0.8 Hz, 3H, CH_3_), 6.36 (d, ^3^*J*_H,H_ = 6.6 Hz, 1H, aromatic), 6.48 (d, ^3^*J*_H,H_ = 6.0 Hz, 1H, aromatic), 7.47 (d, ^4^*J*_H,H_ = 1.0 Hz, 1H, aromatic), 7.88 (d, ^3^*J*_H,H_ = 6.6 Hz, 1H, aromatic), 8.08 (d, ^3^*J*_H,H_ = 6.0 Hz, 1H, aromatic); ^13^C{^1^H}-NMR(CD_3_OD/KOD/D_2_O; 125.78 MHz) δ = 24.5, 112.0, 114.0, 119.9, 124.9, 125.9, 132.6, 139.8, 141.0, 142.5, 148.0, 174.6, 180.9; GC-MS: t_r_ = 7.0 min, (EI) [M-H]^+^ = 225 (1%); UV-Vis (methanol; λ [nm] (logε)): 355 (3.66), 327 (3.89), 306 (3.83), 275 (3.93), 247 (4.14), 224 (4.23), 209 (4.28). 

*4,7-Dioxo-1,4,7,10-tetrahydro-1,10-phenanthroline-5-carbonitrile* (**3e**); beige, 1.5 g (6.4 mmol, 64%); m.p_dec_. > 350 °C; ^1^H-NMR (DMSO–*d_6_*/KOD/D_2_O; 400.2 MHz) δ = 6.37 (d, ^3^*J*_H,H_ = 6.8 Hz, 1H, aromatic), 6.41 (d, ^3^*J*_H,H_ = 6.3 Hz, 1H, aromatic), 8.02 (d, ^3^*J*_H,H_ = 6.7 Hz, 1H, aromatic), 8.16 (d, ^3^*J*_H,H_ = 6.3 Hz, 1H, aromatic), 8.26 (s, 1H, aromatic); ^13^C{^1^H}-NMR (DMSO–*d_6_*/KOD/D_2_O; 125.78 MHz) δ = 101.1, 113.7, 114.2, 122.2, 123.3, 124.8, 131.2, 139.6, 142.2, 144.6, 149.7, 176.1, 176.8; UV-Vis (methanol; λ [nm] (logε)): 382 (3.27), 350 (3.50), 335 (3.44), 277 (3.39), 242 (3.78), 214 (3.81). 

*5-Fluoro-2,9-dimethyl-1,5,10,10a-tetrahydro-1,10-phenanthroline-4,7-dione* (**3f**); brown; 2.2 g (8.7 mmol, 87%); m.p_dec_. = 370.2–372.8 °C; ^1^H-NMR (CD_3_OD/KOD/D_2_O; 400.2 MHz) δ = 2.40 (bs, 6H, CH_3_), 6.26 (bs, 1H, aromatic), 6.35 (bs, 1H, aromatic), 7.19 (bd, ^3^*J*_H,F_ = 11.1 Hz, 1H, aromatic); ^13^C{^1^H}-NMR (CD_3_OD/KOD/D_2_O; 100.5 MHz) δ = 21.4, 22.9, 100.4 (d, ^2^*J*_C,F_ = 24.2 Hz), 112.2 (d, ^1^*J*_C,F_ = 274.4 Hz), 115.5 (d, ^2^*J*_C,F_ = 11.1 Hz), 123.1 (d, ^3^*J*_C,F_ = 9.7 Hz), 135.0, 141.5 (d, ^4^*J*_C,F_ = 3.7 Hz), 154.0, 155.8, 157.8, 158.3, 175.0, 176.2, 176.2; GC-MS: t_r_ = 10.5 min, (EI) M^+^ = 258 (100%), [M + H-CO]^+^ = 229 (98%). 

*2,9-Dimethyl-1,10-phenanthroline-4,7(1H,10H)-dione* (**3g**); brown; [22]; 1.6 g (6.8 mmol, 68%); m.p_dec_. = 291.8–292.5 °C; ^1^H-NMR (CD_3_OD/KOD/D_2_O; 400.2 MHz) δ = 2.29 (s, 6H, CH_3_), 6.24 (s, 2H, aromatic), 7.64 (s, 2H, aromatic); ^13^C{^1^H}-NMR (CD_3_OD/KOD/D_2_O; 100.5 MHz) δ = 21.8, 100.0, 110.1, 117.0, 122.9, 138.9, 155.9, 174.6. 

*5-Fluoro-2,9-dimethyl-1,5,10,10a-tetrahydro-1,10-phenanthroline-4,7-dione* (**3h**); brown; 2.2 g (8.7 mmol, 87%); m.p_dec_. = 370.2-372.8 °C; ^1^H-NMR (CD_3_OD/KOD/D_2_O; 400.2 MHz) δ = 2.40 (bs, 6H, CH_3_), 6.26 (bs, 1H, aromatic), 6.35 (bs, 1H, aromatic), 7.19 (bd, ^3^*J*_H,F_ = 11.1 Hz, 1H, aromatic); ^13^C{^1^H}-NMR (CD_3_OD/KOD/D_2_O; 100.5 MHz) δ = 21.4, 22.9, 100.4 (d, ^2^*J*_C,F_ = 24.2 Hz), 112.2 (d, ^1^*J*_C,F_ = 274.4 Hz), 115.5 (d, ^2^*J*_C,F_ = 11.1 Hz), 123.1 (d, ^3^*J*_C,F_ = 9.7 Hz), 135.0, 141.5 (d, ^4^*J*_C,F_ = 3.7 Hz), 154.0, 155.8, 157.8, 158.3, 175.0, 176.2, 176.2; GC-MS: t_r_ = 10.5 min, (EI) M^+^ = 258 (100%), [M + H-CO]^+^ = 229 (98%); ^19^F{^1^H}-NMR (CD_3_OD/KOD/D_2_O; 470.5 MHz) δ = −119.99; ^19^F-NMR (CD_3_OD/KOD/D_2_O; 470.5 MHz) δ = −119.99 (d, ^3^*J*_F,H_ = 13.9 Hz); UV-Vis (methanol; λ [nm] (logε)): 353 (3.76), 337 (3.85), 319 (4.00), 311 (3.97), 270 (4.21), 254 (4.46), 225 (4.31), 214 (4.44). 

*5-Chloro-2,9-dimethyl-1,5,10,10a-tetrahydro-1,10-phenanthroline-4,7-dione* (**3i**); 2.3 g (8.3 mmol, 83%); brown; m.p_dec_. = 332.7–334.8 °C; ^1^H-NMR (CD_3_OD/KOD/D_2_O; 400.2 MHz) δ = 2.46 (s, 3H, CH_3_), 2.49 (s, 3H, CH_3_), 6.40 (s, 1H, aromatic), 6.41 (s, 1H, aromatic), 7.78 (s, 1H, aromatic); ^13^C{^1^H}-NMR (CD_3_OD/KOD/D_2_O; 100.5 MHz) δ = 21.8, 22.6, 112.0, 114.3, 120.1, 121.1, 124.2, 126.2, 138.4, 141.6, 155.2, 156.8, 175.9, 178.1; GC-MS: t_r_ = 11.3 min, (EI) M^+^ = 273.9 (100%), [M-CO]^+^ = 245 (80%); UV-Vis (methanol; λ [nm] (logε)): 357 (3.65), 340 (3.84), 327 (3.96), 316 (3.94), 280 (4.00), 254 (4.35), 230 (4.25), 216 (4.35). 

*5-Bromo-2,9-dimethyl-1,5,10,10a-tetrahydro-1,10-phenanthroline-4,7-dione* (**3j**); brown; 2.7 g (8.5 mmol, 85%); m.p_dec_. = 325.9–326.6 °C; ^1^H-NMR (CD_3_OD/KOD/D_2_O; 400.2 MHz) δ = 2.48 (s, 3H, CH_3_), 2.50 (s, 3H, CH_3_), 6.40 (s, 1H, aromatic), 6.43 (s, 1H, aromatic), 8.09 (s, 1H, aromatic); ^13^C{^1^H}-NMR (CD_3_OD/KOD/D_2_O; 100.5 MHz) δ = 21.6, 22.7, 112.2, 112.7, 113.9, 121.4, 124.4, 124.9, 138.9, 141.1, 154.4, 157.1, 175.8, 175.8, 178.2; GC-MS: t_r_ = 13.5 min, (EI) M^+^ = 318 (100%), 320 (96%), [M-CO]^+^ = 290 (63%); UV-Vis (methanol; λ [nm] (logε)): 356 (3.87), 339 (4.08), 328 (4.17), 315 (4.13), 281 (4.19), 254 (4.55), 231 (4.46), 217 (4.53). 

*2,5,9-Trimethyl-1,10-dihydro-1,10-phenanthroline-4,7-dione* (**3k**); brown; 2.3 g (9.2 mmol, 92%); m.p_dec_. = 337.4–338.9 °C; ^1^H-NMR (CD_3_OD/KOD/D_2_O; 400.2 MHz) δ = 2.31 (s, 3H, CH_3_), 2.36 (s, 3H, CH_3_), 2.66 (s, 3H, OCH_3_), 6.16 (s, 1H, aromatic), 6.31 (s, 1H, aromatic), 7.28 (s, 1H, aromatic); ^13^C{^1^H}-NMR (CD_3_OD/KOD/D_2_O; 100.5 MHz) δ = 21.1, 23.5, 24.4, 111.4, 113.2, 119.3, 123.0, 123.9, 131.6, 138.2, 139.6, 152.8, 157.5, 174.2, 180.3; GC-MS: t_r_ = 12.1 min, (EI) M^+^ = 254 (100 %), [M + H-CO]^+^ = 225 (56%); UV-Vis (methanol; λ [nm] (logε)): 354 (3.72), 338 (3.95), 320 (4.14), 282 (4.30), 257 (4.45), 226 (4.40), 217 (4.42), 204 (4.40). 

*2,9-Dimethyl-4,7-dioxo-1,4,7,10-tetrahydro-1,10-phenanthroline-5-carbonitrile* (**3l**); brown; 1.6 g (6.2 mmol, 62%); m.p_dec_. = 390.7–392.5 °C; ^1^H-NMR (CD_3_OD/KOD/D_2_O; 400.2 MHz) δ = 2.45 (s, 3H, CH_3_), 2.46 (s, 3H, CH_3_), 6.45 (s, 1H, aromatic), 6.47 (s, 1H, aromatic), 8.21 (s, 1H, aromatic); ^13^C{^1^H}-NMR (CD_3_OD/KOD/D_2_O; 100.5 MHz) δ = 23.8, 100.0, 112.1, 112.2, 122.0, 122.9, 123.3, 130.5, 144.5, 146.7, 159.1, 159.2, 162.1, 169.4, 173.8, 174.1; GC-MS: t_r_ = 9.4 min, (EI) M^+^ = 265 (5%); UV-Vis (methanol; λ [nm] (logε)): 350 (3.90), 343 (3.93), 328 (3.87), 286 (3.82), 276 (3.90), 252 (4.31), 228 (4.15), 217 (4.28). 

*Methyl 2,9-dimethyl-4,7-dioxo-1,4,7,10-tetrahydro-1,10-phenanthroline-5-carboxylate* (**3m**); brown; 2.3 g (7.8 mmol, 78%); m.p_dec_. = 293.8–294.3 °C; ^1^H-NMR (CD_3_OD/KOD/D_2_O; 400.2 MHz) δ = 2.44 (s, 6H, 2CH_3_), 3.85 (s, 3H, OCH_3_), 6.32 (s, 1H, aromatic), 6.44 (s, 1H, aromatic), 7.78 (s, 1H, aromatic); GC-MS: t_r_ = 9.3 min, (EI) [M-MeOH]^+^ = 266 (100%); UV-Vis (methanol; λ [nm] (logε)): 345 (3.83), 330 (4.04), 314 (3.98), 280 (4.03), 252 (4.43), 215 (4.36). 

*Ethyl 2,9-dimethyl-4,7-dioxo-1,4,7,10-tetrahydro-1,10-phenanthroline-5-carboxylate* (**3n**); brown; 2.0 g (6.5 mmol, 65%); m.p_dec_. = 301.4–301.8 °C; ^1^H-NMR (CD_3_OD/KOD/D_2_O; 400.2 MHz) δ = 1.41 (t, ^3^*J*_H,H_ = 7.1 Hz, 3H, OCH_2_C*H_3_*), 2.36 (s, 3H, CH_3_), 2.38 (s, 3H, CH_3_), 4.46 (q, ^3^*J*_H,H_ = 7.0 Hz, 2H, OCH_2_), 6.29 (s, 1H, aromatic), 6.37 (s, 1H, aromatic), 7.79 (s, 1H, aromatic); GC-MS: t_r_ = 17.9 min, (EI) M^+^ = 312 (50%); UV-Vis (methanol; λ [nm] (logε)): 354 (3.58), 345 (3.81), 330 (4.04), 316 (3.99), 279 (4.03), 252 (4.44), 227 (4.26), 214 (4.36).

#### 3.3.3. Step C 

Modifications to existing procedure described in the literature [22,23] rely on the evaporation of excess phosphoryl chloride under reduced pressure, and the addition of CH_2_Cl_2_ or CHCl_3_ into reaction mixture after alkalified by NaOH solution, because of the very exothermic hydrolysis of residual phosphoryl chloride. Next the crude products were purified by chromatography and crystallization from CH_2_Cl_2_.

Freshly distillated phosphoryl chloride (82.0 g, 50 mL, 534.8 mmol) was mixed under argon with compounds **3** (5.0 mmol) and the resulting solutions were stirred at 90 °C for 4 h. The excess of phosphoryl chloride was slowly evaporated, under reduced pressure. The reaction mixture was slowly added to a well stirred mixture of ice (50 g) in water (100 mL). After stirring for 15 min. the resulting reaction mixture was carefully brought to pH 13–14 (pH 7 in the case of molecule **4g**) by adding NaOH solution (40%). The aqueous layer was extracted with CH_2_Cl_2_ (4 × 10 mL). The combined organic layers was separated and dried over MgSO_4_. Evaporation of the brown colored solvent afforded products **4** as light tan crystals. Next, the crude products were purified by chromatography on silica gel using methanol/dichloromethane as eluent and finally crystallization from CH_2_Cl_2_ to yield precipitates as follows:

*4,7-Dichloro-1,10-phenanthroline* (**4a**) [42,43]; beige; 1.0 g (4.2 mmol, 84%); m.p_dec_. = 254.8–255.0 °C; UV-Vis (methanol; λ [nm] (logε)): 300 (3.83), 264 (4.40), 239 (4.33), 225 (4.29), 202 (4.36); IR (KBr): $\tilde{\nu }$ = 3422, 3034, 1563, 1487, 1418, 835, 720 cm^−1^ (Supporting Information Figure S1). 

*4,7-Dichloro-5-fluoro-1,10-phenanthroline* (**4b**); beige; 1.1 g (4.2 mmol, 85%); m.p_dec_. = 217.8–218.0 °C; ^1^H- NMR (CDCl_3_; 600.2 MHz) δ = 7.75 (d, ^3^*J*_H,H_ = 4.7 Hz, 1H, aromatic), 7.77 (d, ^3^*J*_H,H_ = 4.8 Hz, 1H, aromatic), 7.94 (d, ^3^*J*_H,F_ = 13.3 Hz, 1H, aromatic), 9.02 (dd, ^3^*J*_H,H_ = 4.8 Hz, ^5^*J*_H,F_ = 0.5 Hz, 1H, aromatic), 9.08 (d, ^3^*J*_H,H_ = 4.8 Hz, 1H, aromatic); ^13^C{^1^H}-NMR (CDCl_3_; 150.0 MHz) δ = 106.7 (d, ^2^*J*_C,F_ = 25.9 Hz), 119.9 (d, ^2^*J*_C,F_ = 12.6 Hz), 124.4, 126.3, 126.8 (d, ^3^*J*_C,F_ = 10.8 Hz), 140.3, 142.0 (d, ^3^*J*_C,F_ = 6.2 Hz), 144.7, 148.9 (d, ^4^*J*_C,F_ = 2.3 Hz), 149.7 (d, ^4^*J*_C,F_ = 2.6 Hz), 151.0, 156.3 (d, ^1^*J*_C,F_ = 263.6 Hz); ^19^F{^1^H}-NMR (CDCl_3_; 470.5 MHz) δ = −108.98; ^19^F-NMR (CDCl_3_; 470.5 MHz) δ = −108.98 (d, ^3^*J*_F,H_ = 13.4 Hz); GC-MS: t_r_ = 9.0 min, (EI) M^+^ = 266.0 (100%); Anal. Calcd for C_12_H_5_N_2_Cl_2_F: C, 53.96; H, 1.89; N, 10.49; Found: C, 53.90; H, 1.93; N, 10.45; UV-Vis (methanol; λ [nm] (logε)): 305 (4.01), 268 (4.46), 240 (4.47), 225 (4.40), 203 (4.45); IR (KBr): $\tilde{\nu }$ = 3422, 3035, 1626, 1571,1546, 1410, 842, 752 cm^−1^ (Supporting Information Figure S2). 

*4,5,7-Trichloro-1,10-phenanthroline* (**4c**) [42]; beige; 1.0 g (3.8 mmol, 75%); m.p_dec_. = 194.6–195.0 °C; GC-MS: t_r_ = 9.4 min, (EI) M^+^ = 281.9 (100%), 283.9 (95%), [M-Cl]^+^ = 247 (46%); UV-Vis (methanol; λ [nm] (logε)): 314 (3.82), 302 (3.87), 270 (4.32), 247 (4.31), 209 (4.30); IR (KBr): $\tilde{\nu }$ = 3356, 3021, 2920, 2191, 1567, 1403, 833, 716 cm^−1^ (Supporting Information Figure S3). 

*4,7-Dichloro-5-methyl-1,10-phenanthroline* (**4d**) [23]; beige; 1.2 g (4.6 mmol, 91%); m.p_dec_. = 166.8–167.0 °C; GC-MS: t_r_ = 9.6 min, (EI) M^+^ = 262.1 (100%); UV-Vis (methanol; λ [nm] (logε)): 304 (4.09), 271 (4.50), 241 (4.49), 225 (4.42), 205 (4.47); IR (KBr): $\tilde{\nu }$ = 3454, 3089, 2933, 1685, 1550,1432, 1400, 1085, 844, 744 cm^−1^ (Supporting Information Figure S4). 

*4,7-Dichloro-1,10-phenanthroline-5-carbonitrile* (**4e**); beige; 0.6 g (2.4 mmol, 48%); m.p. = 249.7–250.0 °C; ^1^H-NMR (CDCl_3_; 400.2 MHz) δ = 7.84 (m, 2H, aromatic), 8.88 (s, 1H, aromatic), 9.13 (d, ^3^*J*_H,H_ = 4.7 Hz, 1H, aromatic), 9.19 (d, ^3^*J*_H,H_ = 4.7 Hz, 1H, aromatic); ^13^C{^1^H}-NMR (CDCl_3_; 125.78 MHz) δ = 108.1, 118.1, 124.2, 125.2, 125.3, 126.5, 135.4, 142.3, 143.6, 147.5, 148.1, 151.5, 153.4; GC-MS: t_r_ = 10.1 min, (EI) M^+^ = 273.0 (100%); Anal. Calcd for C_13_H_5_N_3_Cl_2_: C, 56.96; H, 1.84; N, 15.33; Found: C, 56.90; H, 1.94; N, 15.29; UV-Vis (methanol; λ [nm] (logε)): 315 (3.67), 302 (3.76), 292 (3.68), 268 (4.15), 249 (4.11), 231 (4.02), 210 (4.11); IR (KBr): $\tilde{\nu }$ = 3406, 3029, 2224, 1569, 1498, 1406, 1084, 846, 798 cm^−1^ (Supporting Information Figure S5). 

*7-Chloropyrrolo [2,3,4-de][1,10]phenanthrolin-5(4H)-one* (**4f**) beige; m.p_dec_. = 310–330 °C; ^1^H-NMR (D_2_O/KOD; 500.18 MHz) δ = 6.47 (d, ^3^*J*_H,H_ = 6.9 Hz, 1H, aromatic), 6.61 (d, ^3^*J*_H,H_ = 5.9 Hz, 1H, aromatic), 7.69 (s, 1H, aromatic), 8.05 (d, ^3^*J*_H,H_ = 6.9 Hz, 1H, aromatic), 8.28 (d, ^3^*J*_H,H_ = 5.9 Hz, 1H, aromatic); ^1^H- NMR (D_2_O/D_2_SO_4_; 400.2 MHz) δ = 7.29 (d, ^3^*J*_H,H_ = 6.8 Hz, 1H, aromatic), 7.42 (d, ^3^*J*_H,H_ = 6.8 Hz, 1H, aromatic), 8.28 (s, 1H, aromatic), 8.78 (d, ^3^*J*_H,H_ = 6.8 Hz, 1H, aromatic), 8.87 (d, ^3^*J*_H,H_ = 6.8 Hz, 1H, aromatic); ^13^C{^1^H}-NMR (D_2_O/KOD; 125.78 MHz) δ = 111.5, 111.6, 114.8, 120.7, 125.1, 132.5, 138.1, 139.2, 140.7, 149.3, 173.8, 178.0, 179.7; MS-IT-TOF [M-H]^-^ = 254 (80%), [M + H]^+^ = 256 (30%), HRMS (IT TOF): m/z Calcd for C_13_H_7_N_3_ClO [M + H]^+^ = 256.0278, Found 256.0279; UV-Vis (methanol; λ [nm] (logε)): 348 (3.28), 334 (3.27), 278 (3.31), 245 (3.64), 221 (3.36); IR (KBr): $\tilde{\nu }$ = 3086, 1560, 1522, 1402, 1385, 1363, 1026 cm^−1^ (Supporting Information Figure S6). 

*4,7-Dichloro-2,9-dimethyl-1,10-phenanthroline* (**4g**) [22,42,44,45]; beige; 1.3 g (4.6 mmol, 92%); m.p_dec_. = 201.0–201.5 °C; ^1^H-NMR (CDCl_3_; 400.2 MHz) δ = 2.93 (s, 6H, 2CH_3_), 7.63 (s, 2H, aromatic), 8.24 (s, 2H, aromatic); ^13^C{^1^H}-NMR (CDCl_3_; 100.5 MHz) δ = 26.0, 122.3, 124.4, 125.1, 143.0, 146.2, 160.1; CP/MAS ^13^C-NMR δ = 25.1, 121.7, 141.3, 143.8, 159.3; CP/MAS ^15^N-NMR δ = −76.15; IR (KBr): $\tilde{\nu }$ = 2963, 1637, 1568, 1522, 1402, 1385, 1363, 1026 cm^−1^ (Supporting Information Figure S7). 

*4,7-Dichloro-5-fluoro-2,9-dimethyl-1,10-phenanthroline* (**4h**) [44]; beige; 1.4 g (4.6 mmol, 93%); m.p_dec_. = 213.0–213.5 °C; ^1^H-NMR (CDCl_3_; 400.2 MHz) δ = 2.97 (s, 3H, CH_3_), 3.01 (s, 3H, CH_3_), 7.67 (s, 1H, aromatic), 7.68 (s, 1H, aromatic), 7.86 (d, ^3^*J*_H,F_ = 13.1 Hz, 1H, aromatic); ^13^C{^1^H}-NMR (CDCl_3_; 100.5 MHz) δ = 25.3, 25.5, 105.6 (d, ^2^*J*_C,F_ = 26.0 Hz), 118.0 (d, ^3^*J*_C,F_ = 12.5 Hz), 125.1 (d, ^3^*J*_C,F_ = 11.1 Hz), 126.1 (d, ^1^*J*_C,F_ = 184.1 Hz), 140.3, 142.8 (d, ^2^*J*_C,F_ = 68.6 Hz), 147.0, 154.9, 157.5, 159.1, 161.3; ^19^F{^1^H}-NMR (CDCl_3_; 470.5 MHz) δ = −110.97; ^19^F-NMR (CDCl_3_; 470.5 MHz) δ = −110.97 (d, ^3^*J*_F,H_ = 11.5 Hz); GC-MS: t_r_ = 8.2 min, (EI) M^+^ = 294 (100%), 296. (64%), [M-Cl]^+^ = 259 (12%); Anal. Calcd for C_14_H_9_N_2_Cl_2_F: C, 56.97; H, 3.07; N, 9.49; Found: C, 56.90; H, 3.19; N, 9.32; UV-Vis (methanol; λ [nm] (logε)): 309 (3.88), 296 (3.95), 273 (4.46), 241 (4.47), 211 (4.40); IR (KBr): $\tilde{\nu }$ = 3471, 3075, 1628, 1579, 1375, 1145, 858 cm^−1^ (Supporting Information Figure S8). 

*4,5,7-Trichloro-2,9-dimethyl-1,10-phenanthroline* (**4i**); beige; 1.2 g (3.9 mmol, 78%); m.p_dec_. = 162.9–163.7 °C; ^1^H-NMR (CDCl_3_; 400.2 MHz) δ = 2.92 (s, 3H, CH_3_), 2.94 (s, 3H, CH_3_), 7.63 (s, 1H, aromatic), 7.66 (s, 1H, aromatic), 8.27 (s, 1H, aromatic); ^13^C{^1^H}-NMR (CDCl_3_; 100.5 MHz) δ = 25.3, 25.7, 122.7, 124.5, 124.6, 125.0, 128.0, 129.0, 142.1, 142.3, 144.8, 147.6, 160.2, 160.3; GC-MS: t_r_ = 9.9 min, (EI) M^+^ = 310 (100%), 312 (95%), [M-Cl]^+^ = 275 (11%), [M-2Cl]^+^ = 240 (40%); Anal. Calcd for C_14_H_9_N_2_Cl_3_: C, 53.97; H, 2.91; N, 8.99; Found: C, 53.90; H, 3.01; N, 8.87; UV-Vis (methanol; λ [nm] (logε)): 316 (4.16), 304 (4.21), 274 (4.64), 247 (4.67), 210 (4.55); IR (KBr): $\tilde{\nu }$ = 3456, 3065, 2922, 1594, 1576, 1528, 1332, 905, 874 cm^−1^ (Supporting Information Figure S9). 

*5-Bromo-4,7-dichloro-2,9-dimethyl-1,10-phenanthroline* (**4j**); beige; 1.2 g (3.4 mmol, 68%); m.p_dec_. = 180.1–181.7 °C; ^1^H-NMR (CDCl_3_; 400.2 MHz) δ = 2.93 (s, 3H, CH_3_), 2.95 (s, 3H, CH_3_), 7.63 (s, 1H, aromatic), 7.69 (s, 1H, aromatic), 8.58 (s, 1H, aromatic); ^13^C{^1^H}-NMR (CDCl_3_; 100.5 MHz) δ = 25.2, 25.8, 116.2, 123.2, 125.0, 125.1, 127.8, 129.2, 142.1, 143.0, 145.0, 147.2, 160.0, 160.5; GC-MS: t_r_ = 12.5 min, (EI) M^+^ = 354 (63.7%), 356 (100%), 358 (44%), [M-Br]^+^ = 275 (28%); Anal. Calcd for C_14_H_9_N_2_Cl_2_Br: C, 47.23; H, 2.55; N, 7.87; Found: C, 47.90; H, 2.67; N, 7.86; UV-Vis (methanol; λ [nm] (logε)): 318 (3.97), 306 (4.02), 275 (4.43), 249 (4.43), 213 (4.34); IR (KBr): $\tilde{\nu }$ = 3452, 2923, 1588, 1438, 1117, 900, 876, 718 cm^−1^ (Supporting Information Figure S10). 

*4,7-Dichloro-2,5,9-trimethyl-1,10-phenanthroline* (**4k**) [42,44]; beige; 1.2 g (4.3 mmol, 86%); m.p. = 111.9–112.6 °C; ^1^H-NMR (CDCl_3_; 400.2 MHz) δ = 2.92 (s, 3H, CH_3_), 2.96 (s, 3H, CH_3_), 3.11 (s, 3H, CH_3_), 7.62 (bs, 2H, aromatic), 7.95 (s, 1H, aromatic); ^13^C{^1^H}-NMR (CDCl_3_; 100.5 MHz) δ = 25.2, 25.6, 26.3, 123.9, 124.5, 124.6, 125.4, 126.9, 133.8, 142.3, 143.1, 144.9, 147.2, 159.0, 159.1; CP/MAS ^13^C-NMR δ = 25.0, 27.5, 122.5, 123.5, 131.0, 139.0, 143.3, 145.3, 158.3; CP MAS ^15^N-NMR δ = −75.52; GC-MS: t_r_ = 9.7 min, (EI) M^+^ = 290 (100 %), [M-Cl]^+^ = 255 (12%); Anal. Calcd for C_15_H_12_N_2_Cl_2_: C, 61.88; H, 4.15; N, 9.62; Found: C, 61.89; H, 4.20; N, 9.60; UV-Vis (methanol; λ [nm] (logε)): 343 (3.57), 311 (3.73), 275 (4.30), 254 (4.34), 213 (4.24); IR (KBr): $\tilde{\nu }$ = 3452, 2924, 1609, 1579, 1530, 1439, 1380, 1236, 902, 871, 716 cm^−1^ (Supporting Information Figure S11). 

*4,7-Dichloro-2,9-dimethyl-1,10-phenanthroline-5-carbonitrile* (**4l**); beige; 0.5 g (1.6 mmol, 32%); m.p_dec_. = 188.6–189.9 °C; ^1^H-NMR (CDCl_3_; 400.2 MHz) δ = 2.96 (s, 3H, CH_3_), 2.99 (s, 3H, CH_3_), 7.72 (s, 1H, aromatic), 7.73 (s, 1H, aromatic), 8.79 (s, 1H, aromatic); ^13^C{^1^H}-NMR (CDCl_3_; 125.78 MHz) δ = 25.6, 26.2, 106.9, 118.4, 122.6, 123.7, 125.6, 127.0, 134.3, 142.2, 143.7, 146.1, 146.8, 161.6, 163.8; GC-MS: t_r_ = 10.5 min, (EI) M^+^ = 301 (100%), [M-Cl]^+^ = 266 (14%); Anal. Calcd for C_15_H_9_N_3_Cl_2_: C, 59.63; H, 3.00; N, 13.91; Found: C, 59.89; H, 3.27; N, 13.87; UV-Vis (methanol; λ [nm] (logε)): 353 (3.07), 337 (3.68), 319 (4.24), 307 (4.31), 275 (4.67), 249 (4.60), 235 (4.54), 211 (4.59); IR (KBr): $\tilde{\nu }$ = 3471, 3406, 2223, 1587, 1443, 1357, 1338, 908, 847 cm^−1^ (Supporting Information Figure S12). 

*Ethyl 4,7-dichloro-2,9-dimethyl-1,10-phenanthroline-5-carboxylate* (**4m**); beige; 0.7 g (1.9 mmol, 38%); m.p. = 115.9–116.2 °C; ^1^H-NMR (CDCl_3_; 400.2 MHz) δ = 1.43 (t, ^3^*J*_H,H_ = 7.1 Hz, 3H, OCH_2_C*H_3_*), 2.93 (s, 3H, CH_3_), 2.94 (s, 3H, CH_3_), 4.49 (q, ^3^*J*_H,H_ = 7.1 Hz, 2H, OCH_2_), 7.65 (2s, 2H, aromatic), 8.31 (s, 1H, aromatic); ^13^C{^1^H}-NMR (CDCl_3_; 100.5 MHz) δ = 14.1, 25.5, 25.9, 62.6, 122.1, 123.5, 123.8, 124.9, 126.0, 129.3, 141.7, 143.6, 146.3, 146.6, 160.5, 161.5, 169.0; GC-MS: t_r_ = 10.9 min, (EI) M^+^ = 348 (45%), [M-Cl]^+^ = 313 (21%); Anal. Calcd for C_17_H_14_N_2_Cl_2_O_2_: C, 58.47; H, 4.04; N, 8.02; Found: C, 58.90; H, 4.25; N, 7.98; UV-Vis (methanol; λ [nm] (logε)): 308 (3.88), 295 (3.95), 272 (4.43), 245 (4.44), 212 (4.33); IR (KBr): $\tilde{\nu }$ = 3432, 2980, 1727, 1577, 1275, 1248, 1214,1190, 1122, 1031 cm^−1^ (Supporting Information Figure S13).

#### 3.3.4. Hydrolyses of 4**e**

Compound **4**e (1.0 g, 3.7 mmol) was dissolved in concentrated hydrochloric acid (35%) (23 mL) at r.t. After stirring for 72 h at 100 °C, the volatiles were evaporated. The crude product was purified by extraction at Soxhlet apparatus (H_2_O) and finally was dried over P_4_O_10_ to yield a precipitate of 7*-*chloropyrrolo [2,3,4-de][1,10]phenanthrolin-5(4H)-one (**4f**, 0.3 g, 1.4 mmol, 37%).

### 3.4. Syntheses of 4,7-di(pyrrolidin-1-yl)-1,10-phenanthrolines ***5a***, ***5b***, ***5c***, ***5d*** and ***5e***

*These were* based on the procedure described in [24]. Our *modifications to this procedure involve the purification*. The appropriate compounds **4a**, **4c**, **4g**, **4h** or **4k** (1.0 mmol) were mixed with pyrrolidine (1.5 g, 21.0 mmol) in a microvave vial equipped with a magnetic stir bar. The vial was sealed, placed in a microwave reactor and heated at 130 °C for 45 min (for compound **5a**) or 2 h (for compounds **5b**, **5c**, **5d**, and **5e**). The mixture was cooled to r.t., transferred to a round bottom flask (100 mL) and evaporated under reduced pressure. The residue has been washed with saturated NaHCO_3_ (2 × 10 mL) with stirring, and then with water (2 × 10 mL). The organic residue has been dissolved with CH_2_Cl_2_ (20 mL), dried over MgSO_4_ and evaporated under reduced pressure affording the corresponding products. Next, the crude products were purified by chromatography on silica gel using methanol/dichloromethane as eluent and finally crystallization from CH_2_Cl_2_ to yield precipitates as follows:

*4,7-Di(pyrrolidin-1-yl)-1,10-phenanthroline* (**5a**) [24,46]; 0.1 g (0.4 mmol, 38%); m.p_dec_. = 246.8–247.7 °C; ^1^H-NMR (CDCl_3_; 400.2 MHz) δ = 2.03 (m, 8H, 4CH_2_), 3.67 (m, 8H, 4CH_2_), 6.69 (d, ^3^*J*_H,H_ = 5.5 Hz, 2H, aromatic), 7.93 (s, 2H, aromatic), 8.72 (d, ^3^*J*_H,H_ = 5.4 Hz, 2H, aromatic); ^13^C{^1^H}-NMR (CDCl_3_; 100.5 MHz) δ = 26.1, 52.4, 105.6, 119.5, 119.6, 148.5, 149.4, 152.9; MS-IT-TOF [M+H]^+^ = 319 (100%), HRMS (IT TOF): m/z Calcd for C_20_H_23_N_4_ [M+H]^+^ = 319.1917, Found 319.1916; UV-Vis (methanol; λ [nm] (logε)): 362 (4.26), 328 (4.18), 274 (4.48), 255 (4.43), 217 (4.54); IR (ATR): $\tilde{\nu }$ = 2952, 2935, 2860, 1662, 1615, 1552, 1434,1349, 791, 729 cm^−1^; (Supporting Information Figure S14). 

*5-Chloro-4,7-di(pyrrolidin-1-yl)-1,10-phenanthroline* (**5b**); 0.1 g (0.4 mmol, 42%); m.p. = 151.0–151.6 °C; ^1^H-NMR (CDCl_3_; 400.2 MHz) δ = 1.99 (m, 4H, 2CH_2_), 2.07 (m, 4H, 2CH_2_), 3.53 (m, 4H, 2CH_2_), 3.70 (m, 4H, 2CH_2_), 6.67 (d, ^3^*J*_H,H_ = 5.8 Hz, 1H, aromatic), 6.90 (d, ^3^*J*_H,H_ = 5.6 Hz, 1H, aromatic), 7.92 (s, 1H, aromatic), 8.73 (d, ^3^*J*_H,H_ = 5.7 Hz, 1H, aromatic), 8.74 (d, ^3^*J*_H,H_ = 5.7 Hz, 1H, aromatic); ^13^C{^1^H}-NMR (CDCl_3_; 100.5 MHz) δ = 25.1, 26.1, 52.1, 52.4, 105.9, 107.9, 117.7, 118.5, 122.4, 124.1, 145.2, 147.7, 148.2, 148.9, 152.7, 153.9; MS-IT-TOF [M+H]^+^ = 353 (100%), HRMS (IT TOF): *m*/*z* Calcd for C_20_H_22_ClN_4_ [M + H]^+^ = 353.1527, Found 353.1526; UV-Vis (methanol; λ [nm] (logε)): 372 (3.98), 333 (3.88), 281 (4.22), 262 (4.19), 220 (4.65); IR (KBr): $\tilde{\nu }$ = 2956, 2870,1572, 1514, 1445, 1350, 803 cm^−1^; (Supporting Information Figure S15). 

*2,9-Dimethyl-4,7-di(pyrrolidin-1-yl)-1,10-phenanthroline* (**5c**); 0.2 g (0.5 mmol, 48%); m.p. = 165.8–166.0 °C; ^1^H-NMR (CDCl_3_; 500.18 MHz) δ = 2.04 (m, 8H, 4CH_2_), 2.77 (s, 6H, 2CH_3_), 3.68 (m, 8H, 4CH_2_), 6.61 (s, 2H, aromatic), 7.90 (s, 2H, aromatic); ^13^C{^1^H}-NMR (CDCl_3_; 100.5 MHz) δ = 25.8, 26.0, 52.4, 105.7, 118.2, 119.0, 146.3, 158.4, 157.5; CP/MAS ^13^C-NMR δ = 26.0, 51.8, 104.7, 117.5, 147.3, 149.6, 154.6; CP/MAS ^15^N-NMR δ = −291.94, −62.66; MS-IT-TOF [M+H]^+^ = 347 (100%), HRMS (IT TOF): m/z Calcd for C_22_H_27_N_4_ [M+H]^+^ = 347.2230, Found 347.2229; UV-Vis (methanol; λ [nm] (logε)): 387 (3.80), 358 (4.10), 322 (4.04), 273 (4.34), 245 (4.26), 214 (4.35); IR (ATR): $\tilde{\nu }$ = 2954, 2866, 1539, 1423, 1349, 1078, 802 cm^−1^; (Supporting Information Figure S16). 

*5-Fluoro-2,9-dimethyl-4,7-di(pyrrolidin-1-yl)-1,10-phenanthroline* (**5d**); 0.2 g (0.5 mmol, 51%); m.p. = 100.9–101.0 °C; ^1^H-NMR (CDCl_3_; 500.18 MHz) δ = 1.96 (m, 4H, 2CH_2_), 2.01 (m, 4H, 2CH_2_), 2.74 (s, 3H, CH_3_), 2.76 (s, 3H, CH_3_), 3.47 (m, 4H, 2CH_2_), 3.61 (m, 4H, 2CH_2_), 6.58 (s, 1H, aromatic), 6.65 (s, 1H, aromatic), 7.45 (d, ^3^*J*_H,F_ = 15.5 Hz, 1H, aromatic); ^13^C{^1^H}-NMR (CDCl_3_; 100.5 MHz) δ = 25.6, 25.6, 25.8, 25.9, 25.9, 51.7, 51.8, 52.0, 102.9 (d, ^1^*J*_C,F_ = 27.1 Hz), 106.1, 109.9 (d, ^2^*J*_C,F_ = 16.2 Hz), 117.6 (d, ^2^*J*_C,F_ = 10.6 Hz), 144.5, 148.7 (d, ^3^*J*_C,F_ = 4.1 Hz), 152.1 (d, *J* = 1.0 Hz), 152.3, 152.9 (d, ^3^*J*_C,F_ = 4.3 Hz), 154.8, 157.1 (d, ^4^*J*_C,F_ = 1.6 Hz), 158.7; ^19^F-NMR (CDCl_3_; 470.5 MHz) δ = −112.29 (d, ^3^*J*_F,H_ = 15.5 Hz); ^19^F{^1^H}-NMR (CDCl_3_; 470.5 MHz) δ = −112.29; MS-IT-TOF [M + H]^+^ = 365 (100%), HRMS (IT TOF): *m*/*z* Calcd for C_22_H_26_FN_4_ [M + H]^+^ = 365.2136, Found 365.2135; UV-Vis (methanol; λ [nm] (logε)): 407 (3.91), 359 (4.30), 325 (4.26), 281 (4.56), 256 (4.46), 221 (4.73); IR (KBr): $\tilde{\nu }$ = 2957, 2866, 1564, 1431, 1352, 1090, 820 cm^−1^; CCDC 1479401; (Supporting Information Figure S17). 

*2,5,9-Trimethyl-4,7-di(pyrrolidin-1-yl)-1,10-phenanthroline* (**5e**); 0.1 g (0.4 mmol, 39%); m.p. = 101.7–102.0 °C; ^1^H-NMR (CDCl_3_; 400.2 MHz) δ = 1.97 (m, 4H, 2CH_2_), 2.03 (m, 4H, 2CH_2_), 2.68 (s, 3H, CH_3_), 2.76 (s, 3H, CH_3_), 2.77 (s, 3H, CH_3_), 3.35 (m, 4H, 2CH_2_), 3.68 (m, 4H, 2CH_2_), 6.61 (s, 1H, aromatic), 6.85 (s, 1H, aromatic), 7.63 (s, 1H, aromatic); ^13^C{^1^H}-NMR (CDCl_3_; 100.5 MHz) δ = 21.4, 24.4, 25.6, 25.7, 26.0, 52.0, 52.3, 105.8, 109.3, 117.9, 120.8, 122.4, 127.4, 145.1, 146.7, 153.2, 155.4, 156.7, 157.5; MS-IT-TOF [M + H]^+^ = 361 (100%), HRMS (IT TOF): *m*/*z* Calcd for C_23_H_29_N_4_ [M + H]^+^ = 361.2386, Found 361.2385; UV-Vis (methanol; λ [nm] (logε)): 361 (4.17), 325 (4.15), 285 (4.44), 260 (4.36), 216 (4.54); IR (ATR): $\tilde{\nu }$ = 2962, 2846, 1541, 1424, 1349, 1077, 801 cm^−1^; (Supporting Information Figure S18).

### 3.5. Syntheses of 4,7-di(9H-carbazol-9-yl)-1,10-phenanthrolines ***5f***, ***5g***, ***5h*** and ***5m***

These were based on the *procedure described in the literature* [25]. To s suspension of NaH (0.1 g, 3.94 mmol) in THF (50 mL) 9H-carbazole (0.5 g, 3.01 mmol) was added and stirred until evolution of H_2_ ceased. Reagents were stirred under reflux for 30 min. under argon. Compounds **4a**, **4b**, **4d** or **4e** (1.50 mmol) was then added to the reaction mixture, which was refluxed overnight. After evaporation of the solvent to give a solid, water (20 mL) and chloroform (100 mL) was added. The organic layer was separated and the aqueous layer was extracted four times with chloroform. The combined organic layers were dried over MgSO_4_. After solvent evaporating, the crude product was purified by column chromatography on silica gel using methanol/dichloromethane as eluent to afford a white powder, and finally crystallization from a mixture of CH_2_Cl_2_ and hexane to yield solids as follows:

*4,7-Di(9H-carbazol-9-yl)-1,10-phenanthroline* [25] (**5f**) white 0.7 g (1.29 mmol, 86%); m.p_dec_. = 275.1–280.0 °C; ^1^H-NMR (CDCl_3_; 500.2 MHz) δ = 7.06 (d, ^3^*J*_H,H_ = 7.9 Hz, 4H, aromatic), 7.28–7.36 (m, 10H, aromatic), 7.85 (d, ^3^*J*_H,H_ = 4.7 Hz, 2H, aromatic), 8.15 (d, ^3^*J*_H,H_ = 7.2 Hz, 4H, aromatic), 9.49 (d, ^3^*J*_H,H_ = 4.7 Hz, 2H, aromatic); ^13^C{^1^H}-NMR (CDCl_3_; 125.78 MHz) δ = 110.1, 120.7, 121.0, 123.0, 124.1, 126.5, 126.7, 141.3, 143.5, 148.8, 151.6; CP/MAS ^13^C-NMR δ = 109.4, 120.2, 122.0, 123.6, 124.8, 127.4, 139.4, 141.2, 147.5, 149.2, 150.3; CP/MAS ^15^N-NMR δ = −250.68, −73.05; UV-Vis (methanol; λ [nm] (logε)): 353 (3.10), 332 (3.54), 319 (3.54), 277 (4.03), 268 (4.06), 247 (4.16), 230 (4.50), 210 (4.18); IR (ATR): $\tilde{\nu }$ = 3062, 2371, 1448, 1222, 750, 725 cm^−1^; (Supporting Information Figure S19). 

*4,7-Di(9H-carbazol-9-yl)-5-fluoro-1,10-phenanthroline* (**5g**) 0.4 g (0.81 mmol, 54%); m.p_dec_. = 184.3–185.0 °C; ^1^H-NMR (CDCl_3_; 400.2 MHz) δ = 7.03 (d, ^3^*J*_H,F_ = 12.6 Hz, 1H, aromatic), 7.09 (dd, ^3^*J*_H,H_ = 7.7 Hz, ^3^*J*_H,H_ = 5.2 Hz, 4H, aromatic), 7.30–7.43 (m, 8H, aromatic), 7.91 (dd, ^3^*J*_H,H_ = 9.9 Hz, ^3^*J*_H,H_ = 4.7 Hz, 2H, aromatic), 8.18 (t, ^3^*J*_H,H_ = 8.4 Hz, 4H, aromatic), 9.47 (d, ^3^*J*_H,H_ = 4.4 Hz, 1H, aromatic), 9.54 (d, ^3^*J*_H,H_ = 4.6 Hz, 1H, aromatic); ^13^C{^1^H}-NMR (CDCl_3_; 100.5 MHz) δ = 106.5 (d, ^1^*J*_C,F_ = 24.7 Hz), 109.4, 110.0, 119.6, 120.7, 120.8 (d, ^4^*J*_C,F_ = 1.0 Hz), 121.0, 121.2, 121.4, 123.6, 124.1 (d, ^2^*J*_C,F_ = 17.7 Hz), 125.1, 125.5, 126.56 (d, ^3^*J*_C,F_ = 4.8 Hz), 126.58 (d, ^2^*J*_C,F_ = 23.6 Hz), 141.1, 141.6, 141.7 (d, ^4^*J*_C,F_ = 1.9 Hz), 143.2 (d, ^3^*J*_C,F_ = 6.0 Hz), 146.1, 150.0 (d, ^4^*J*_C,F_ = 2.5 Hz), 150.9, 152.6, 154.2, 156.8; ^19^F-NMR (CDCl_3_; 470.5 MHz) δ = −112.43 (d, ^3^*J*_F,H_ = 10.6 Hz); ^19^F{^1^H}-NMR (CDCl_3_; 470.5 MHz) δ = −112.43; MS-IT-TOF [M+H]^+^ = 529 (100%), HRMS (IT TOF): *m*/*z* Calcd for C_36_H_22_FN_4_ [M + H]^+^ = 529.1828, Found 529.1823. UV-Vis (methanol; λ [nm] (logε)): 367 (3.14), 329 (3.37), 318 (3.37), 278 (3.88), 256 (3.88), 230 (4.33), 209 (4.08); IR (ATR): $\tilde{\nu }$ = 3045, 3023, 2361, 1445, 1224, 744, 720 cm^−1^; (Supporting Information Figure S20). 

*4,7-Di(9H-carbazol-9-yl)-5-methyl-1,10-phenanthroline* (**5h**) white 0.7 g (1.34 mmol, 89%); m.p_dec_. > 350 °C; ^1^H-NMR (CDCl_3_; 500.2 MHz) δ = 1.60 (d, ^4^*J*_H,H_ = 0.8 Hz, 3H, CH_3_), 6.93 (d, ^3^*J*_H,H_ = 8.1 Hz, 2H, aromatic), 7.10 (d, ^3^*J*_H,H_ = 8.1 Hz, 2H, aromatic), 7.22 (d, ^4^*J*_H,H_ = 1.0 Hz, 1H, aromatic), 7.29-7.40 (m, 8H, aromatic), 7.68 (d, ^3^*J*_H,H_ = 4.6 Hz, 1H, aromatic), 7.80 (d, ^3^*J*_H,H_ = 4.7 Hz, 1H, aromatic), 8.16 (d, ^3^*J*_H,H_ = 7.7 Hz, 2H, aromatic), 8.18 (d, ^3^*J*_H,H_ = 8.1 Hz, 2H, aromatic), 9.43 (dd, ^3^*J*_H,H_ = 6.9 Hz, ^3^*J*_H,H_ = 4.7 Hz, 2H, aromatic); ^13^C{^1^H}-NMR (CDCl_3_; 100.5 MHz) δ = 21.1, 109.7, 110.1, 120.70, 120.73, 120.8, 121.0, 123.1, 123.7, 124.0, 124.5, 125.6, 126.2, 126.5, 126.8, 128.2, 133.6, 141.4, 142.2, 142.4, 143.4, 148.0, 149.7, 150.9, 151.2; CP/MAS ^13^C-NMR δ = 23.4, 111.3, 116.6, 120.7, 123.3, 126.7, 128.2, 133.7, 136.6, 142.0, 147.4, 148.5, 150.3; CP/MAS ^15^N-NMR δ = −254.60, −249.48, −78.17, −54.38; MS-IT-TOF [M + H]^+^ = 525 (30%), HRMS (IT TOF): *m*/*z* Calcd for C_37_H_25_N_4_ [M + H]^+^ = 525.2001, Found 525.2091; UV-Vis (methanol; λ [nm] (logε)): 355 (3.32), 333 (3.83), 319 (3.76), 277 (4.28), 256 (4.25), 245 (4.41), 232 (4.72), 210 (4.38); IR (ATR): $\tilde{\nu }$ = 3046, 2363, 1449, 1230, 747, 723 cm^−1^; (Supporting Information Figure S21). 

*4,7-Di(9H-carbazol-9-yl)-1,10-phenanthroline-5-carbonitrile***(5m)** brown 0.7 g (1.29 mmol, 86%); m.p_dec_. = 145–147 °C; ^1^H-NMR (CDCl_3_; 400.2 MHz) δ = 6.93 (d, ^3^*J*_H,H_ = 7.4 Hz, 2H, aromatic), 7.07 (d, ^3^*J*_H,H_ = 7.3 Hz, 2H, aromatic), 7.31-7.43 (m, 8H, aromatic), 7.88 (d, ^3^*J*_H,H_ = 4.6 Hz, 1H, aromatic), 7.93 (d, ^3^*J*_H,H_ = 4.7 Hz, 1H, aromatic), 8.05 (s, 1H, aromatic), 8.18 (d, ^3^*J*_H,H_ = 7.3 Hz, 4H, aromatic), 9.55 (d, ^3^*J*_H,H_ = 4.6 Hz, 1H, aromatic), 9.60 (d, ^3^*J*_H,H_ = 4.7 Hz, 1H, aromatic); ^13^C{^1^H}-NMR (CDCl_3_; 100.5 MHz) δ = 107.1, 109.2, 109.6, 115.7, 121.0, 121.1, 121.3, 121.7, 123.8, 124.4, 124.5, 125.2, 125.6, 125.8, 126.6, 126.9, 134.4, 141.2, 142.3, 143.1, 144.5, 148.8, 149.6, 153.1, 154.7; MS-IT-TOF [M+H]^+^ = 536 (100%), HRMS (IT TOF): *m*/*z* Calcd for C_37_H_22_N_5_ [M + H]^+^ = 536.1875, Found 536.1876; UV-Vis (methanol; λ [nm] (logε)): 368 (3.28), 323 (3.77), 292 (4.23), 286 (4.22), 257 (4.33), 232 (4.73); IR (ATR): $\tilde{\nu }$ = 3050, 2925, 2224, 1450, 1226, 752, 726 cm^−1^. (Supporting Information Figure S26).

### 3.6. Syntheses of 4,7-di(10H-phenothiazin-10-yl)-1,10-phenanthrolines ***5i***, ***5j***, ***5k*** and ***5n***

To the suspension of NaH (1.1 g, 48.1 mmol) in THF (50 mL) 10*H*-phenothiazine (8.0 g, 40.0 mmol) was added and stirred until evolution of H_2_ ceased. Reagents were stirred under reflux for 30 min. under argon. Then compounds **4a**, **4b**, **4d** or **4e** (20.0 mmol) were added to the reaction mixture in one portion. After heating under reflux for 24 h, the solvent was evaporated and a mixture composed of CH_2_Cl_2_ (20 mL) and water (20 mL) was added. The organic layer was separated and the aqueous layer was extracted four times with chloroform (4 × 20 mL). The combined organic layers were dried over MgSO_4_, and the crude product was purified by crystallization from mixture of THF (CH_2_Cl_2_) and hexane to yield solid as follows:

*4,7-Di(10H-phenothiazin-10-yl)-1,10-phenanthroline* (**5i**) greenish 10.8 g (18.8 mmol, 94%); m.p_dec_. = 149.4–150.0 °C; ^1^H-NMR (CDCl_3_; 500.2 MHz) δ = 6.10 (dd, ^3^*J*_H,H_ = 8.2 Hz, ^4^*J*_H,H_ = 1.1 Hz, 4H, aromatic), 6.76 (dt, ^3^*J*_H,H_ = 7.8 Hz, ^4^*J*_H,H_ = 1.6 Hz, 4H, aromatic), 6.82 (td, ^3^*J*_H,H_ = 7.5 Hz, ^4^*J*_H,H_ = 1.2 Hz, 4H, aromatic), 7.07 (dd, ^3^*J*_H,H_ = 7.6 Hz, ^4^*J*_H,H_ = 1.5 Hz, 4H, aromatic), 7.88 (d, ^3^*J*_H,H_ = 4.7 Hz, 2H, aromatic), 8.15 (s, 2H, aromatic), 9.50 (d, ^3^*J*_H,H_ = 4.7 Hz, 2H, aromatic); ^13^C{^1^H}-NMR (CDCl_3_; 125.78 MHz) δ = 116.0, 121.1, 123.4, 123.5, 127.1, 127.3, 128.5, 142.9, 146.5, 149.5, 152.0; MS-IT-TOF [M + H]^+^ = 575 (100%), HRMS (IT TOF): *m*/*z* Calcd for C_36_H_23_N_4_S_2_ [M + H]^+^ = 575.1364, Found 575.1358. UV-Vis (methanol; λ [nm] (logε)): 302 (3.56), 270 (4.09), 251 (4.33), 237 (4.21), 228 (4.15), 208 (4.14); IR (ATR): $\tilde{\nu }$ = 3055, 2984, 1460, 1305, 1234, 748, 734, 632 cm^−1^; (Supporting Information Figure S22). 

*5-Fluoro-4,7-di(10H-phenothiazin-10-yl)-1,10-phenanthroline* (**5j**) brown 11.4 g (19.2 mmol, 96%); m.p_dec_. = 186.1–187.0 °C; ^1^H-NMR (CDCl_3_; 500.2 MHz) δ = 5.84 (d, ^3^*J*_H,H_ = 8.2 Hz, ^4^*J*_H,H_ = 0.8 Hz, 2H, aromatic), 6.17 (d, ^3^*J*_H,H_ = 8.1 Hz, ^4^*J*_H,H_ = 1.2 Hz, 2H, aromatic), 6.69 (td, ^3^*J*_H,H_ = 7.4 Hz, ^4^*J*_H,H_ = 1.6 Hz, 2H, aromatic), 6.75 (td, ^3^*J*_H,H_ = 7.5 Hz, ^4^*J*_H,H_ = 1.3 Hz, 2H, aromatic), 6.80 (td, ^3^*J*_H,H_ = 7.4 Hz, ^4^*J*_H,H_ = 1.7 Hz, 2H, aromatic), 6.85 (td, ^3^*J*_H,H_ = 7.4 Hz, ^4^*J*_H,H_ = 1.3 Hz, 2H, aromatic), 6.98 (dd, ^3^*J*_H,H_ = 7.6 Hz, ^4^*J*_H,H_ = 1.5 Hz, 2H, aromatic), 7.08 (dd, ^3^*J*_H,H_ = 7.5 Hz, ^4^*J*_H,H_ = 1.6 Hz, 2H, aromatic), 7.82 (d, ^3^*J*_H,H_ = 4.6 Hz, 1H, aromatic), 7.87 (d, ^3^*J*_H,F_ = 12.5 Hz, 1H, aromatic), 7.92 (d, ^3^*J*_H,H_ = 4.6 Hz, 1H, aromatic), 9.47 (d, ^3^*J*_H,H_ = 4.6 Hz, 1H, aromatic), 9.52 (d, ^3^*J*_H,H_ = 4.6 Hz, 1H, aromatic); ^13^C{^1^H}-NMR (CDCl_3_; 125.78 MHz) δ = 106.5 (d, ^3^*J*_C,F_ = 24.7 Hz), 115.4 (d, ^1^*J*_C,F_ = 108.4 Hz), 119.8, 121.4, 122.4 (d, ^4^*J*_C,F_ = 13.5 Hz), 123.3 (d, ^2^*J*_C,F_ = 91.9 Hz), 127.0 (d, ^4^*J*_C,F_ = 14.5 Hz), 127.3 (d, ^3^*J*_C,F_ = 16.0 Hz), 128.2, 128.4 (d, ^2^*J*_C,F_ = 66.8 Hz), 142.8 (d, ^5^*J*_C,F_ = 11.9 Hz), 144.3, 145.7 (d, ^5^*J*_C,F_ = 5.3 Hz), 147.3, 150.6, 151.1, 153.9, 154.7, 156.8; ^19^F-NMR (CDCl_3_; 470.5 MHz) δ = −113.33 (d, ^3^*J*_F,H_ = 11.9 Hz); ^19^F{^1^H}-NMR (CDCl_3_; 470.5 MHz) δ = −113.33; MS-IT-TOF [M + Na]^+^ = 615 (30%), HRMS (IT TOF): *m*/*z* Calcd for C_20_H_23_N_4_ [M + Na]^+^ = 615.1089, Found 615.1083. UV-Vis (methanol; λ [nm] (logε)): 307 (3.56), 271 (4.04), 250 (4.33), 239 (4.23), 227 (4.14), 210 (4.19); IR (ATR): $\tilde{\nu }$ = 3057, 2962, 2360, 1459, 1312, 1235, 739, 643 cm^−1^; (Supporting Information Figure S23).

*5-Methyl-4,7-di(10H-phenothiazin-10-yl)-1,10-phenanthroline* (**5k**) purple 9.9 g (16.8 mmol, 84%); m.p_dec_. >350 °C; ^1^H-NMR (CDCl_3_; 500.2 MHz) δ = 2.91 (s, 3H, CH_3_), 5.80 (d, ^3^*J*_H,H_ = 8.0 Hz, 2H, aromatic), 6.11 (d, ^3^*J*_H,H_ = 7.9 Hz, 2H, aromatic), 6.65-6.85 (m, 8H, aromatic), 6.96 (d, ^3^*J*_H,H_ = 7.3 Hz, 2H, aromatic), 7.07 (d, ^3^*J*_H,H_ = 7.4 Hz, 2H, aromatic), 7.70 (d, ^3^*J*_H,H_ = 4.5 Hz, 1H, aromatic), 7.83 (d, ^3^*J*_H,H_ = 4.5 Hz, 1H, aromatic), 7.92 (s, 1H, aromatic), 9.45 (dd, ^3^*J*_H,H_ = 8.4 Hz, ^3^*J*_H,H_ = 4.6 Hz, 2H, aromatic); ^13^C{^1^H} -NMR(CDCl_3_; 100.5 MHz) δ = 23.6, 115.6, 116.0, 119.0, 120.9, 123.0, 123.3, 124.2, 126.8, 127.06, 127.1, 127.2, 127.8, 129.0, 129.4, 134.5, 142.6, 142.8, 145.4, 146.4, 148.6, 151.0, 151.3, 151.7; CP/MAS ^13^C-NMR δ = 25.9, 116.8, 123.4, 124.6, 127.2, 135.3, 141.5, 144.8, 147.1, 149.0, 152.6, 153.5, 155.6; CP/MAS ^15^N-NMR δ = −273.57, −62.21, −51.07; MS-IT-TOF [M + H]^+^ = 589 (100%), HRMS (IT TOF): *m*/*z* Calcd for C_37_H_25_N_4_S_2_ [M + H]^+^ = 589.15207, Found 589.15059; UV-Vis (methanol; λ [nm] (logε)): 332 (3.28), 307 (3.79), 273 (4.29), 255 (4.58), 242 (4.48); IR (ATR): $\tilde{\nu }$ = 3059, 2960, 1461, 1307, 1241, 736, 631 cm^−1^; (Supporting Information Figure S24). 

*4,7-Di(10H-phenothiazin-10-yl)-1,10-phenanthroline-5-carbonitrile* (**5n**) brown 9.9 g (16.6 mmol, 83%); m.p_dec_. = 270–275 °C; ^1^H-NMR (CDCl_3_; 500.2 MHz) δ = 5.67 (d, ^3^*J*_H,H_ = 8.2 Hz, 2H, aromatic), 6.10 (d, ^3^*J*_H,H_ = 8.1 Hz, 2H, aromatic), 6.67 (t, ^3^*J*_H,H_ = 7.8 Hz, 2H, aromatic), 6.77 (t, ^3^*J*_H,H_ = 7.4 Hz, 2H, aromatic), 6.82 (t, ^3^*J*_H,H_ = 7.8 Hz, 2H, aromatic), 6.90 (t, ^3^*J*_H,H_ = 7.2 Hz, 2H, aromatic), 6.98 (d, ^3^*J*_H,H_ = 7.6 Hz, 2H, aromatic), 7.13 (d, ^3^*J*_H,H_ = 7.6 Hz, 2H, aromatic), 7.89 (d, ^3^*J*_H,H_ = 4.6 Hz, 1H, aromatic), 8.01 (d, ^3^*J*_H,H_ = 4.6 Hz, 1H, aromatic), 8.76 (s, 1H, aromatic), 9.58 (d, ^3^*J*_H,H_ = 4.6 Hz, 1H, aromatic), 9.65 (d, ^3^*J*_H,H_ = 4.6 Hz, 1H, aromatic); ^13^C{^1^H}-NMR(CDCl_3_; 125.78 MHz) δ = 107.5, 115.1, 115.9, 117.6, 119.6, 121.7, 123.4, 124.1, 126.9, 127.1, 127.3, 127.7, 128.6, 129.2, 135.0, 142.1, 142.8, 146.4, 147.4, 149.8, 150.7, 154.3, 155.1; MS-IT-TOF [M + H]^+^ = 600 (100%), HRMS (IT TOF): *m*/*z* Calcd for C_37_H_22_N_5_S_2_ [M + H]^+^ = 600.1317, Found 600.1323; UV-Vis (methanol; λ [nm] (logε)): 332 (3.59), 313 (3.79), 298 (3.87), 272 (4.18), 249 (4.47), 206 (4.35); IR (ATR): $\tilde{\nu }$ = 3053, 2926, 2215, 1466, 1313, 1239, 742, 633 cm^−1^; (Supporting Information Figure S25).

*1,10-Phenanthrolin-5-amine* (**5l**) CP/MAS ^13^C-NMR δ = 101.4, 122.9, 130.5, 132.4, 140.8, 142.9, 145.4, 146.5, 152.7; CP/MAS ^15^N NMR δ = −317.08, −78.63, −75.62; (Supporting Information Figure S27).

### 3.7. Synthesis of 4,7-di(9H-carbazol-9-yl)-9-oxo-9,10-dihydro-1,10-phenanthroline-5-carbonitrile (***6a***) and 2-oxo-4,7-di(10H-phenothiazin-10-yl)-1,2-dihydro-1,10-phenanthroline-5-carbonitrile (***6b***)

Compounds **5m** or **5n** (3.5 mmol) dissolved in a mixture composed of THF and 10% sodium hydroxide (55 mL) were stirred for 4 days at 50 °C. The cooled solution was acidified to neutral pH with concentrated hydrochloric acid. After solvent evaporation, the crude product was purified by column chromatography on silica gel using methanol/dichloromethane as eluent to afford a white powder, and finally crystallization from the mixture of CH_2_Cl_2_ and hexane to yield solids as follows:

Compound **6a**: yellowish 0.6 g (1.1 mmol, 31%); m.p_dec_. = 195–200 °C; ^1^H-NMR (CDCl_3_; 400.2 MHz) δ = 6.90 (d, ^3^*J*_H,H_ = 7.3 Hz, 2H, aromatic), 7.14 (s, 1H, aromatic), 7.21 (d, ^3^*J*_H,H_ = 8.0 Hz, 2H, aromatic), 7.29–7.45 (m, 8H, aromatic), 7.65 (s, 1H, aromatic), 7.83 (d, ^3^*J*_H,H_ = 4.3 Hz, 1H, aromatic), 8.16–8.17 (m, 4H, aromatic), 9.27 (d, ^3^*J*_H,H_ = 4.3 Hz, 1H, aromatic), 11.24 (bs, 1H, NH); ^13^C{^1^H}-NMR (CDCl_3_; 100.5 MHz) δ = 100.8, 109.1, 110.0, 115.5, 115.7, 121.0, 121.2, 121.5, 121.7, 124.2, 124.4, 124.5, 126.3, 126.7, 126.8, 126.9, 133.5, 139.0, 140.1, 140.5, 142.2, 143.6, 146.5, 151.8, 161.7; MS-IT-TOF [M + H]^+^ = 552.1830 (100%), [M − H]^-^ = 550.1668 (100%), HRMS (IT TOF): *m*/*z* Calcd for C_37_H_22_N_5_O [M + H]^+^ = 552.1824, Found 552.1830; UV-Vis (methanol; λ [nm] (logε)): 374 (3.46), 355 (3.57), 329 (3.82), 316 (3.76), 292 (4.27), 287 (4.25), 282 (4.27), 247 (4.31), 231 (4.62), 210 (4.33); IR (ATR): $\tilde{\nu }$ = 3051, 2360, 2218, 1675, 1446, 1226, 747, 723 cm^−1^; CCDC 1,917,090 (Supporting Information Figure S28). 

Compound **6b:** yellowish 1.0 g (1.6 mmol, 46%); mp_dec._ = 220–230 °C; ^1^H-NMR (CDCl_3_; 400.2 MHz) δ = 5.63 (d, ^3^*J*_H,H_ = 8.2 Hz, 2H, aromatic), 6.63-6.58 (m, 2H, aromatic), 6.66 (dd, ^3^*J*_H,H_ = 7.8 Hz, ^4^*J*_H,H_ = 0.9 Hz, 2H, aromatic), 6.77 (t, ^3^*J*_H,H_ = 7.5 Hz, 2H, aromatic), 6.90–7.00 (m, 6H, aromatic), 7.12-7.18 (m, 2H, aromatic), 7.25 (s, 1H, aromatic), 7.84 (d, ^3^*J*_H,H_ = 4.5 Hz, 1H, aromatic), 8.45 (s, 1H, aromatic), 9.27 (d, ^3^*J*_H,H_ = 4.6 Hz, 1H, aromatic), 11.11 (bs. 1H, NH); ^13^C{^1^H}-NMR (CDCl_3_; 100.5 MHz) δ = 101.0, 115.1, 116.2, 116.7, 117.5, 119.8, 122.6, 123.5, 124.5, 127.0, 127.2, 127.5, 127.9, 128.5, 129.7, 129.8 134.1, 139.6, 141.0, 141.9, 142.1, 146.9, 149.2, 153.0, 161.7; MS-IT-TOF [M + H]^+^ = 616.1271 (100%), [M − H]^-^ = 614.1110 (100%), HRMS (IT TOF): *m*/*z* Calcd for C_37_H_20_N_5_OS [M − H]^−^ = 614.1109, Found 614.1110; UV-Vis (methanol; λ [nm] (logε)): 339 (3.94), 295 (4.52), 284 (4.38), 273 (4.18), 251 (4.79), 205 (4.59); IR (ATR): $\tilde{\nu }$ = 3059, 2974, 2360, 2216, 1673, 1461, 1312, 743, 648 cm^−1^; CCDC 1,919,692 (Supporting Information Figure S29).

### 3.8. Electrochemical Setup

Cyclic voltammetry was measured using a potentiostat PGSTAT12 (Metrohm Autolab B.V., Utrecht, The Netherlands) and a three-electrode electrochemical cell with an Ag|AgCl|1M LiCl reference electrode separated from the test solution by a salt bridge. A glassy carbon electrode (with diameter 0.7 mm) and a platinum wire were the working and counter electrodes, respectively. Oxygen was removed from the solution by passing a stream of argon. Stock solution of analyte was prepared in degassed anhydrous 99.8% acetonitrile (ACN, anhydrous, Sigma-Aldrich, content of water < 0.001%) supplied under argon. Tetrabutylammonium hexafluorophosphate (Sigma Aldrich) was used as a supporting electrolyte and dried before use.

### 3.9. Molecular Orbital Calculations

Theoretical calculations of frontier molecular orbital energies were performed using the density functional theory (DFT) calculations employing the B3LYP functional and 6-31G* basis set for all atoms with Spartan’14, v.1.1.8 software (Wavefunction, Inc., Irvine, CA USA). The spatial distribution of HOMO and LUMO orbitals was calculated for the geometry optimized molecules in vacuum.

## 4. Conclusions

This research has focused on the synthesis of 27 4,7-disubstituted-1,10-phenanthrolines, including their 4,7-dichloro-, 4,7-di(9*H*-carbazol-9-yl)-, 4,7-di(10*H*-phenothiazin-10-yl)- and 4,7-di(pyrrolidin-1-yl) derivatives, giving 23 novel compounds. The presented protocols allowed us to synthesize the targeted compounds more efficiently, with yields up to 96%. The structures of the obtained molecules were proved by a combination of varies techniques, such as NMR, GC-MS, MS, HRMS, UV-Vis and X-ray crystallography. A variety of substituents (methyl, halogen (F, Cl and Br), CN, 9*H*-carbazole, pyrrolidine, 10*H*-phenothiazine, COOEt and COOH groups) were chosen in order to represent different electronic features. For the first time ^15^N CP/MAS-NMR spectra of selected 4,7-disubstituted-1,10-phenanthroline derivatives were elucidated to differentiate the nitrogen nucleus and to give an insight into their characteristics. The electrochemical studies showed the influence of substituents on the redox properties of synthesized compounds. Compounds with methyl as **R** substituent were the most difficult ones to reduce. On the contrary, compounds substituted with 9*H*-carbazole as **R^2^** had the highest oxidation potentials and were the most stable ones against oxidative processes. Compounds substituted with phenothiazine and pyrrolidine as **R^2^** were the most easily oxidized due to the oxidation of the substituent **R^2^**. Phenothiazine derivatives were also stronger electron acceptors and were more facile to reduction than other compounds. Regarding the largest potential gap, methylated compounds **4g**, **4k** and compound **5f** containing 9*H*-carbazole are the most stable structures against oxidative and reductive processes.

## Figures and Tables

**Table 1:** Chemical structures and HOMO and LUMO distribution of selected compounds.

	HOMO	LUMO
**4a**	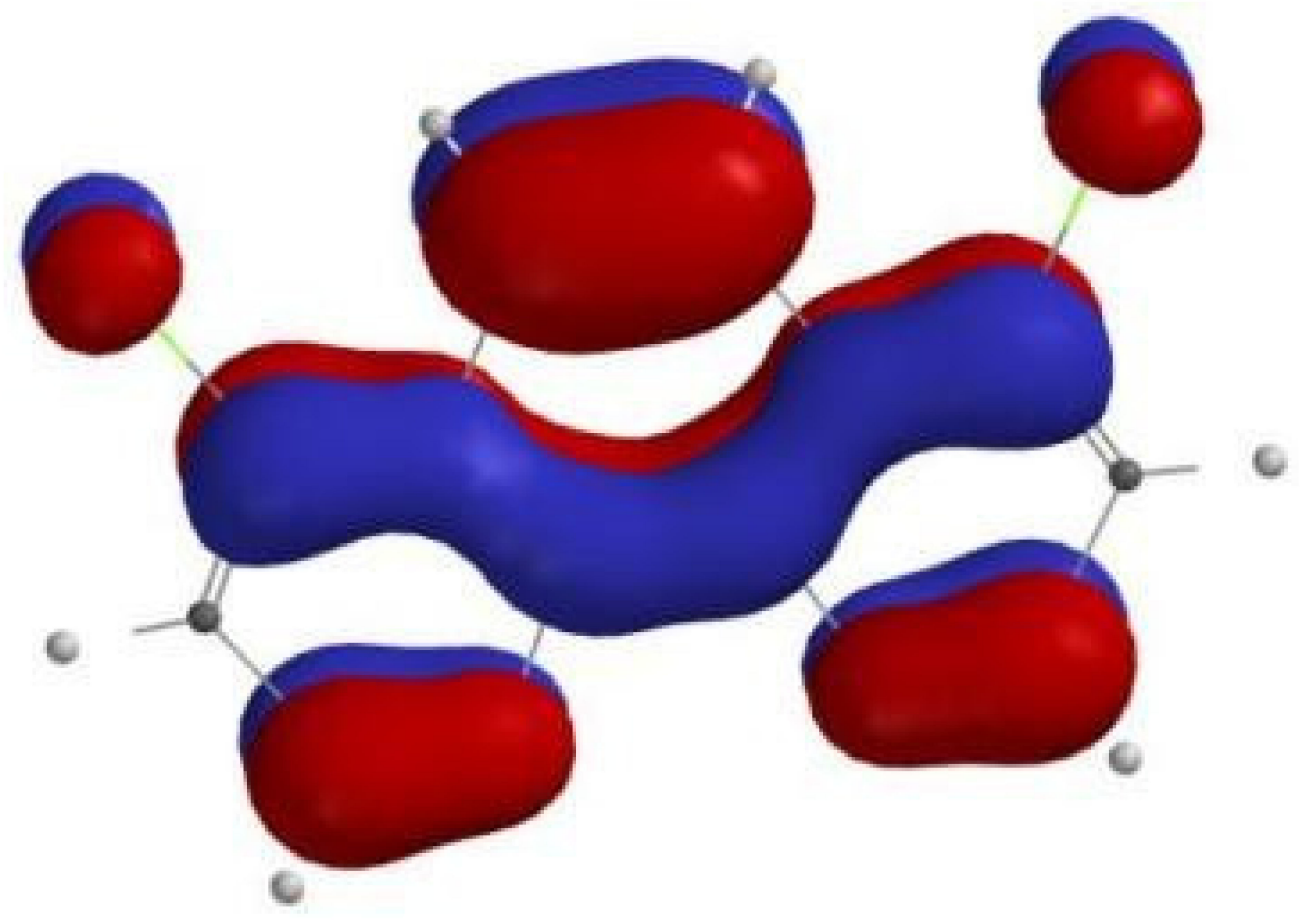	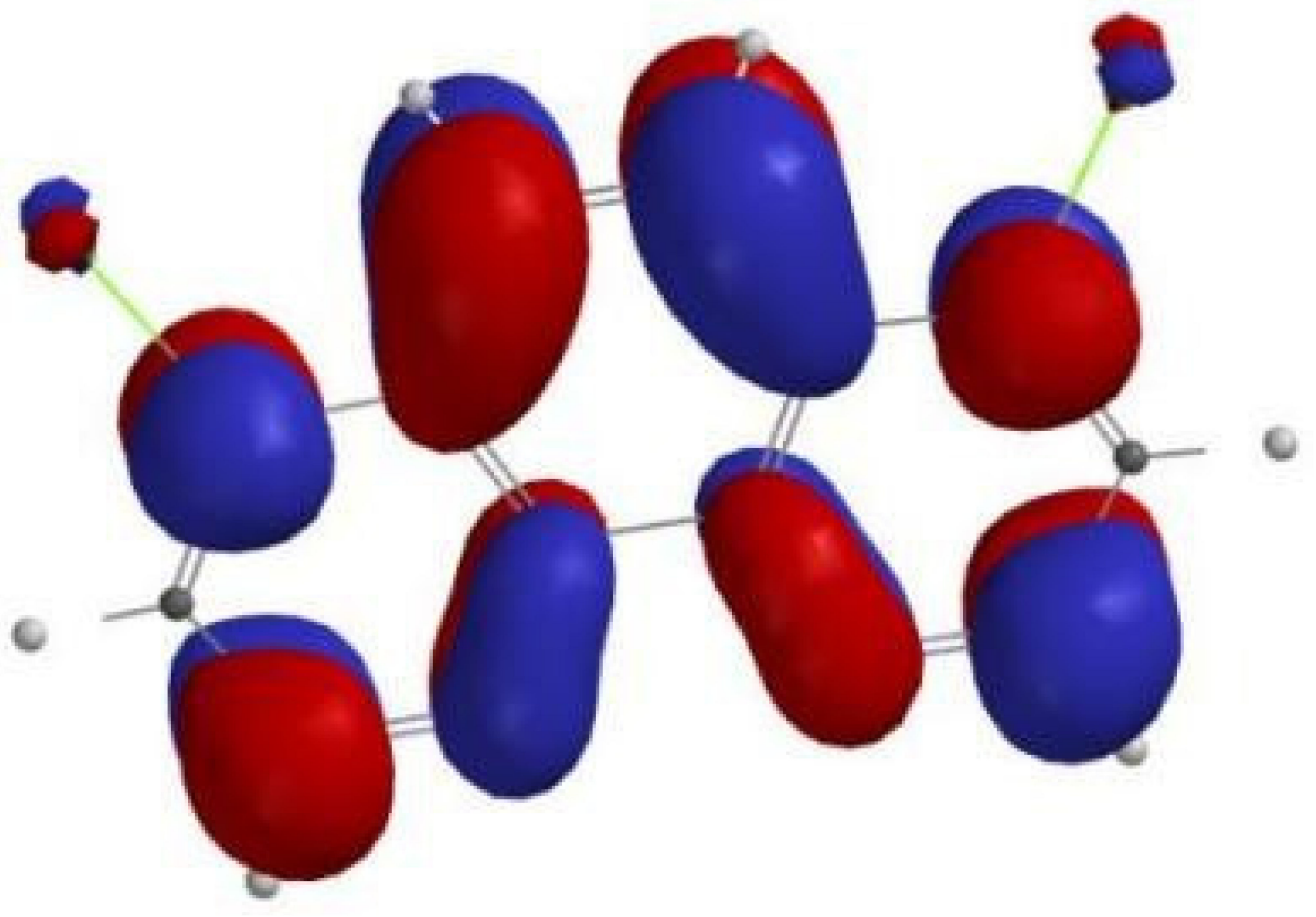
**4n**	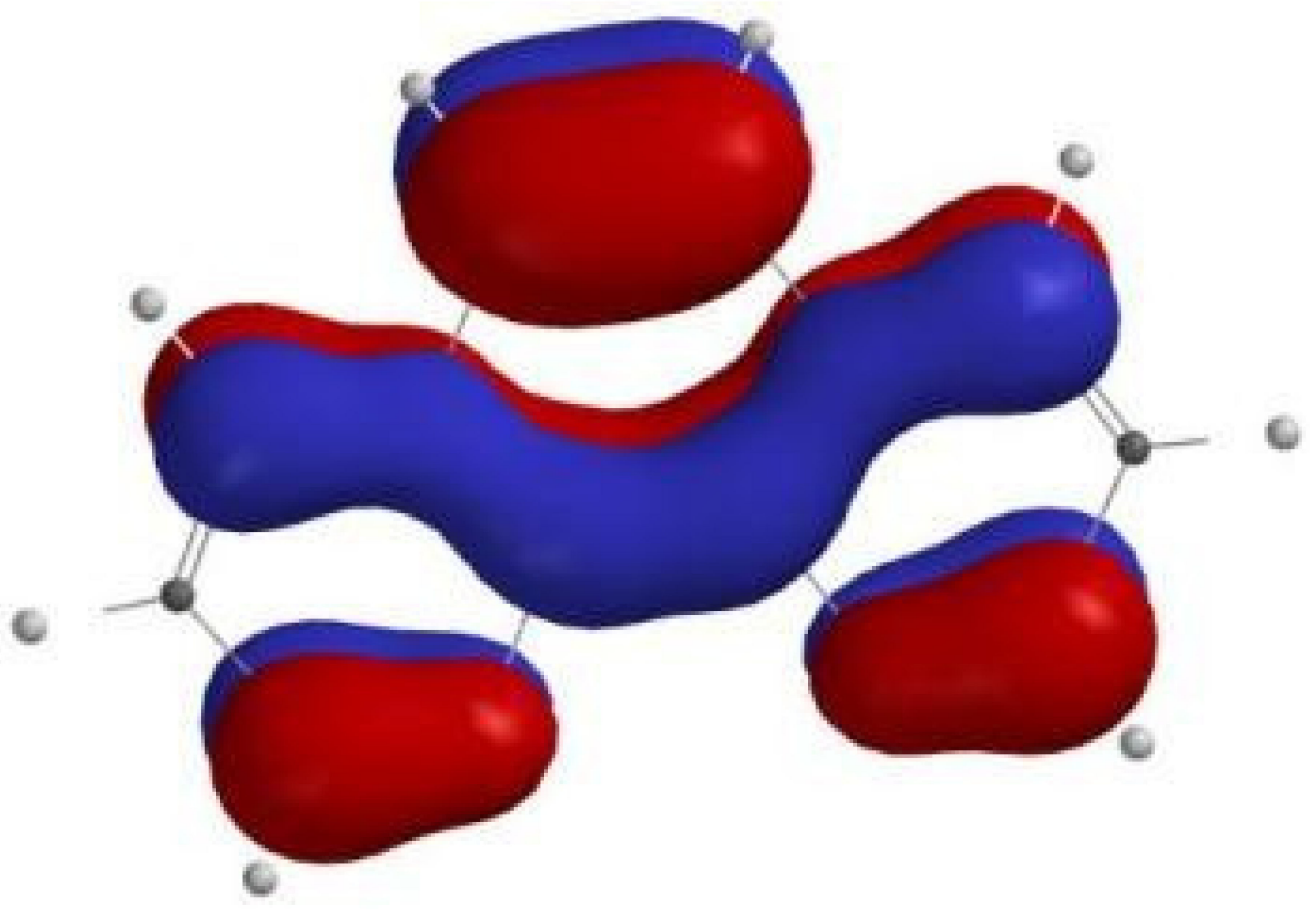	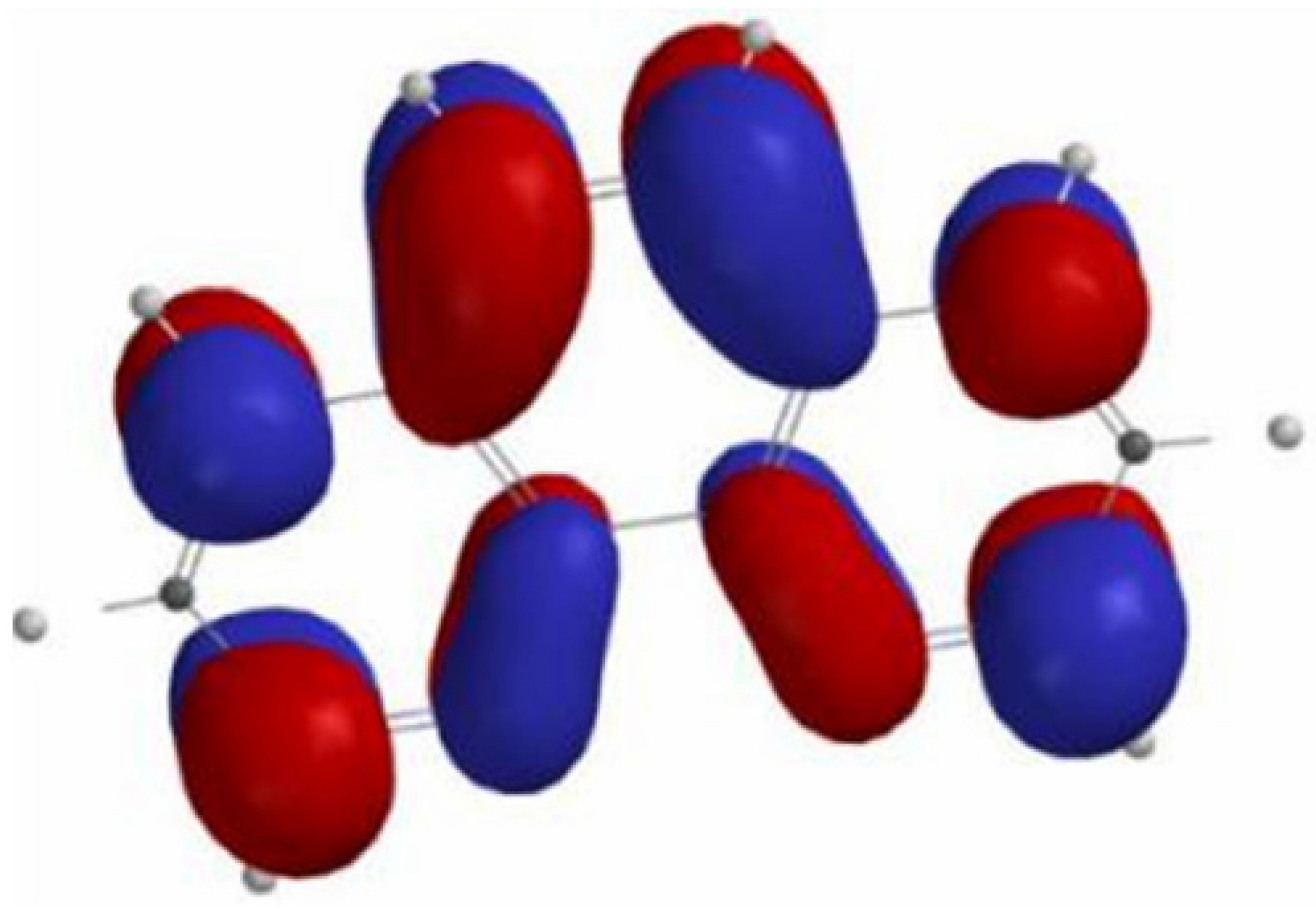
**5a**	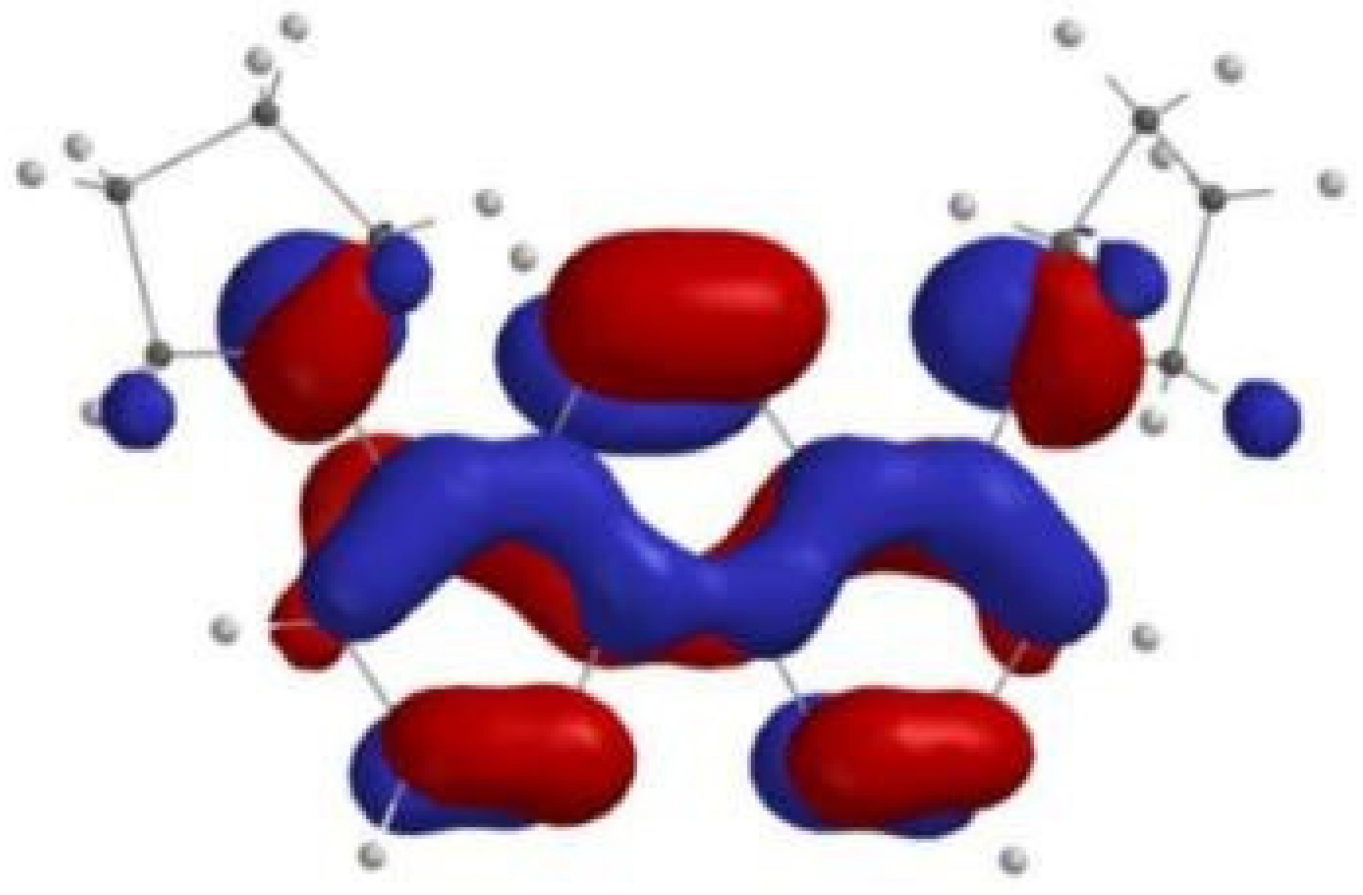	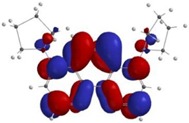
**5f**	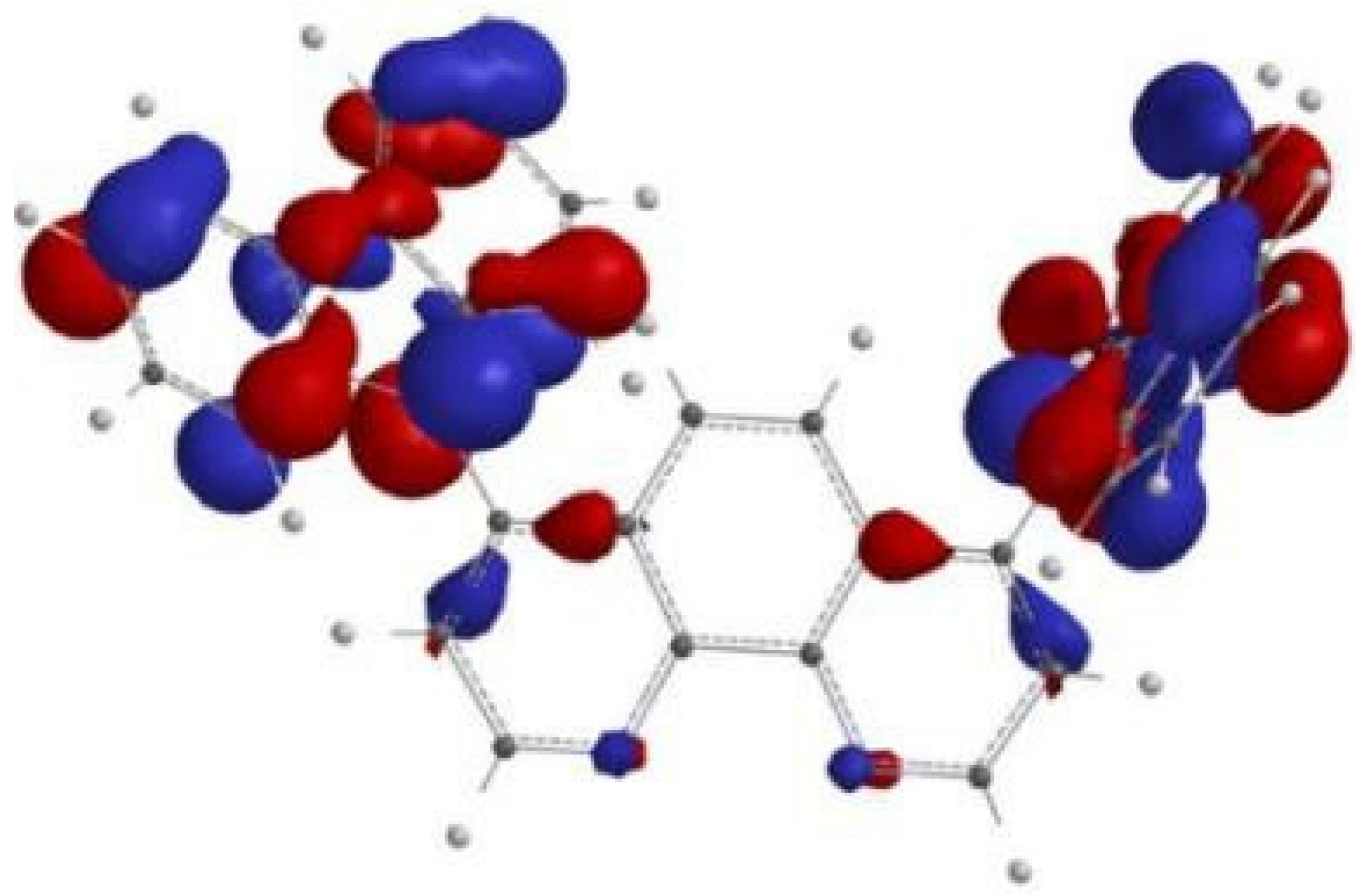	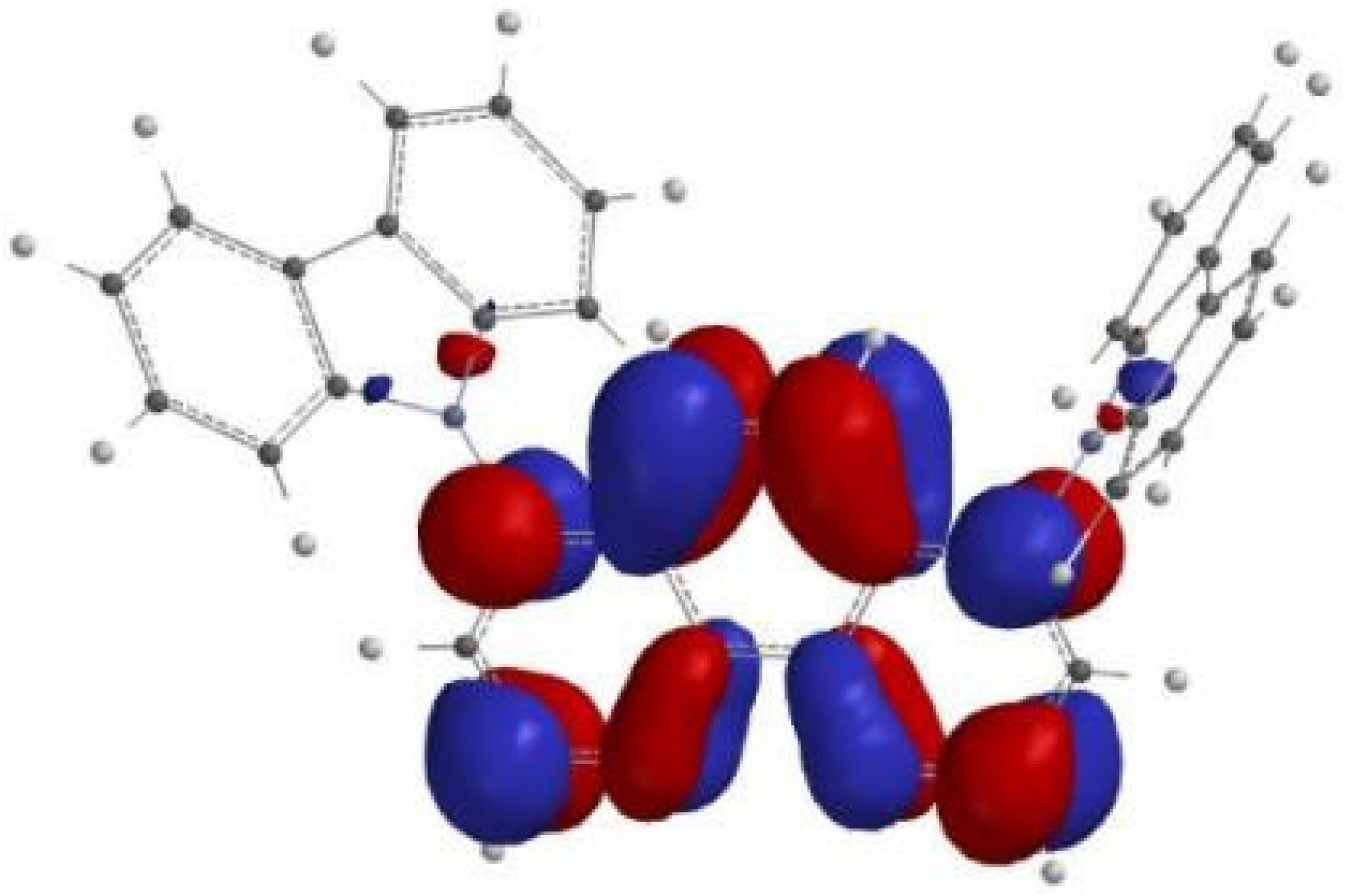
**5i**	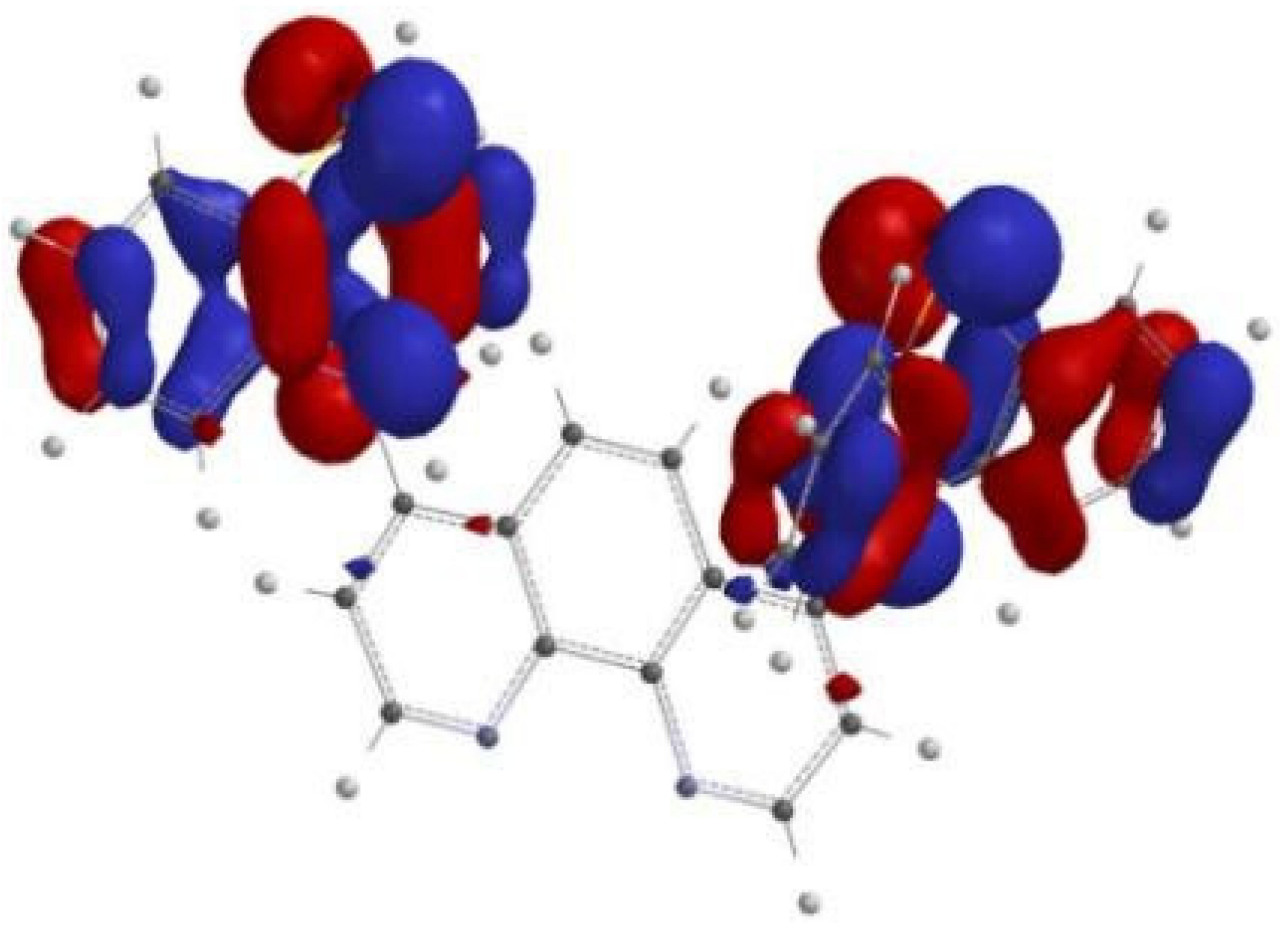	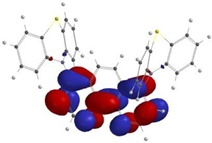

**Table 2:** Experimentally obtained electrochemical data (the first oxidation *E*_p1_^ox^ and the first reduction potential *E*_p1_^red^) at glassy carbon electrode, and calculated energies of HOMO and LUMO by DFT (B3LYP/6-31G*).

No.	*E*_p1_^ox^/V	*E*_p1_^red^/V	Dipole *µ* (calc.)/D	*E*^HOMO^/eV	*E*^LUMO^/eV
**4a**	1.181	−1.572	0.74	−6.56	−1.89
**4d**	1.212	−1.471	1.12	−6.36	−1.79
**4g**	1.221	−1.670	0.47	−6.31	−1.66
**4i**	1.253	−1.330	2.14	−6.31	−1.90
**4b**	1.312	−1.331	0.46	−6.51	−1.97
**4k**	1.601	−1.582	0.24	−6.14	−1.60
**4n**	1.971	−1.911	3.23	−6.25	−1.41
**5k**	0.668	−1.185	0.39	−5.08	−1.75
**5c**	0.900	−1.560	*4.37*	−5.21	−0.85
**5a**	0.905	−1.505	*5.38*	−5.41	−1.05
**5i**	0.951	−1.231	0.47	−5.17	−1.76
**5j**	0.956	−1.205	2.47	−5.04	−1.95
**5h**	1.238	−1.235	1.53	−5.59	−1.74
**5g**	1.325	−1.205	0.61	−5.55	−1.90
**5f**	1.575	−1.365	1.42	−5.61	−1.80

## Supplementary Materials

The following are available online at https://www.mdpi.com/1420-3049/24/22/4102/s1. CCDC 1479401 for **5d**, CCDC 1917090 for **6a** and CCDC 1919692 for **6b** contains the supplementary crystallographic data for this paper. These data can be obtained free of charge via http://www.ccdc.cam.ac.uk/conts/retrieving.html (or from the CCDC, 12 Union Road, Cambridge CB2 1EZ, UK; Fax: +44 1223 336033; E-mail: deposit@ccdc.cam.ac.uk)”. This text may be included in the experimental section or as a suitably referenced endnote.
