# Supplementary material for: Synthesis and Electrochemical and Spectroscopic Characterization of 4,7-diamino-1,10-phenanthrolines and Their Precursors
Source: Molecules. 2019 Nov 13;24(22):4102. doi: 10.3390/molecules24224102 (PMC6891714; doi:10.3390/molecules24224102)
Supplement: Supplementary file 1 [file molecules-24-04102-s001.pdf]

Supporting Information for

Synthesis and electrochemical and spectroscopic characterization of 4,7-diamino-1,10-phenanthrolines and their precursors

Jacek E. Nycz [1],* Jakub Wantulok [1], Romana Sokolova [2], Lukasz Pajchel [3], Marek Stankevič [4], Marcin Szala [5], Jan Grzegorz Malecki [1] and Daniel Swoboda [1]

Table of Contents

Table S1. Crystal data and structure refinement details of compounds **5d**, **6a**, **6b** S2

Table S2. Hydrogen bonds for compounds **5d**, **6a** and **6b** (Å and °) S2

Table S3. C-Cl…π stacking interactions in compound **5d** and π…π interaction in **6a** S3

Table S4. The experimental ^{1}H chemical shifts of compounds **4**, **5** and **6** in $CDCl_3$ S3

Table S5. The experimental $^{13}C\{^{1}H\}$ chemical shifts of compounds **4**, **5** and **6** in $CDCl_3$ S3

Table S6. The experimental CP/MAS ^{13}C chemical shifts of selected compounds **4** and **5** S4

Table S7. The experimental CP/MAS ^{15}N chemical shifts of selected compounds **4** and **5** S5

Table S8. Calculated HOMO and LUMO distribution of selected compounds **4** S6

Table S9. Calculated HOMO and LUMO distribution of selected compounds **5** S7

Fig. S1. Natural atomic charges of compounds **5m** (left) and **5n** (right) S8

Fig. S2. The plot of the electrostatic potential for compounds **5m** (left) and **5n** (right) S8

^{1}H, ^{13}C and HMQC; ^{15}N NMR Spectra and MS for compounds **4** S9-S37

^{1}H, ^{13}C and HMQC; ^{15}N NMR Spectra and MS for compounds **5** S38-S70

^{1}H, ^{13}C and HMQC; ^{15}N NMR Spectra and MS for compounds **6** S71-S74

Table S1. Crystal data and structure refinement details of compounds **5d**, **6a** and **6b**.

	**5d**	**6a**	**6b**
Empirical formula	$C_{22}H_{25}FN_4$, 2($CHCl_3$)	$C_{37}H_{21}N_5O$, 2(C_4H_8O),0.5($C_2H_8O_2$)	$C_{37}H_{21}N_5OS_2$, 2(C_4H_8O)
Formula weight	603.19	727.84	759.91
Temperature [K]	295(2)	295(2)	295(2)
Wavelength (Å)	0.71073	0.71073	0.71073
Crystal system	triclinic	triclinic	monoclinic
Space group	*P*–1	*P*–1	$P2_1/c$
Unit cell dimensions			
a [Å]	9.1767(13)	12.1394(7)	9.0665(4)
b [Å]	10.3249(14)	12.6397(5)	27.0123(10)
c [Å]	14.9935(12)	12.9635(6)	15.0843(5)
α [°]	83.725(9)	89.230(4)	90
β [°]	82.567(9)	75.454(4)	91.867(4)
γ [°]	87.109(12)	79.332(4)	90
Volume [$Å^3$]	1399.3(3)	1890.91(16)	3692.3(2)
Z	2	2	4
Calculated density [Mg/m^3]	1.432	1.278	1.367
Absorption coefficient [mm^{-1}]	0.642	0.083	0.195
F(000)	620	768	1592
Crystal dimensions [mm]	0.36 x 0.08 x 0.06	0.17 x 0.08 x 0.07	0.37 x 0.15 x 0.14
θ range for data collection [°]	3.33 − 25.05	3.57 − 27.95	3.46 – 29.56
Index ranges	$-12 \le h \le 12$<br>$-12 \le k \le 14$<br>$-18 \le l \le 21$	$-16 \le h \le 15$<br>$-15 \le k \le 14$<br>$-17 \le l \le 14$	$-10 \le h \le 11$<br>$-36 \le k \le 27$<br>$-20 \le l \le 15$
Reflections collected	11285	16918	22276
Independent reflections	6529 [R(int) = 0.0784]	8928 [R(int) = 0.0412]	9010 [R(int) = 0.0310]
Data / restraints / parameters	6529 /0/328	8928/2/516	9010/0/500
Goodness-of-fit on F^2	0.912	1.019	1.034
Final R indices [I>2σ(I)]*	R_1 = 0.0650, wR_2 = 0.1458	R_1 = 0.0667, wR_2 = 0.1589	R_1 = 0.0604, wR_2 = 0.1438
R indices (all data)	R_1 = 0.1830, wR_2 = 0.2091	R_1 = 0.1391, wR_2 = 0.1999	R_1 = 0.0991, wR_2 = 0.1665
Largest diff. Peak and hole	0.530 /-0.437	0.312/-0.266	0.401/-0.329
CCDC number	1479401	1917090	1919692

*Structure was refined on F_o^2: $wR2 = [\sum[w(F_o^2 - F_c^2)^2] / \sum w(F_o^2)^2]^{1/2}$, where $w^{-1} = [\sum(F_o^2) + (aP)^2 + bP]$ and $P = [\max(F_o^2, 0) + 2F_c^2]/3$.

Table S2. Hydrogen bonds for compounds **5d** and **6a** (Å and °).

D-H···A	d(D–H)	d(H···A)	d(D···A)	<(DHA)
**5d**				
C(19)–H(19B)…F(1)	0.97	2.16	2.715(5)	116.0
C(23)–H(23)…N(1)	0.98	2.43	3.281(6)	145.2
C(23)–H(23)…N(2)	0.98	2.34	3.205(5)	147.2
**6a**				

N(1)–H(1)…O(2) #1	0.86	2.10	2.902(6)	156.0
N(1)–H(1)…N(2)	0.86	2.32	2.691(1)	106.0
C(5)–H(5)…N(3)	0.93	2.62	2.927(9)	100.0
C(25)–H(25)…O(2) #2	0.93	2.49	3.301(8)	146.0
C(42)–H(42A)…O(1) #3	0.97	2.59	3.341(4)	135.0
**6b**				
N(1)–H(1)…N(2)	0.87(3)	2.37(3)	2.700(3)	103(2)
N(1)–H(1)…O(2) #4	0.87(3)	2.38(3)	3.132(3)	146(2)
C(5)–H(5)…N(4)	0.93	2.54	2.868(3)	101.0
C(10)–H(10)…O(3)	0.93	2.59	3.499(7)	1.66.0

Symmetry code: #1 = x,1+y,z; #2 = 1-x,1-y,1-z; #3 = x,-1+y,z; #4 = -x,1-y,1-z

Table S3. C-X…π stacking interactions in compounds **5d**, **6b** and π…π interaction in **6a**, **6b**.

Y-X(I)•••Cg(J)	X(I)•••Cg(J) [Å]	X-Perp [Å]	γ [°]	Y-X(I)•••Cg(J) [°]
**5d**				
Cg(1): N(2)-C(10)-C(9)-C(8)-C(7)-C(11); Cg(2): C(4)-C(5)-C(6)-C(7)-C(11)-C(12)				
C(24)-Cl(4)•••Cg(1)#1	3.882(2)	-3.619	21.20	108.06(17)
C(24)-Cl(5)•••Cg(2)#1	3.942(2)	-3.767	17.13	132.07(17)
**6b**				
Cg(1): S(2)-C(31)-C(26)-N(5)-C(37)-C(32)				
C(21)-H(21)•••Cg(1)#2	2.690	2.674	5.67	135.0
C(13)-N(3)•••Cg(1)	3.180	-3.137	9.45	81.05

Cg(I)•••Cg(J)	Cg(I)•••Cg(J) [Å]	α[°]	β[°]	γ [°]	Cg(I)-Perp [Å]	Cg(J)-Perp [Å]
**6a**						
Cg(1): N(1)-C(12)-C(4)-C(3)-C(2)-C(1); Cg(2): N(2)-C(11)-C(7)-C(8)-C(9)-C(10)						
Cg(1)•••Cg(2) #3	3.887	0.00	8.06	85.40	-4.773	-5.179
**6b**						
Cg(2): C(20)-C(21)-C(22)-C(23)-C(24)-C(25)						
Cg(2)•••Cg(2) #4	3.7791(14)	0.00	13.89	89.72	-5.068	-5.068

α = dihedral angle between *Cg*(I) and *Cg*(J); *Cg*(I)-Perp = Perpendicular distance of *Cg*(I) on ring J; *Cg*(J)-Perp = perpendicular distance of *Cg*(J) on ring I; β = angle *Cg*(I)→*Cg*(J) vector and normal to ring I; γ = angle *Cg*(I) →*Cg*(J) vector and normal to plane J

Symmetry code: #1 = 1-x,2-y,-z; #2 = x,3/2-y,-1/2+z; #3 = 1-x,2-y,1-z; #4 = -x,1-y,-z

Table S4. The experimental ^{1}H chemical shifts of compounds **4**, **5** and **6** in $CDCl_3$ (* in D_2O/KOD).

	**Aromatic**	**Others**
**4a**	7.71, 8.24, 9.06	–
**4b**	7.75, 7.77, 7.94, 9.02, 9.08	–
**4c**	7.85, 8.41, 9.12, 9.18	–
**4d**	7.69, 7.72, 8.04, 8.99, 9.00	3.15
**4e**	7.84, 8.88, 9.13, 9.19	–
**4f***	6.47, 6.61, 7.69, 8.05, 8.28	–
**4g**	7.63, 8.24	2.93
**4h**	7.67, 7.68, 7.86	2.97, 3.01

**4i**	7.63, 7.66, 8.27	2.92, 2.94
**4j**	7.63, 7.69, 8.58	2.93, 2.95
**4k**	7.62, 7.95	2.92, 2.96, 3.11
**4l**	7.72, 7.73, 8.79	2.96, 2.99
**4m**	7.65, 8.31	1.43, 2.93, 2.94, 4.49
**5a**	6.69, 7.93, 8.72	2.03, 3.67
**5b**	6.67, 6.90, 7.92, 8.74	1.99, 2.07, 3.53, 3.70
**5c**	6.61, 7.90	2.04, 2.77, 3.68
**5d**	6.58, 6.65, 7.45	1.96, 2.01, 2.74, 2.76, 3.47, 3.61
**5e**	6.61, 6.85, 7.63	1.97, 2.03, 2.68, 2.76, 2.77, 3.35, 3.68
**5f**	7.06, 7.28−7.36, 7.85, 8.15, 9.49	−
**5g**	7.03, 7.09, 7.30−7.43, 7.91, 8.18, 9.47, 9.54	−
**5h**	6.93, 7.10, 7.22, 7.29−7.40, 7.68, 7.80, 8.16, 8.18, 9.43	1.60
**5i**	6.10, 6.76, 6.82, 7.07, 7.88, 8.15, 9.50	−
**5j**	5.84, 6.17, 6.69, 6.75, 6.80, 6.85, 6.98, 7.08, 7.82, 7.87, 7.92, 9.47, 9.52	−
**5k**	5.80, 6.11, 6.65−6.85, 6.96, 7.07, 7.70, 7.83, 7.92, 9.45	2.91
**5m**	6.93, 7.07, 7.31-7.43, 7.88, 7.93, 8.05, 8.18, 9.55, 9.60	−
**5n**	5.67, 6.10, 6.67, 6.77, 6.82, 6.90, 6.98, 7.13, 7.89, 8.01, 8.76, 9.58, 9.65	−
**6a**	6.90, 7.14, 7.21, 7.29-7.45, 7.65, 7.83, 8.16-8.17, 9.27	11.24
**6b**	5.63, 6.63-6.58, 6.66, 6.77, 6.90-7.00, 7.12-7.18, 7.25, 7.84, 8.45, 9.27	11.11

For clarity the coupling constants are omitted. **5l** purchased from Sigma–Aldrich

Table S5. The experimental $^{13}C\{^1H\}$ chemical shifts of compounds **4**, **5** and **6** in $CDCl_3$ (* in D_2O/KOD).

	**Aromatic**	**Others**
**4a**	123.1, 123.9, 126.6, 142.8, 146.9, 150.2	−
**4b**	106.7, 119.9, 124.4, 126.3, 126.8, 140.3, 142.0, 144.7, 148.9, 149.7, 151.0, 156.3	−
**4c**	124.8, 124.9, 125.6, 126.4, 128.1, 130.7, 143.2, 143.3, 144.4, 147.3, 149.9, 150.4	−
**4d**	124.1, 124.9, 126.3, 126.5, 127.2, 134.9, 141.8, 143.1, 146.6, 148.7, 149.5, 149.6	26.5
**4e**	108.1, 118.1, 124.2, 125.2, 125.3, 126.5, 135.4, 142.3, 143.6, 147.5, 148.1, 151.5, 153.4	−
**4f***	111.5, 111.6, 114.8, 120.7, 125.1, 132.5, 138.1, 139.2, 140.7, 149.3, 173.8, 178.0, 179.7	−
**4g**	122.3, 124.4, 125.1, 143.0, 146.2, 160.1	26.0
**4h**	105.6, 118.0, 125.1, 126.1, 140.3, 142.8, 147.0, 154.9, 157.5, 159.1, 161.3	25.3, 25.5
**4i**	122.7, 124.5, 124.6, 125.0, 128.0, 129.0, 142.1, 142.3, 144.8, 147.6, 160.2, 160.3	25.3, 25.7
**4j**	116.2, 123.2, 125.0, 125.1, 127.8, 129.2, 142.1, 143.0, 145.0, 147.2, 160.0, 160.5	25.2, 25.8
**4k**	123.9, 124.5, 124.6, 125.4, 126.9, 133.8, 142.3, 143.1, 144.9, 147.2, 159.0, 159.1	25.2, 25.6, 26.3
**4l**	106.9, 118.4, 122.6, 123.7, 125.6, 127.0, 134.3, 142.2, 143.7, 146.1, 146.8, 161.6, 163.8	25.6, 26.2
**4m**	122.1, 123.5, 123.8, 124.9, 126.0, 129.3, 141.7, 143.6, 146.3, 146.6, 160.5, 161.5, 169.0	14.1, 25.5, 25.9, 62.6
**5a**	105.6, 119.5, 119.6, 148.5, 149.4, 152.9	26.1, 52.4
**5b**	105.9, 107.9, 117.7, 118.5, 122.4, 124.1, 145.2, 147.7, 148.2, 148.9, 152.7, 153.9	25.1, 26.1, 52.1, 52.4

**5c**	105.7, 118.2, 119.0, 146.3, 158.4, 157.5	25.8, 26.0, 52.4
**5d**	102.9, 106.1, 109.9, 117.6, 144.5, 148.7, 152.1, 152.3, 152.9, 154.8, 157.1, 158.7	25.6, 25.6, 25.8, 25.9, 25.9, 51.7, 51.8, 52.0
**5e**	105.8, 109.3, 117.9, 120.8, 122.4, 127.4, 145.1, 146.7, 153.2, 155.4, 156.7, 157.5	21.4, 24.4, 25.6, 25.7, 26.0, 52.0, 52.3
**5f**	110.1, 120.7, 121.0, 123.0, 124.1, 126.5, 126.7, 141.3, 143.5, 148.8, 151.6	–
**5g**	106.5, 109.4, 110.0, 119.6, 120.7, 120.8, 121.0, 121.2, 121.4, 123.6, 124.1, 125.1, 125.5, 126.56, 126.58, 141.1, 141.6, 141.7, 143.2, 146.1, 150.0, 150.9, 152.6, 154.2, 156.8	–
**5h**	109.7, 110.1, 120.70, 120.73, 120.8, 121.0, 123.1, 123.7, 124.0, 124.5, 125.6, 126.2, 126.5, 126.8, 128.2, 133.6, 141.4, 142.2, 142.4, 143.4, 148.0, 149.7, 150.9, 151.2	21.1
**5i**	116.0, 121.1, 123.4, 123.5, 127.1, 127.3, 128.5, 142.9, 146.5, 149.5, 152.0	–
**5j**	106.5, 115.4, 119.8, 121.4, 122.4, 123.3, 127.0, 127.3, 128.2, 128.4, 142.8, 144.3, 145.7, 147.3, 150.6, 151.1, 153.9, 154.7, 156.8	–
**5k**	115.6, 116.0, 119.0, 120.9, 123.0, 123.3, 124.2, 126.8, 127.06, 127.1, 127.2, 127.8, 129.0, 129.4, 134.5, 142.6, 142.8, 145.4, 146.4, 148.6, 151.0, 151.3, 151.7	23.6
**5m**	107.1, 109.2, 109.6, 115.7, 121.0, 121.1, 121.3, 121.7, 123.8, 124.4, 124.5, 125.2, 125.6, 125.8, 126.6, 126.9, 134.4, 141.2, 142.3, 143.1, 144.5, 148.8, 149.6, 153.1, 154.7	–
**5n**	107.5, 115.1, 115.9, 117.6, 119.6, 121.7, 123.4, 124.1, 126.9, 127.1, 127.3, 127.7, 128.6, 129.2, 135.0, 142.1, 142.8, 146.4, 147.4, 149.8, 150.7, 154.3, 155.1	–
**6a**	100.8, 109.1, 110.0, 115.5, 115.7, 121.0, 121.2, 121.5, 121.7, 124.2, 124.4, 124.5, 126.3, 126.7, 126.8, 126.9, 133.5, 139.0, 140.1, 140.5, 142.2, 143.6, 146.5, 151.8, 161.7	–
**6b**	101.0, 115.1, 116.2, 116.7, 117.5, 119.8, 122.6, 123.5, 124.5, 127.0, 127.2, 127.5, 127.9, 128.5, 129.7, 129.8 134.1, 139.6, 141.0, 141.9, 142.1, 146.9, 149.2, 153.0, 161.7	–

For clarity the coupling constants are omitted. **5l** purchased from Sigma–Aldrich

Table S6. The experimental CP/MAS ^{13}C chemical shifts of selected compounds **4** and **5**.

	**Aromatic**	**Others**
**4g**	121.7, 141.3, 143.8, 159.3	25.1
**4k**	122.5, 123.5, 131.0, 139.0, 143.3, 145.3, 158.3	25.0, 27.5
**5c**	104.7, 117.5, 147.3, 149.6, 154.6	26.0, 51.8
**5f**	109.4, 120.2, 122.0, 123.6, 124.8, 127.4, 139.4, 141.2, 147.5, 149.2, 150.3	–
**5h**	111.3, 116.6, 120.7, 123.3, 126.7, 128.2, 133.7, 136.6, 142.0, 147.4, 148.5, 150.3	23.4
**5k**	116.8, 123.4, 124.6, 127.2, 135.3, 141.5, 144.8, 147.1, 149.0, 152.6, 153.5, 155.6	25.9
**5l**	101.4, 122.9, 130.5, 132.4, 140.8, 142.9, 145.4, 146.5, 152.7	–

Table S7. The experimental CP/MAS ^{15}N chemical shifts of selected compounds **4** and **5**.

	**Aromatic**	**Others**
**4g**	−76.15	–
**4k**	−75.52	–
**5c**	−62.66	−291.94
**5f**	−73.05	−250.68
**5h**	−78.17, −54.38	−254.60, −249.48
**5k**	−62.21, −51.07	−273.57

**5l**	−78.63, −75.62	−317.08

Table S8. Calculated HOMO and LUMO distribution of selected compounds **4**.

	**HOMO**	**LUMO**
**4b**	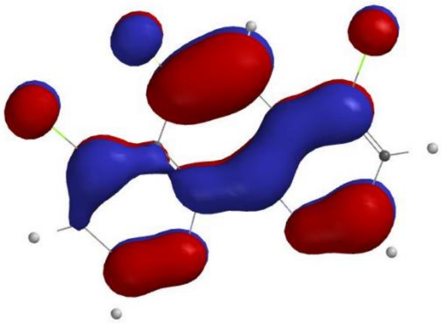	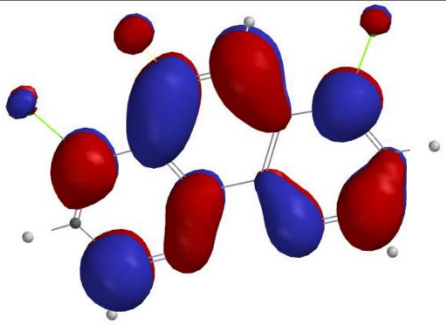
**4d**	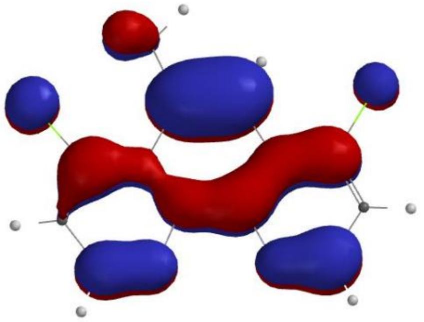	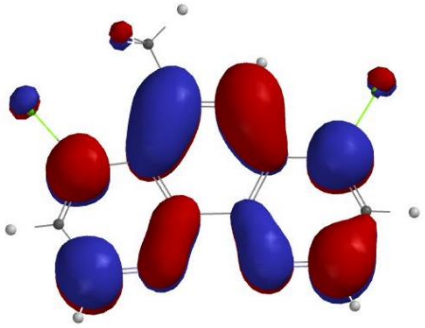
**4g**	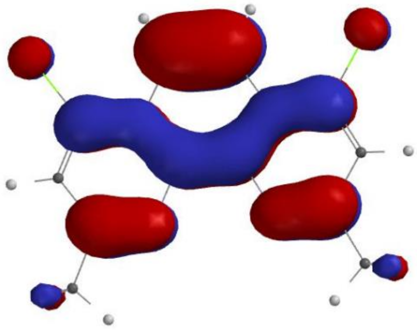	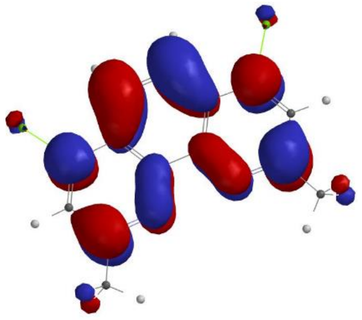
**4i**	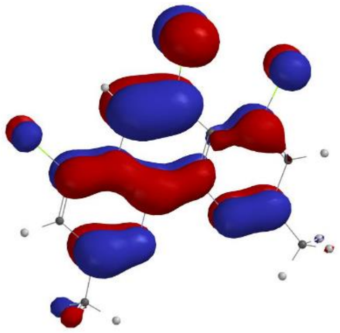	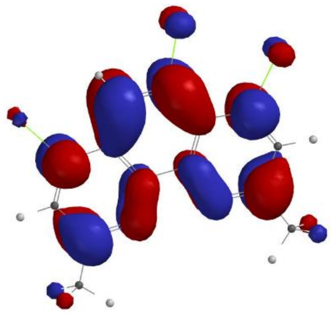

**4k**	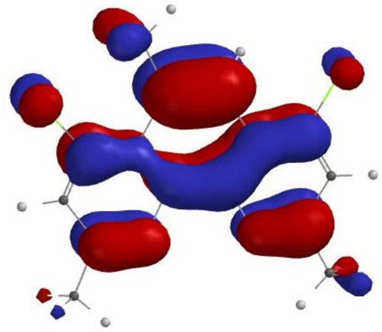	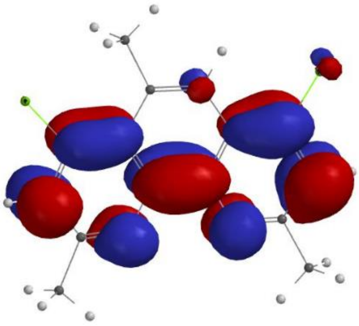

Table S9. Calculated HOMO and LUMO distribution of selected compounds **5**.

	**HOMO**	**LUMO**
**5c**	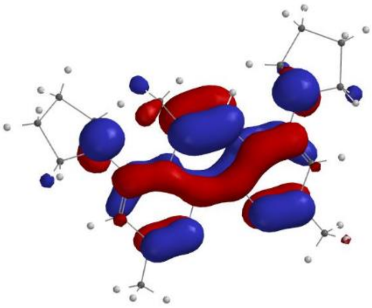	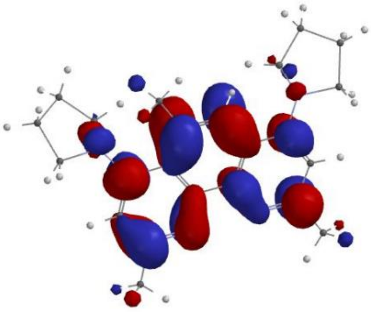
**5g**	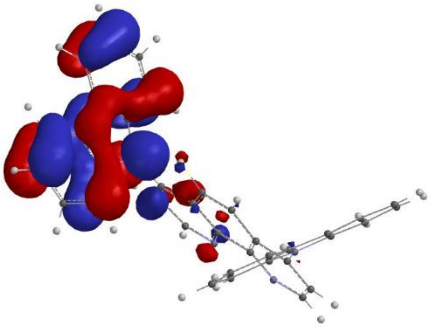	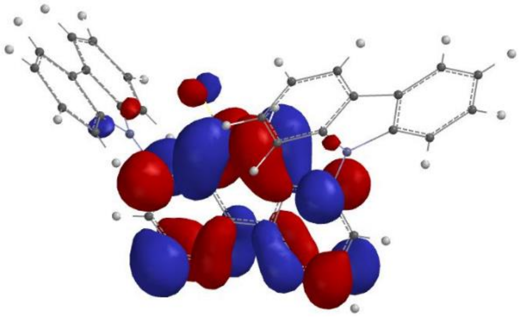
**5h**	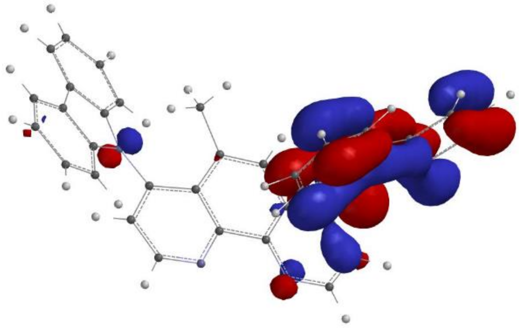	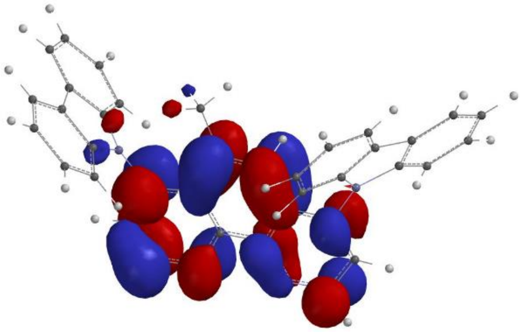

**5j**	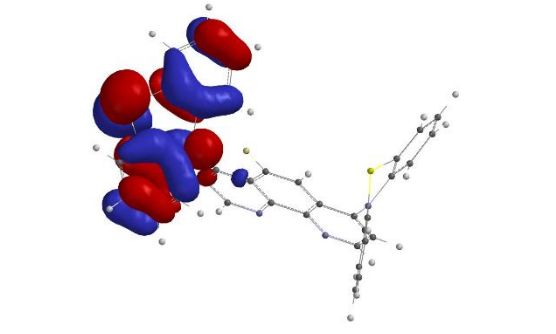	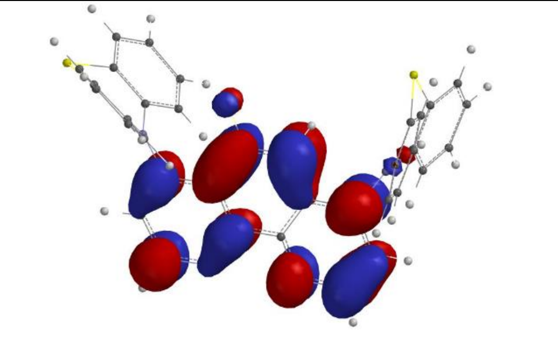
**5k**	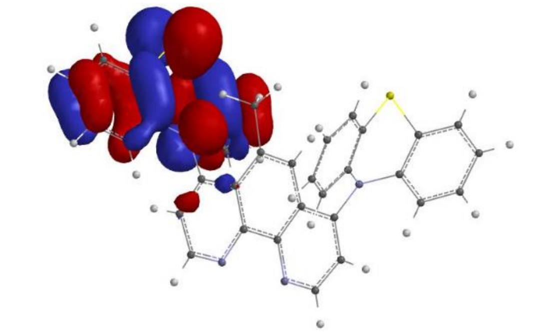	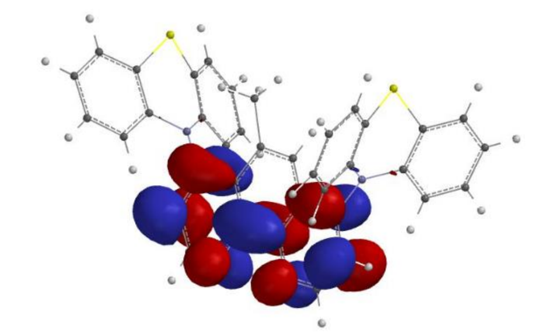

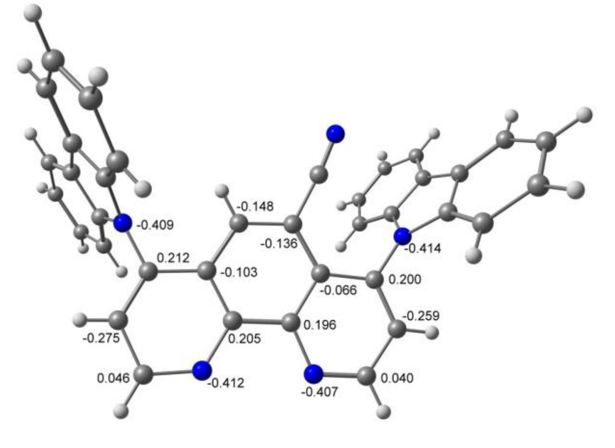	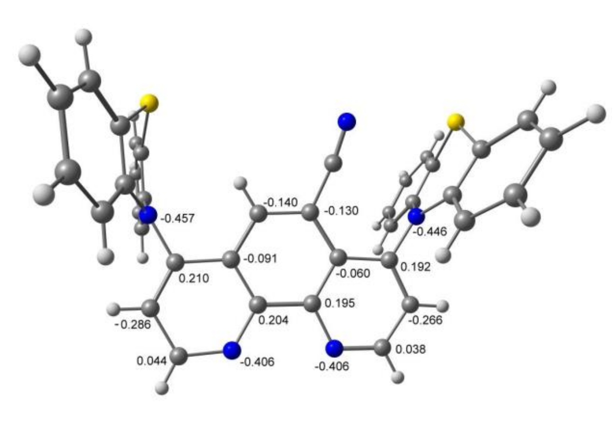
**5m**	**5n**

Fig. S1. Natural atomic charges of compounds **5m** (left) and **5n** (right). #

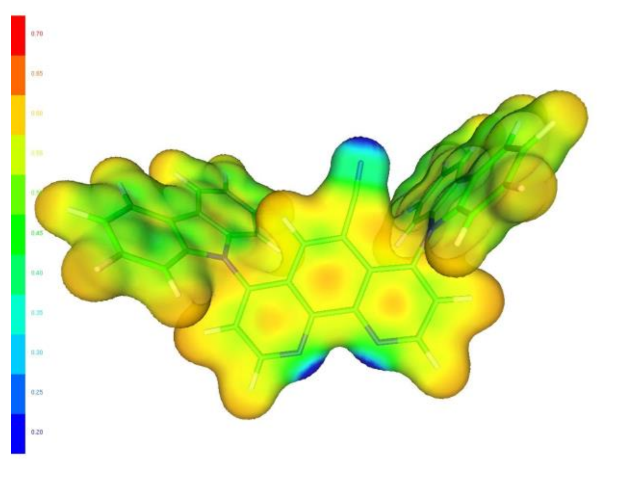	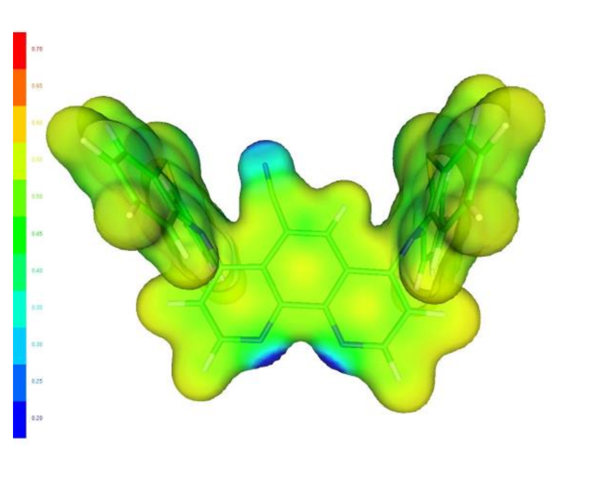

5m	5n

Fig. S2. The plot of the electrostatic potential for compounds **5m** (left) and **5n** (right).#

The calculations were done with the use of the density functional theory (DFT) and were carried out using the Gaussian09 program [1] on B3LYP/6-31g++ level [2, 3]. Molecular geometry of the singlet ground state of the compounds was optimized in the gas phase.

1. Gaussian 09, Revision A.02, Frisch M. J., Trucks G. W., Schlegel H. B., Scuseria G. E., Robb M. A., Cheeseman J. R., Scalmani G., Barone V., Petersson G. A., Nakatsuji H., Li X., Caricato M., Marenich A., Bloino J., Janesko B. G., Gomperts R., Mennucci B., Hratchian H. P., Ortiz J. V., Izmaylov A. F., Sonnenberg J. L., Williams-Young D., Ding F., Lipparini F., Egidi F., Goings J., Peng B., Petrone A., Henderson T., Ranasinghe D., Zakrzewski V. G., Gao J., Rega N., Zheng G., Liang W., Hada M., Ehara M., Toyota K., Fukuda R., Hasegawa J., Ishida M., Nakajima T., Honda Y., Kitao O., Nakai H., Vreven T., Throssell K., Montgomery J. A. Jr., Peralta J. E., Ogliaro F., Bearpark M., Heyd J. J., Brothers E., Kudin K. N., Staroverov V. N., Keith T., Kobayashi R., Normand J., Raghavachari K., Rendell A., Burant J. C., Iyengar S. S., Tomasi J., Cossi M., Millam J. M., Klene M., Adamo C., Cammi R., Ochterski J. W., Martin R. L., Morokuma K., Farkas O., Foresman J. B., and D. J. Fox, Gaussian, Inc., Wallingford CT, 2016.

2. Becke A. D., J.Chem.Phys. 98, 1993 5648-5652.

3. Lee C., Yang W., Parr R.G., Phys. Rev. B 37, 1988, 785-789.

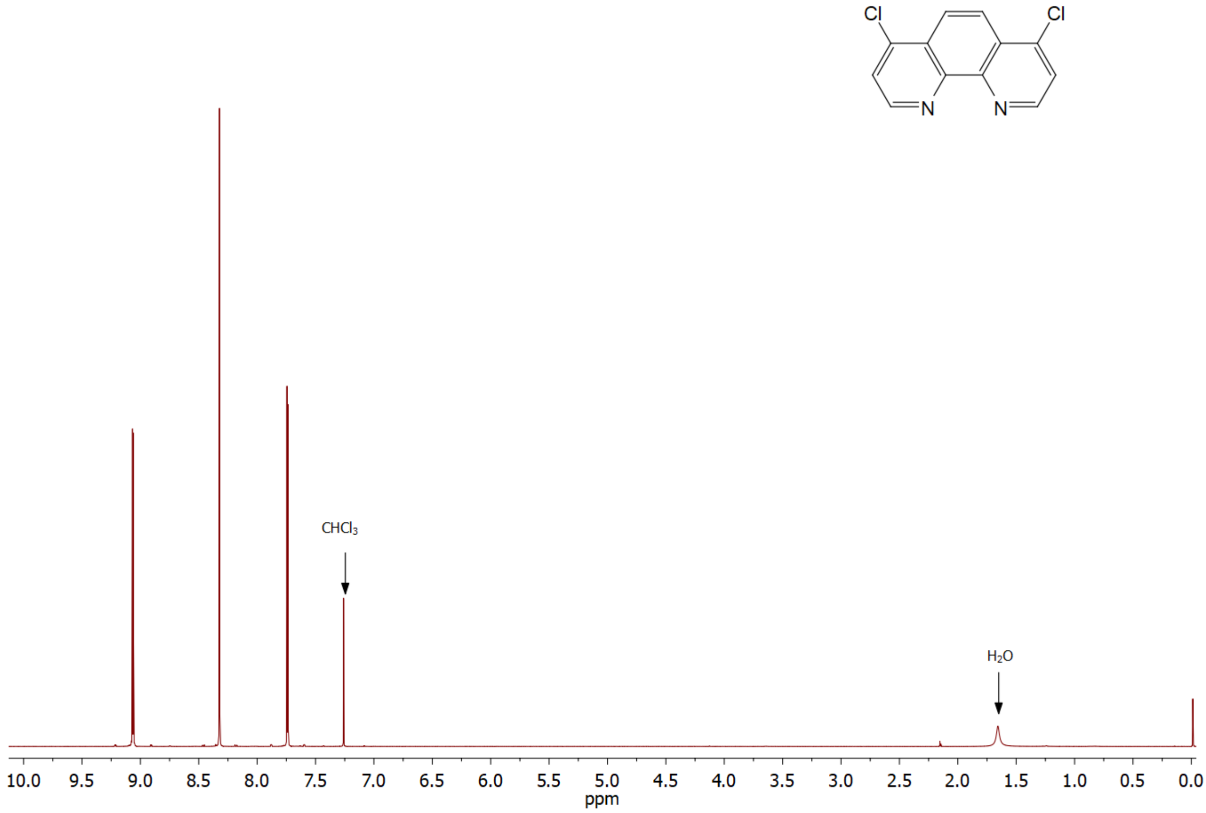

Fig. S1a. ^{1}H NMR ($CDCl_3$; 400.2 MHz) spectrum of **4a**.

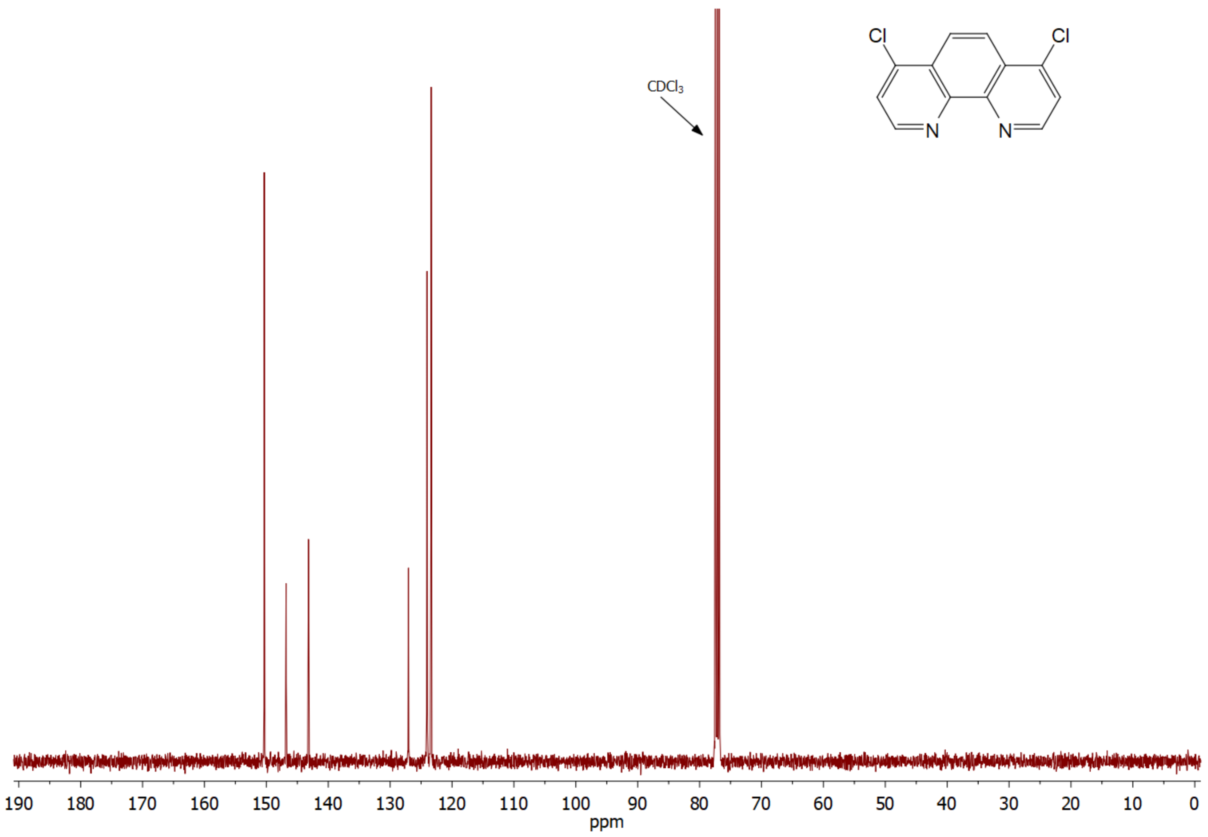

Fig. S1b. $^{13}C\{^{1}H\}$ NMR ($CDCl_3$; 100.5 MHz) spectrum of **4a**.

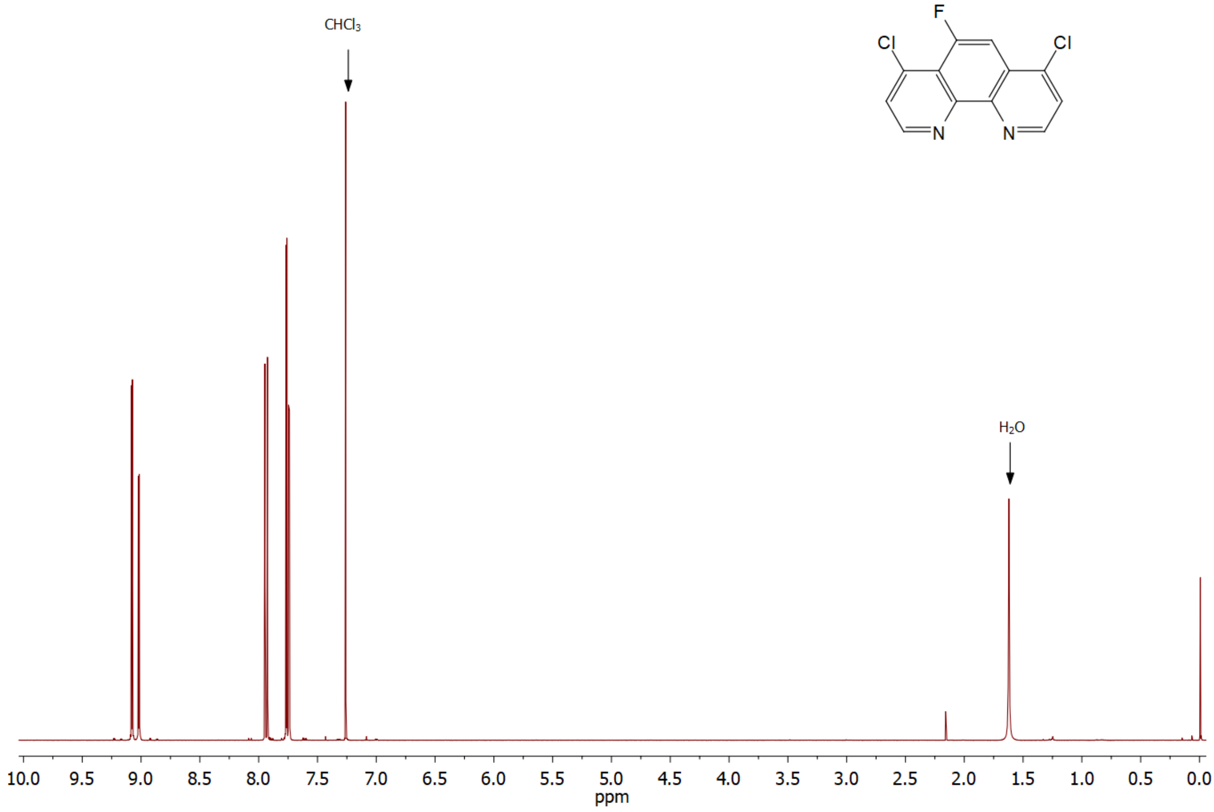

Fig. S2a. ¹H NMR ($CDCl_3$; 600.2 MHz) spectrum of **4b**.

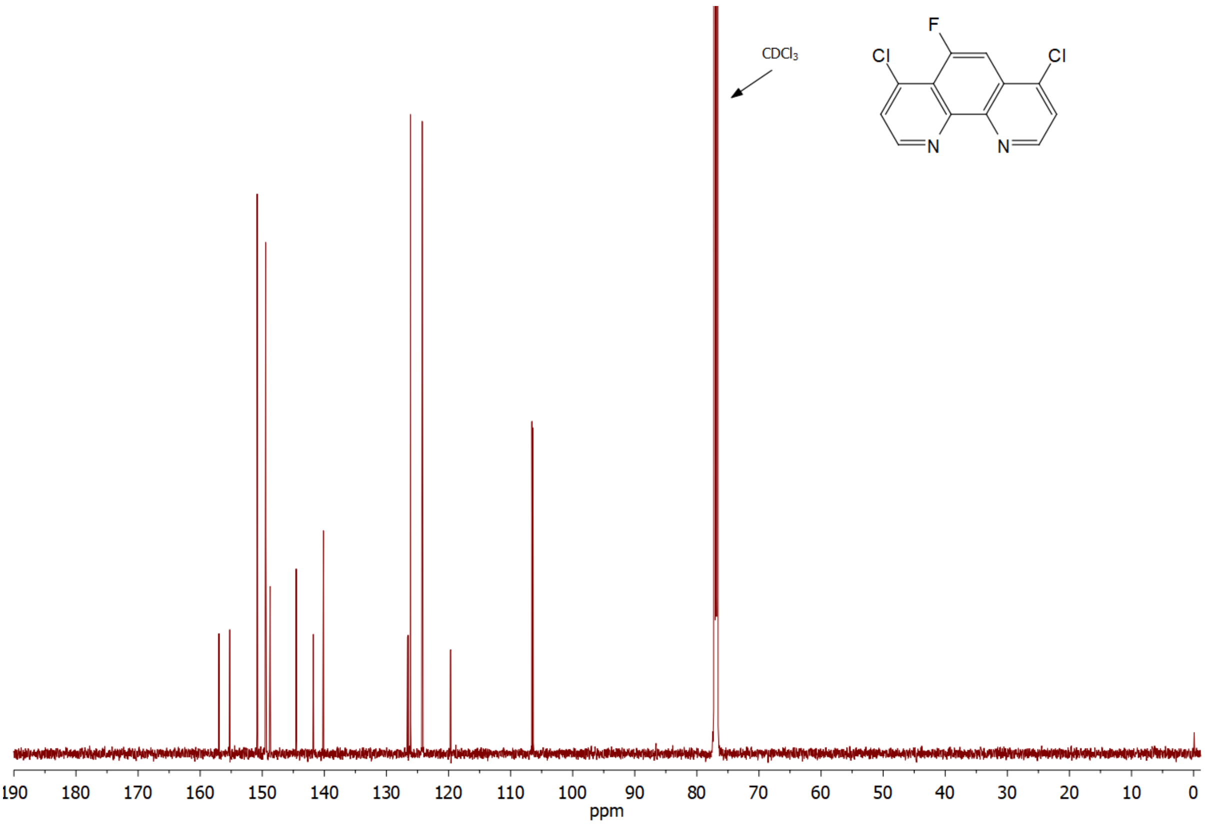

Fig. S2b. ¹³C{¹H} NMR ($CDCl_3$; 150.0 MHz) spectrum of **4b**.

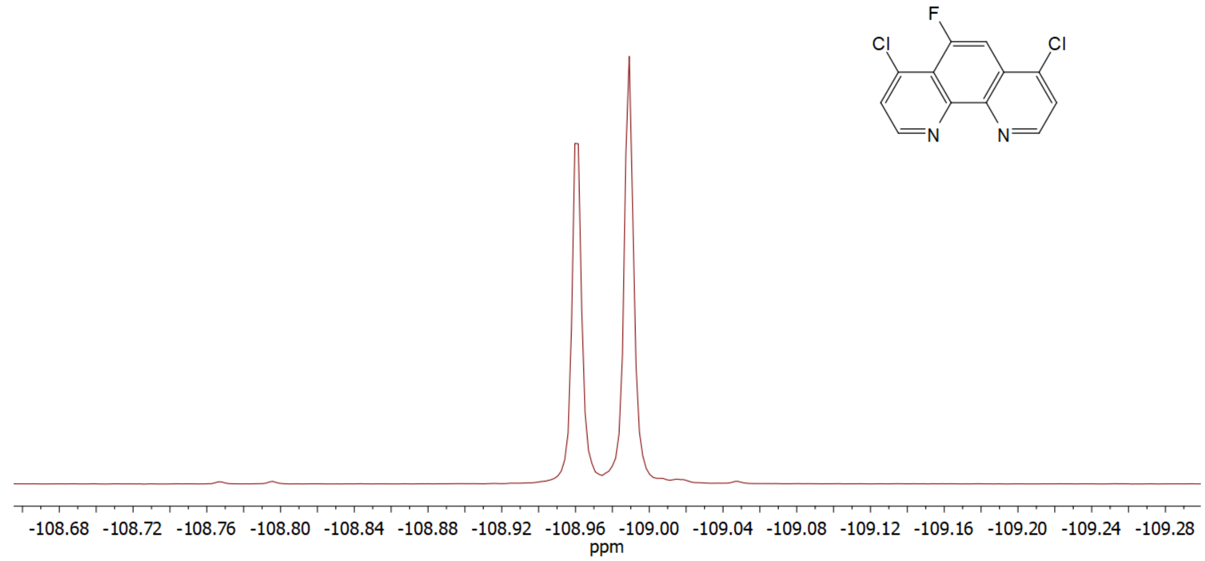

Fig. S2c. ¹⁹F NMR ($CDCl_3$; 470.5 MHz) spectrum of **4b**.

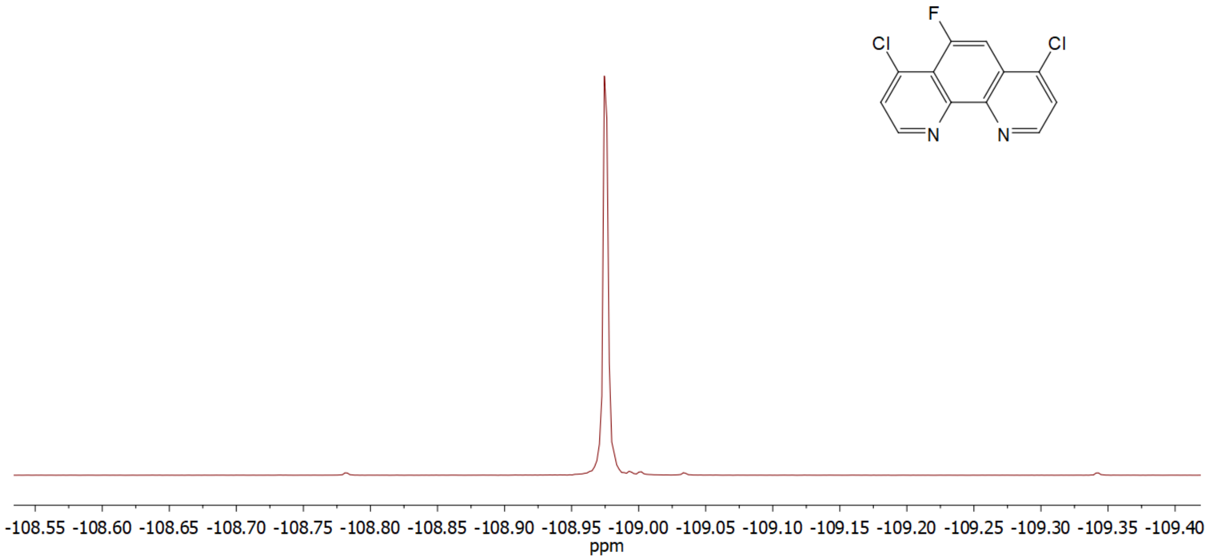

Fig. S2d. $^{19}F\{^{1}H\}$ NMR ($CDCl_3$; 470.5 MHz) spectrum of **4b**.

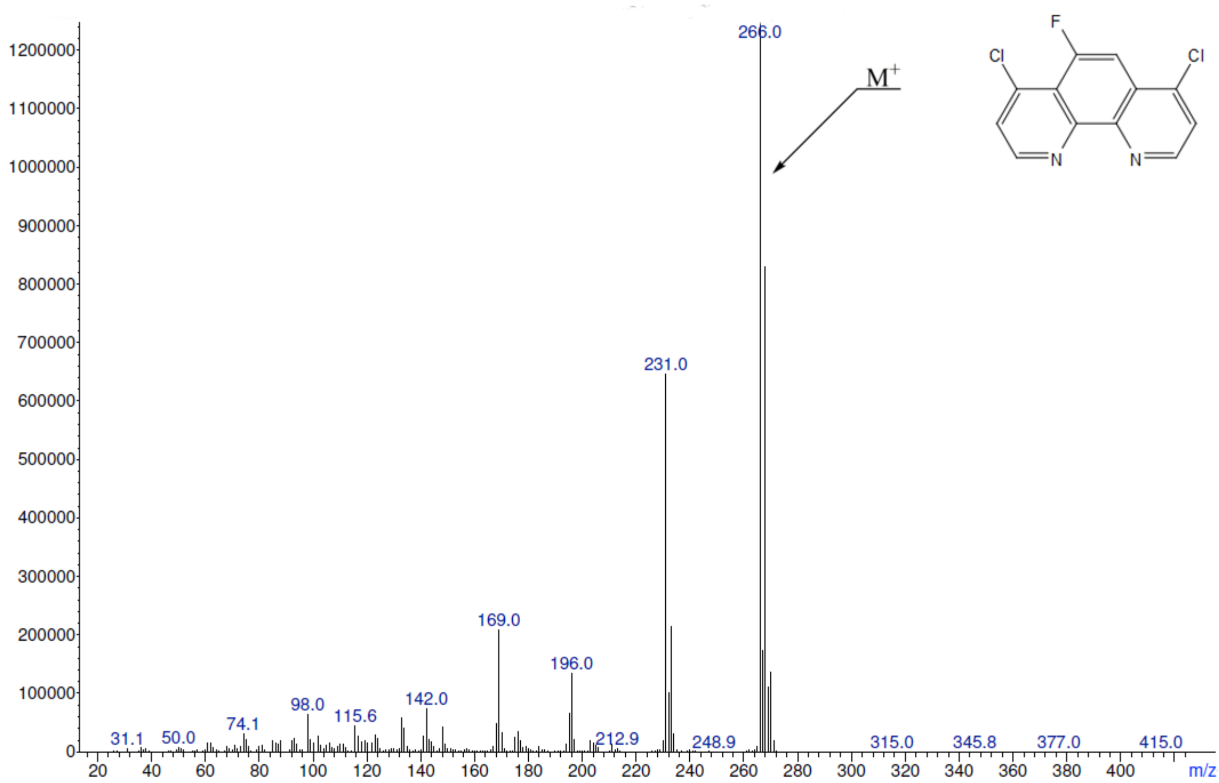

Fig. S2e. MS spectrum of **4b**.

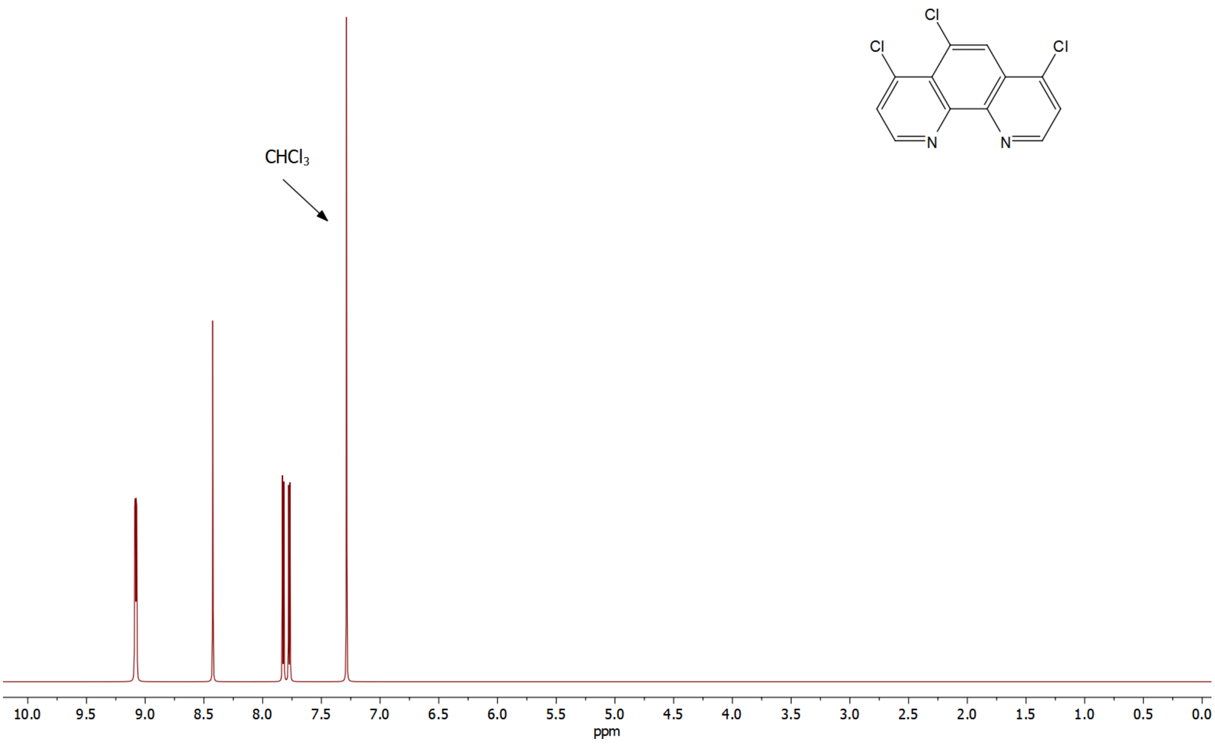

Fig. S3a. ^{1}H NMR ($CDCl_3$; 400.2 MHz) spectrum of **4c**.

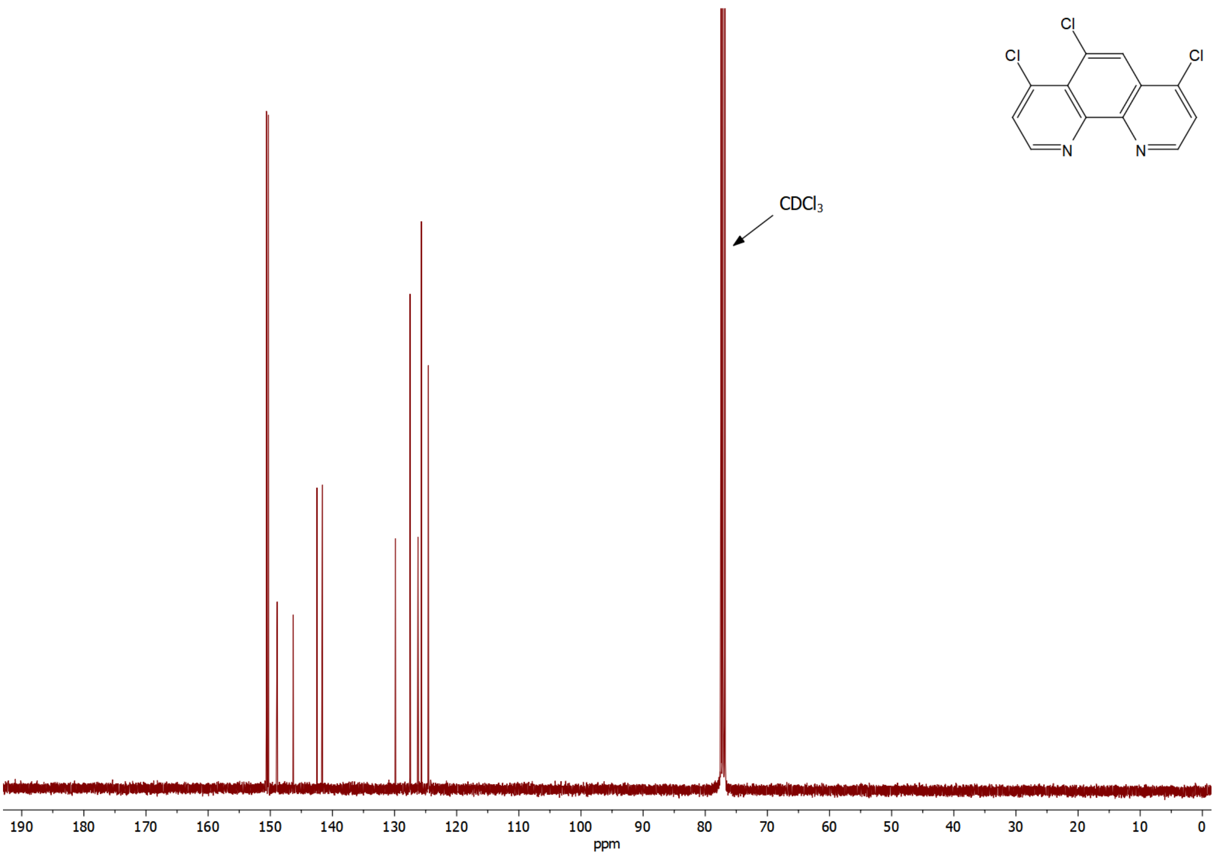

Fig. S3b. $^{13}C\{^1H\}$ NMR ($CDCl_3$; 100.5 MHz) spectrum of **4c**.

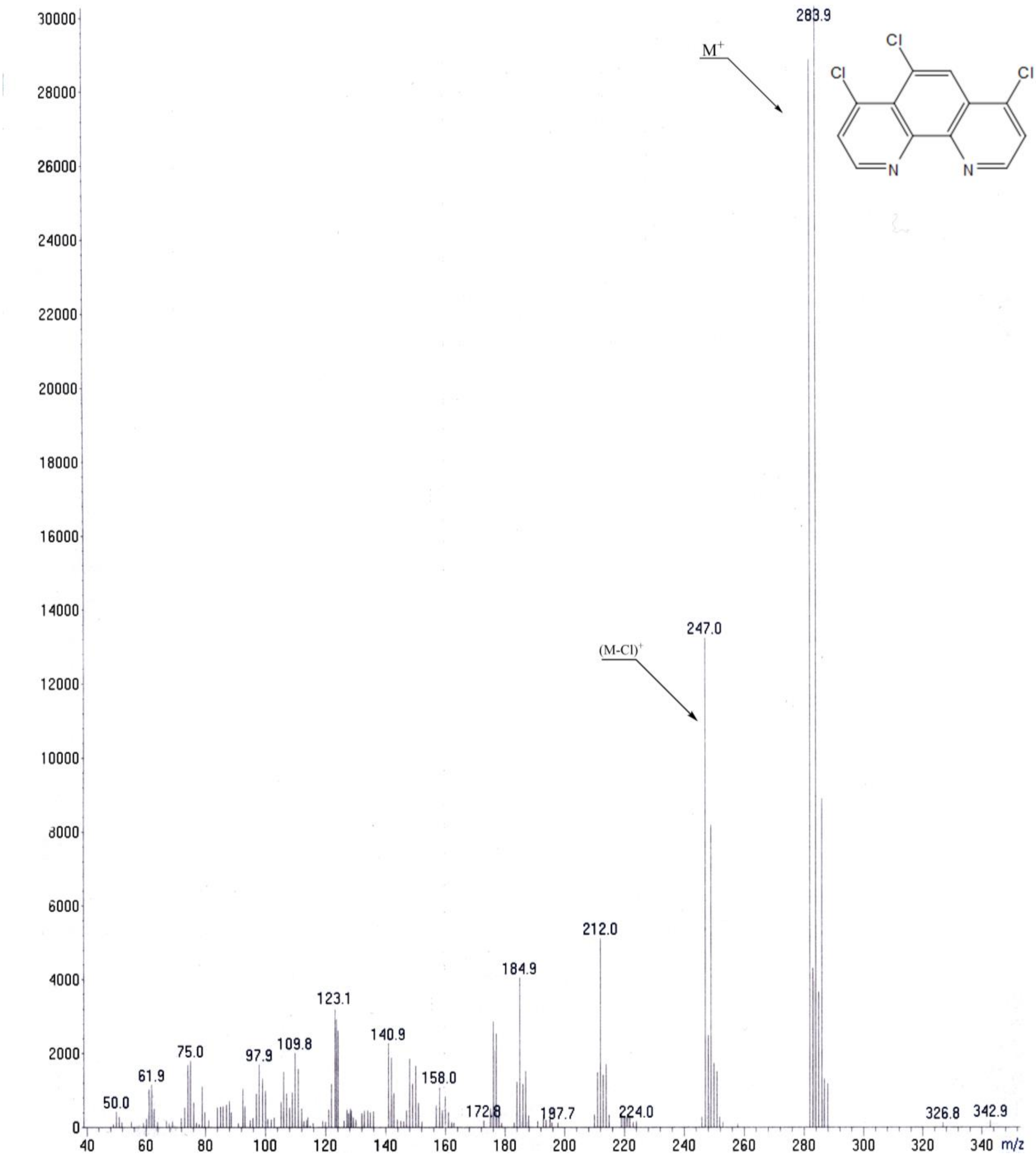

Fig. S3c. MS spectrum of **4c**.

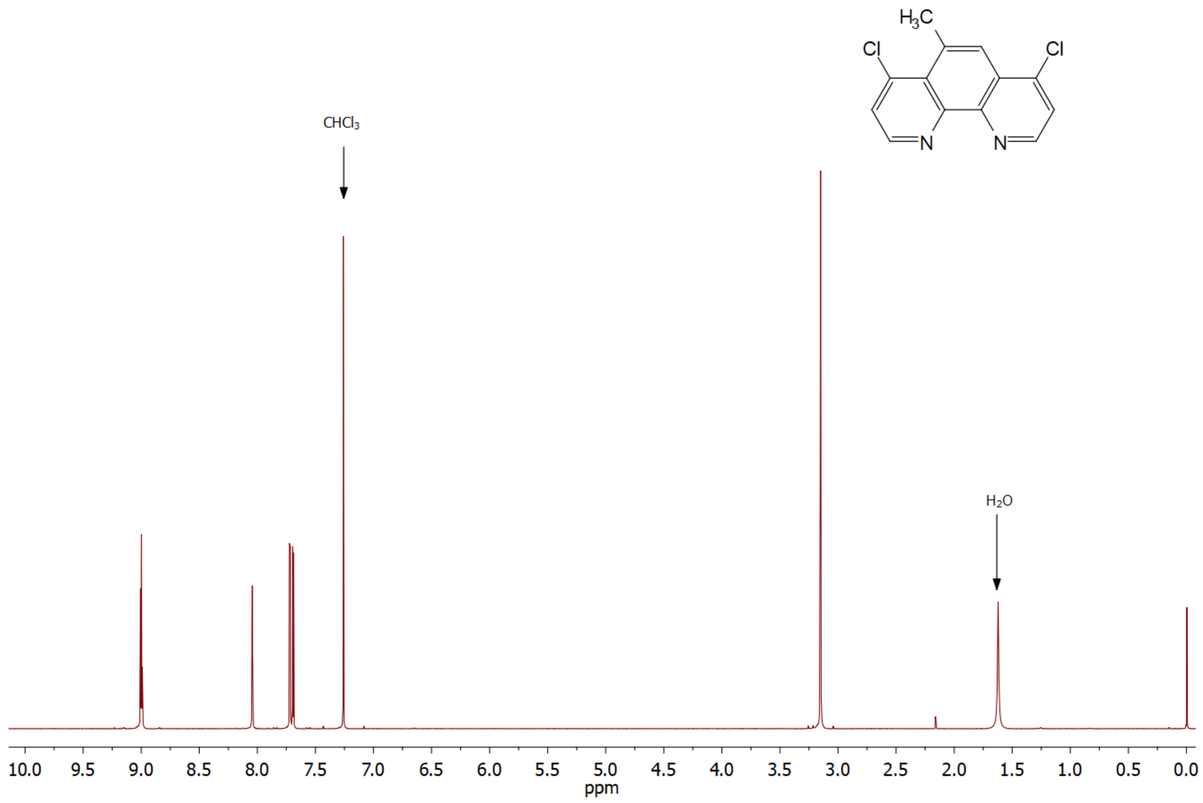

Fig. S4a. ^{1}H NMR ($CDCl_3$; 600.1 MHz) spectrum of **4d**.

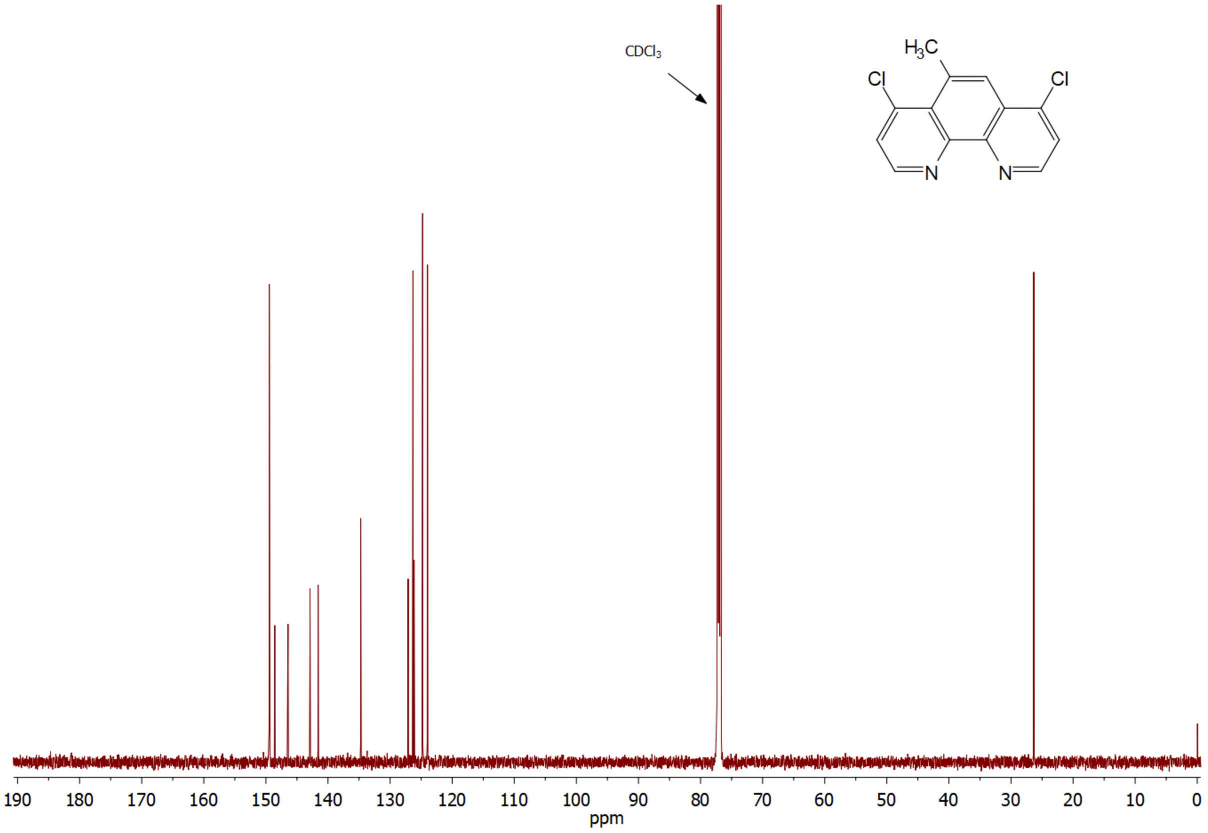

Fig. S4b. ^{13}C{^{1}H} NMR ($CDCl_3$; 150.0 MHz) spectrum of **4d**.

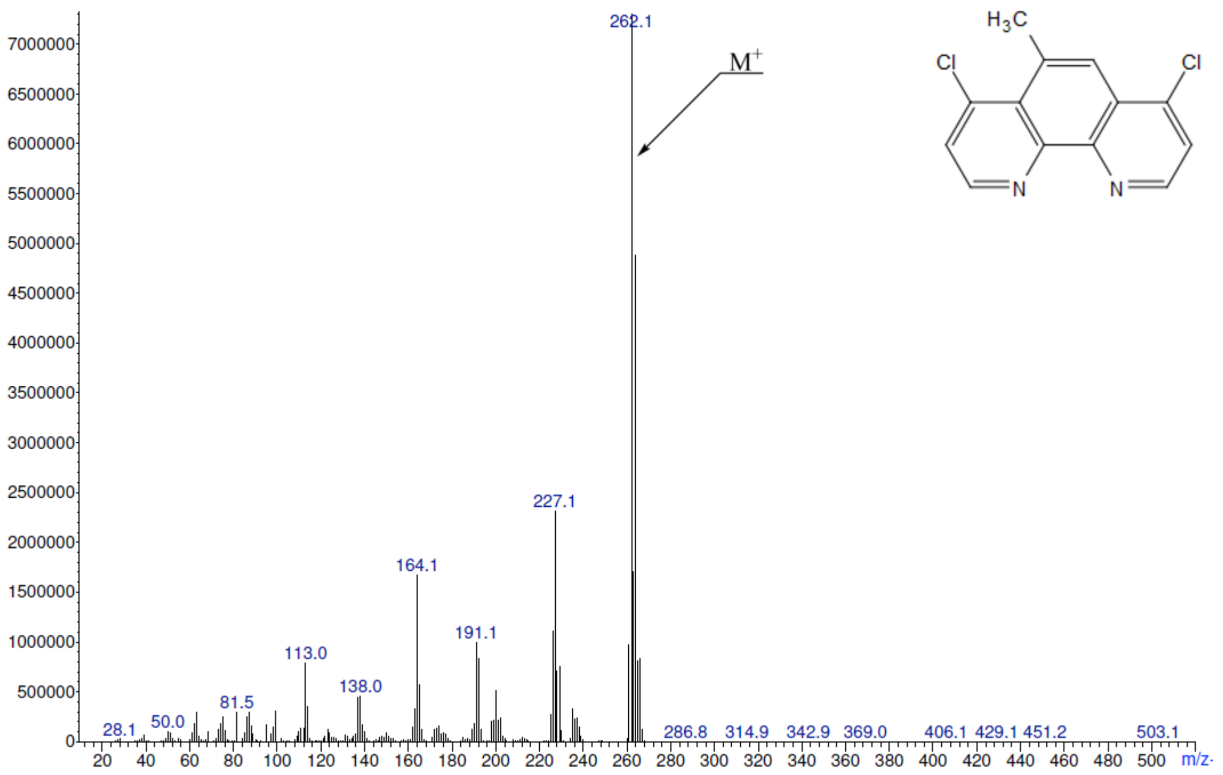

Fig. S4c. MS spectrum of **4d**.

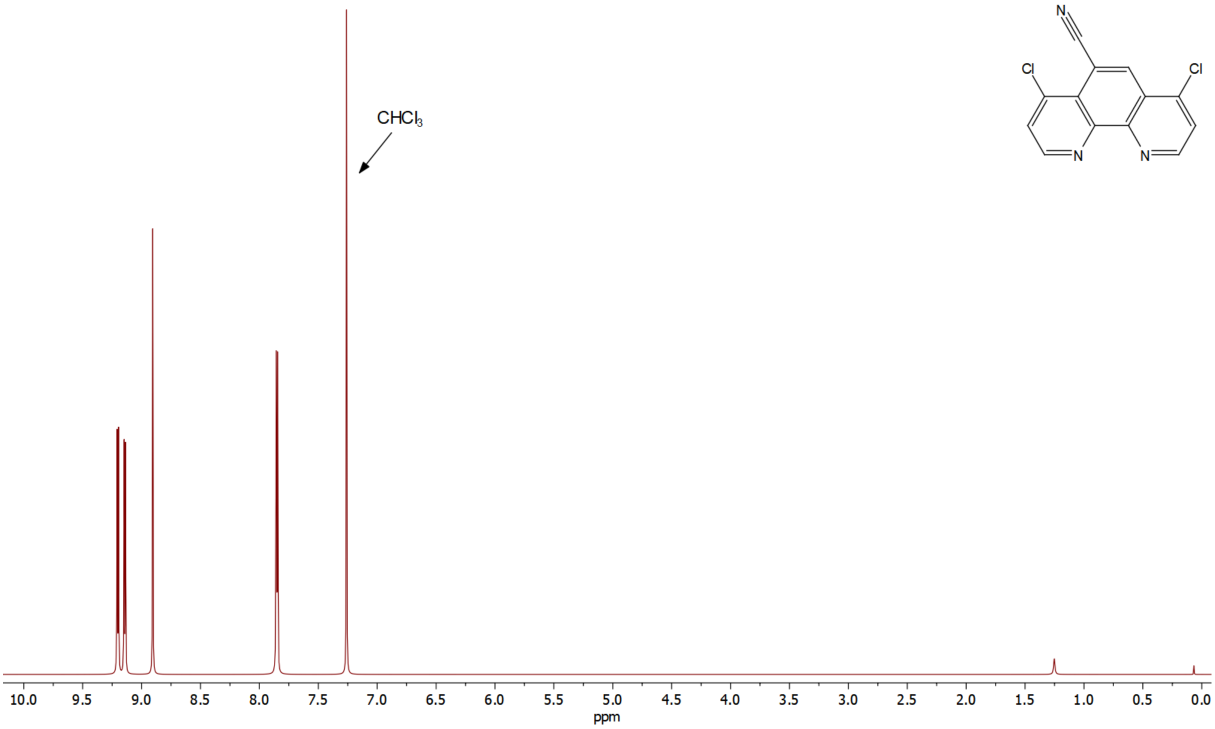

Fig. S5a. ^{1}H NMR ($CDCl_3$; 400.2 MHz) spectrum of **4e**

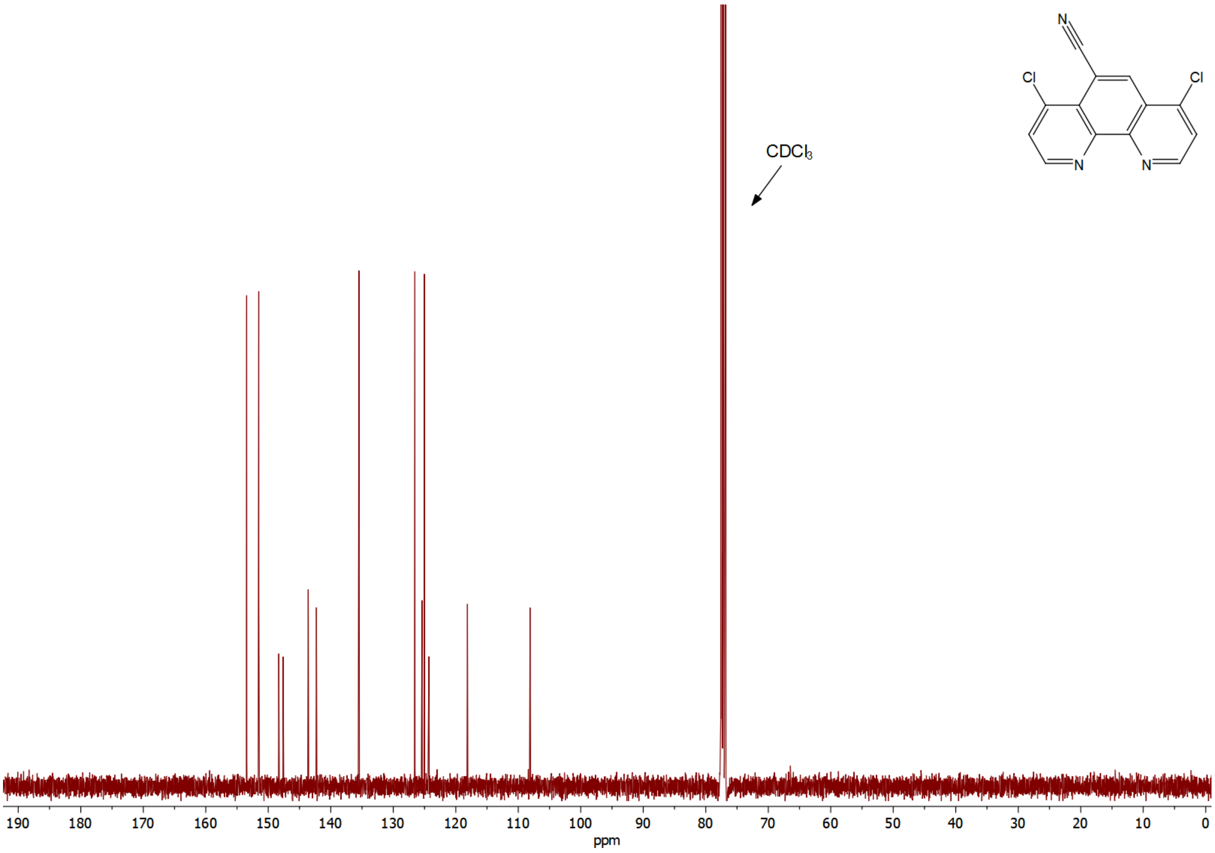

Fig. S5b. ^{13}C{^{1}H} NMR ($CDCl_3$; 100.5 MHz) spectrum of **4e**.

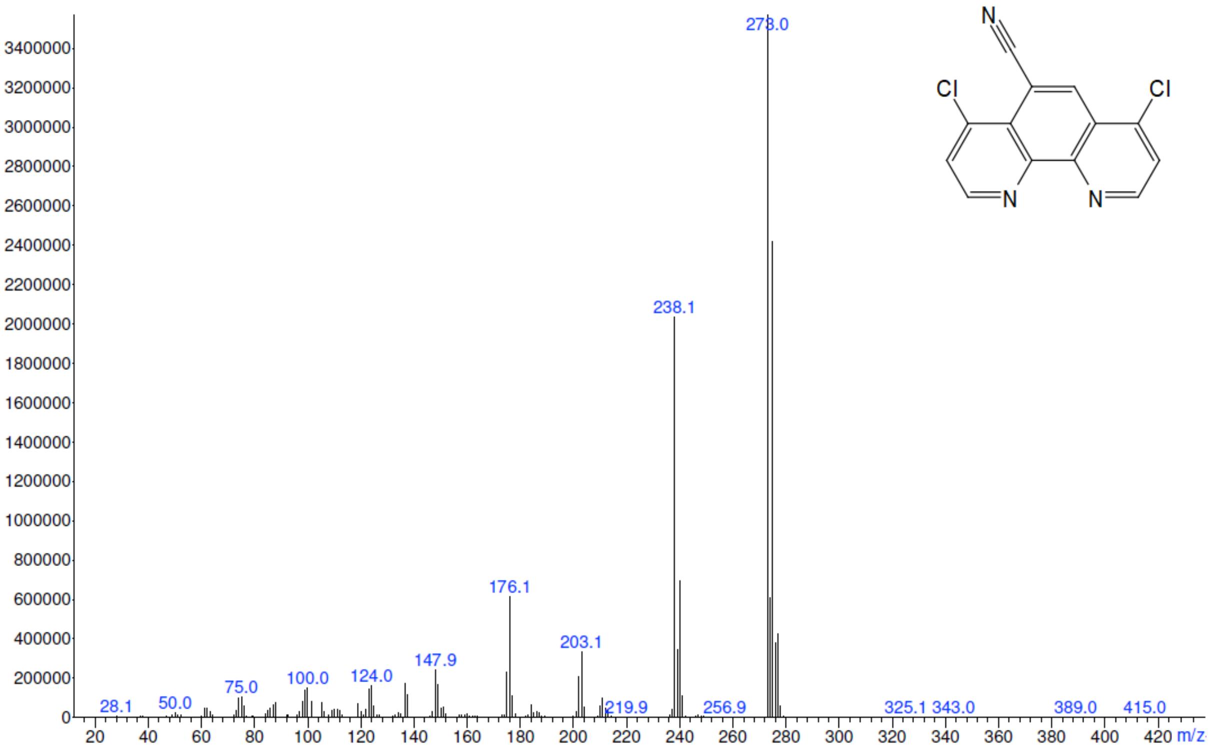

Fig.S5c. MS spectrum of **4e**

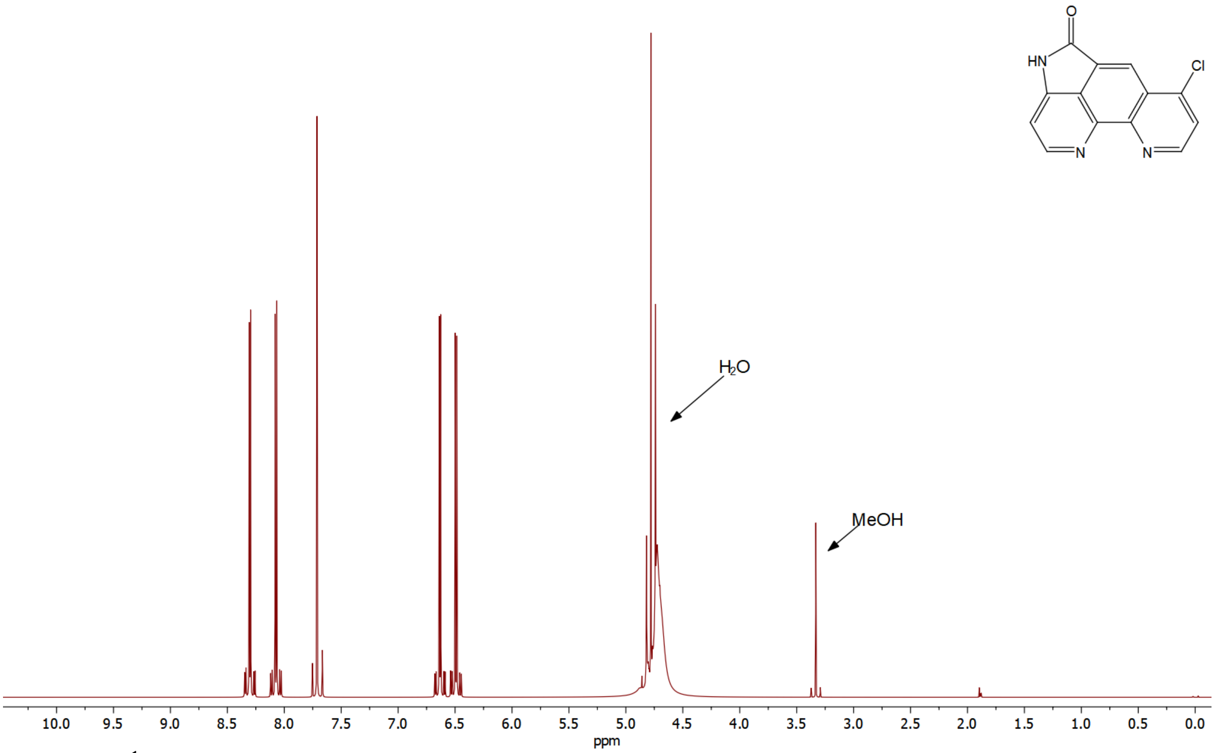

Fig. S6a. ^{1}H NMR (D_2O/KOD; 500.1 MHz) spectrum of **4f**.

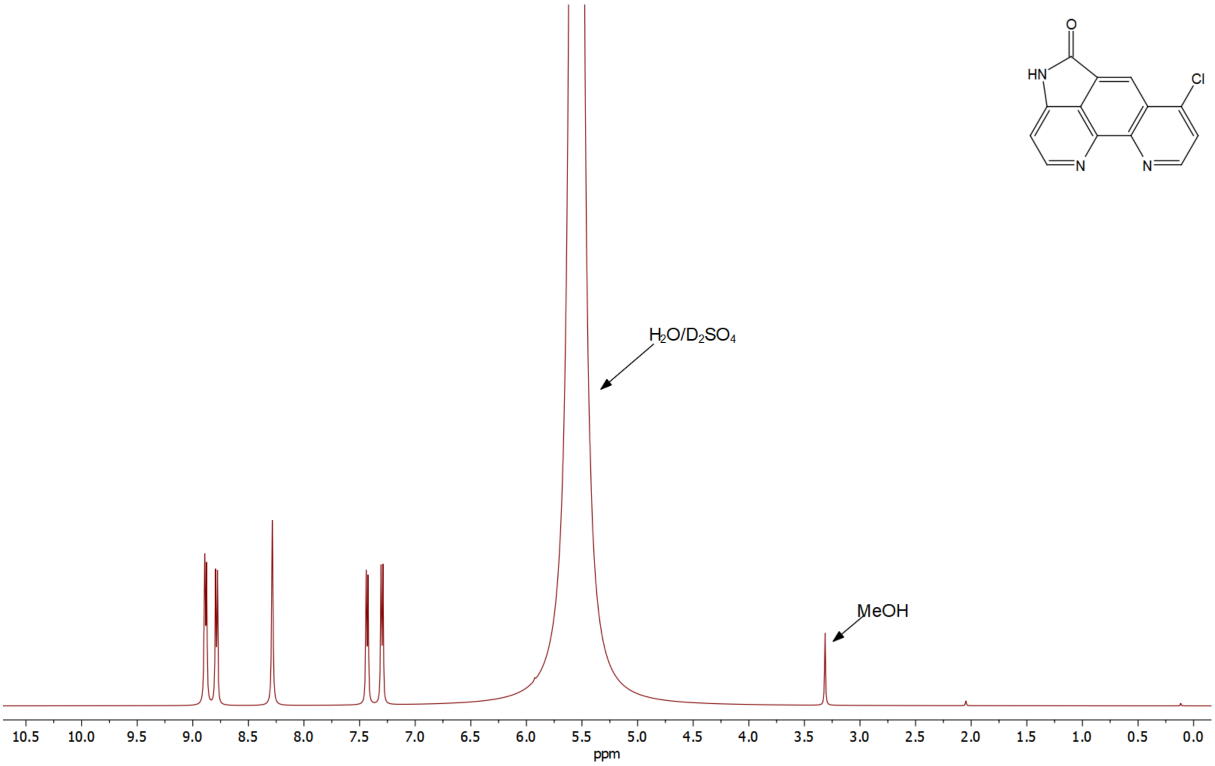

Fig. S6b. ^{1}H NMR (D_2O/D_2SO_4; 400.1 MHz) spectrum of **4f**.

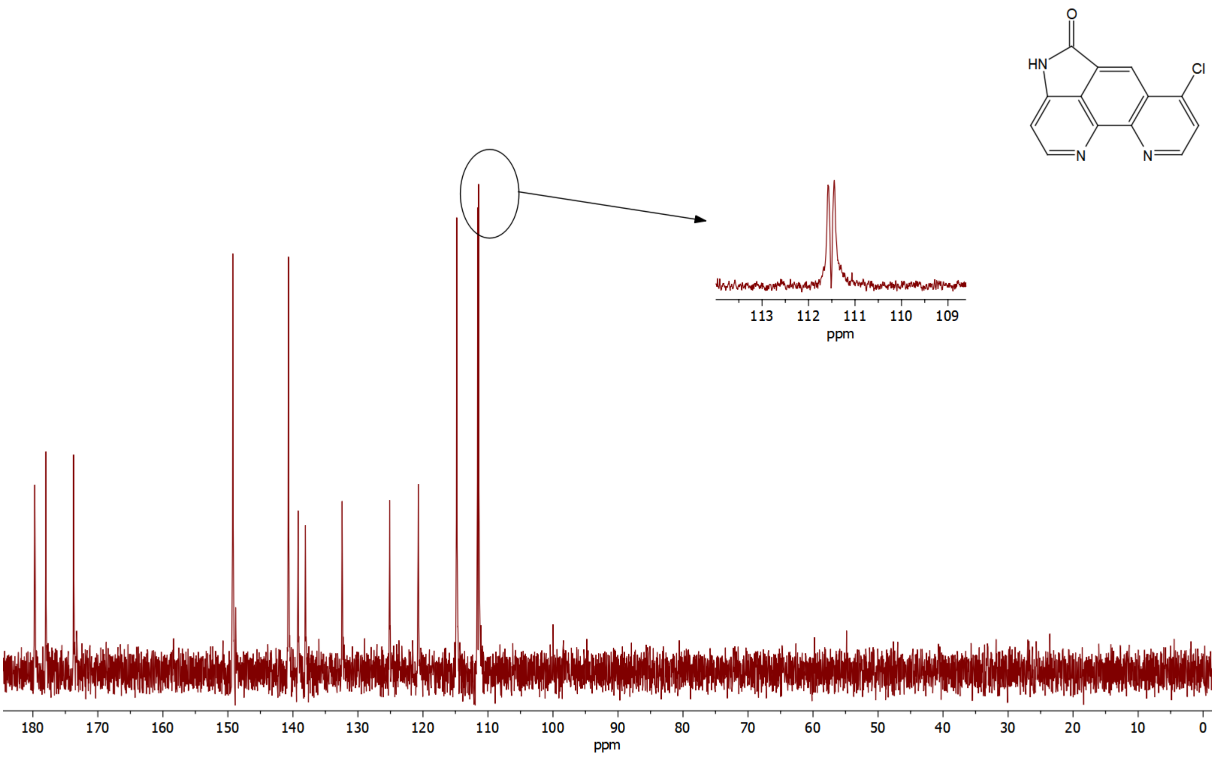

Fig. S6c. $^{13}C\{^{1}H\}$ NMR (D_2O/KOD; 125.5 MHz) spectrum of **4f**.

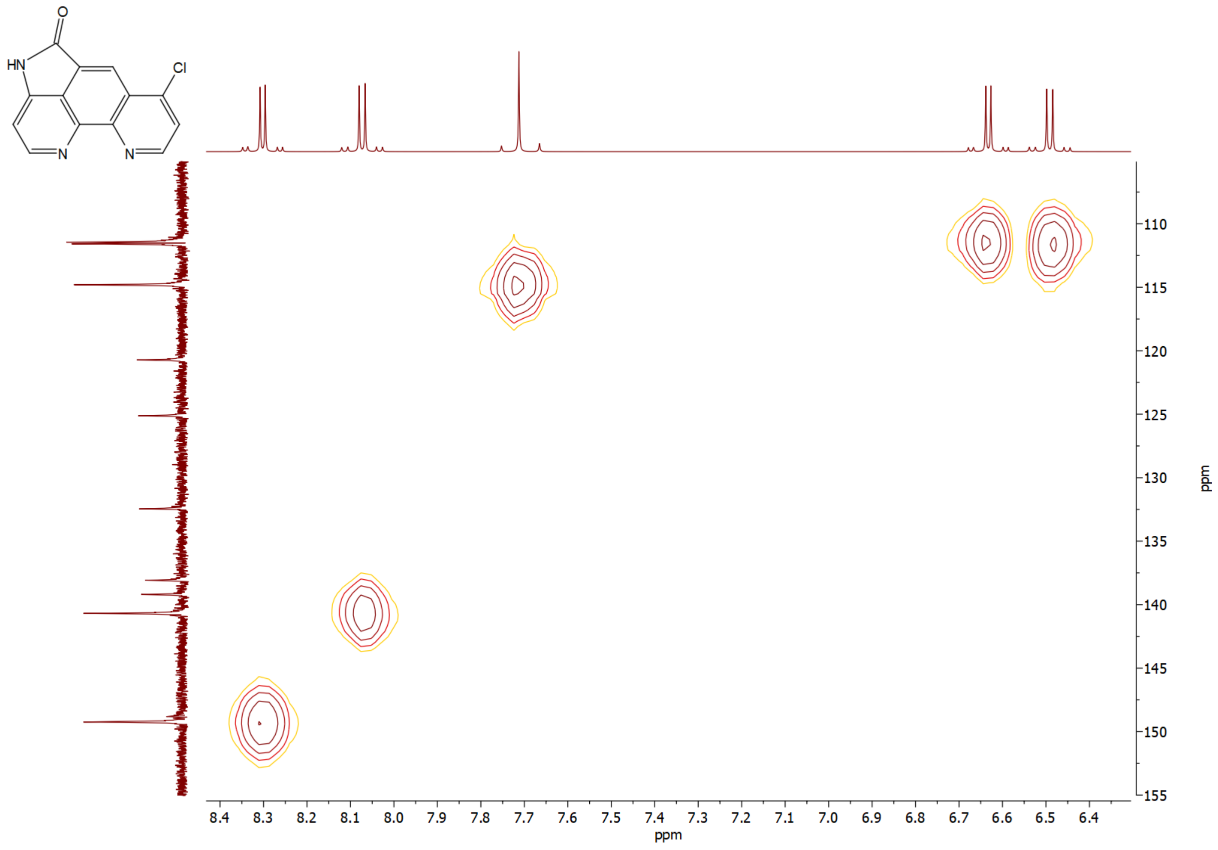

Fig. S6d. ^{1}H, ^{13}C NMR HMQC in D_2O spectrum of **4f**.

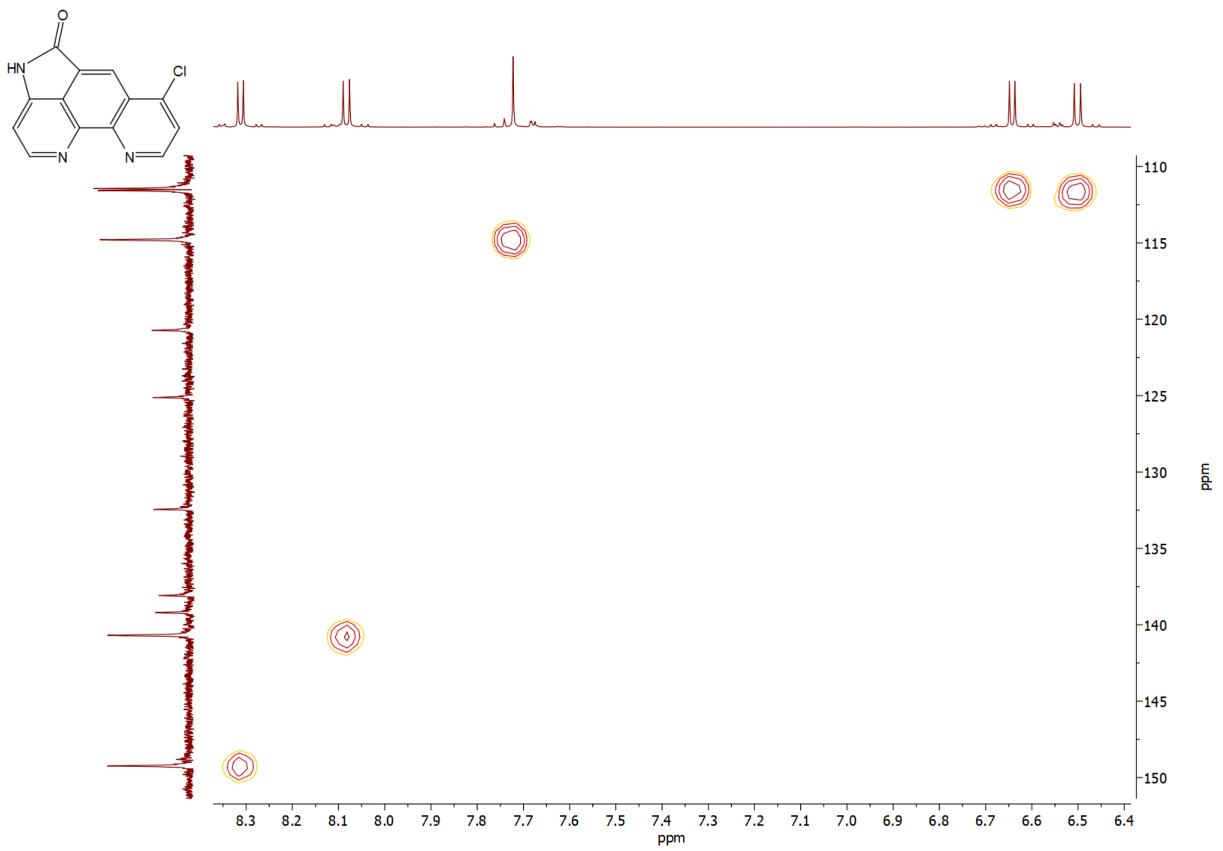

Fig. S6e. ^{1}H, ^{13}C NMR HSQC in D_2O spectrum of **4f**.

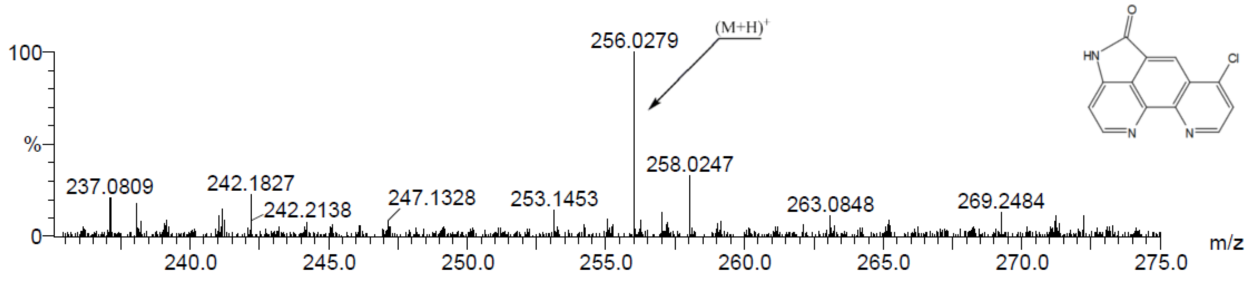

Fig.S6e. MS spectrum of **4f.**

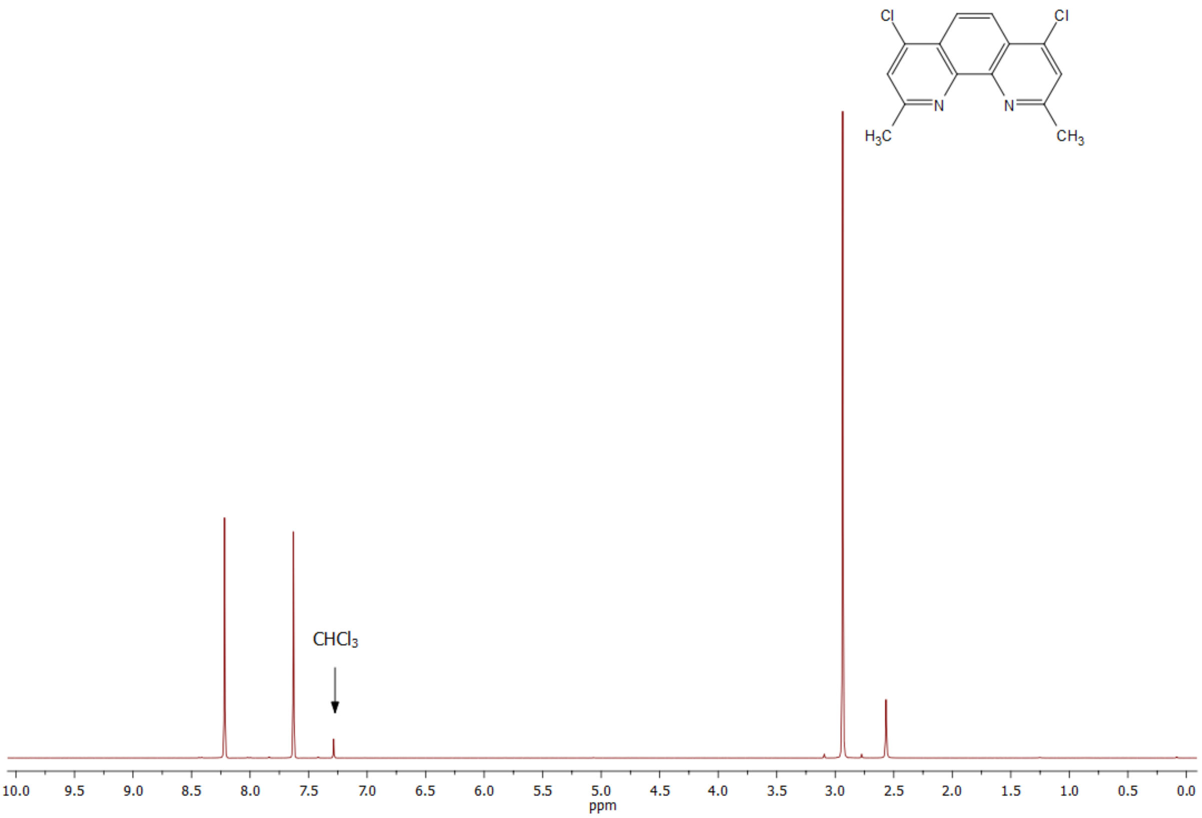

Fig. S7a. ^{1}H NMR ($CDCl_3$; 400.2 MHz) spectrum of **4g**.

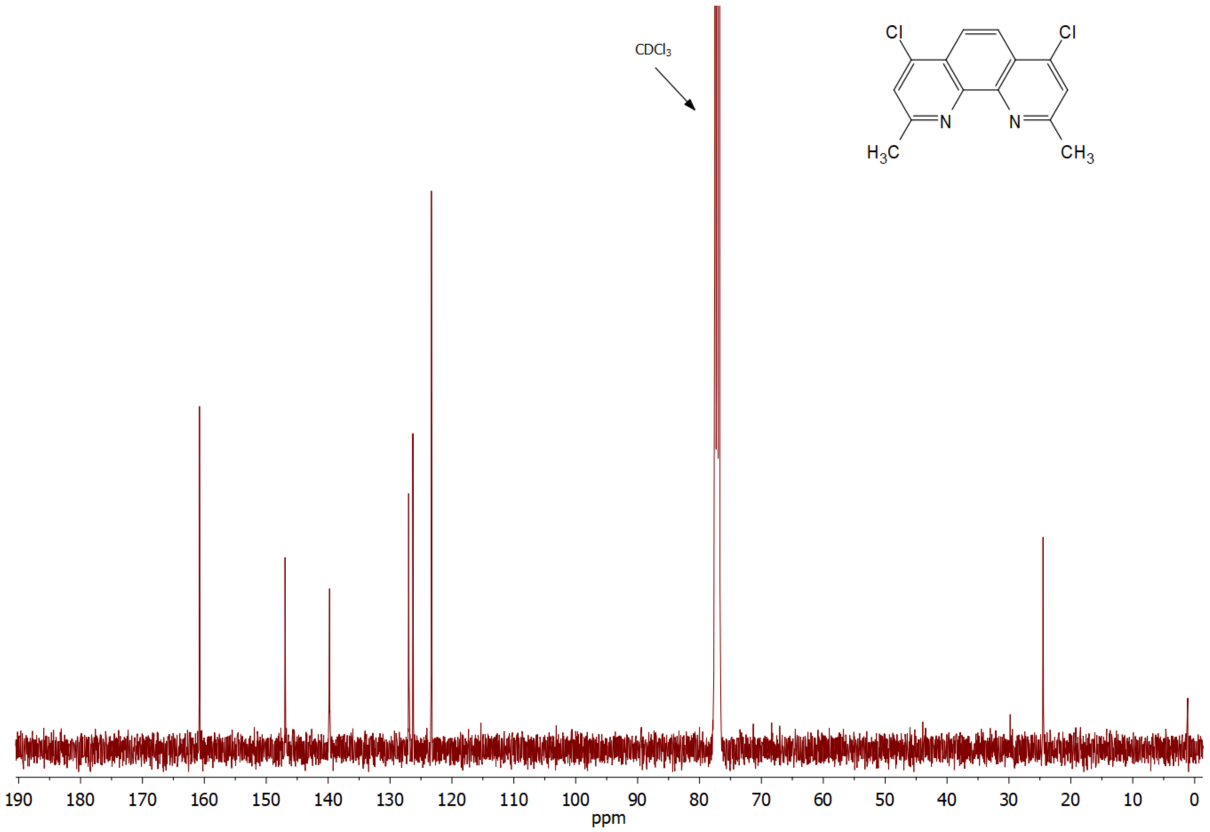

Fig. S7b. $^{13}C\{^1H\}$ NMR ($CDCl_3$; 100.5 MHz) spectrum of **4g**.

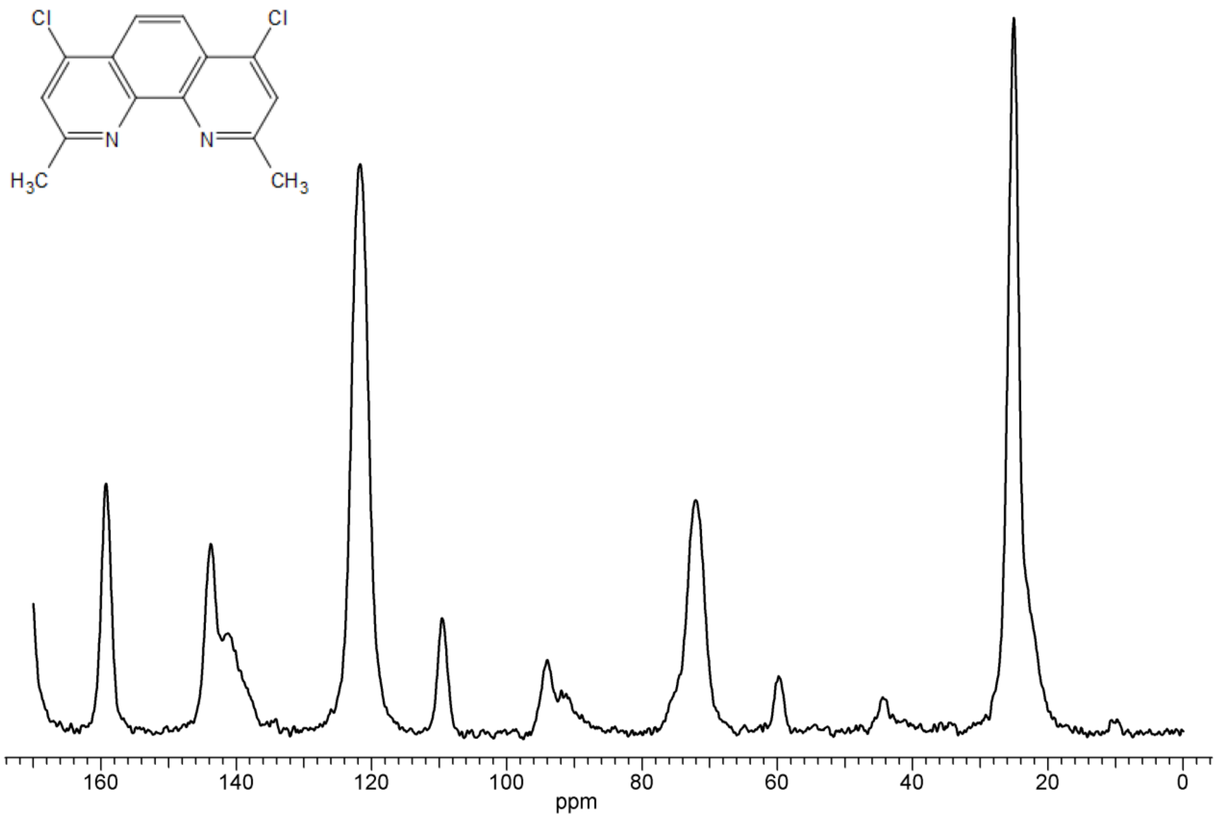

Fig. S7c. ^{13}C CP/MAS NMR spectrum of **4g**.

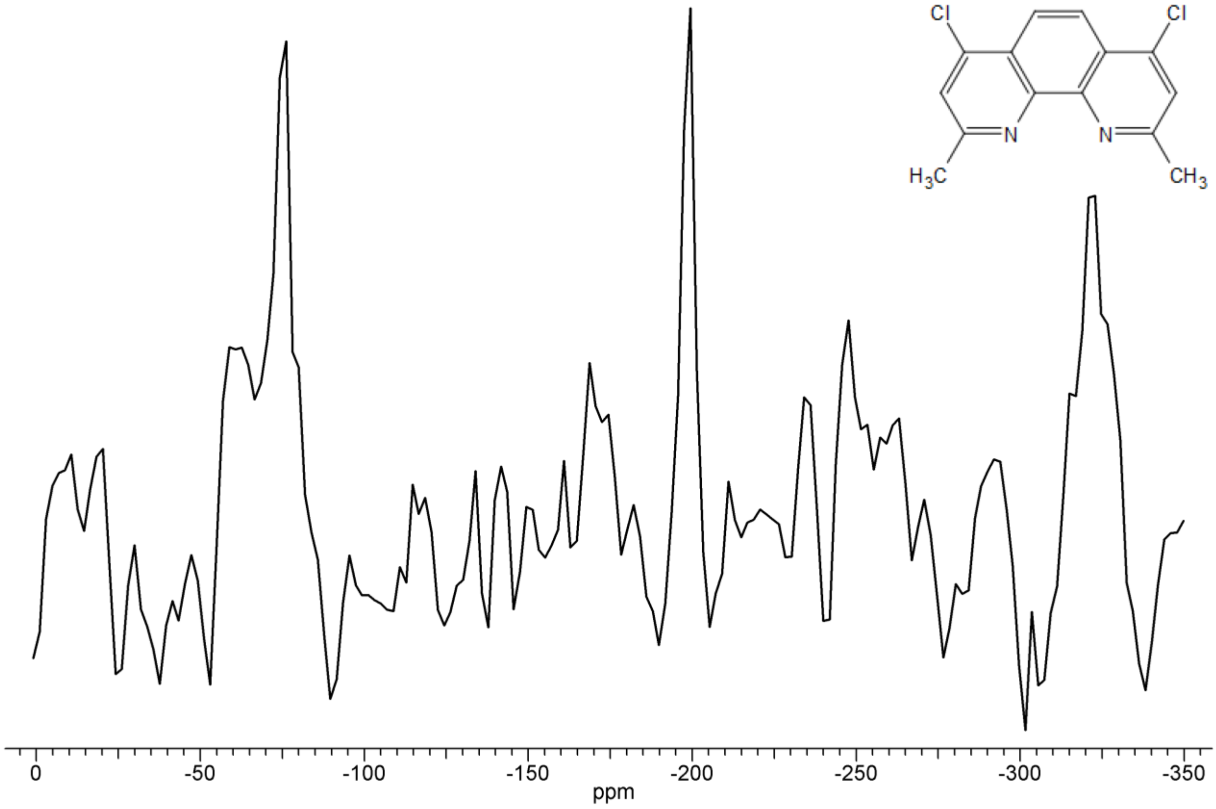

Fig. S7d. ^{15}N CP/MAS NMR spectrum of **4g**.

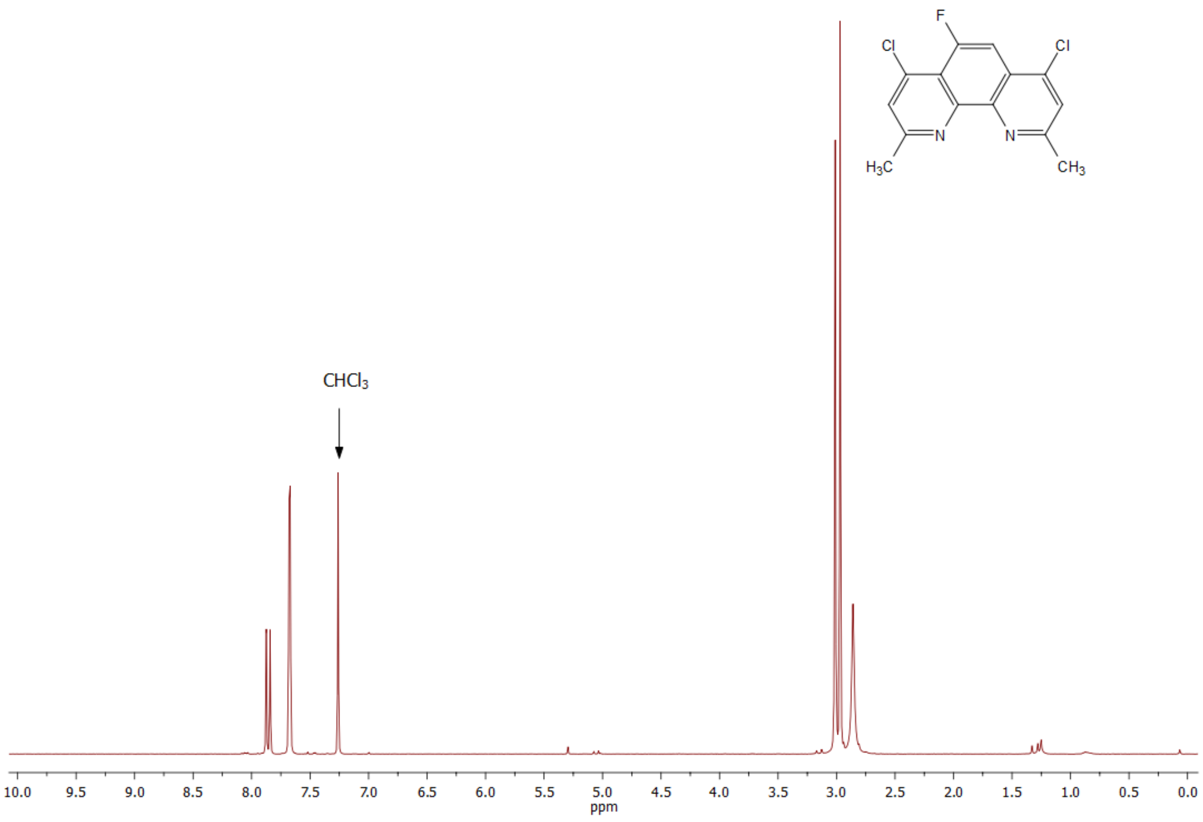

Fig. S8a. ^{1}H NMR ($CDCl_3$; 400.2 MHz) spectrum of **4h**.

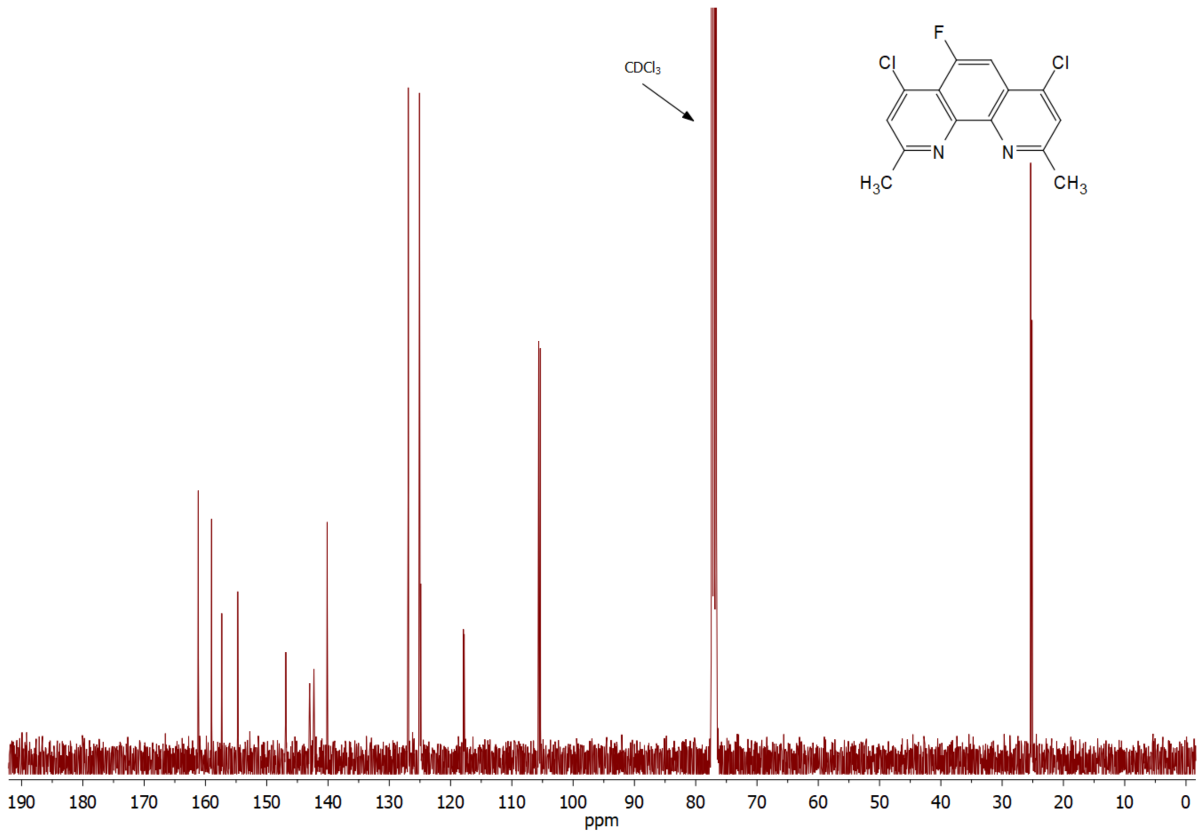

Fig. S8b. $^{13}C\{^{1}H\}$ NMR ($CDCl_3$; 100.5 MHz) spectrum of **4h**.

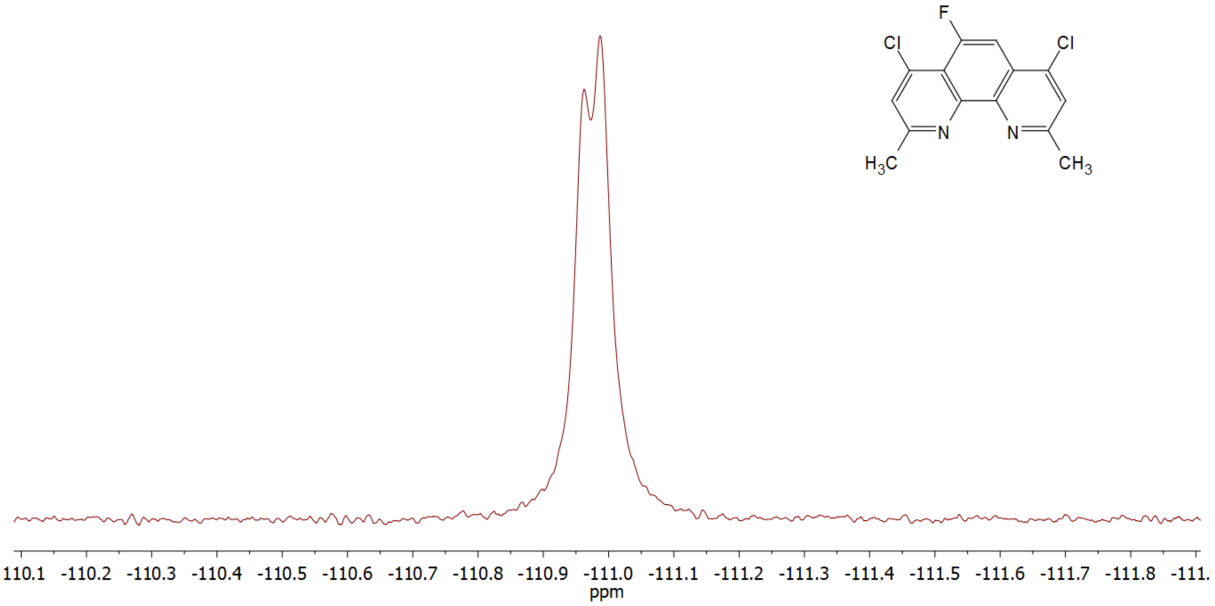

Fig. S8c. ^{19}F NMR ($CDCl_3$; 470.5 MHz) spectrum of **4h**.

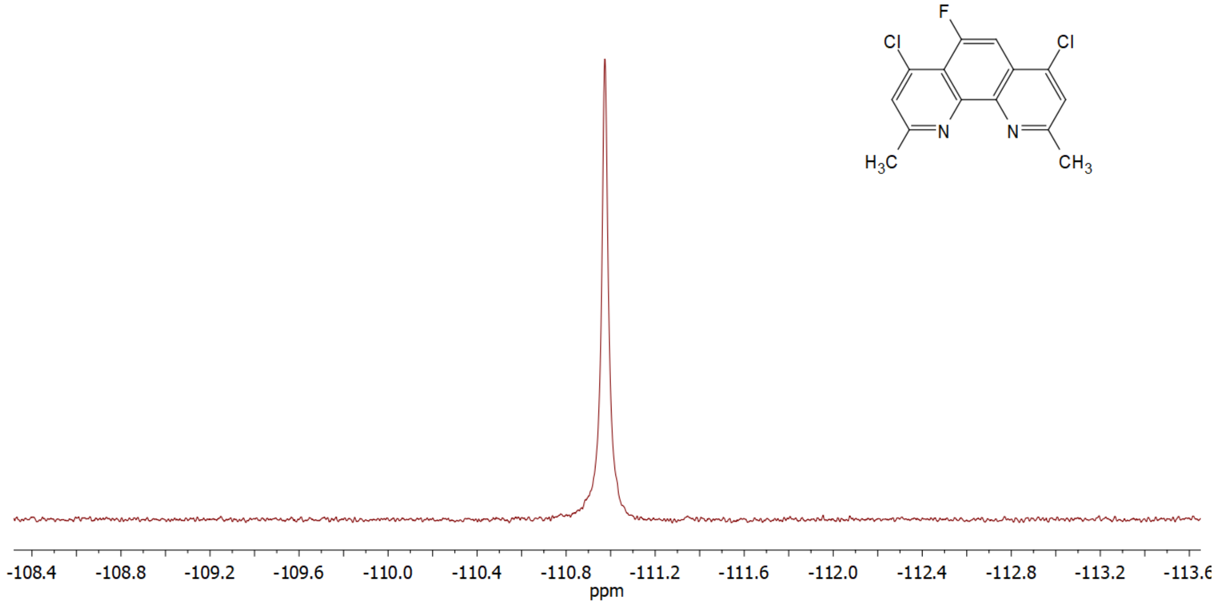

Fig. S8d. ^{19}F{^{1}H} NMR ($CDCl_3$; 470.5 MHz) spectrum of **4h**.

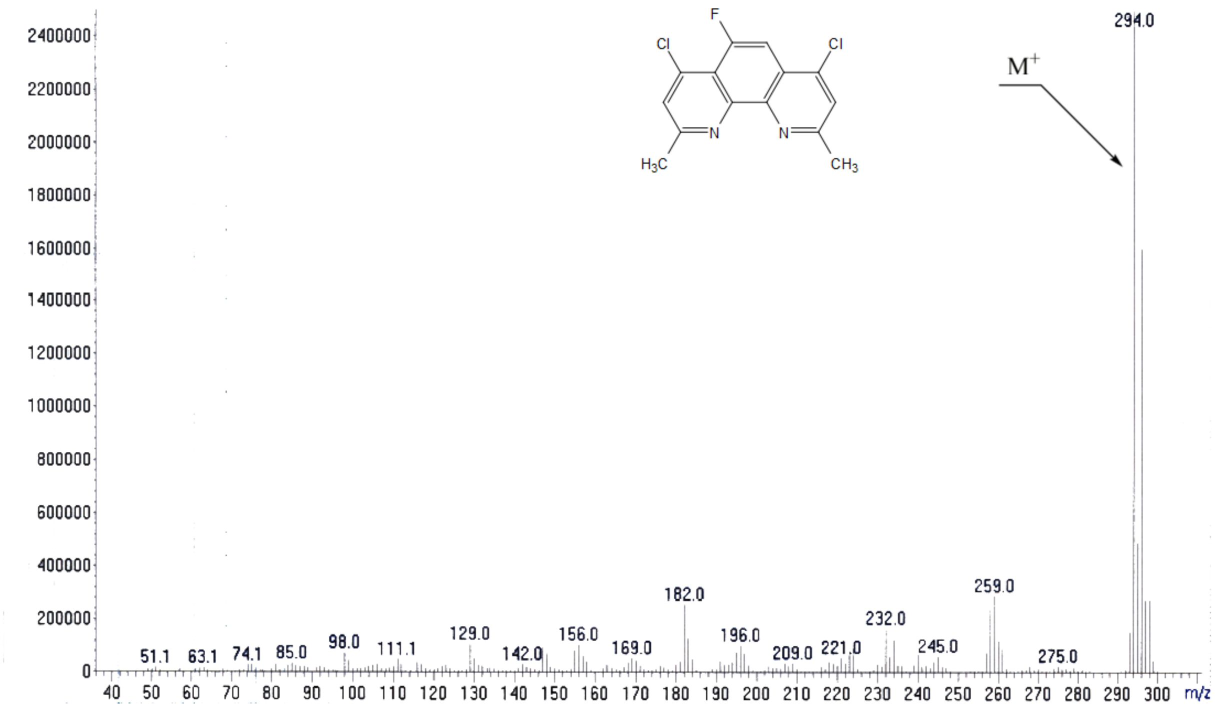

Fig. S8e. MS spectrum of **4h**.

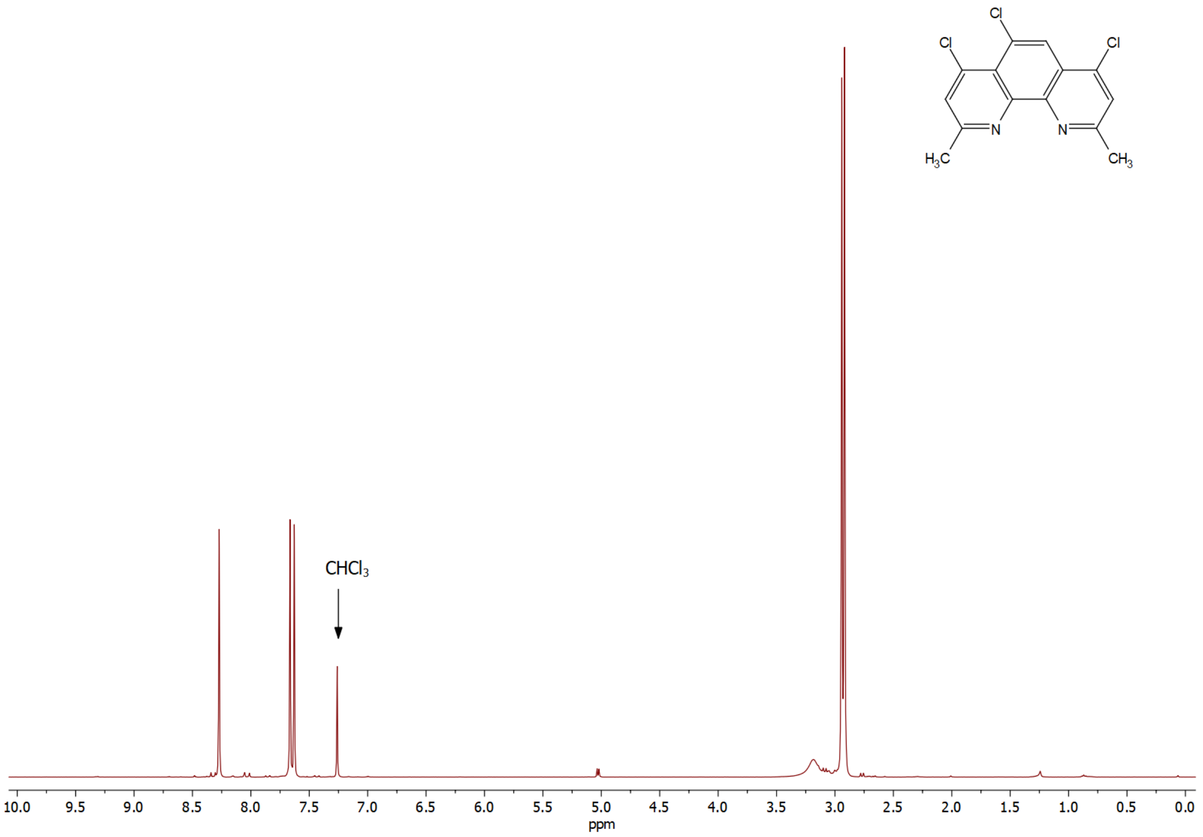

Fig. S9a. ^{1}H NMR ($CDCl_3$; 400.2 MHz) spectrum of **4i**.

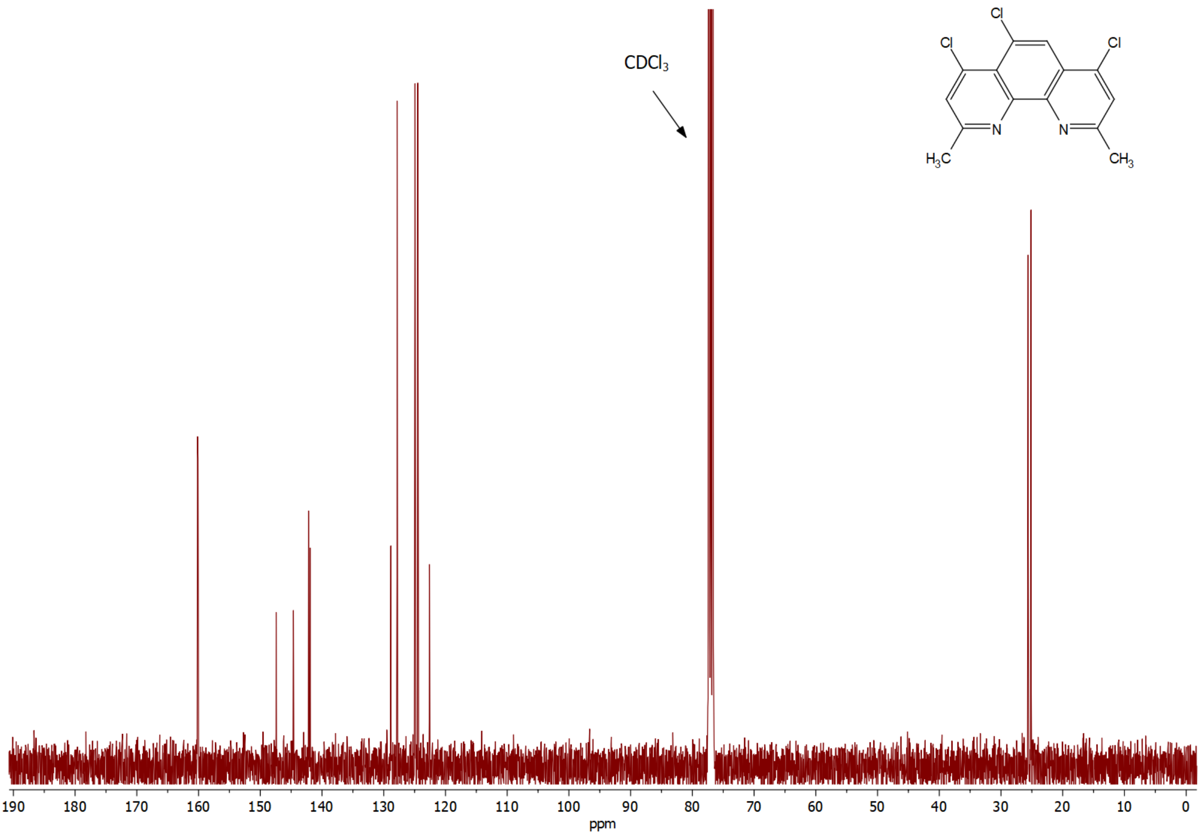

Fig. S9b. ^{13}C{^{1}H} NMR ($CDCl_3$; 100.5 MHz) spectrum of **4i**.

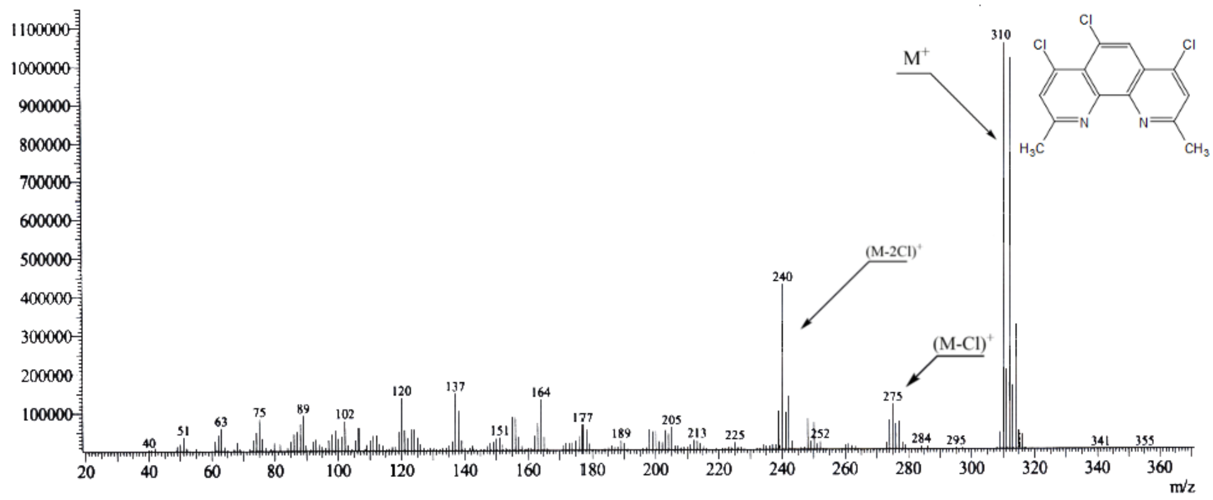

Fig. S9c. MS spectrum of **4i**.

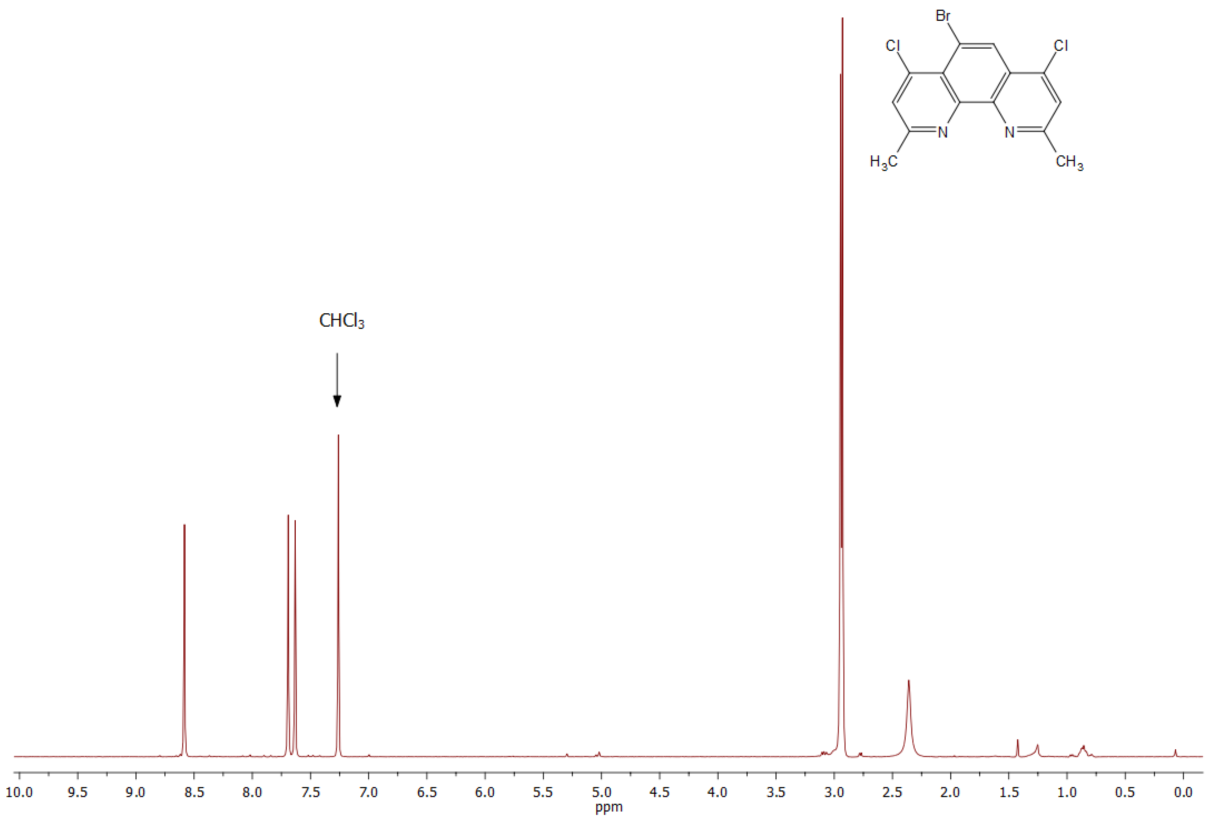

Fig. S10a. ^{1}H NMR ($CDCl_3$; 400.2 MHz) spectrum of **4j**.

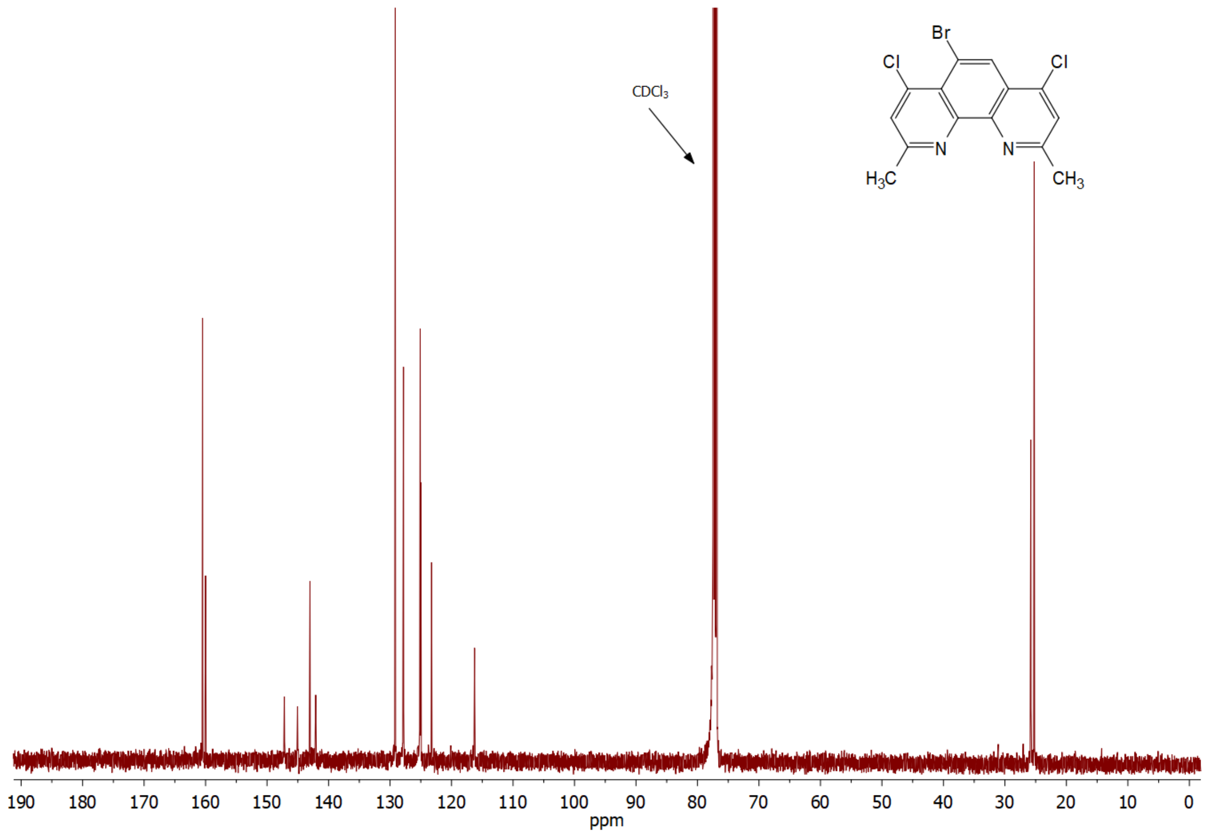

Fig. S10b. $^{13}C\{^1H\}$ NMR ($CDCl_3$; 100.5 MHz) spectrum of **4j**.

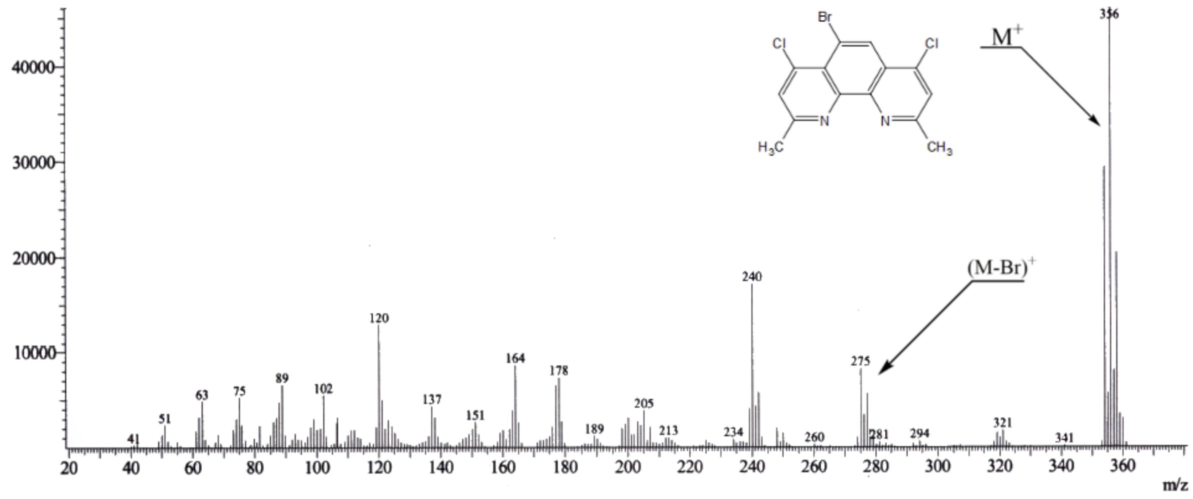

Fig. S10c. MS spectrum of **4j**.

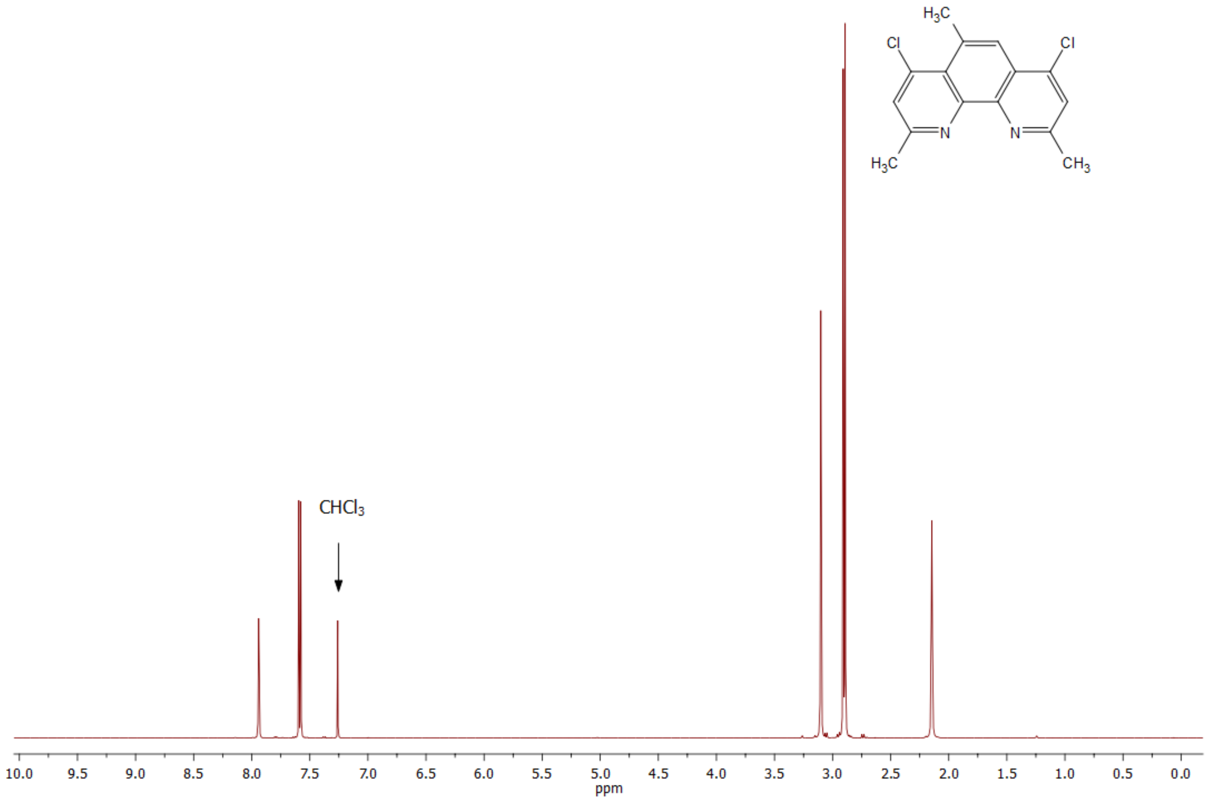

Fig. S11a. ^{1}H NMR ($CDCl_3$; 400.2 MHz) spectrum of **4k**.

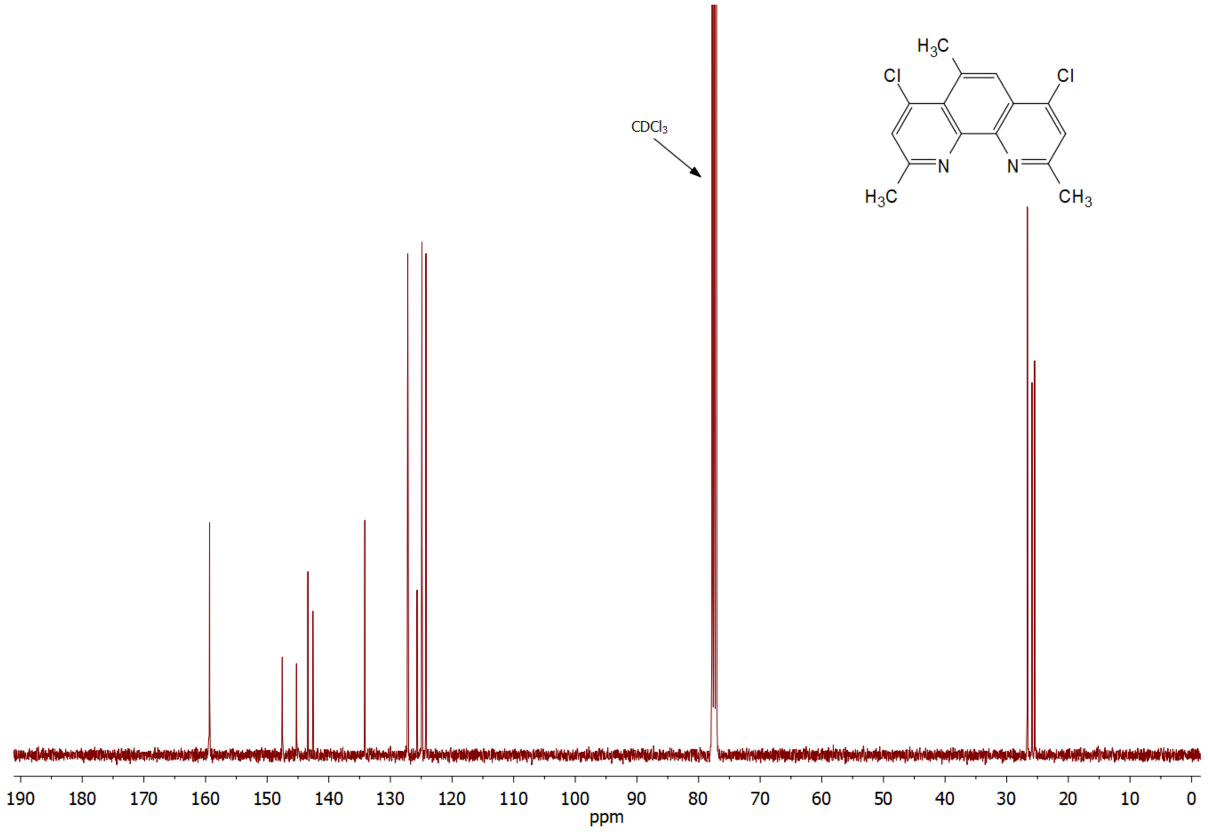

Fig. S11b. $^{13}C\{^{1}H\}$ NMR ($CDCl_3$; 100.5 MHz) spectrum of **4k**.

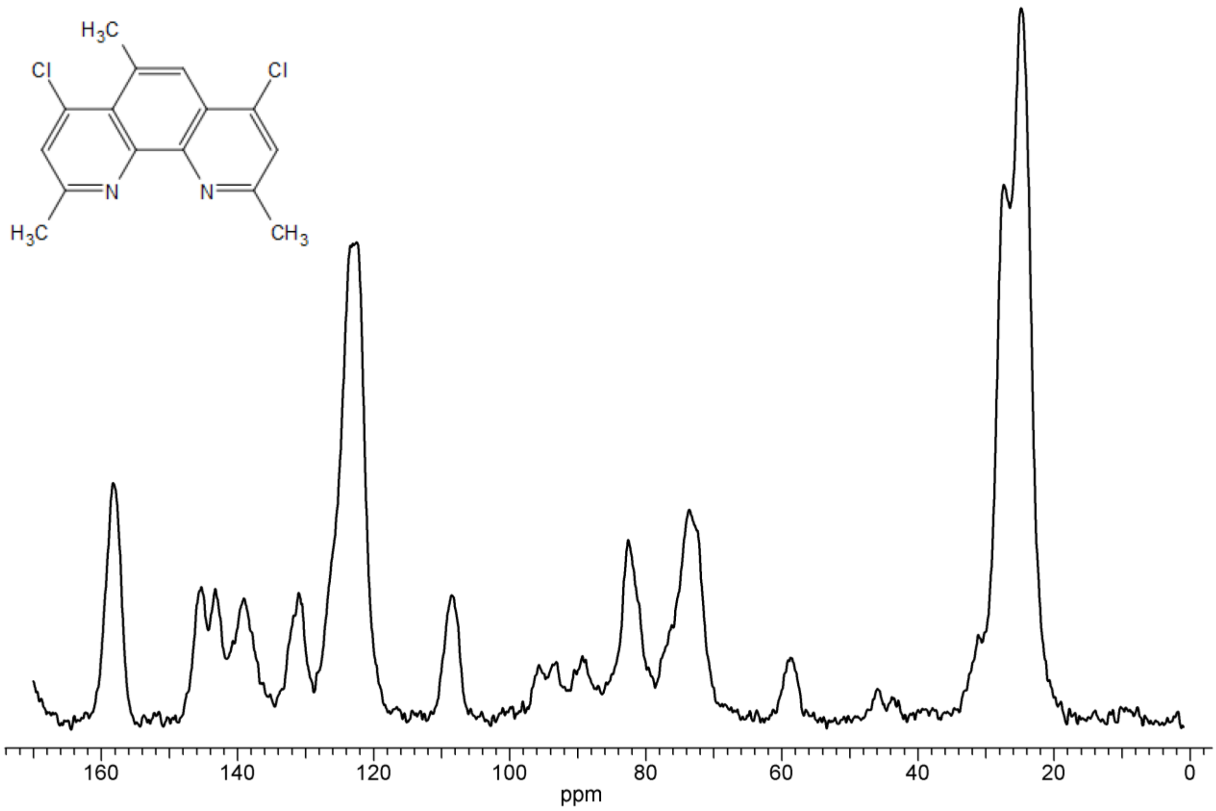

Fig. S11c. ^{13}C CP/MAS NMR spectrum of **4k**.

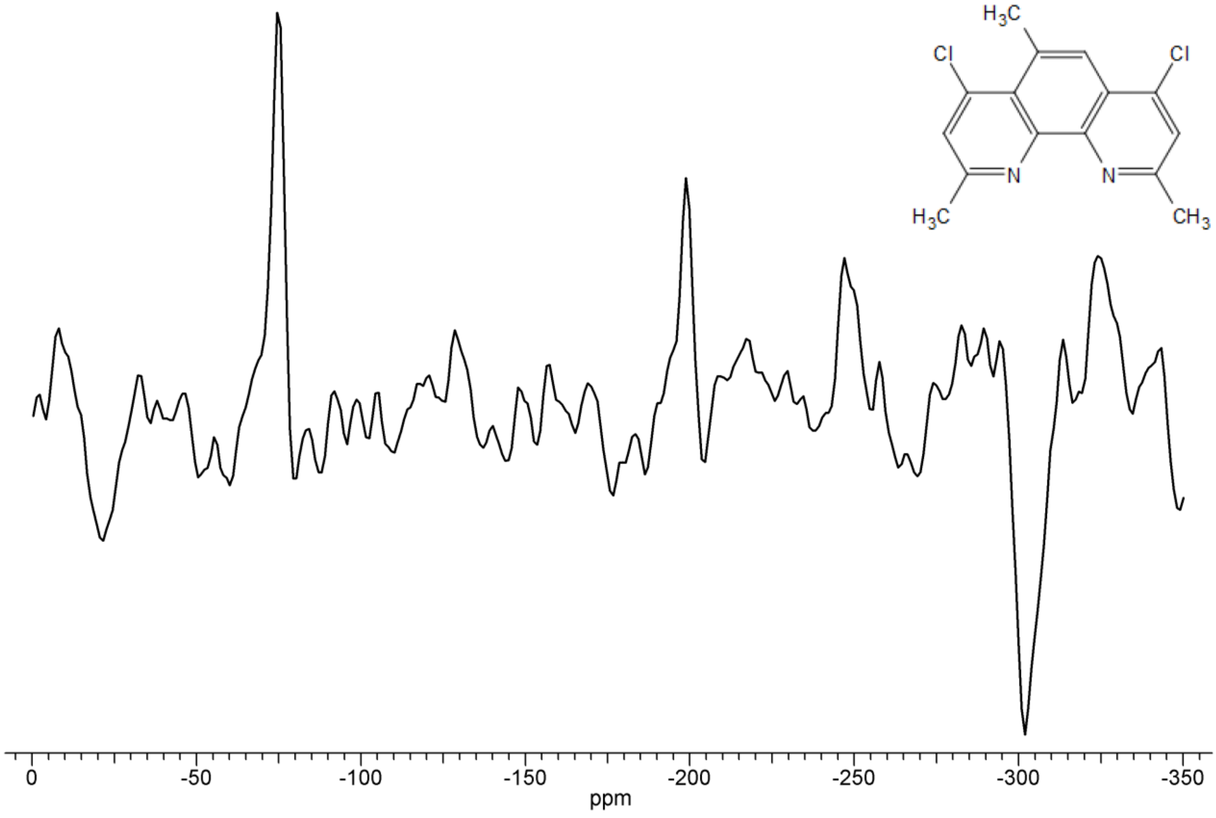

Fig. S11d. ^{15}N CP/MAS NMR spectrum of **4k**.

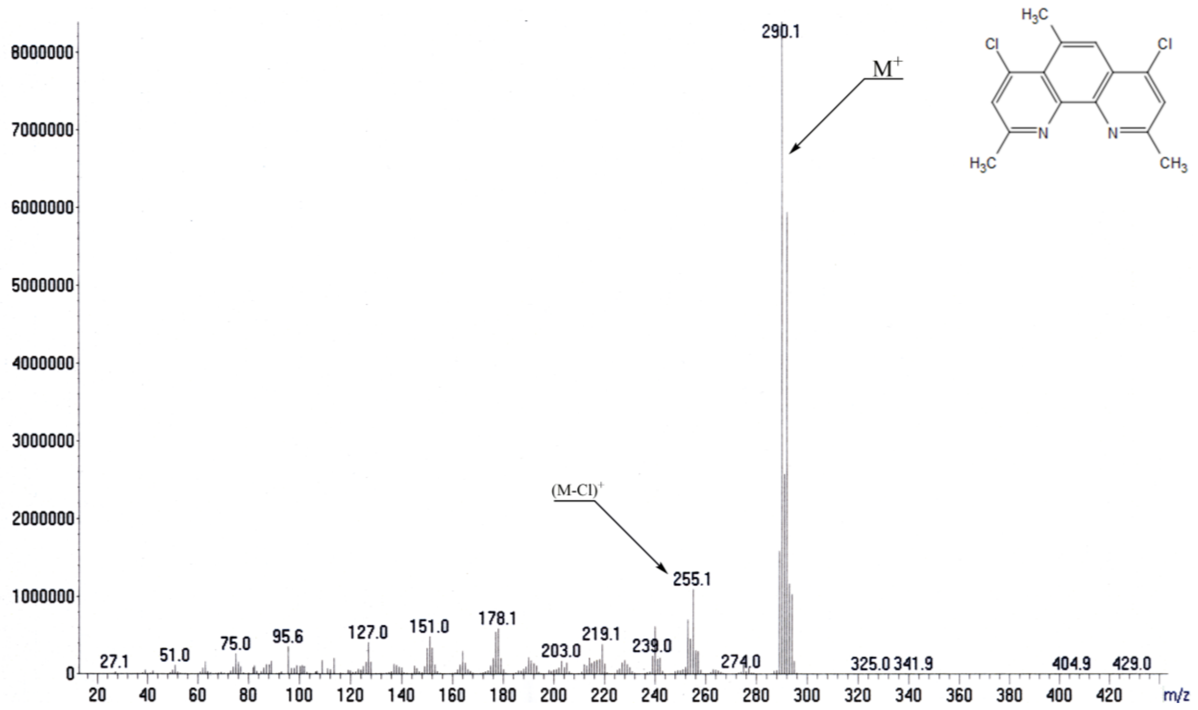

Fig. S11e. MS spectrum of **4k**.

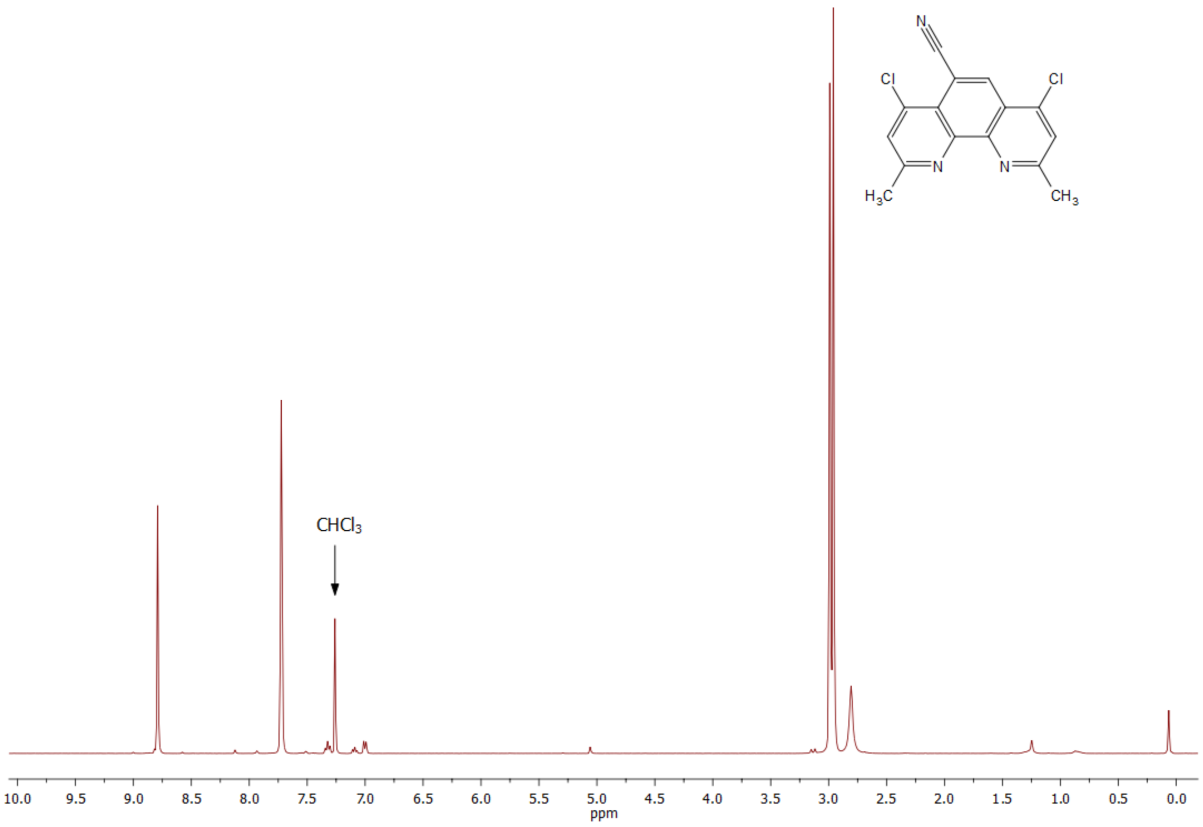

Fig. S12a. ^{1}H NMR ($CDCl_3$; 400.2 MHz) spectrum of **4l**.

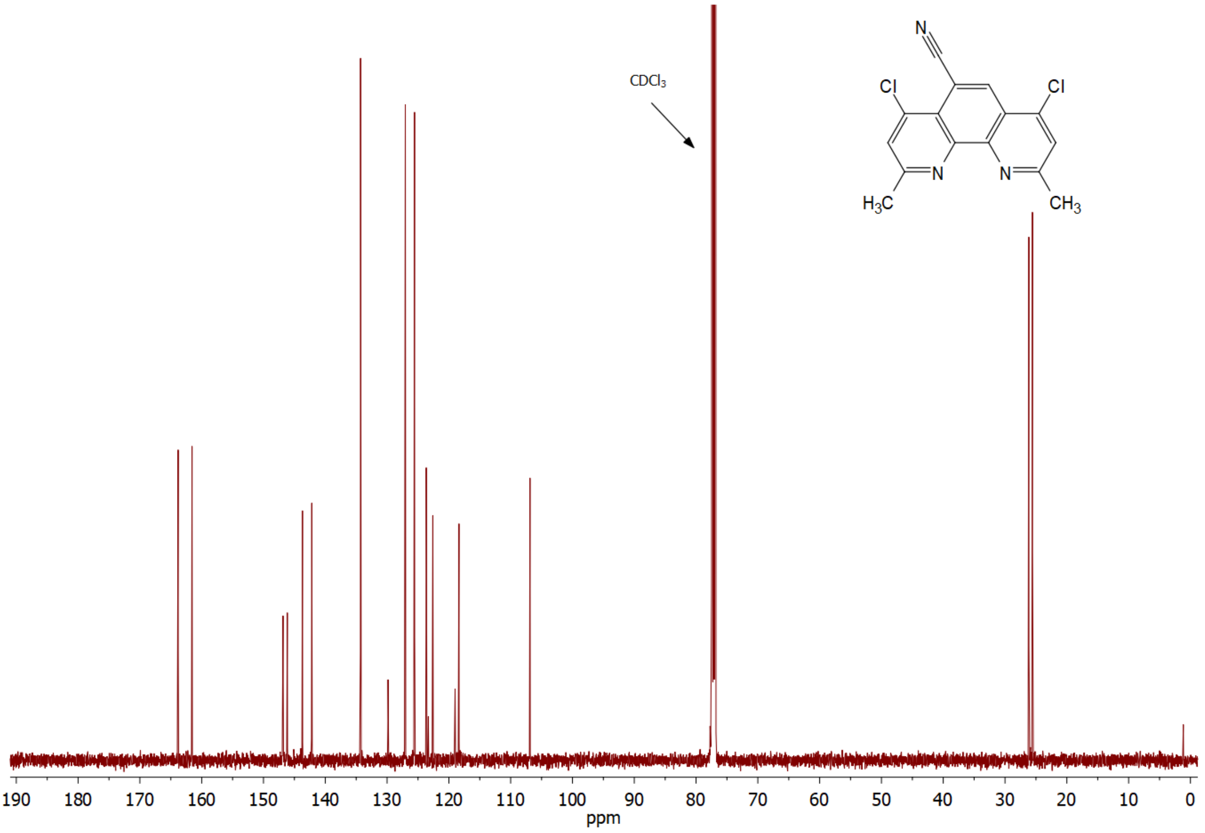

Fig. S12b. $^{13}C\{^{1}H\}$ NMR ($CDCl_3$; 125.8 MHz) spectrum of **4l**.

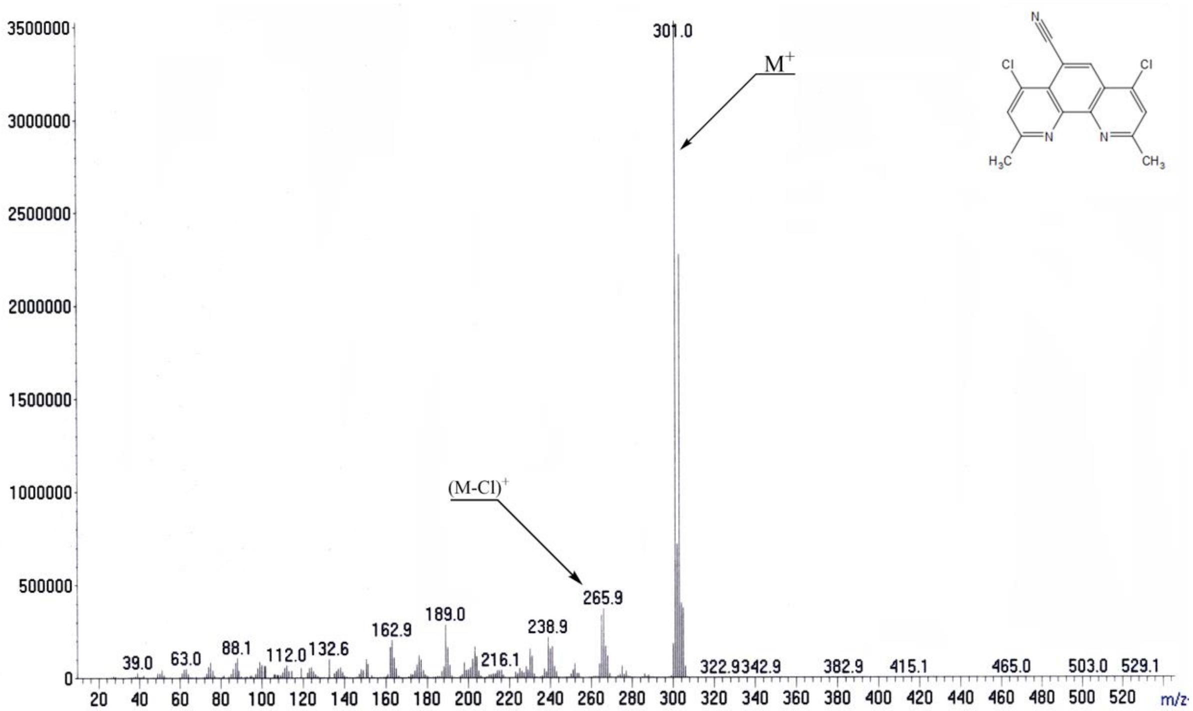

Fig. S12c. MS spectrum of **4l**.

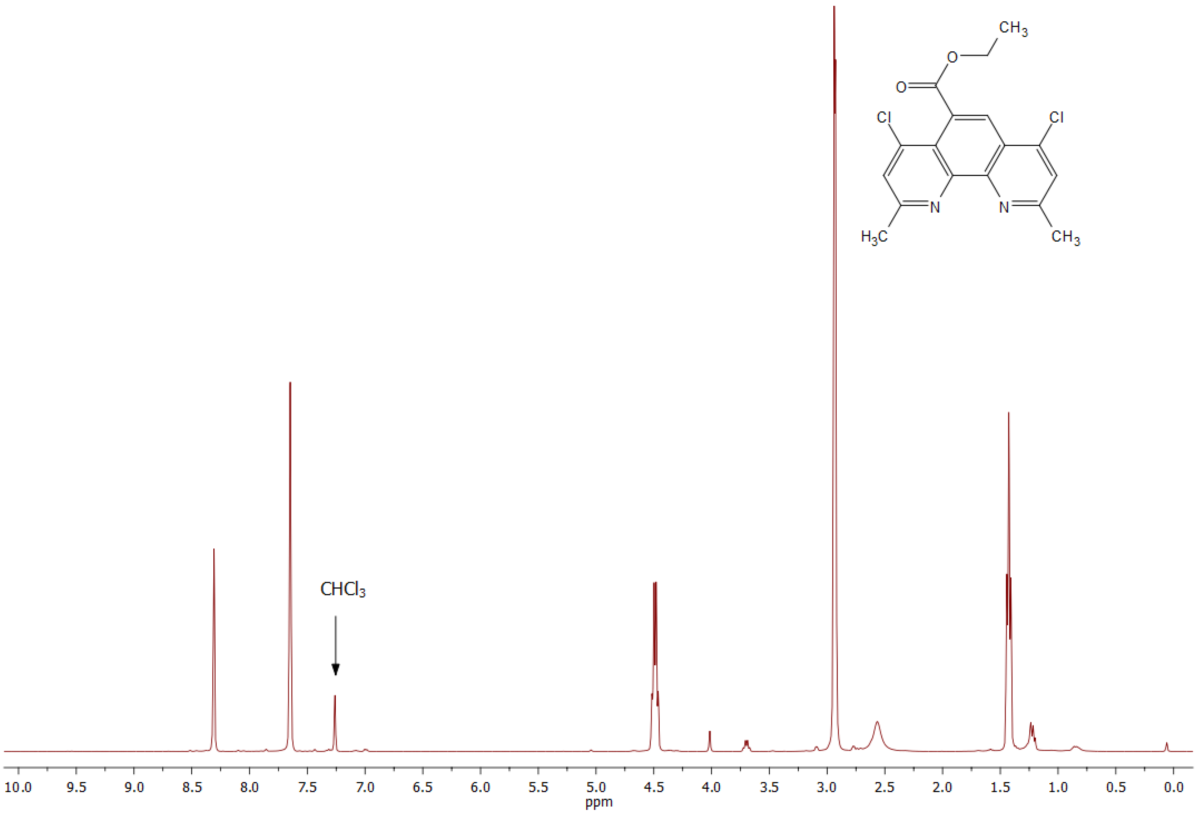

Fig. S13a. ^{1}H NMR ($CDCl_3$; 400.2 MHz) spectrum of **4m**.

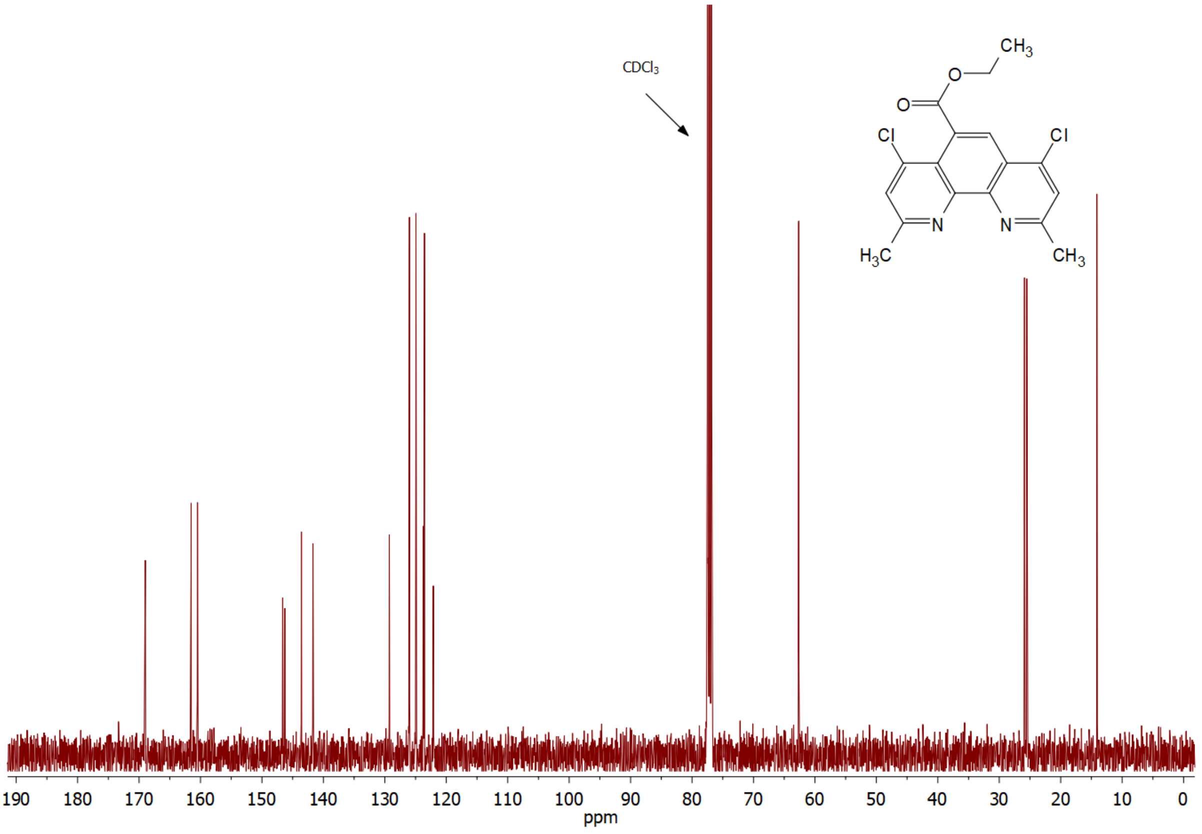

Fig. S13b. $^{13}C\{^{1}H\}$ NMR ($CDCl_3$; 100.5 MHz) spectrum of **4m**.

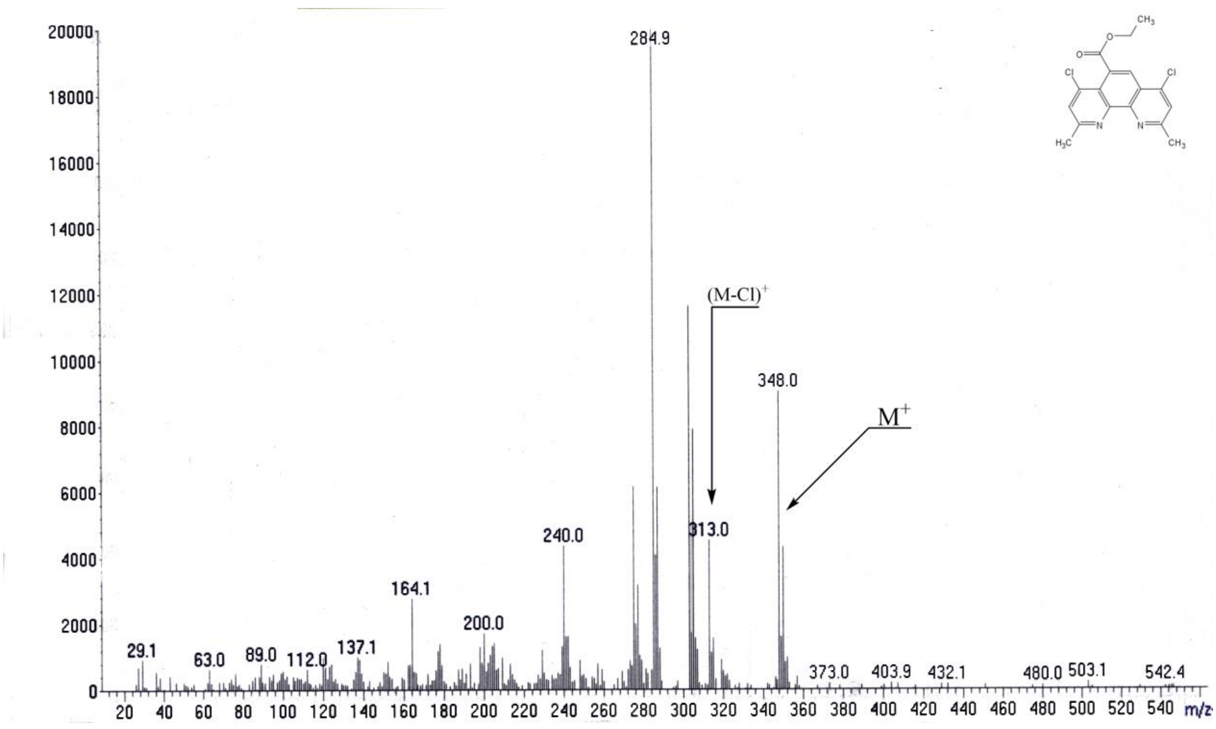

Fig. S13c. MS spectrum of **4m**.

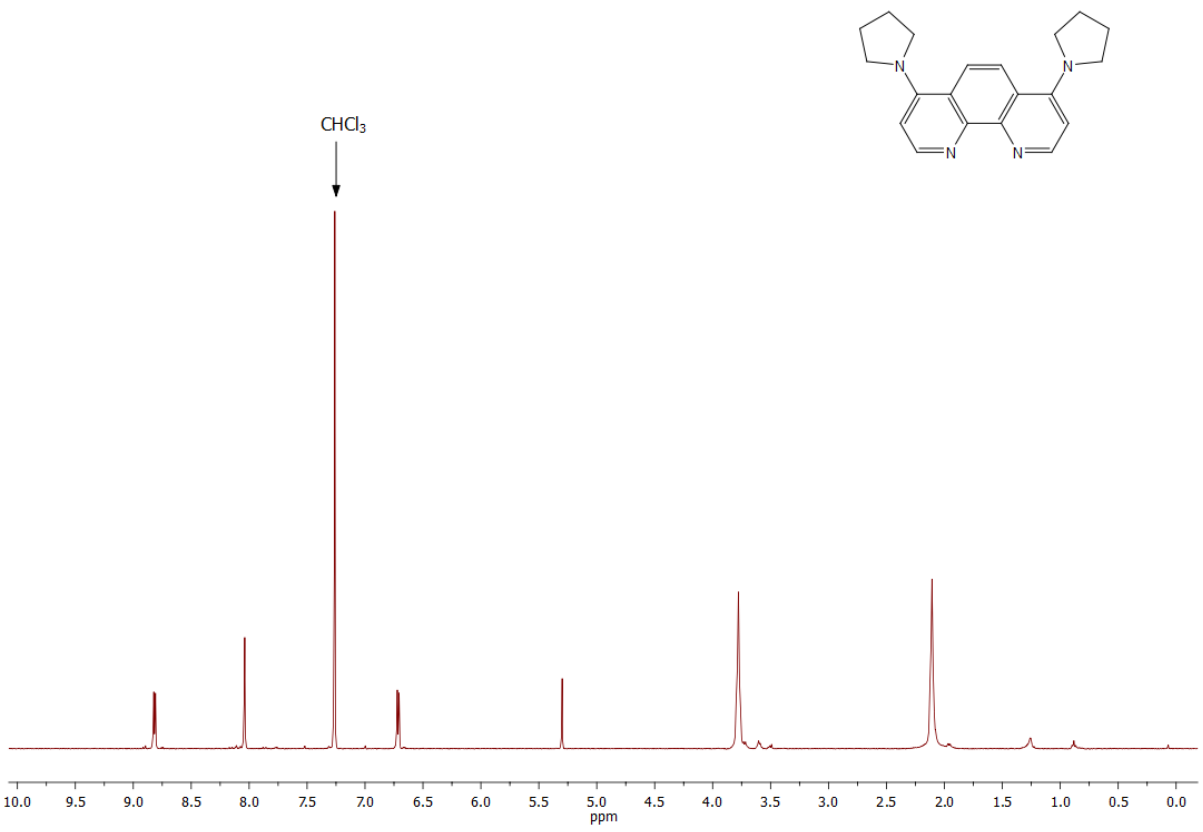

Fig. S14a. ^{1}H NMR ($CDCl_3$; 400.2 MHz) spectrum of **5a**.

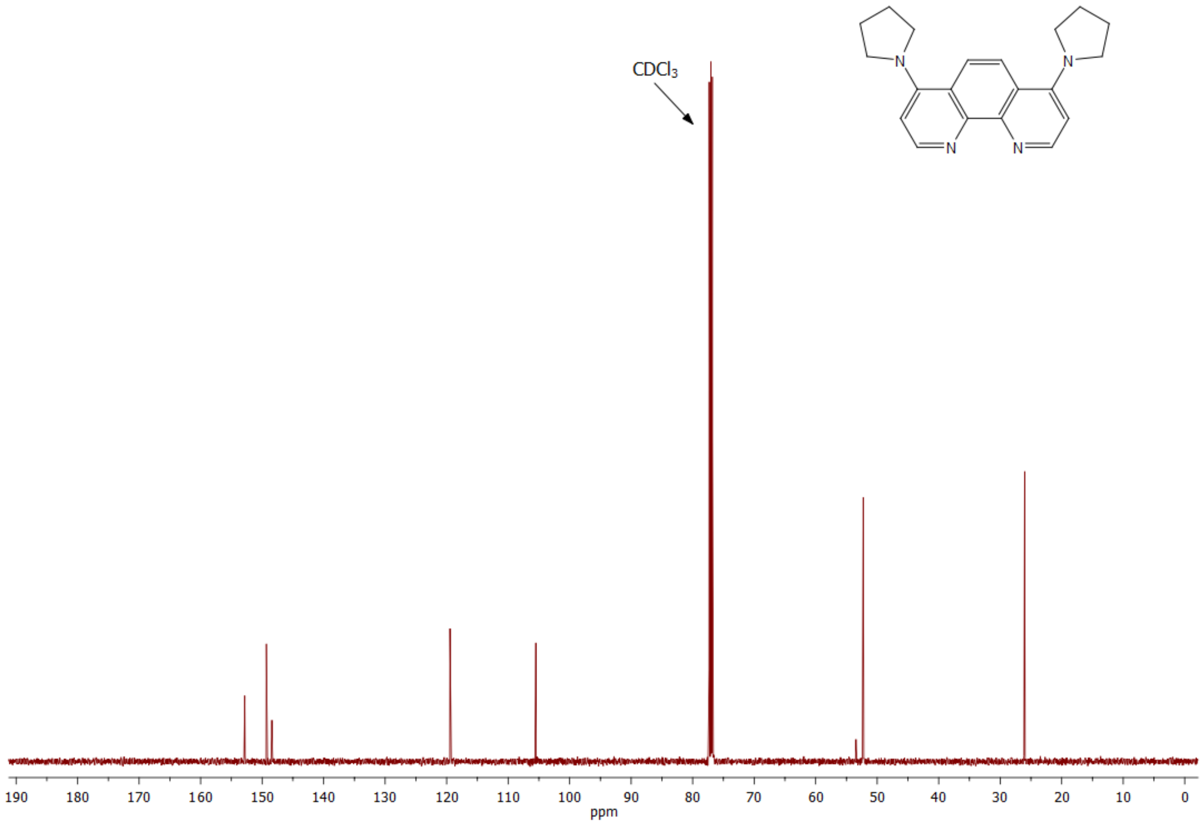

Fig. S14b. $^{13}C\{^{1}H\}$ NMR ($CDCl_3$; 100.5 MHz) spectrum of **5a**.

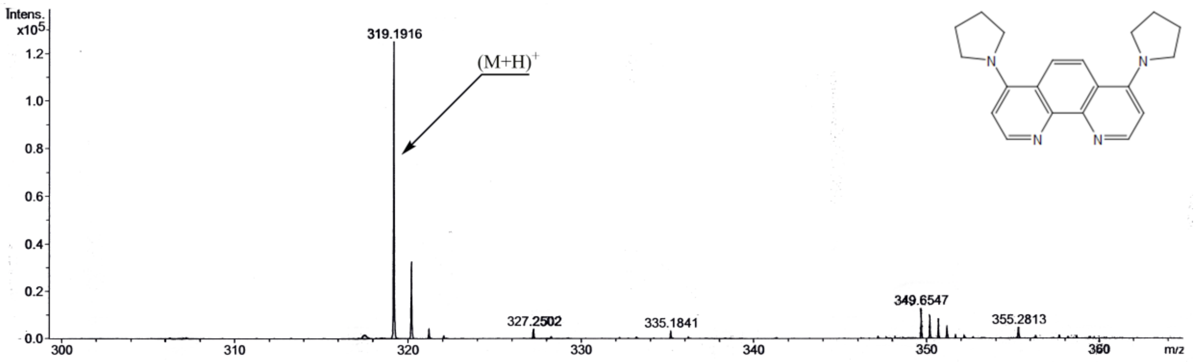

Fig. S14c. MS spectrum of **5a**.

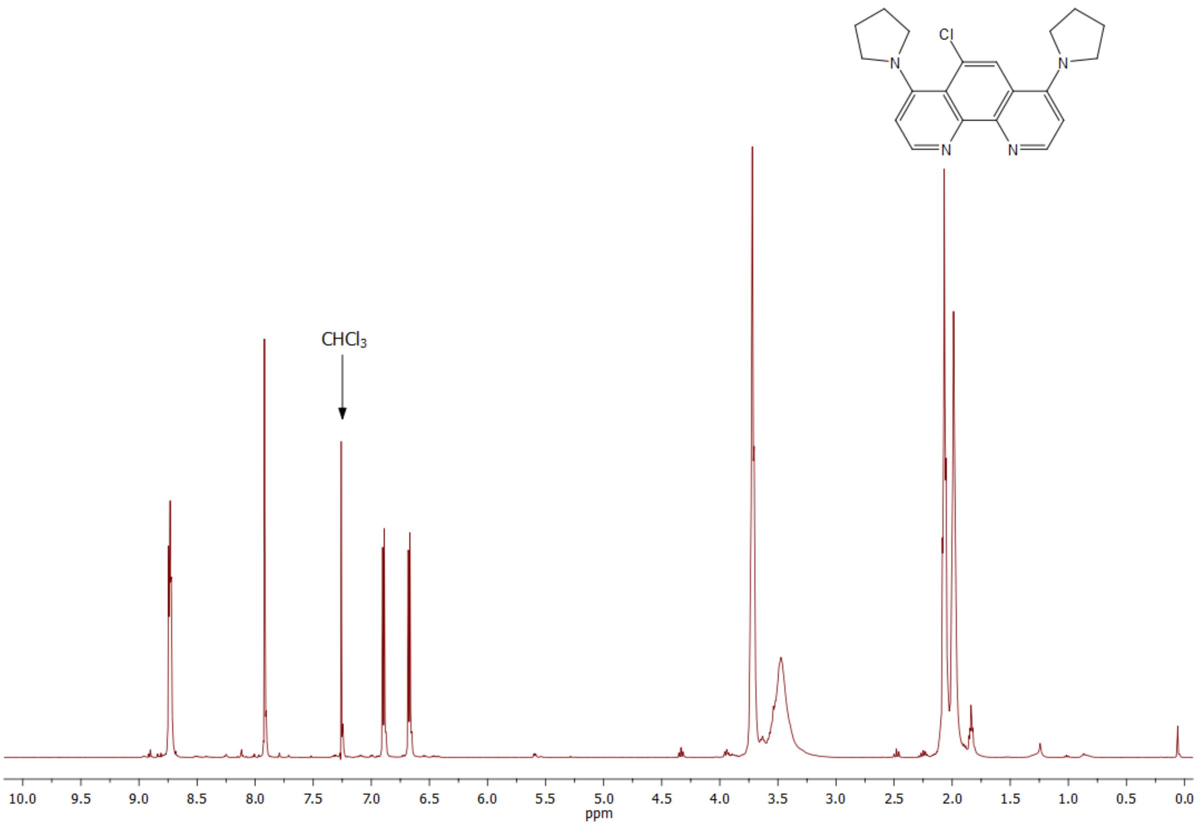

Fig. S15a. ^{1}H NMR ($CDCl_3$; 400.2 MHz) spectrum of **5b**.

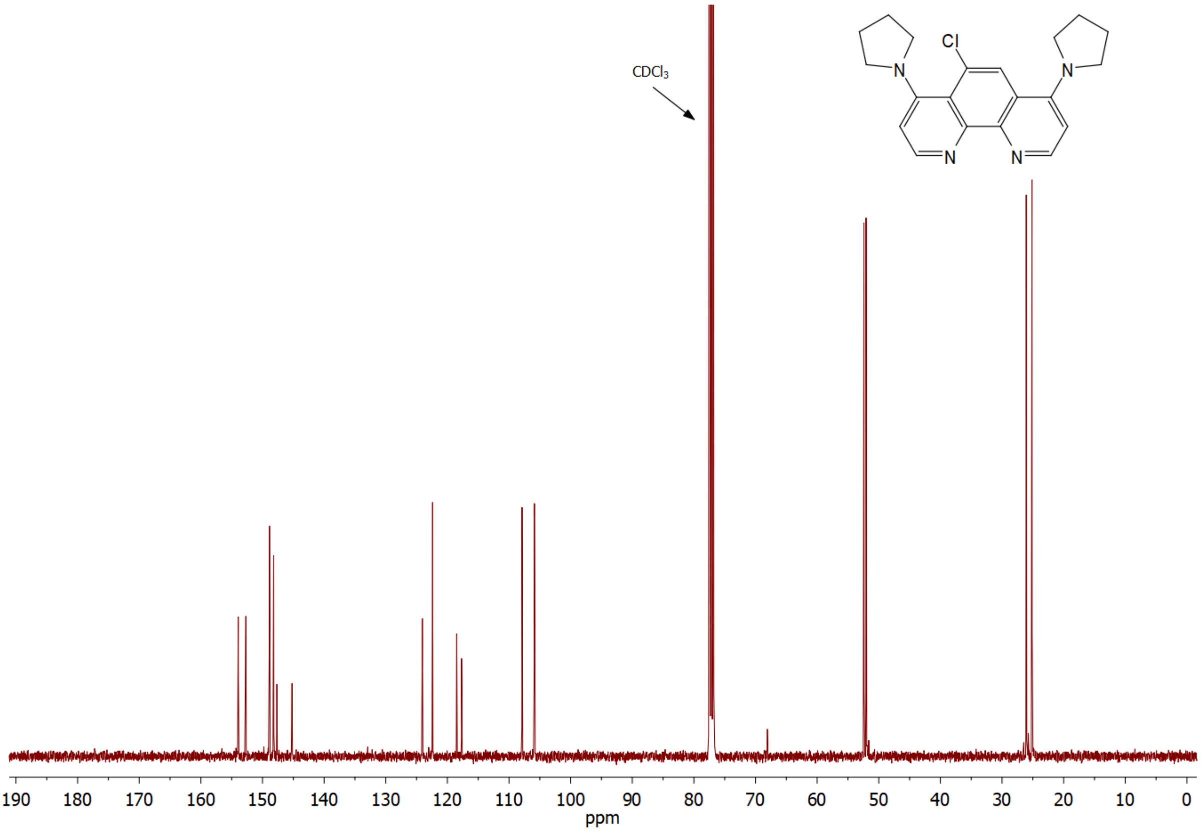

Fig. S15b. $^{13}C\{^{1}H\}$ NMR ($CDCl_3$; 100.5 MHz) spectrum of **5b**.

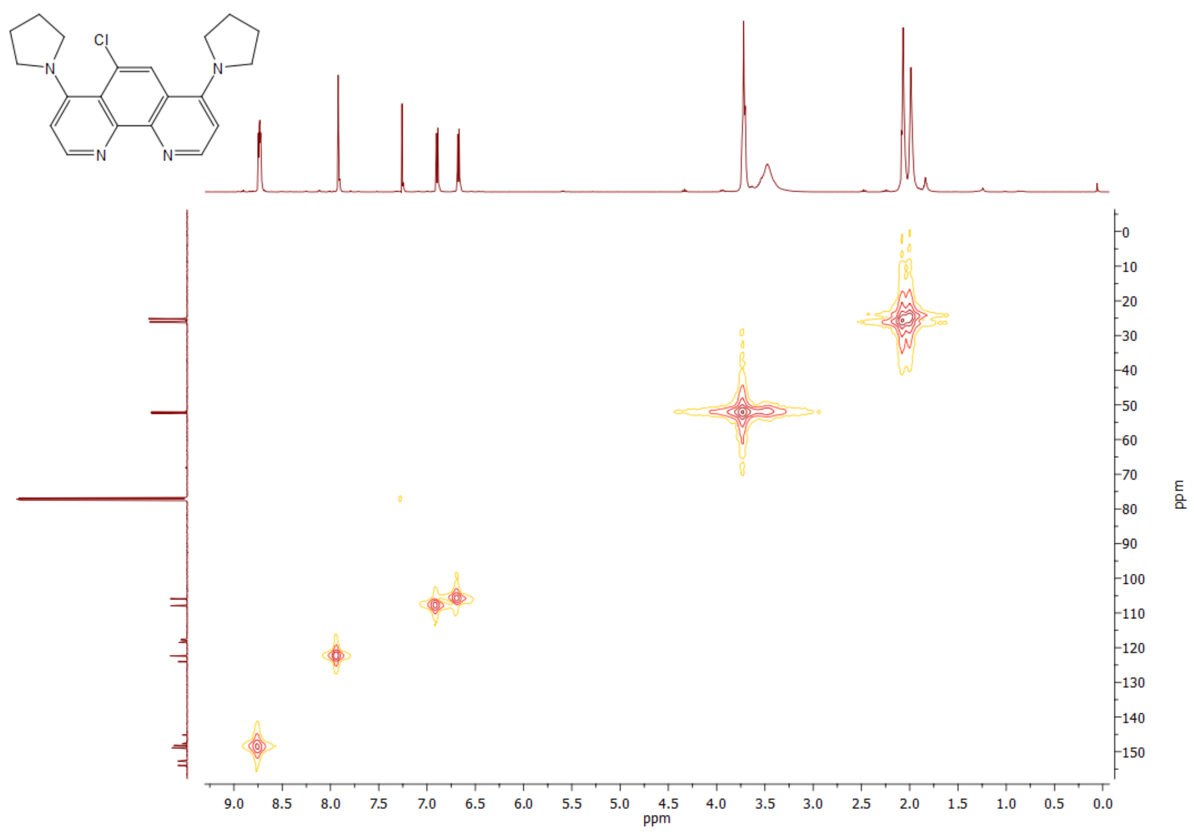

Fig. S15c. ^{1}H, ^{13}C NMR HMQC in $CDCl_3$ spectrum of **5b**.

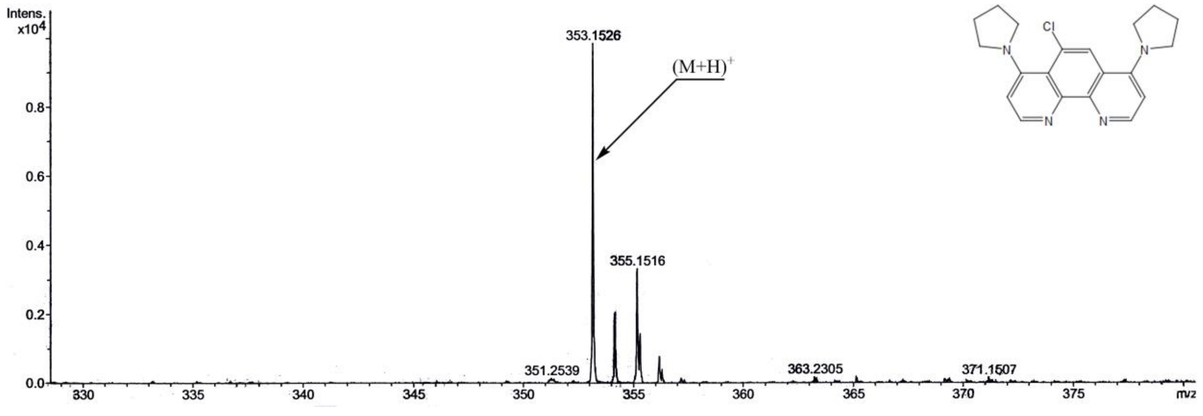

Fig. S15d. MS spectrum of **5b**.

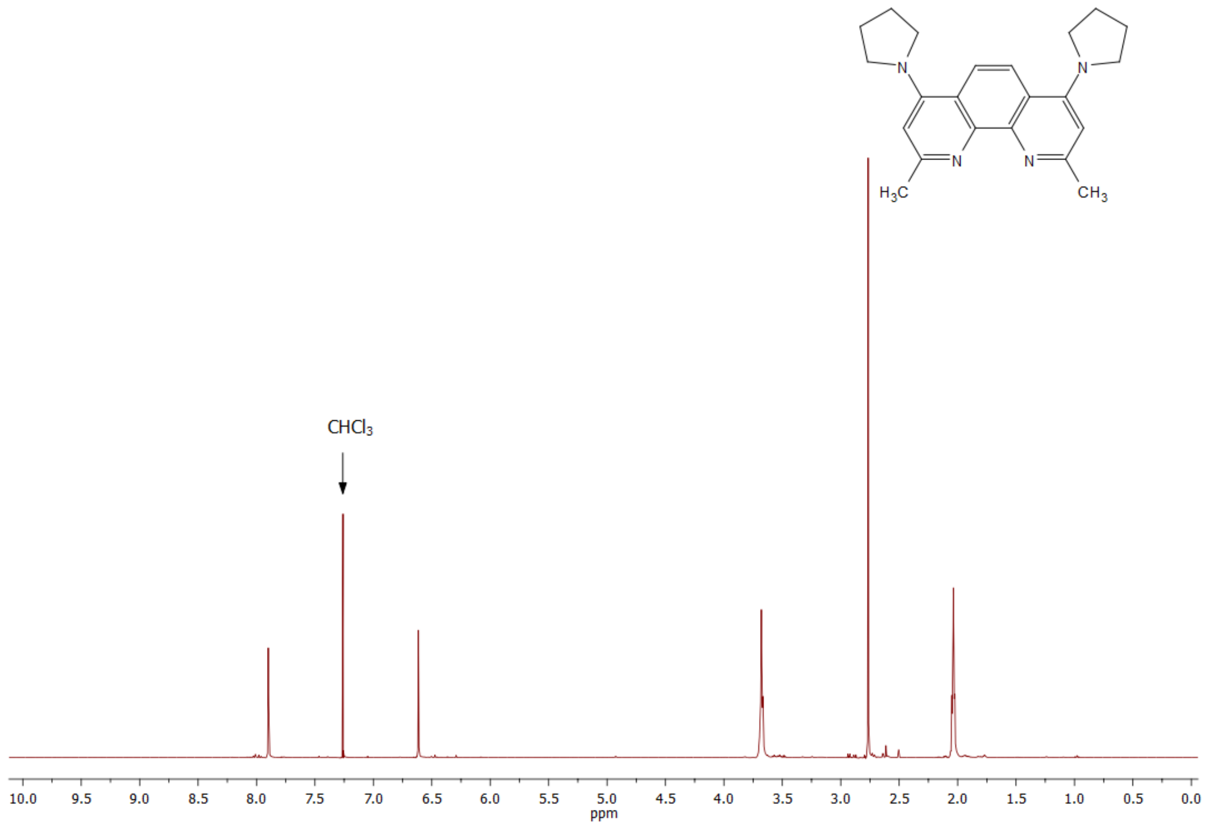

Fig. S16a. ^{1}H NMR ($CDCl_3$; 500.2 MHz) spectrum of **5c**.

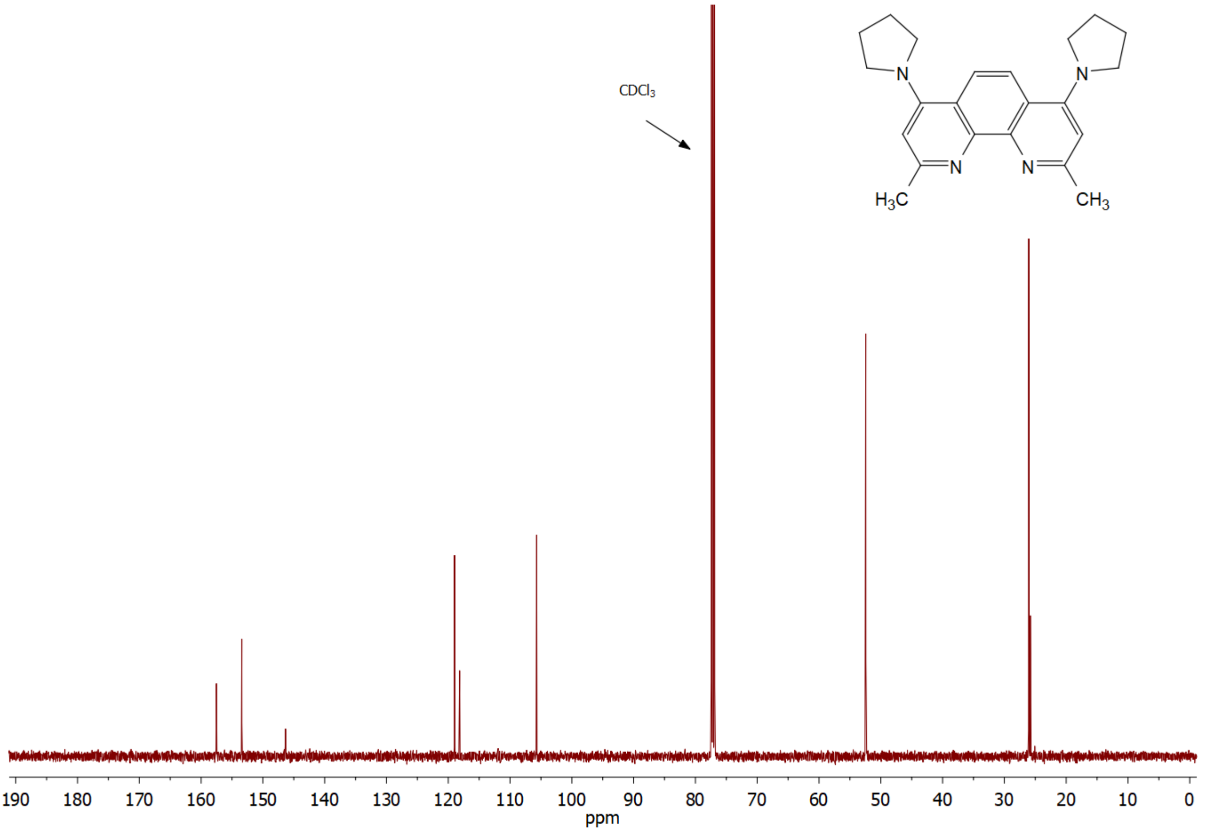

Fig. S16b. $^{13}C\{^{1}H\}$ NMR ($CDCl_3$; 100.5 MHz) spectrum of **5c**.

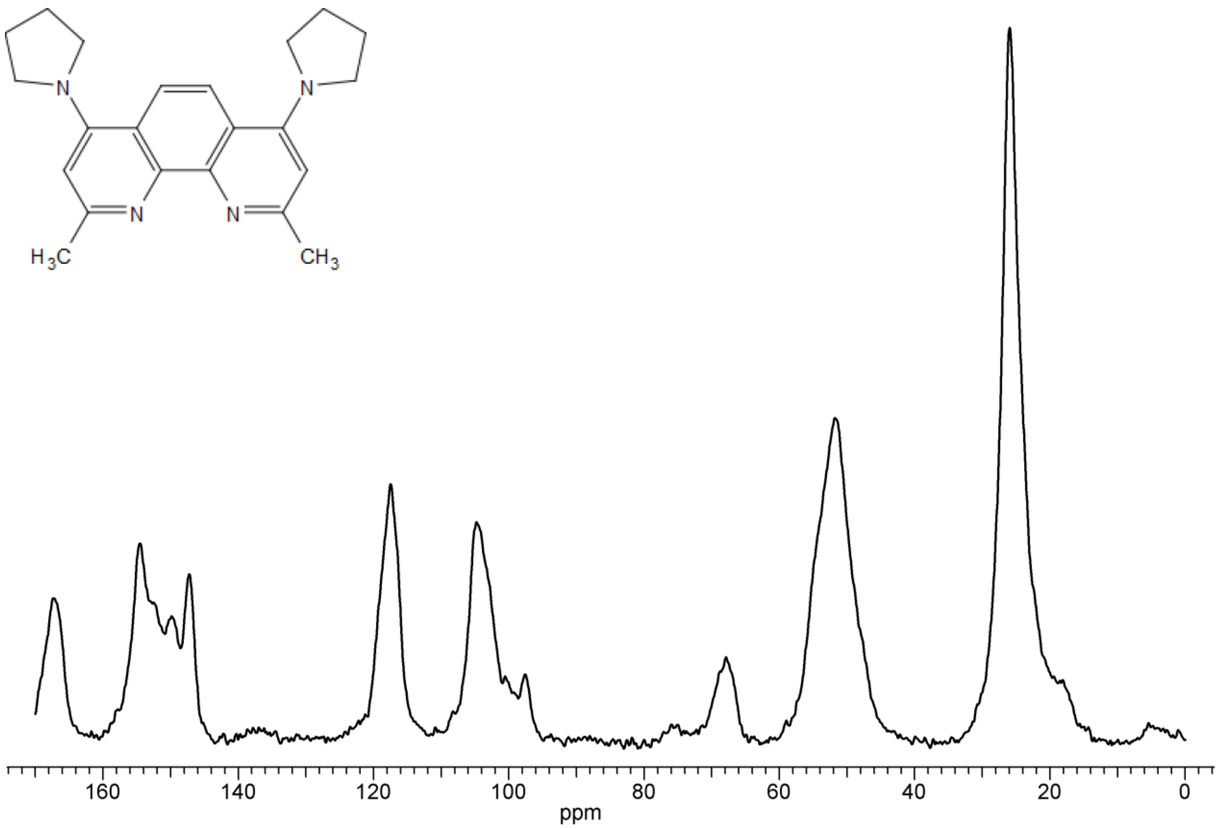

Fig. S16c. ^{13}C CP/MAS NMR spectrum of **5c**.

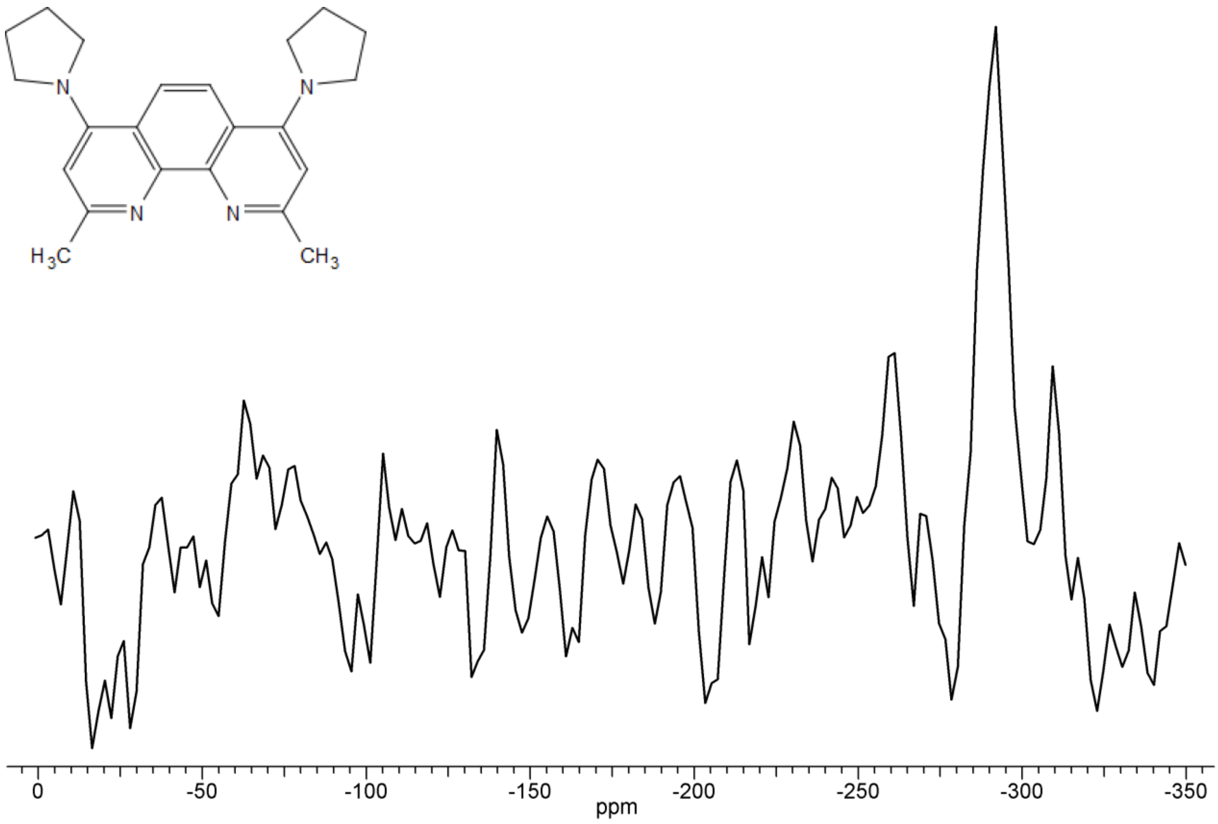

Fig. S16d. ^{15}N CP/MAS NMR spectrum of **5c**.

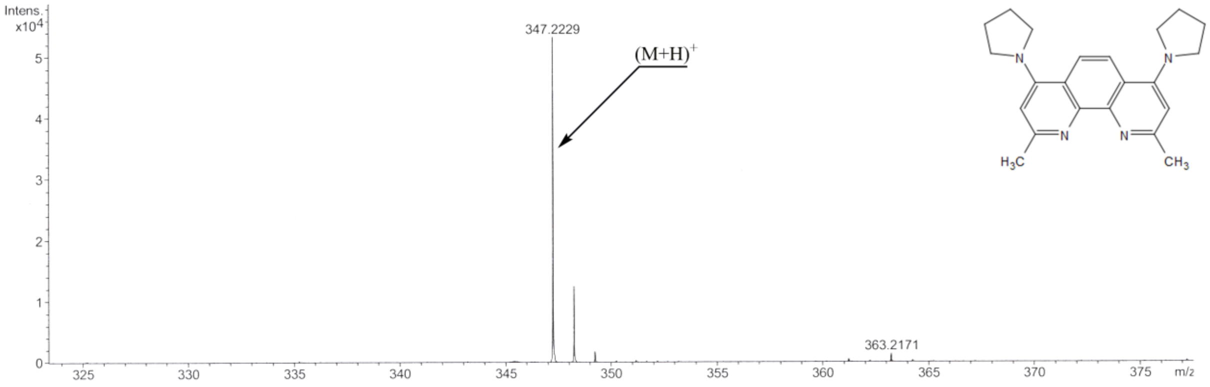

Fig. S16e. MS spectrum of **5c**.

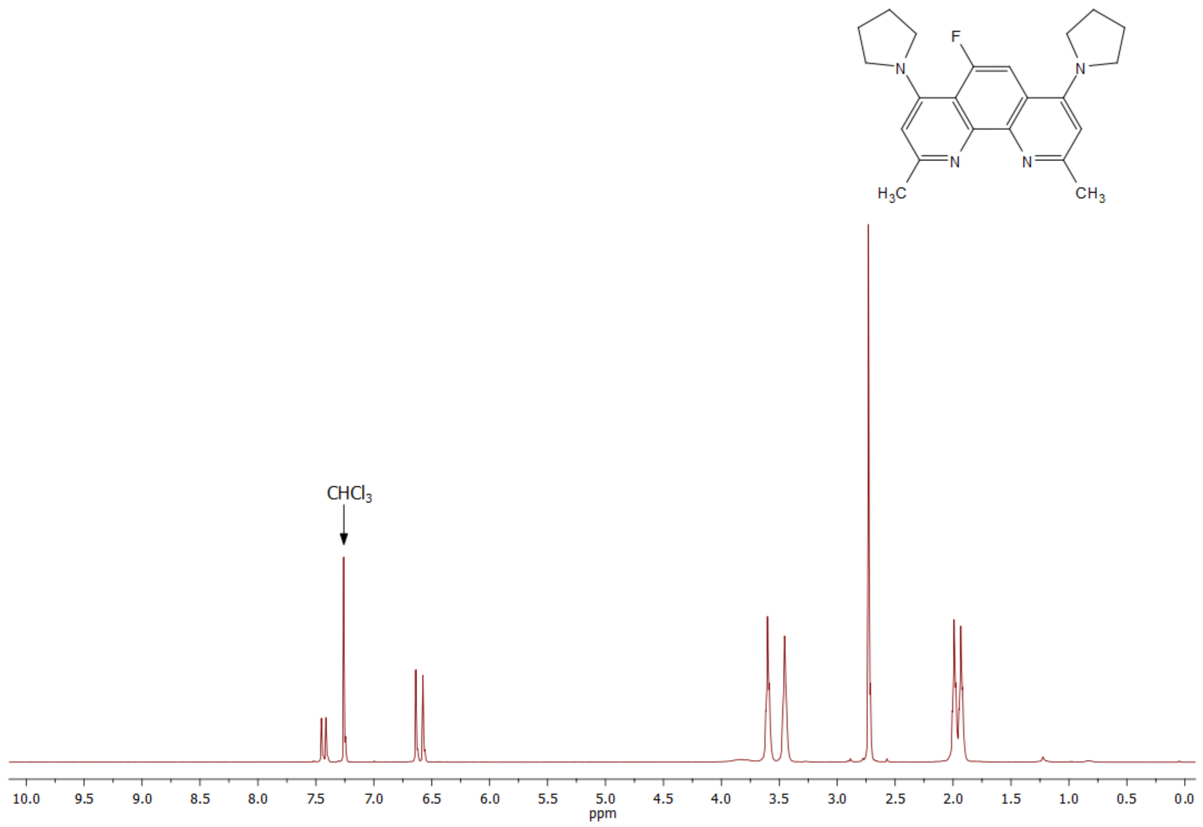

Fig. S17a. ^{1}H NMR ($CDCl_3$; 500.2 MHz) spectrum of **5d**.

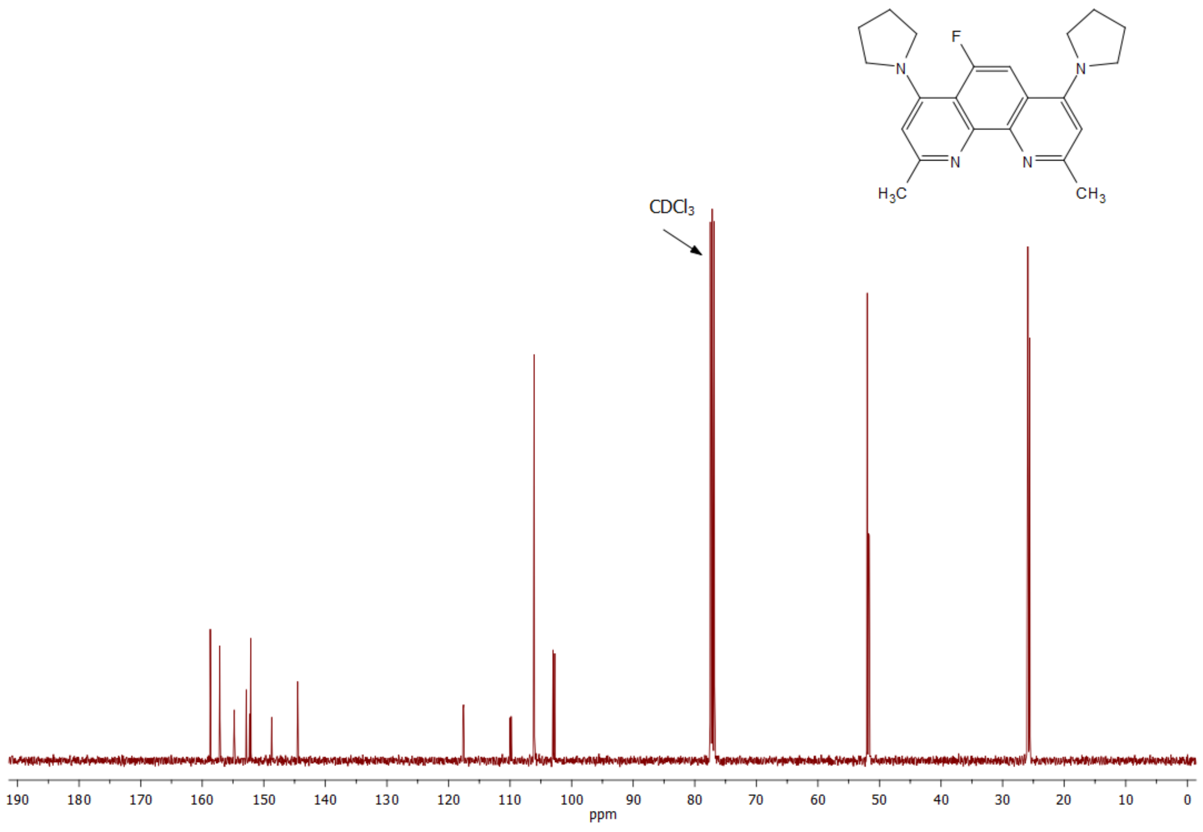

Fig. S17b. ^{13}C{^{1}H} NMR ($CDCl_3$; 100.5 MHz) spectrum of **5d**.

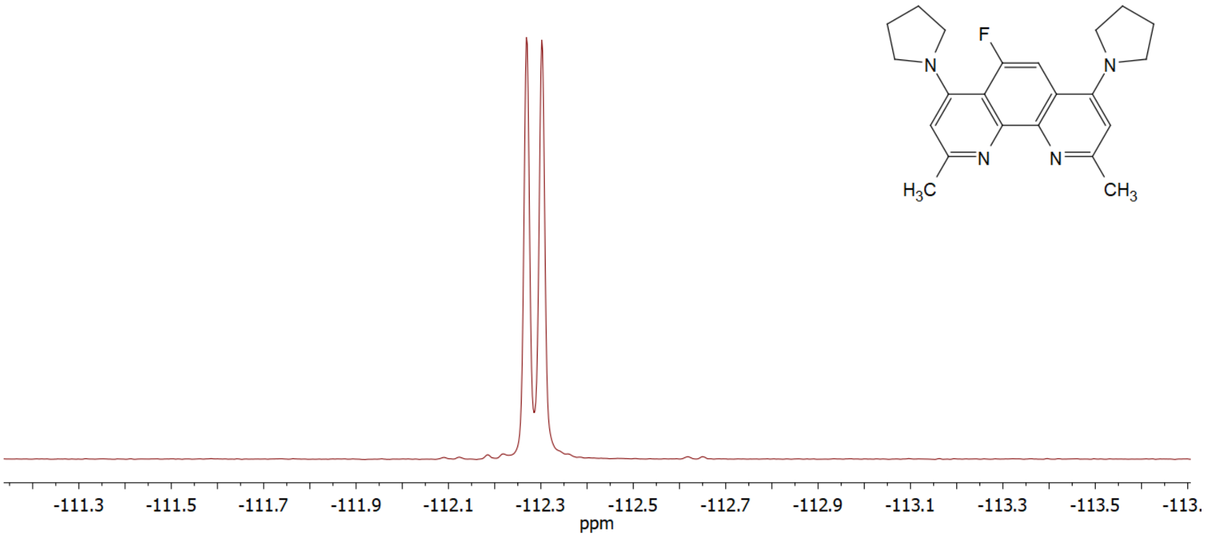

Fig. S17c. ^{19}F NMR ($CDCl_3$; 470.5 MHz) spectrum of **5d.**

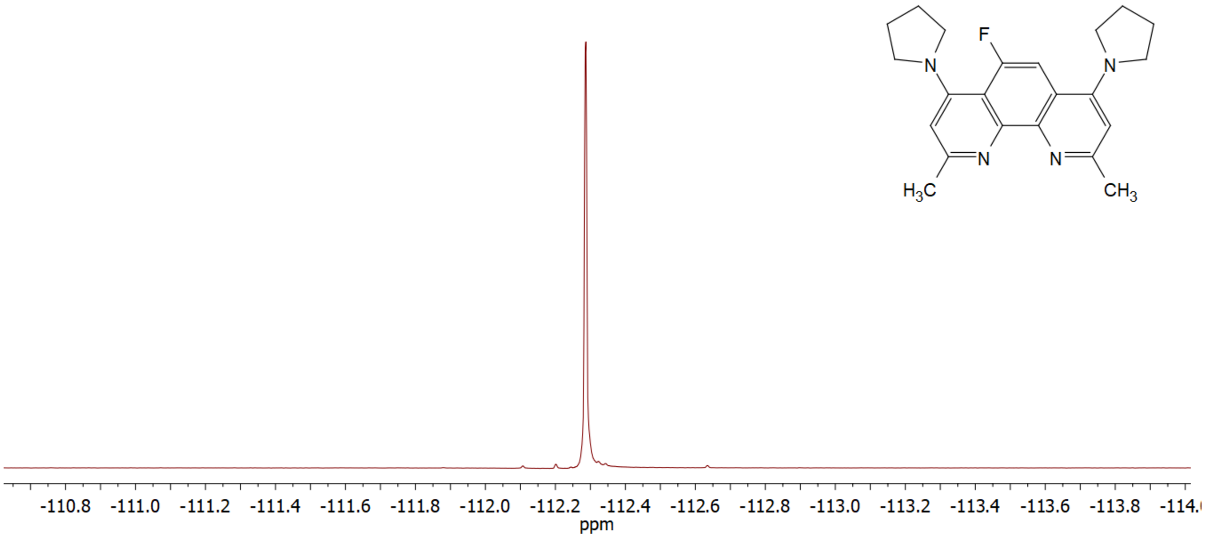

Fig. S17d. $^{19}F\{^1H\}$ NMR ($CDCl_3$; 470.5 MHz) spectrum of **5d**.

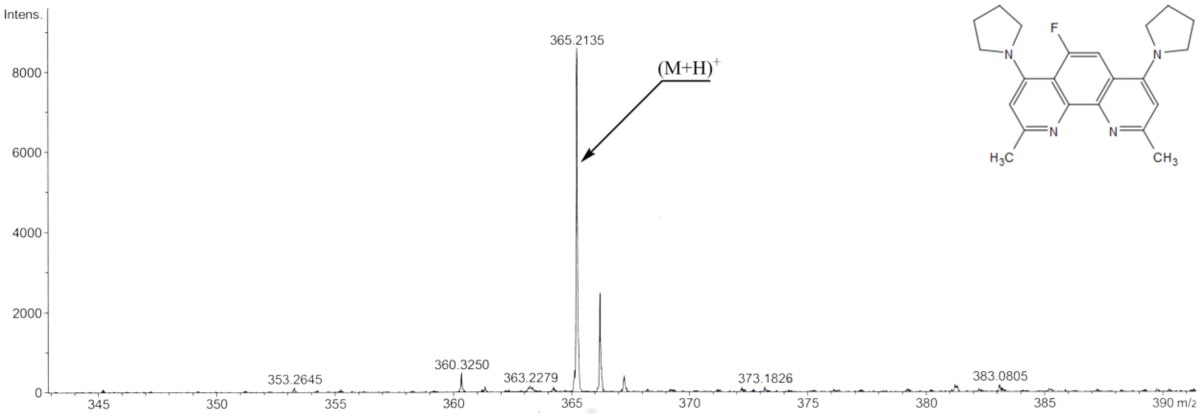

Fig. S17e. MS spectrum of **5d**.

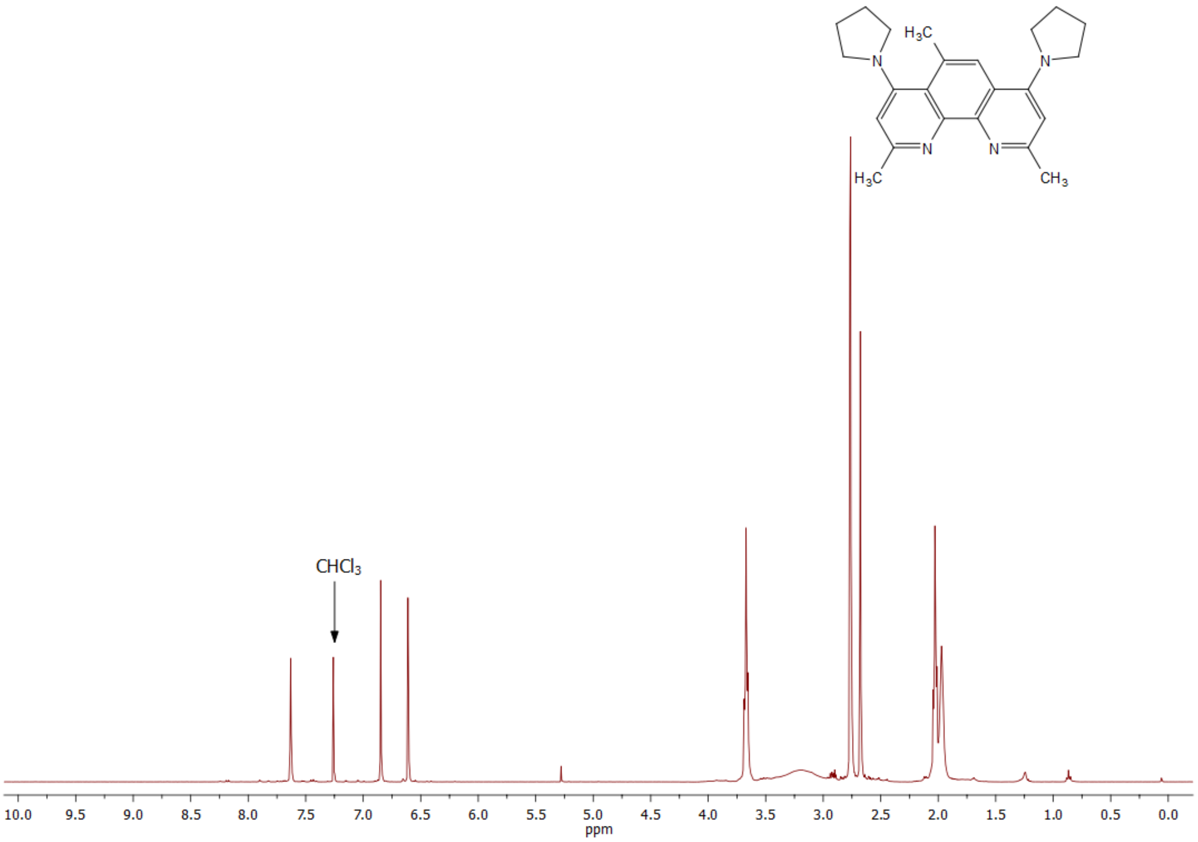

Fig. S18a. ^{1}H NMR ($CDCl_3$; 400.2 MHz) spectrum of **5e**.

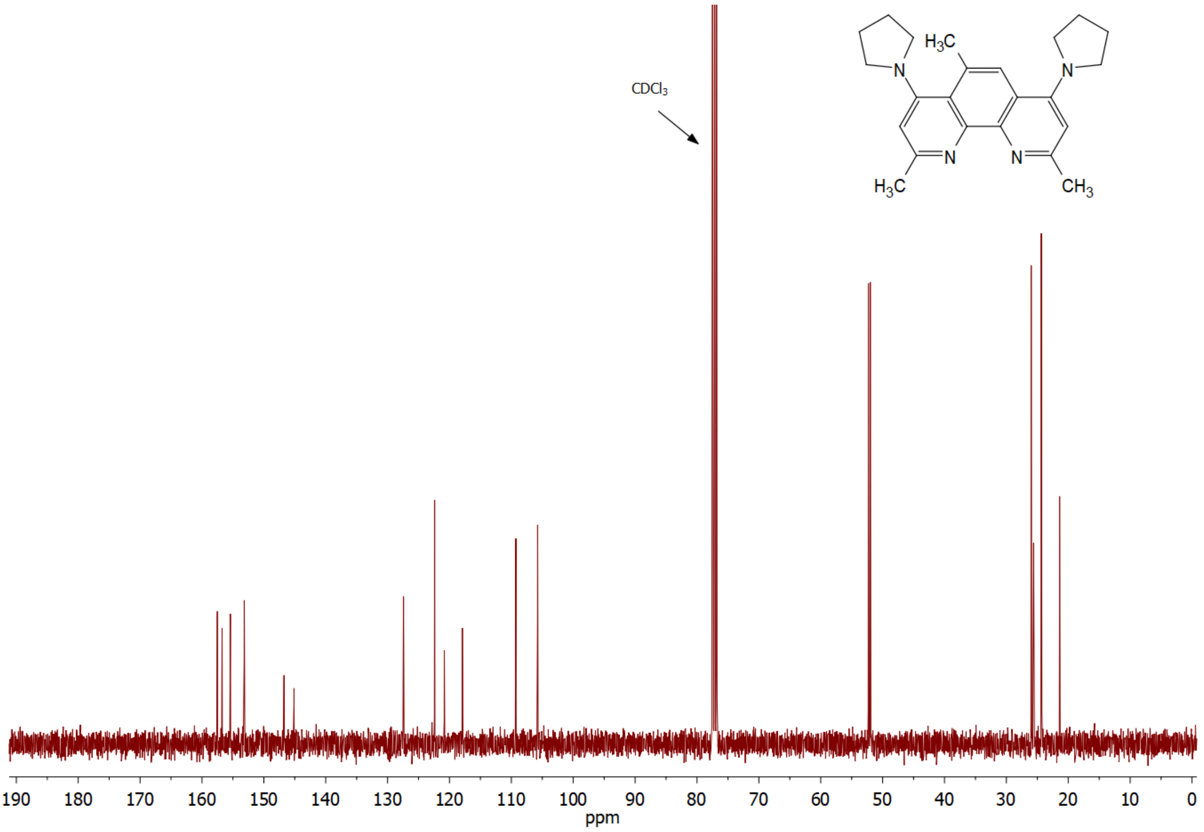

Fig. S18b. $^{13}C\{^{1}H\}$ NMR ($CDCl_3$; 100.5 MHz) spectrum of **5e**.

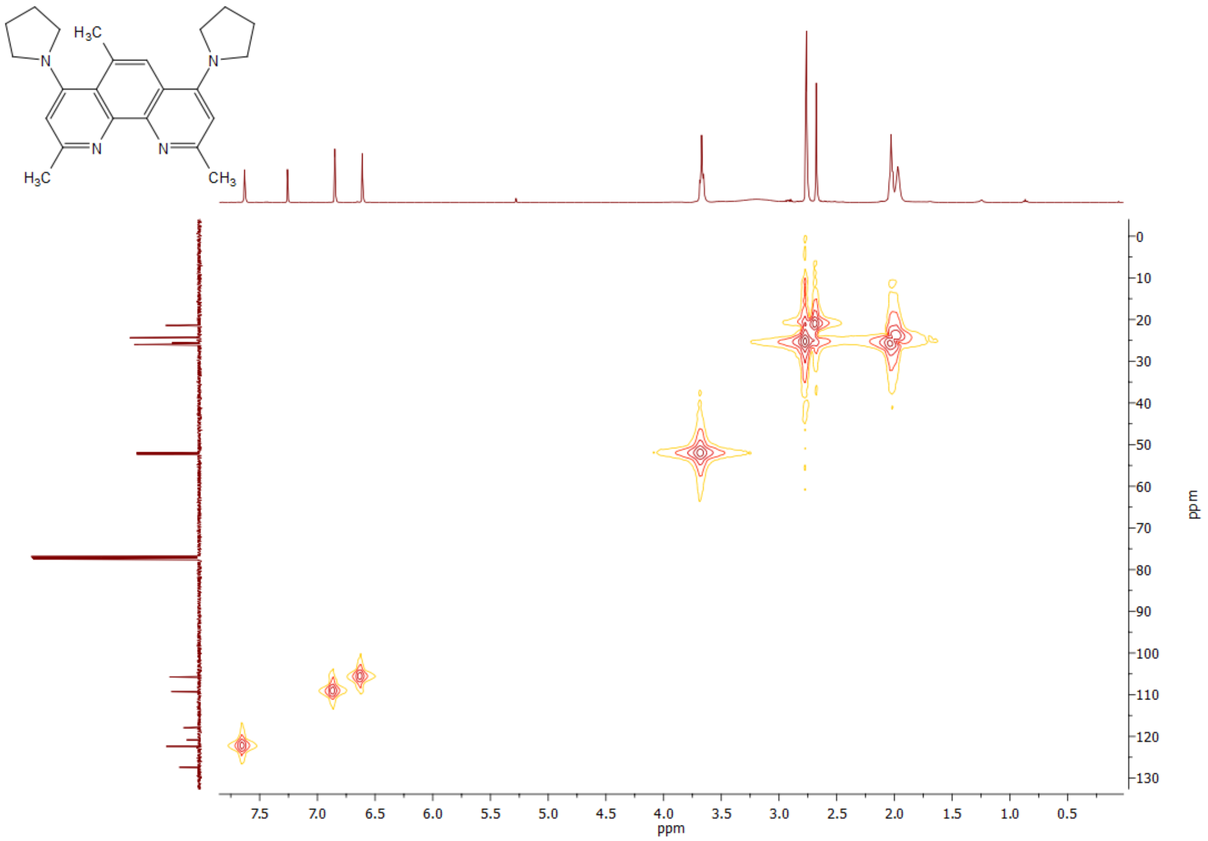

Fig. S18c. ^{1}H, ^{13}C NMR HMQC in $CDCl_3$ spectrum of **5e**.

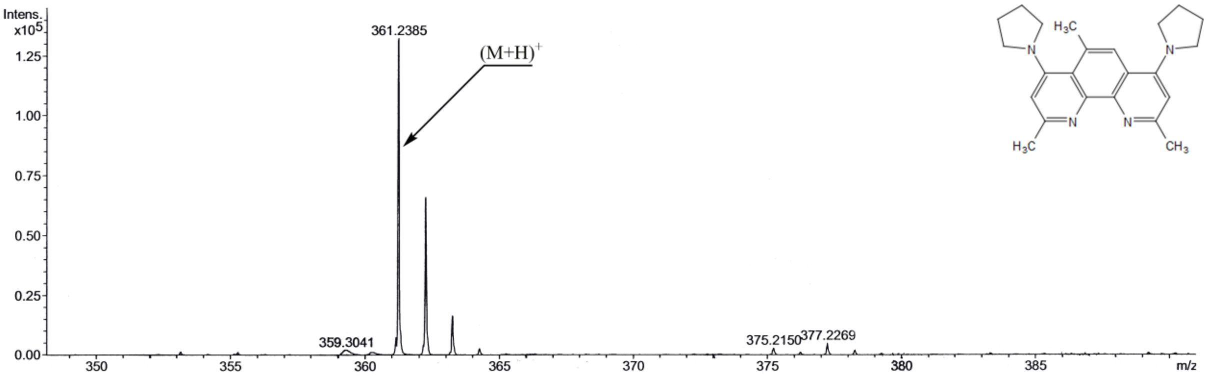

Fig. S18d. MS spectrum of **5e**

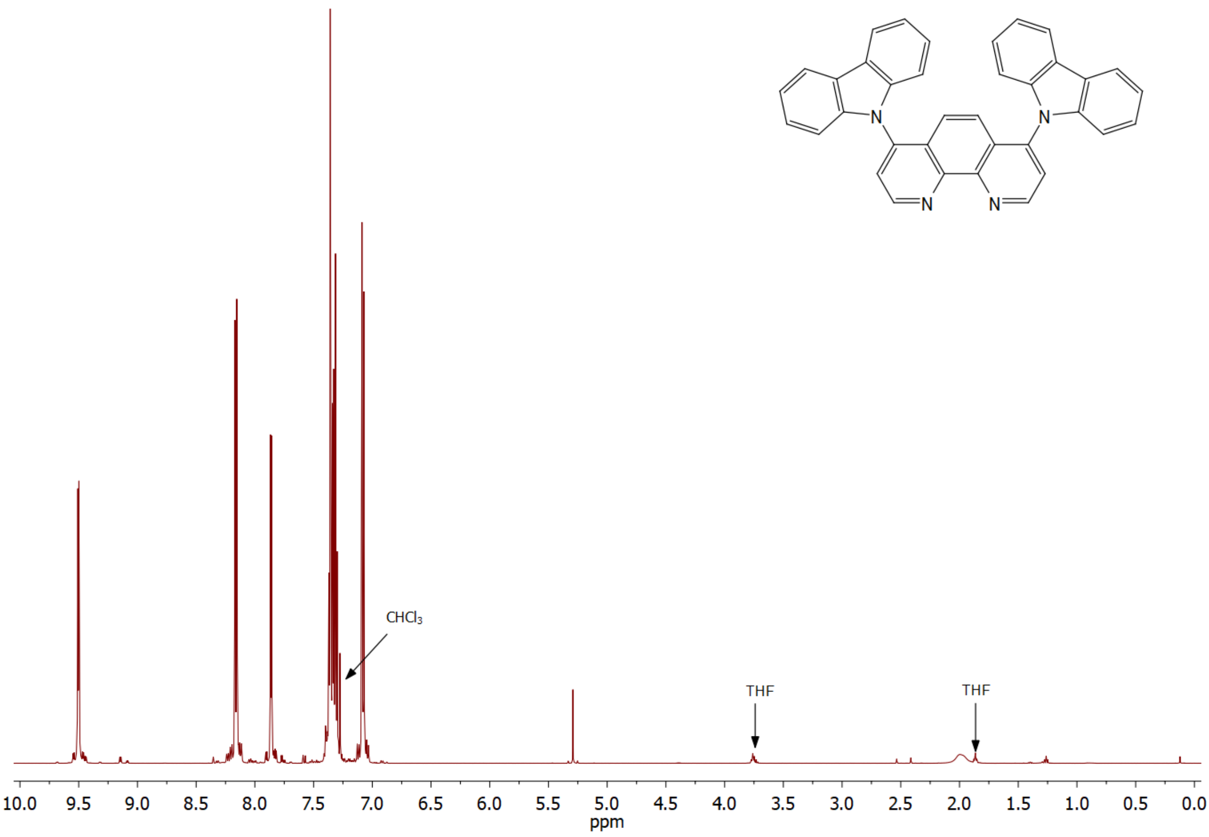

Fig. S19a. ^{1}H NMR ($CDCl_3$; 500.2 MHz) spectrum of **5f**.

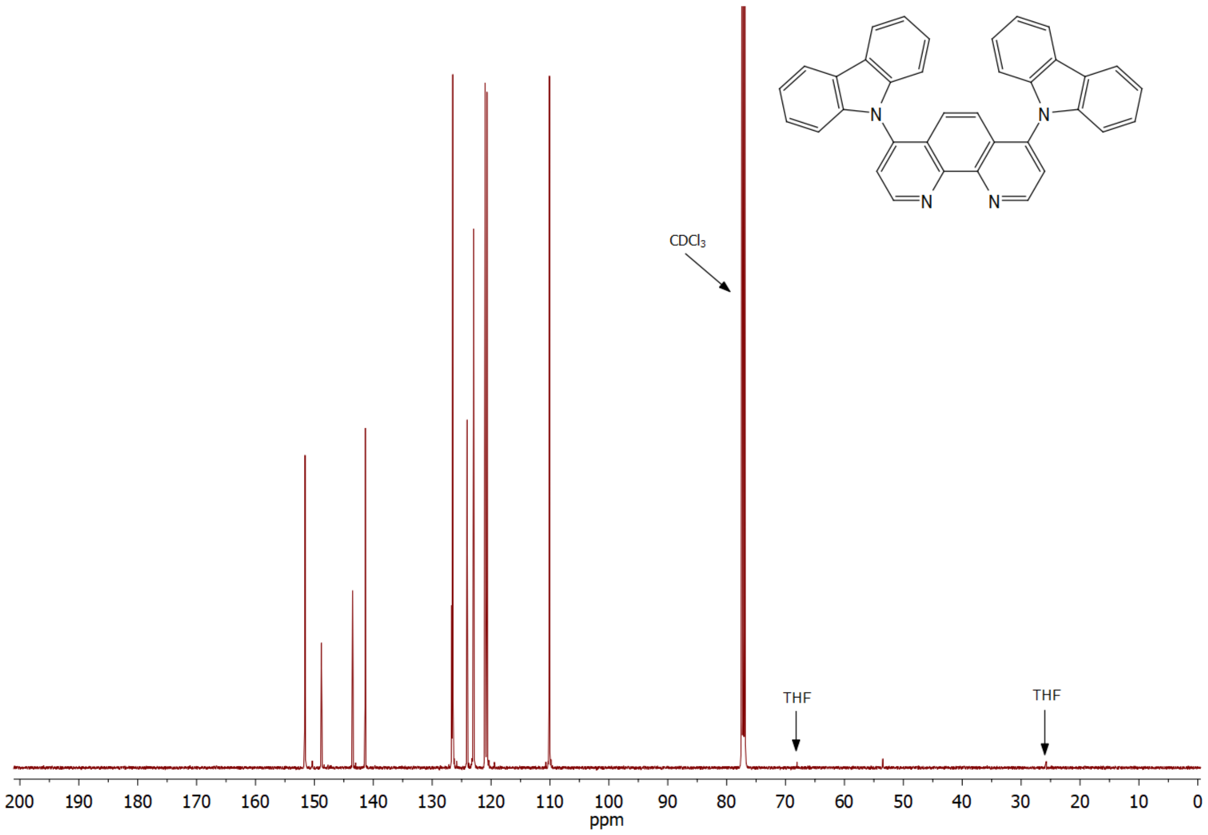

Fig. S19b. ^{13}C{^{1}H} NMR ($CDCl_3$; 125.8 MHz) spectrum of **5f**.

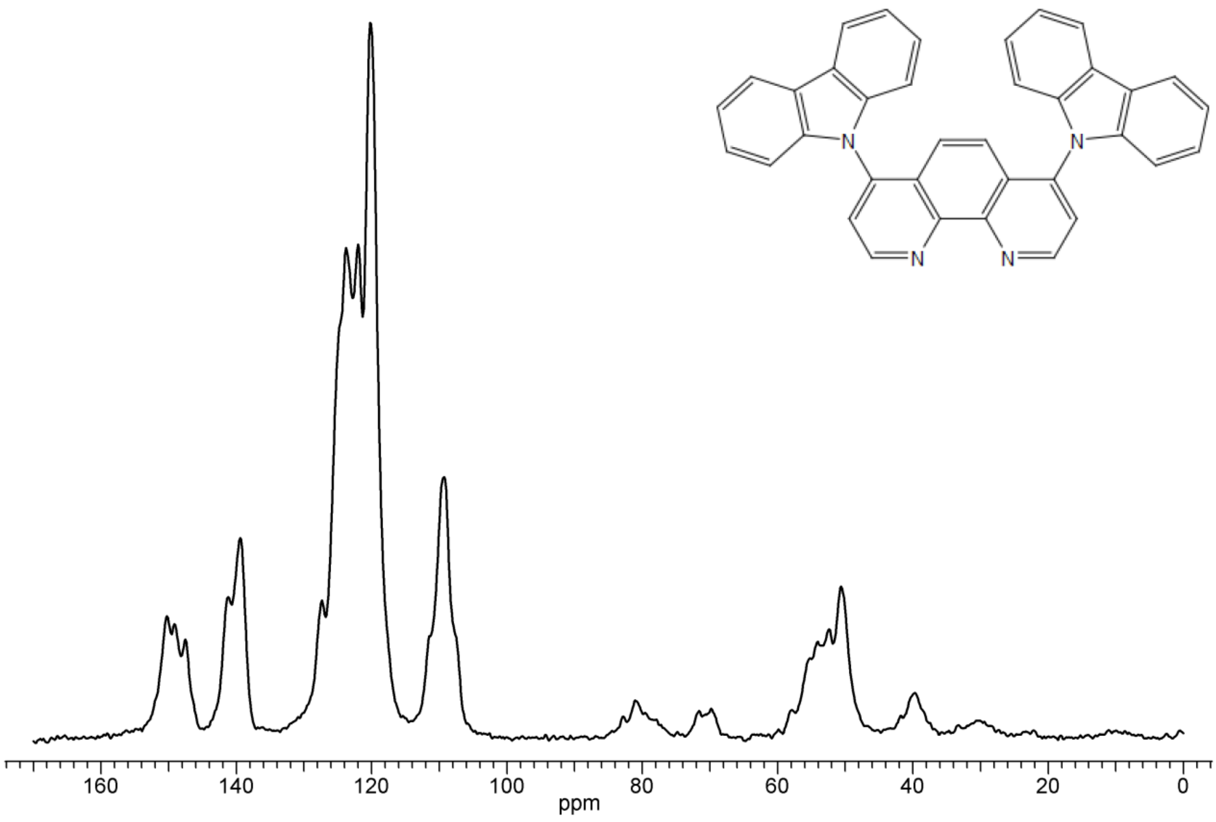

Fig. S19c. ^{13}C CP/MAS NMR spectrum of **5f**.

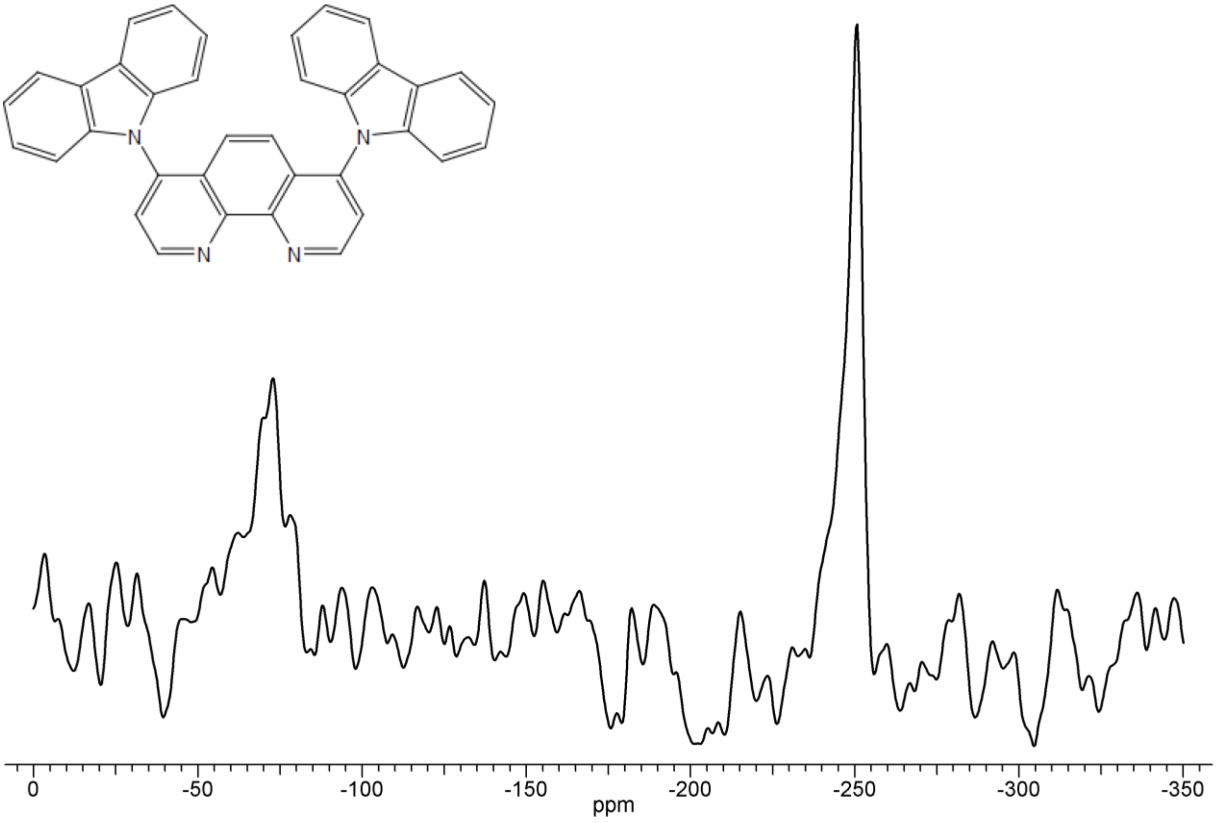

Fig. S19d. ^{15}N CP/MAS NMR spectrum of **5f**.

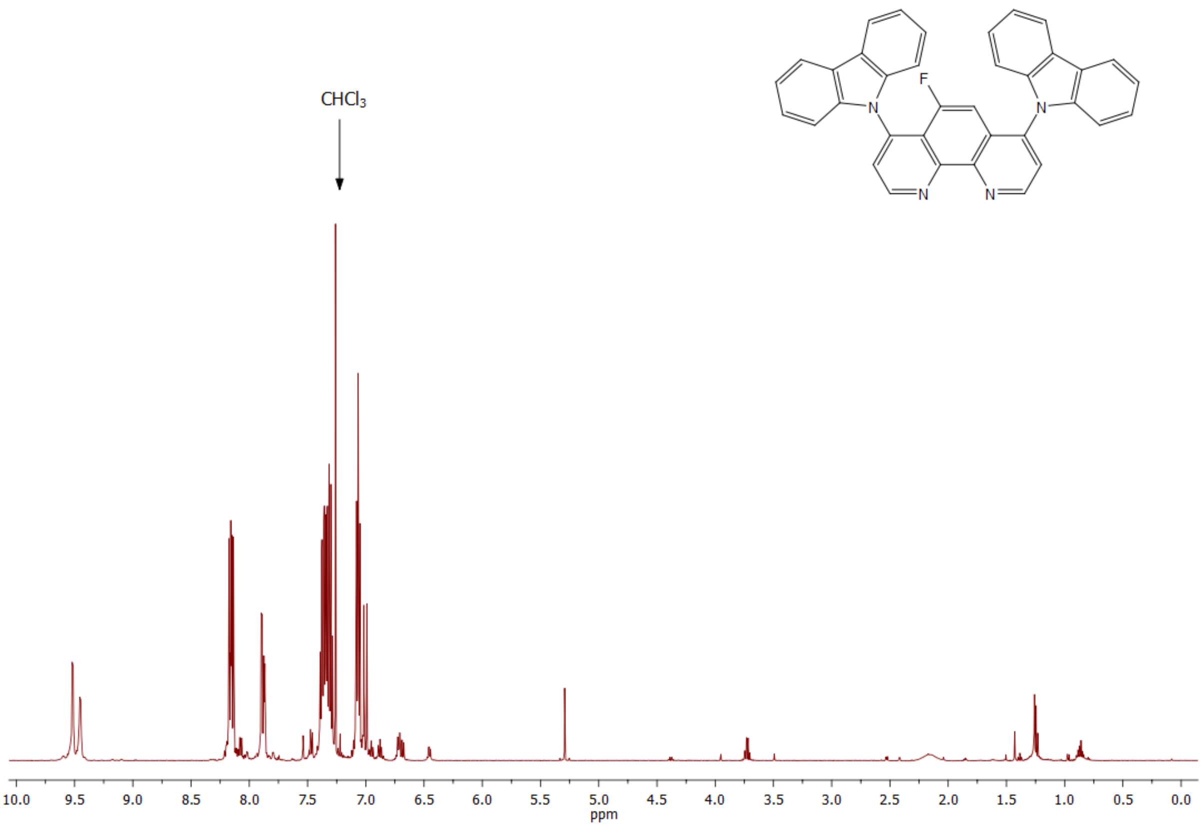

Fig. S20a. ^{1}H NMR ($CDCl_3$; 400.2 MHz) spectrum of **5g**.

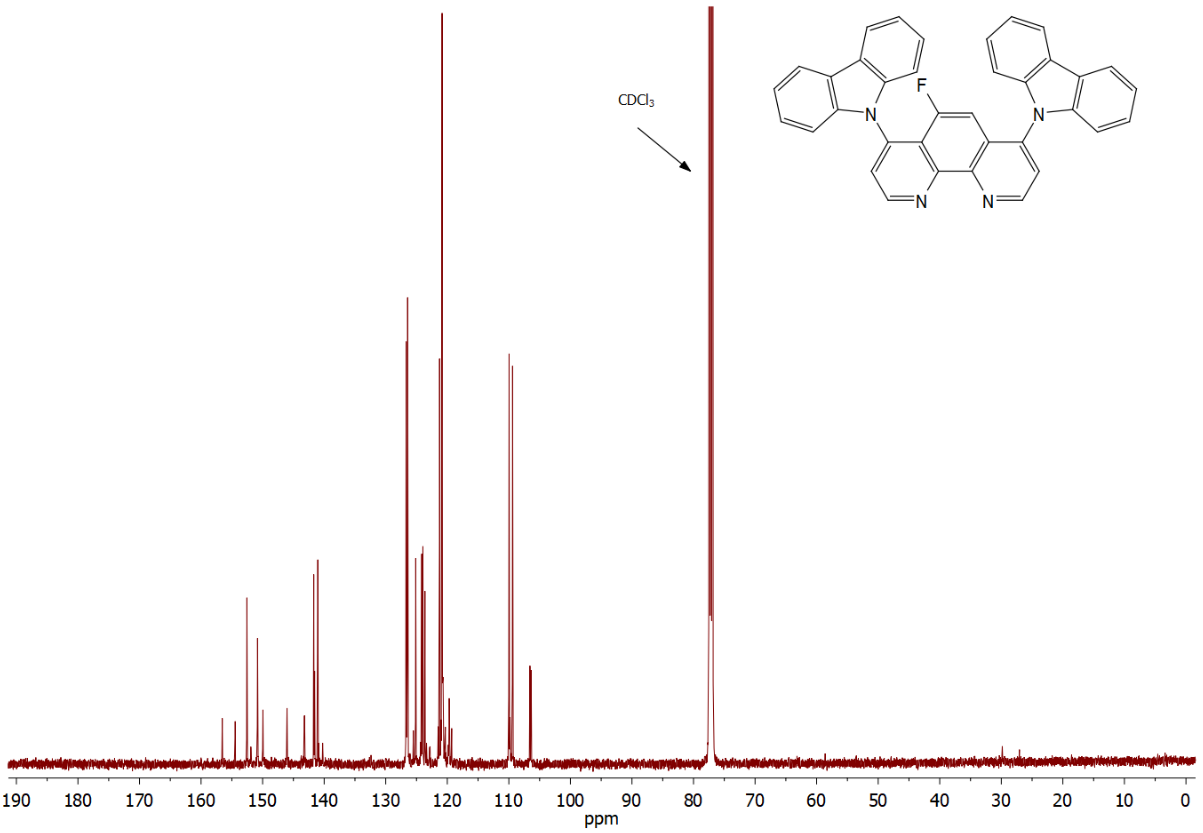

Fig. S20b. $^{13}C\{^{1}H\}$ NMR ($CDCl_3$; 100.5 MHz) spectrum of **5g**.

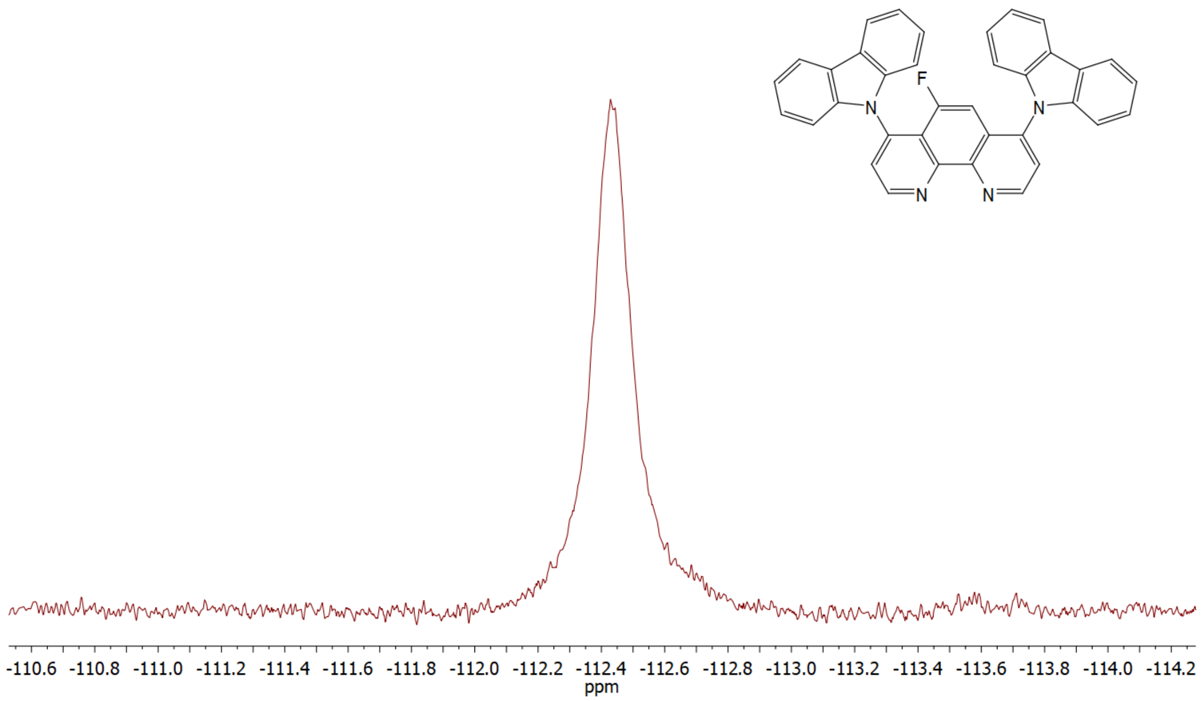

Fig. S20c. ^{19}F NMR ($CDCl_3$; 470.5 MHz) spectrum of **5g**.

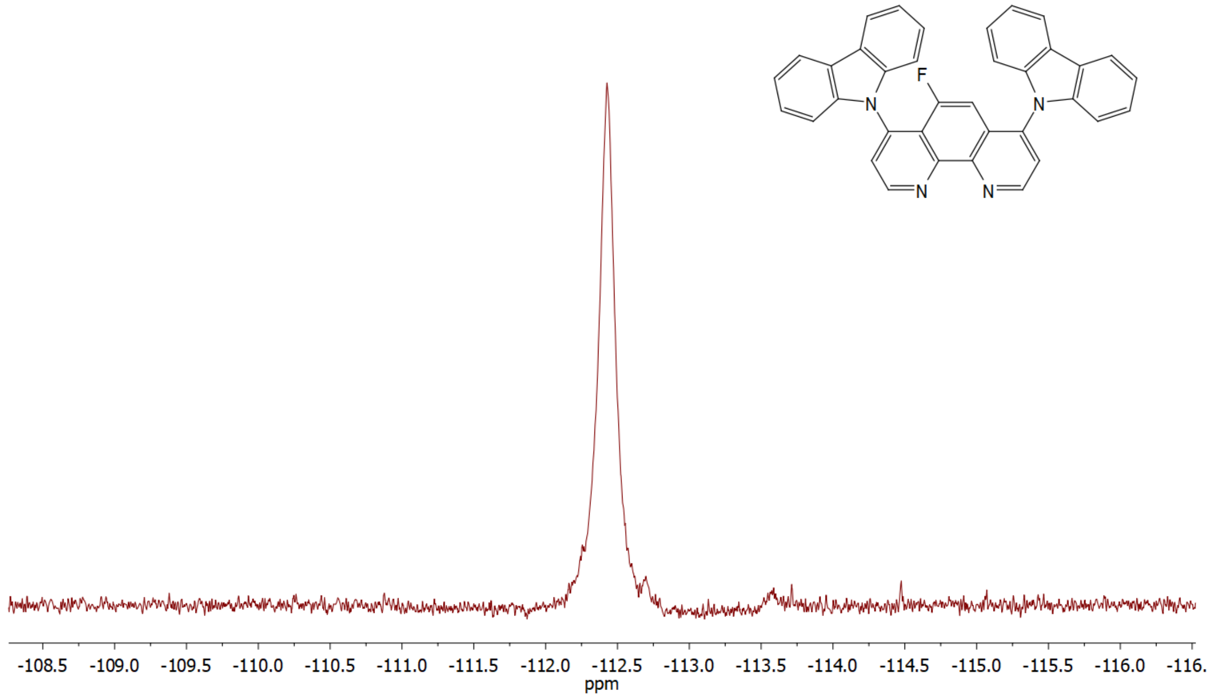

Fig. S20d. ^{19}F{^{1}H} NMR ($CDCl_3$; 470.5 MHz) spectrum of **5g**.

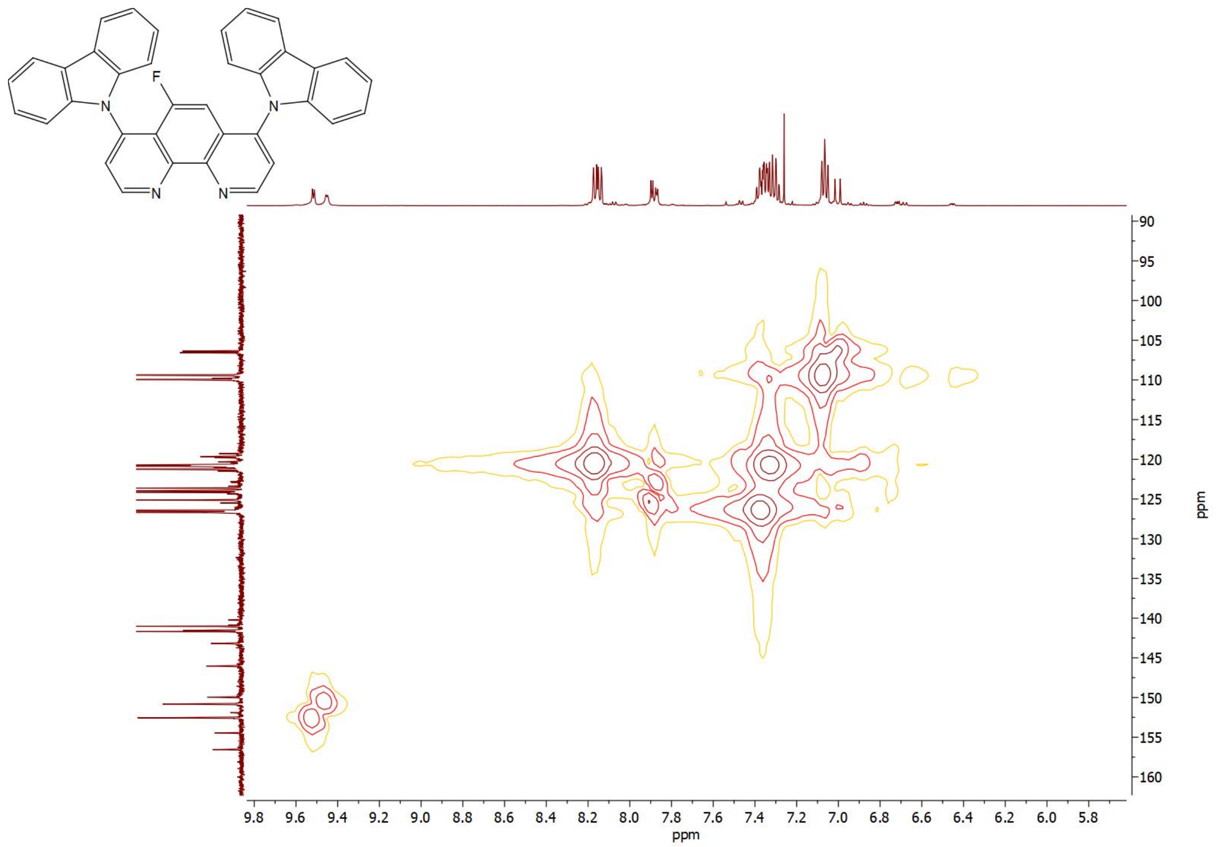

Fig. S20e. ^{1}H, ^{13}C NMR HMQC in $CDCl_3$ spectrum of **5g**.

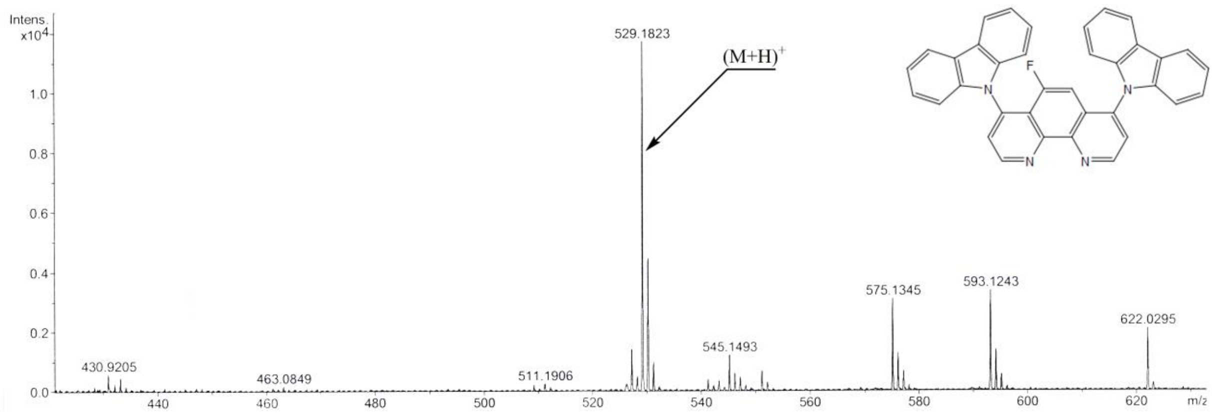

Fig. S20f. MS spectrum of **5g**.

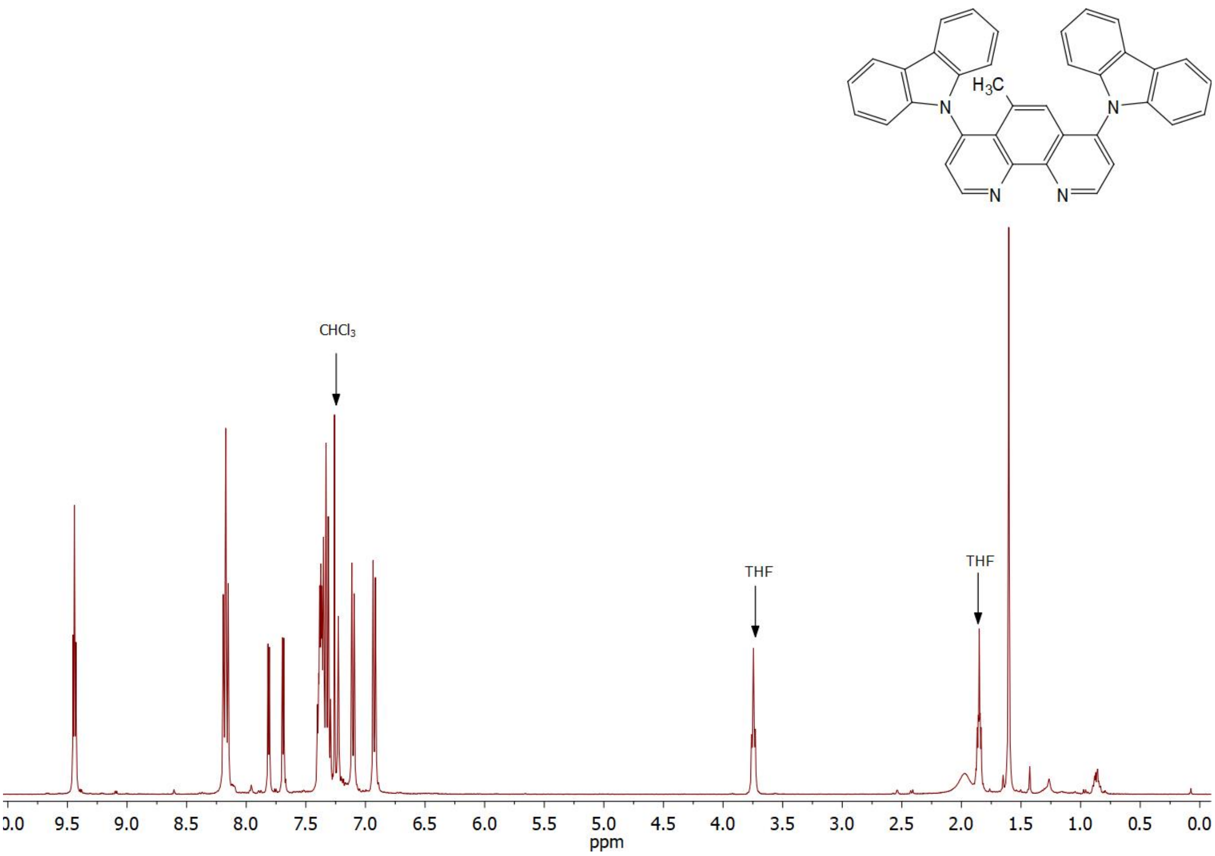

Fig. S21a. ^{1}H NMR ($CDCl_3$; 500.2 MHz) spectrum of **5h**.

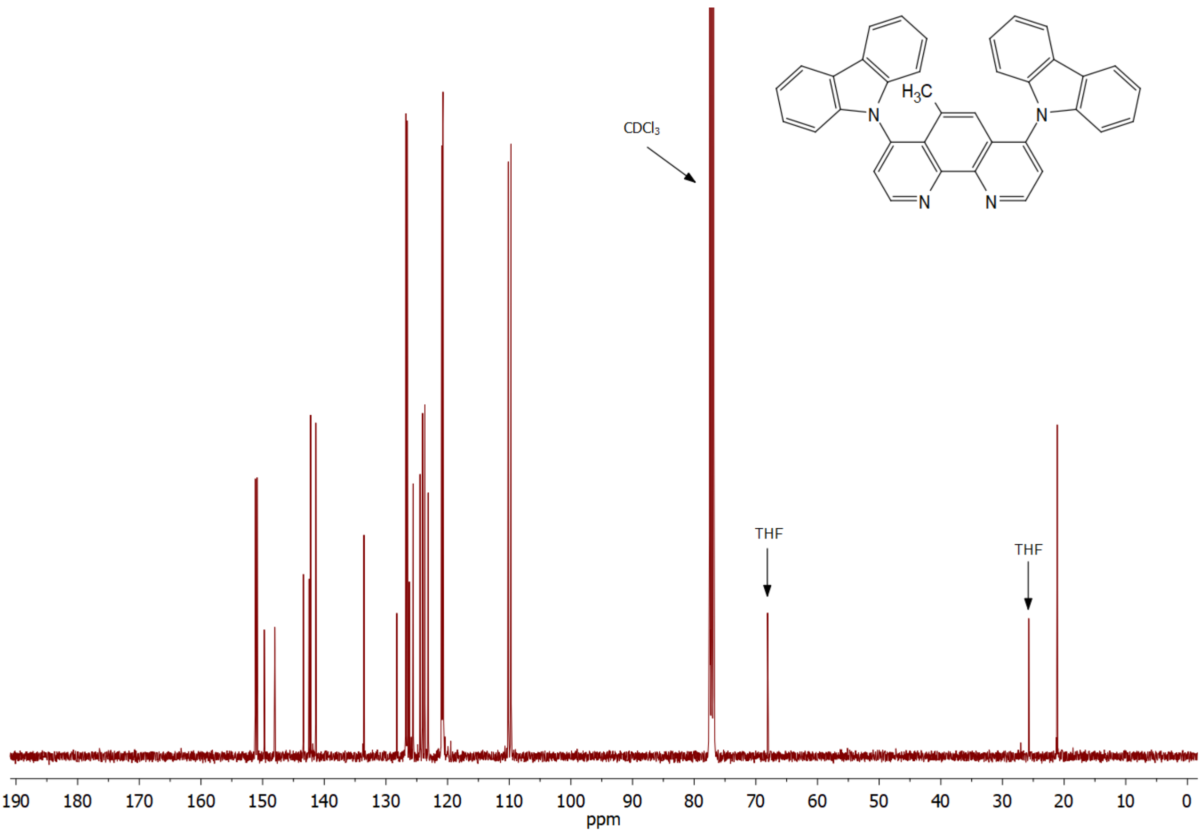

Fig. S21b. ^{13}C{^{1}H} NMR ($CDCl_3$; 100.5 MHz) spectrum of **5h**.

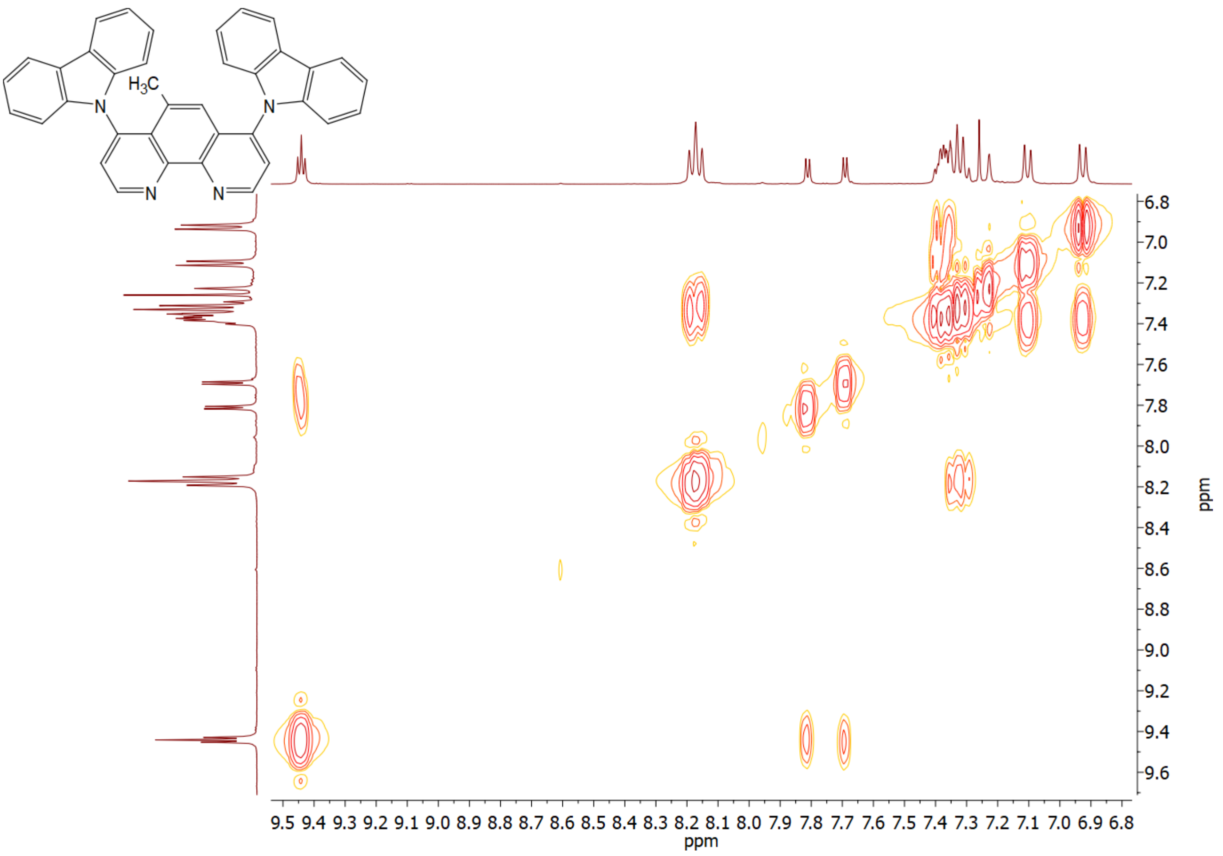

Fig. S21c. 2D-COSY NMR in $CDCl_3$ spectrum of **5h**.

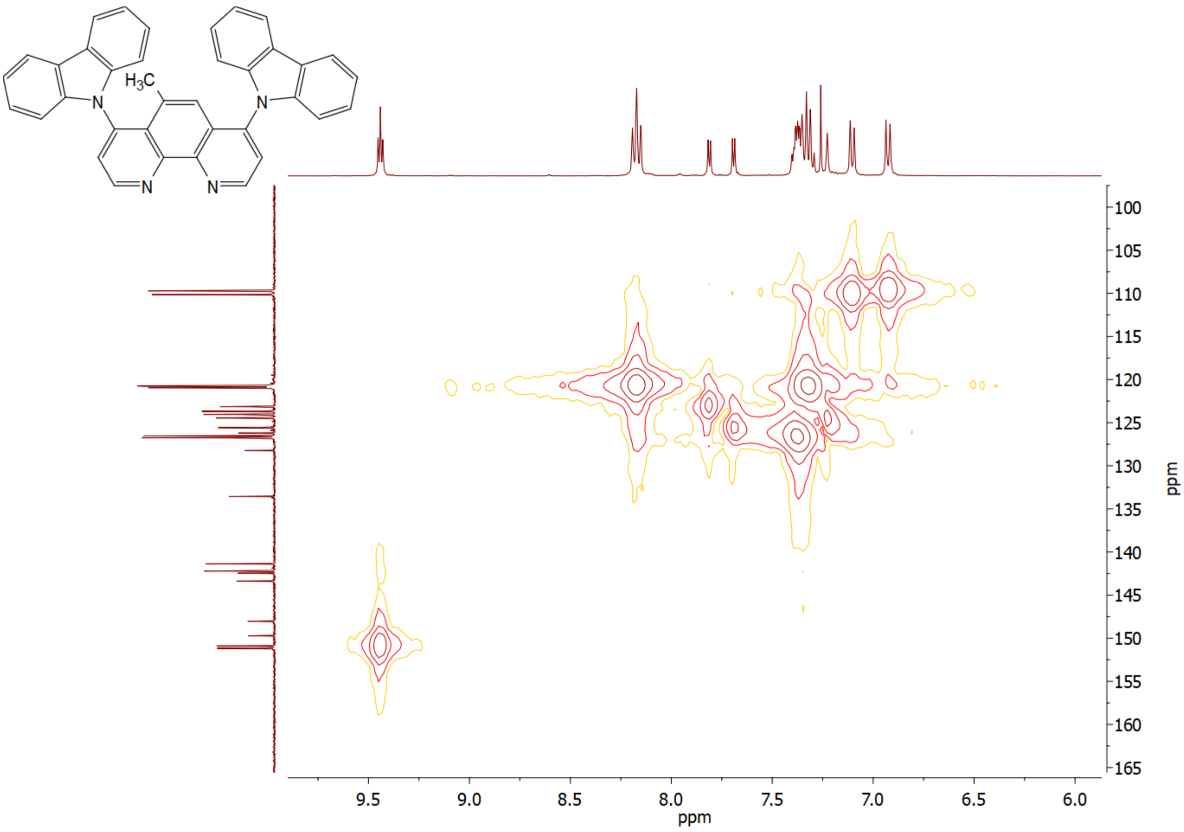

Fig. S21d. ^{1}H, ^{13}C NMR HMQC in $CDCl_3$ spectrum of **5h**.

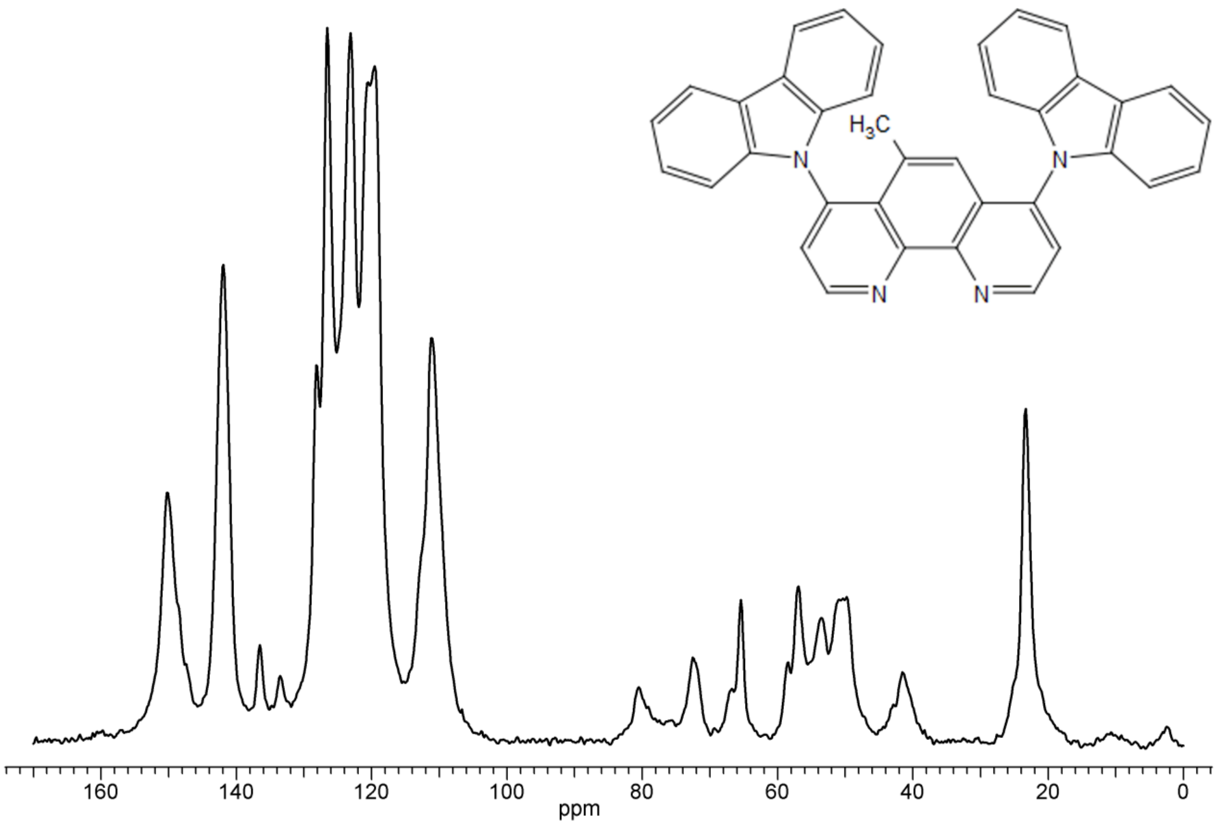

Fig. S21e. ^{13}C CP/MAS NMR spectrum of **5h.**

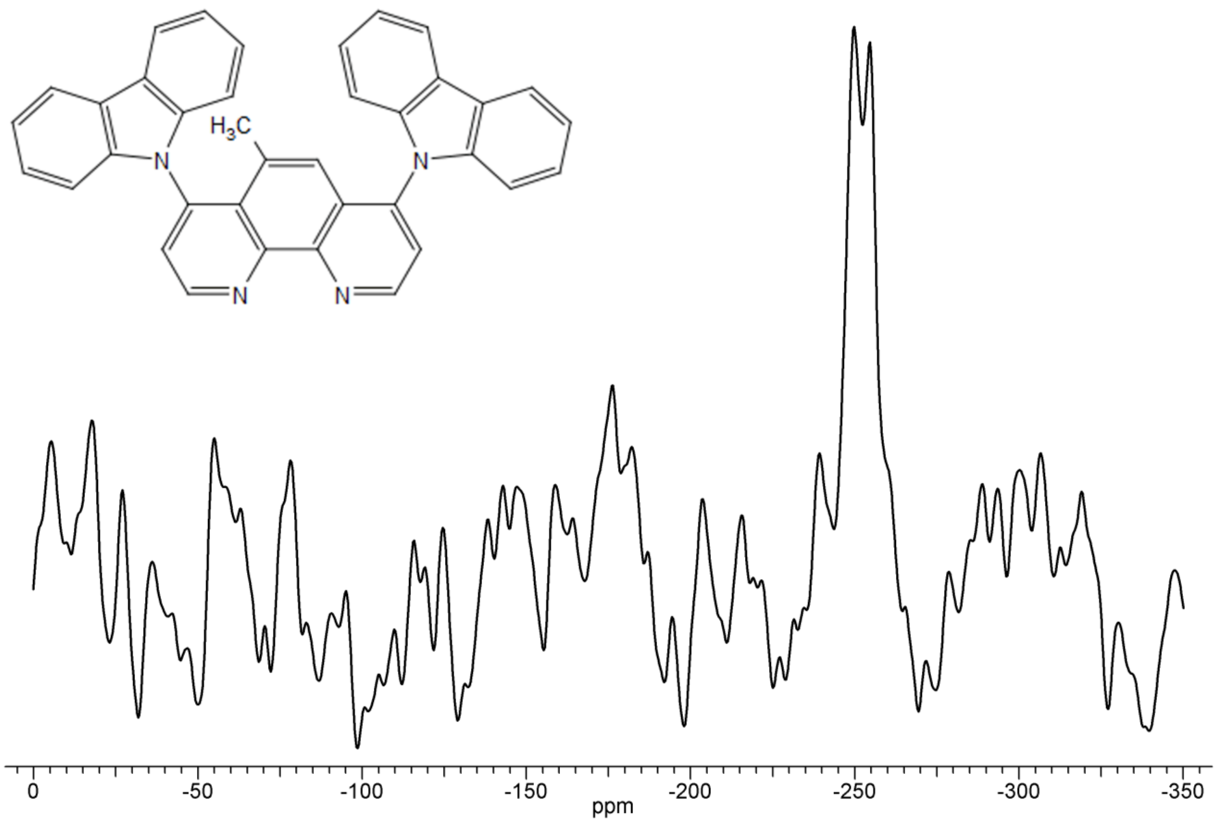

Fig. S21f. ^{15}N CP/MAS NMR spectrum of **5h.**

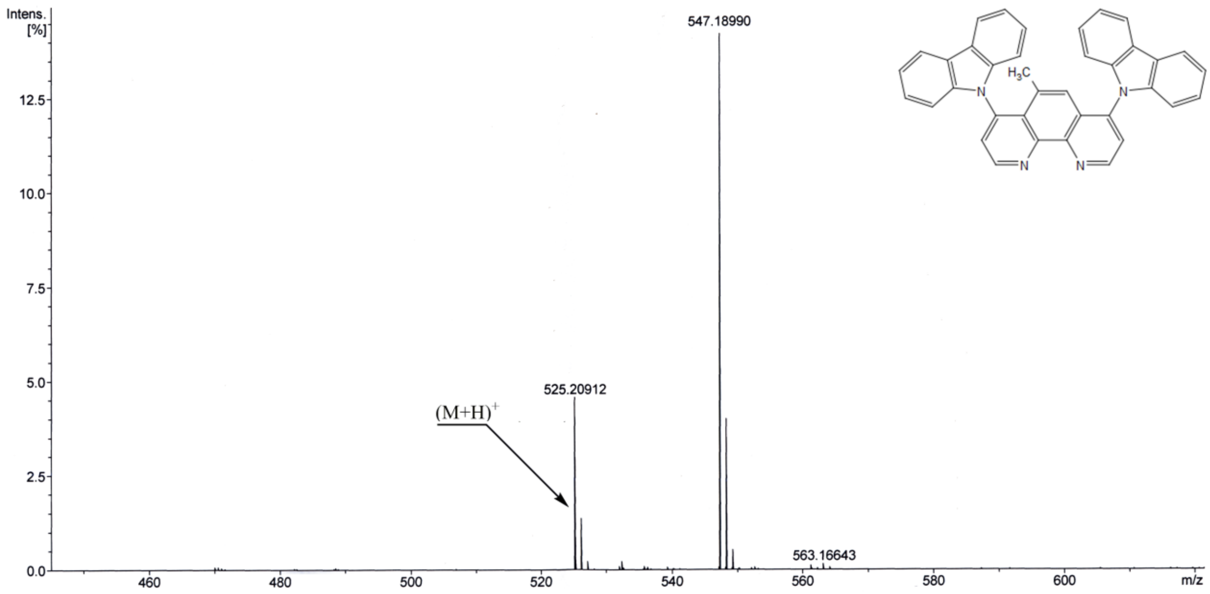

Fig. S21g. MS spectrum of **5h**.

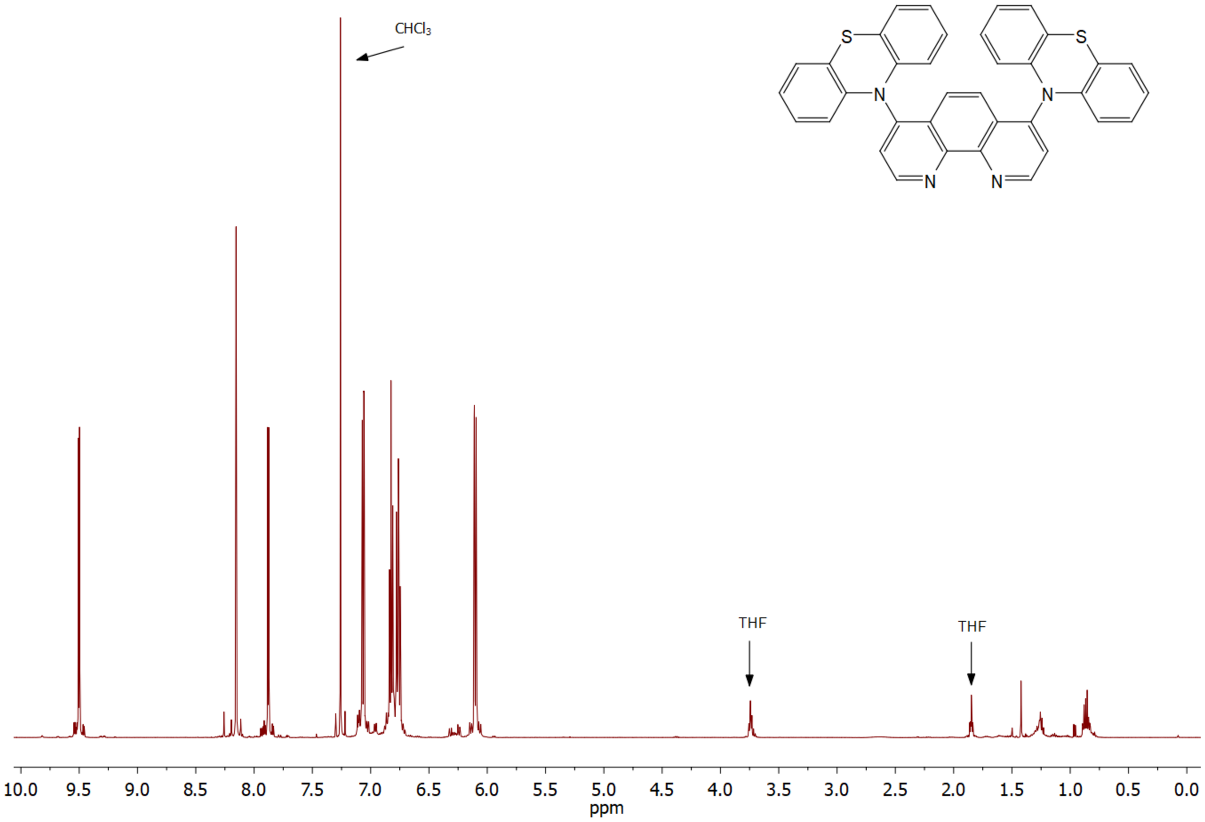

Fig. S22a. ^{1}H NMR ($CDCl_3$; 500.2 MHz) and spectrum of **5i**.

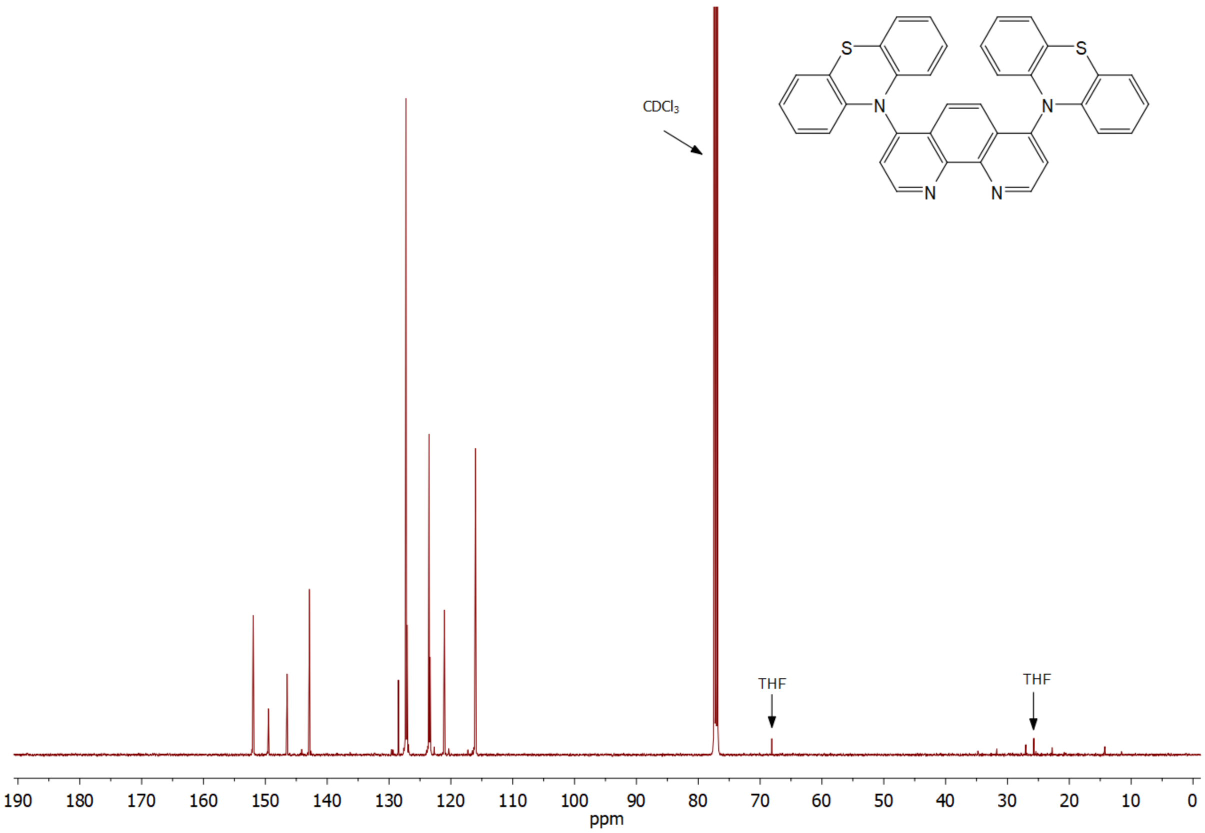

Fig. S22b. $^{13}C\{^{1}H\}$ NMR ($CDCl_3$; 100.5 MHz) spectrum of **5i**.

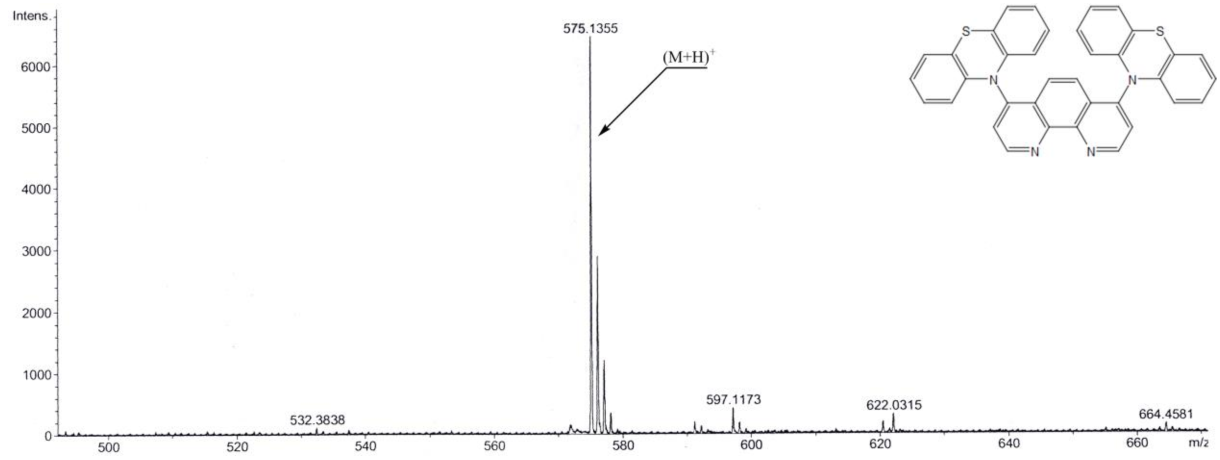

Fig. S22c. MS spectrum of **5i**.

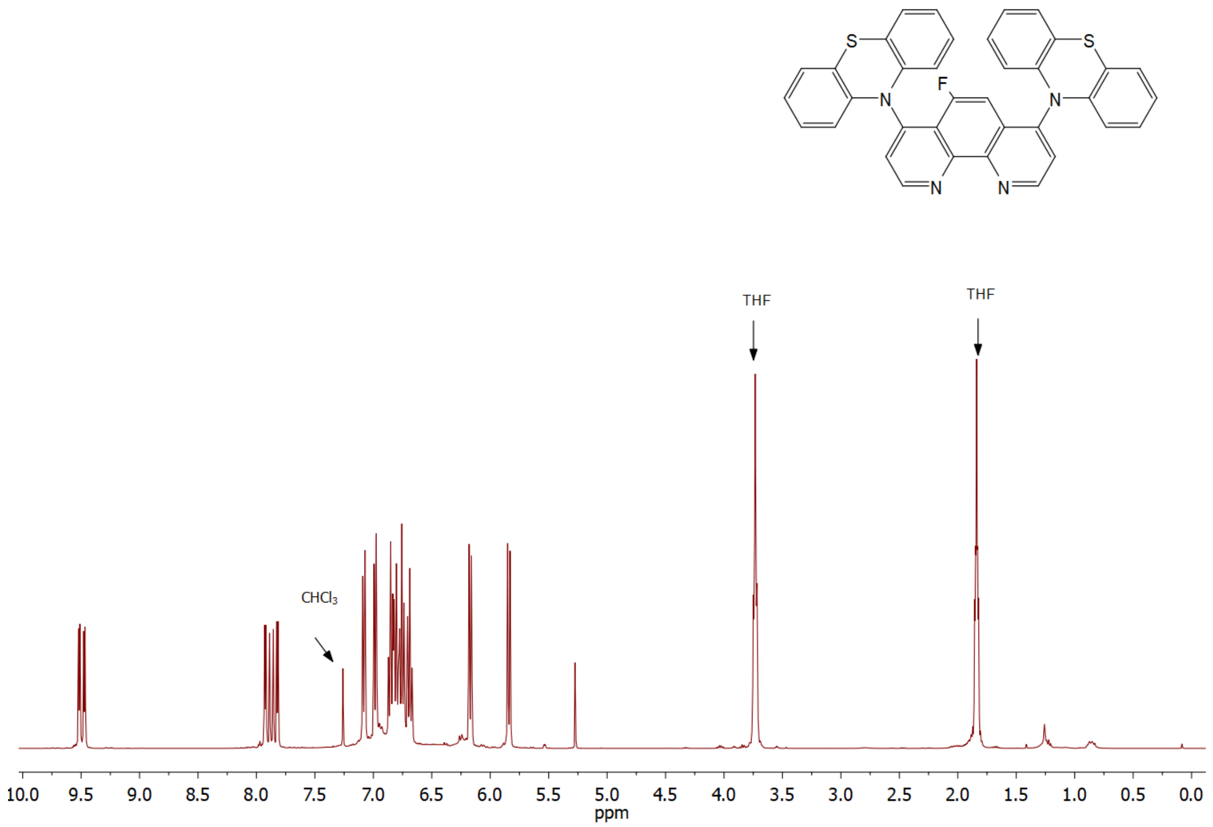

Fig. S23a. ^{1}H NMR ($CDCl_3$; 500.2 MHz) spectrum of **5j**.

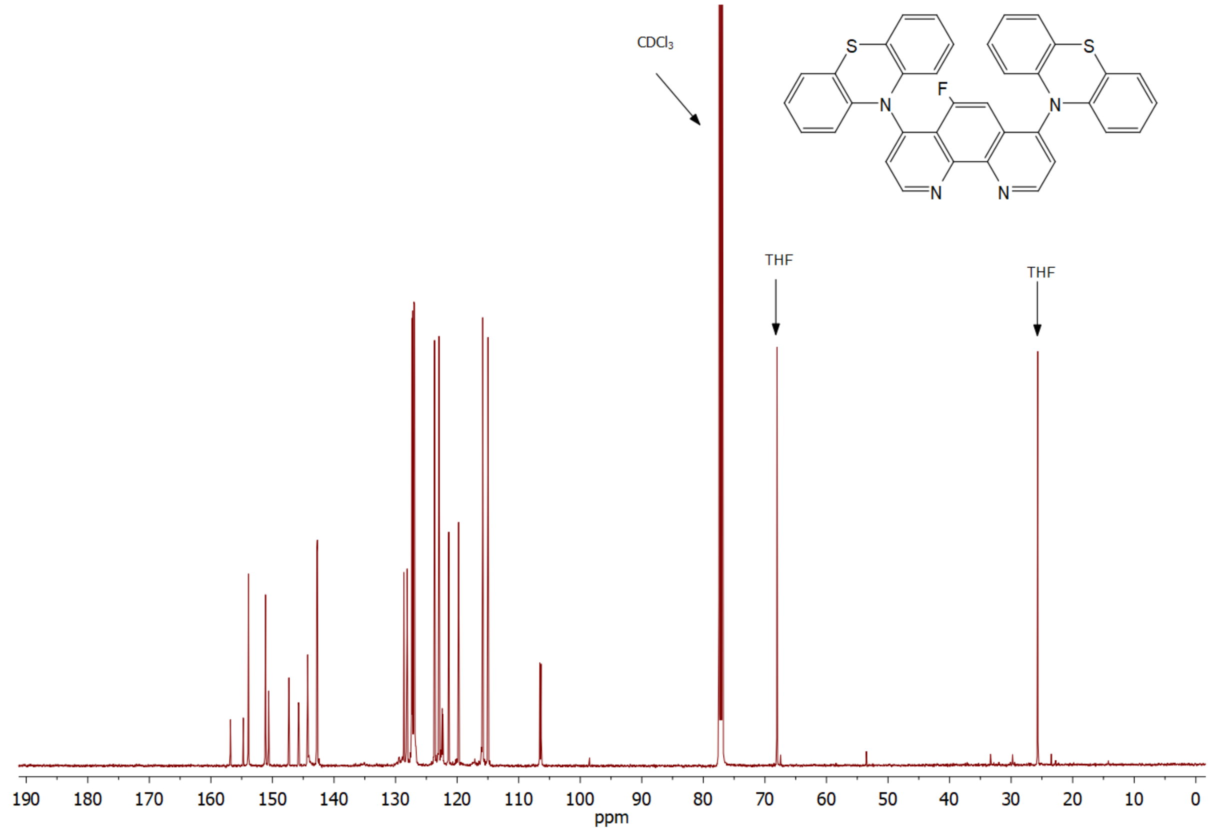

Fig. S23b. $^{13}C\{^{1}H\}$ NMR ($CDCl_3$; 125.8 MHz) spectrum of **5j**.

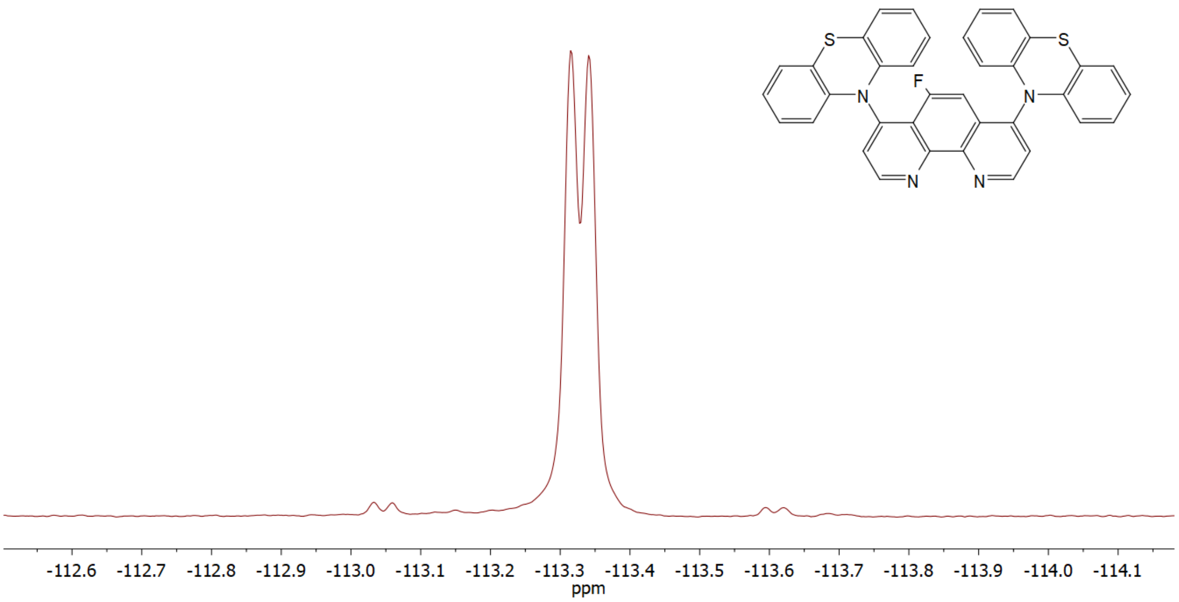

Fig. S23c. ^{19}F NMR ($CDCl_3$; 470.5 MHz) spectrum of **5j**.

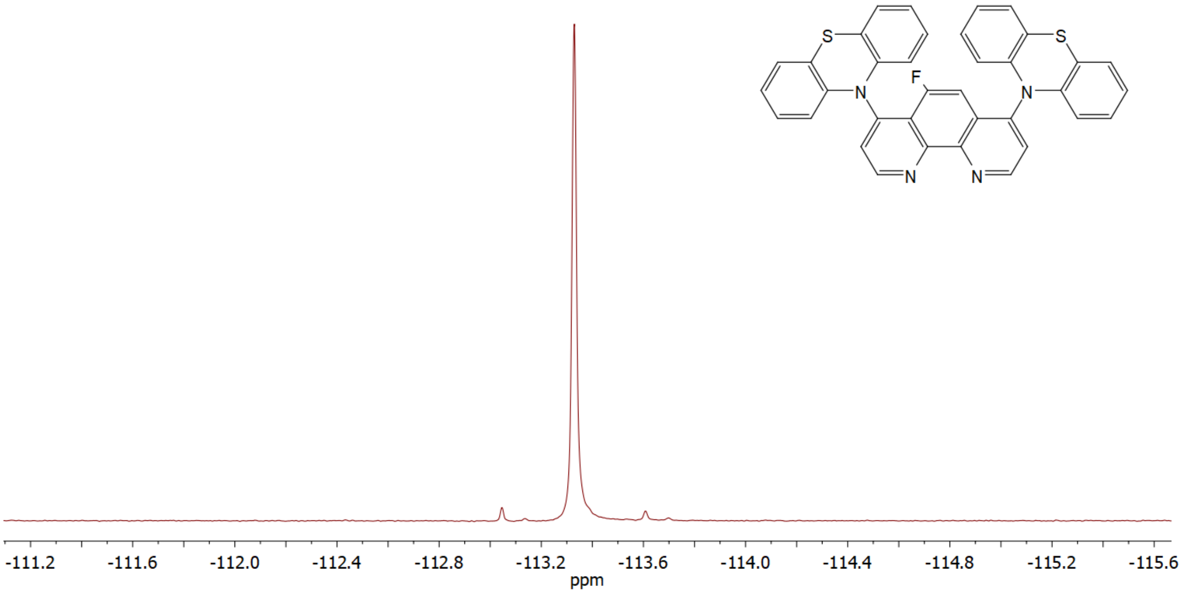

Fig. S23d. ^{19}F{^{1}H} NMR ($CDCl_3$; 470.5 MHz) spectrum of **5j**.

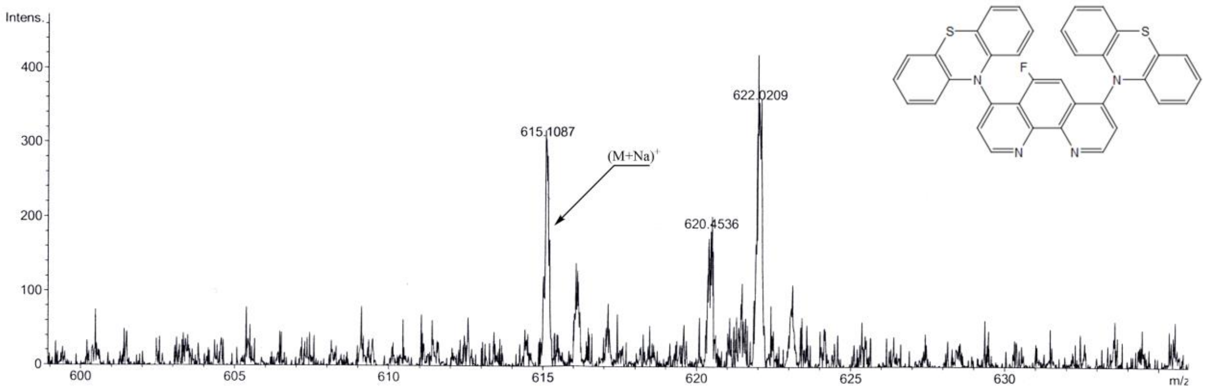

Fig. S23e. MS spectrum of **5j**.

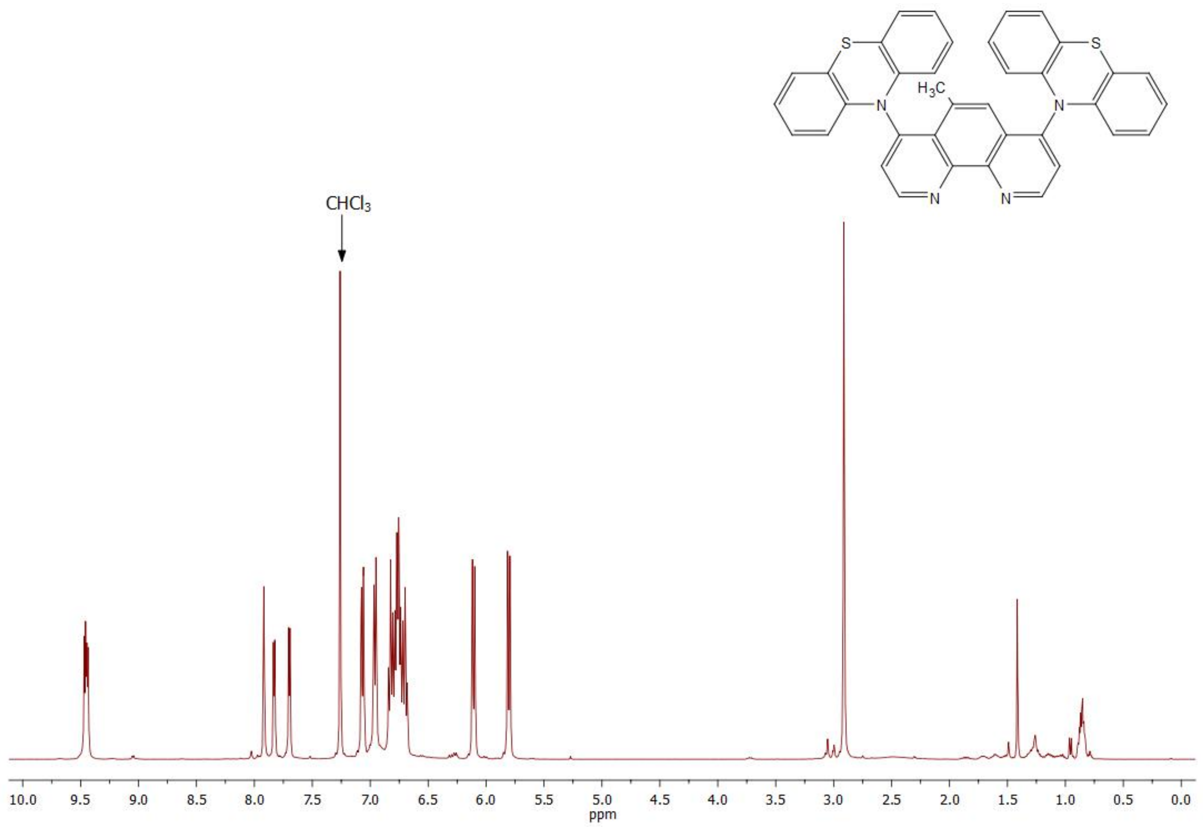

Fig. S24a. ^{1}H NMR ($CDCl_3$; 500.2 MHz) spectrum of **5k**.

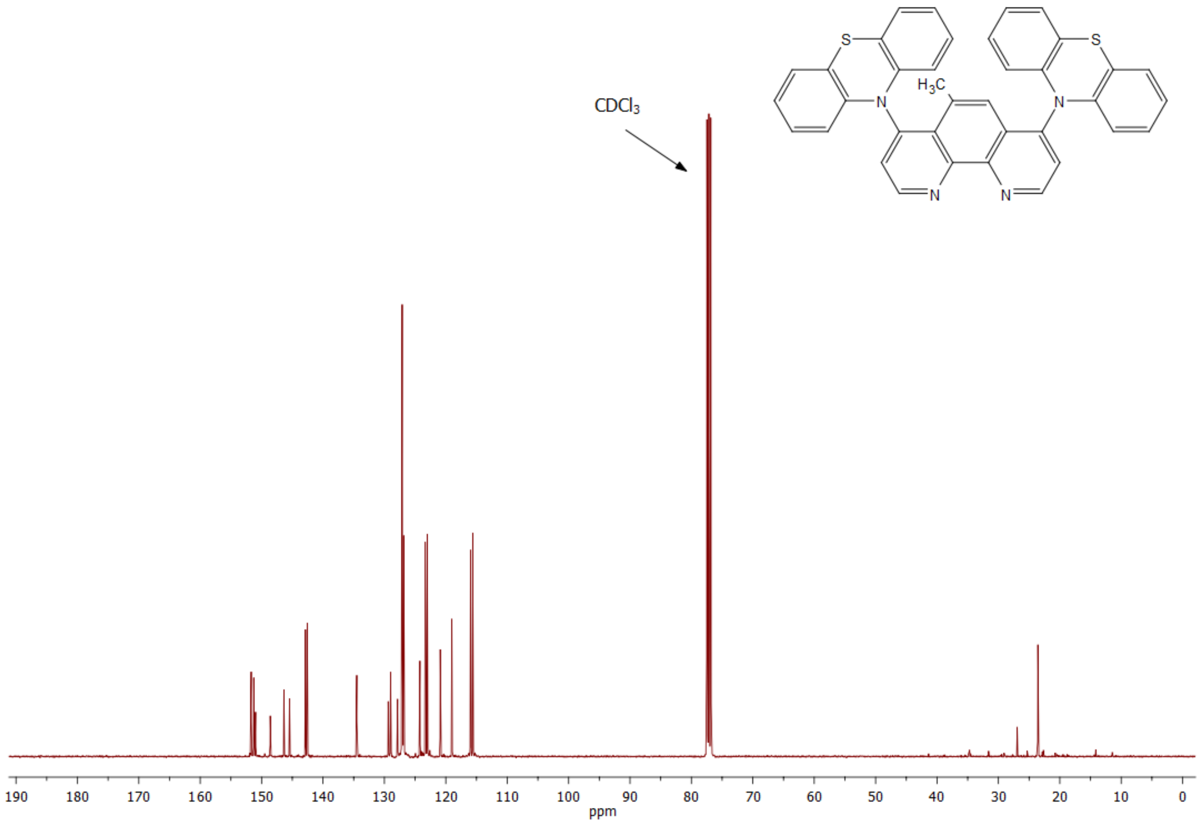

Fig. S24b. $^{13}C\{^{1}H\}$ NMR ($CDCl_3$; 100.5 MHz) spectrum of **5k**.

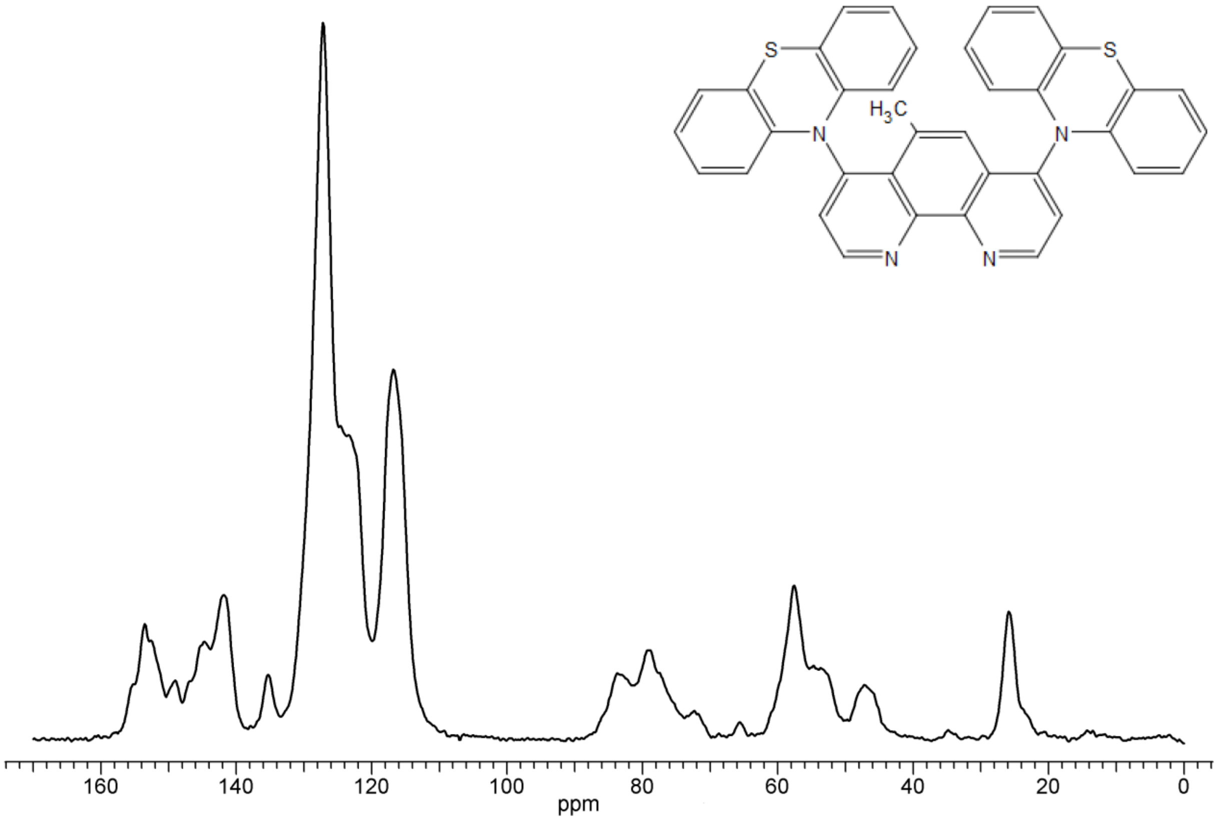

Fig. S24c. ^{13}C CP/MAS NMR spectrum of **5k**.

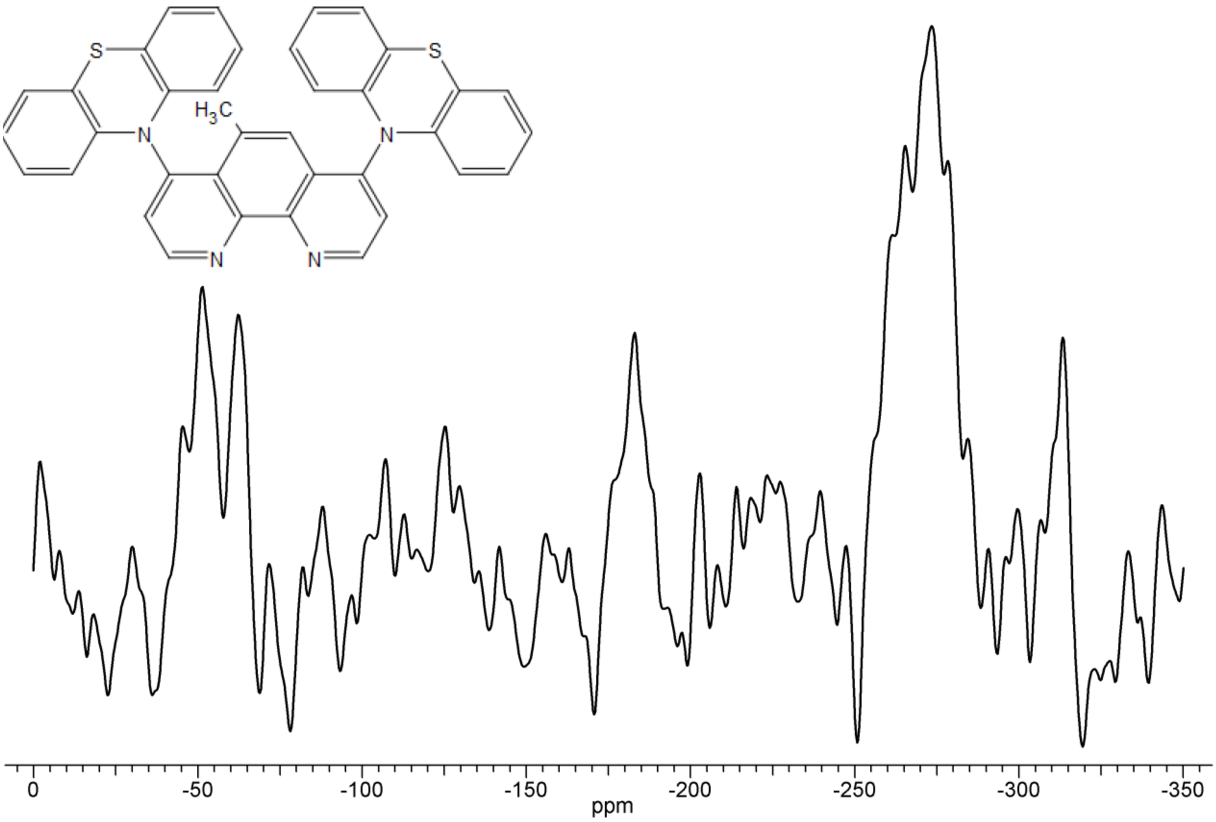

Fig. S24d. ^{15}N CP/MAS NMR spectrum of **5k**.

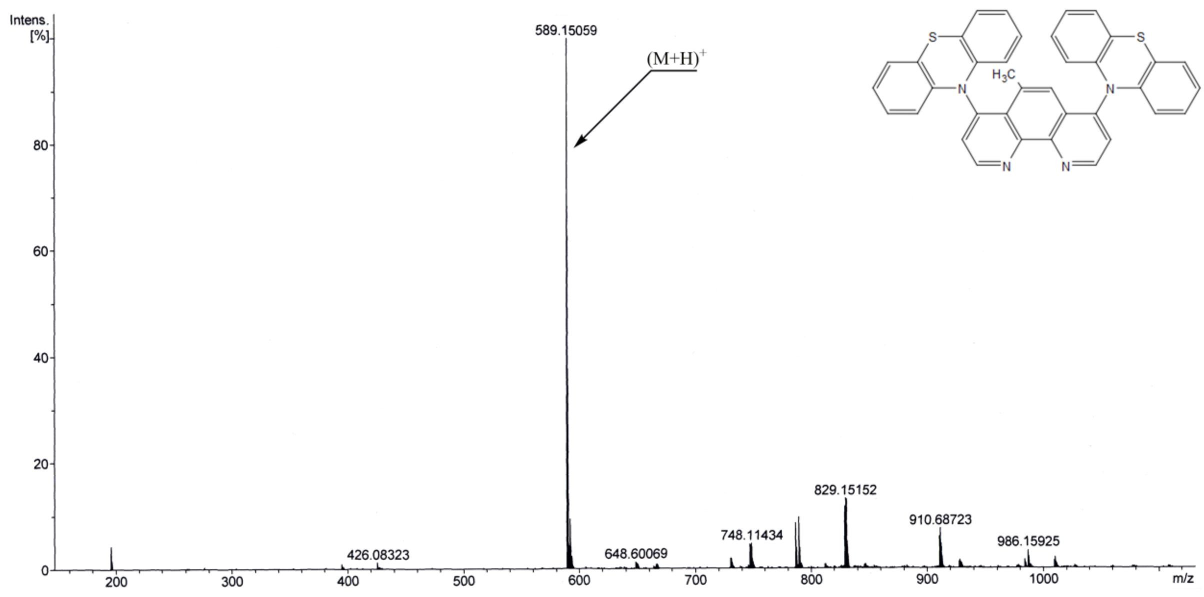

Fig. S24e. MS spectrum of **5k**.

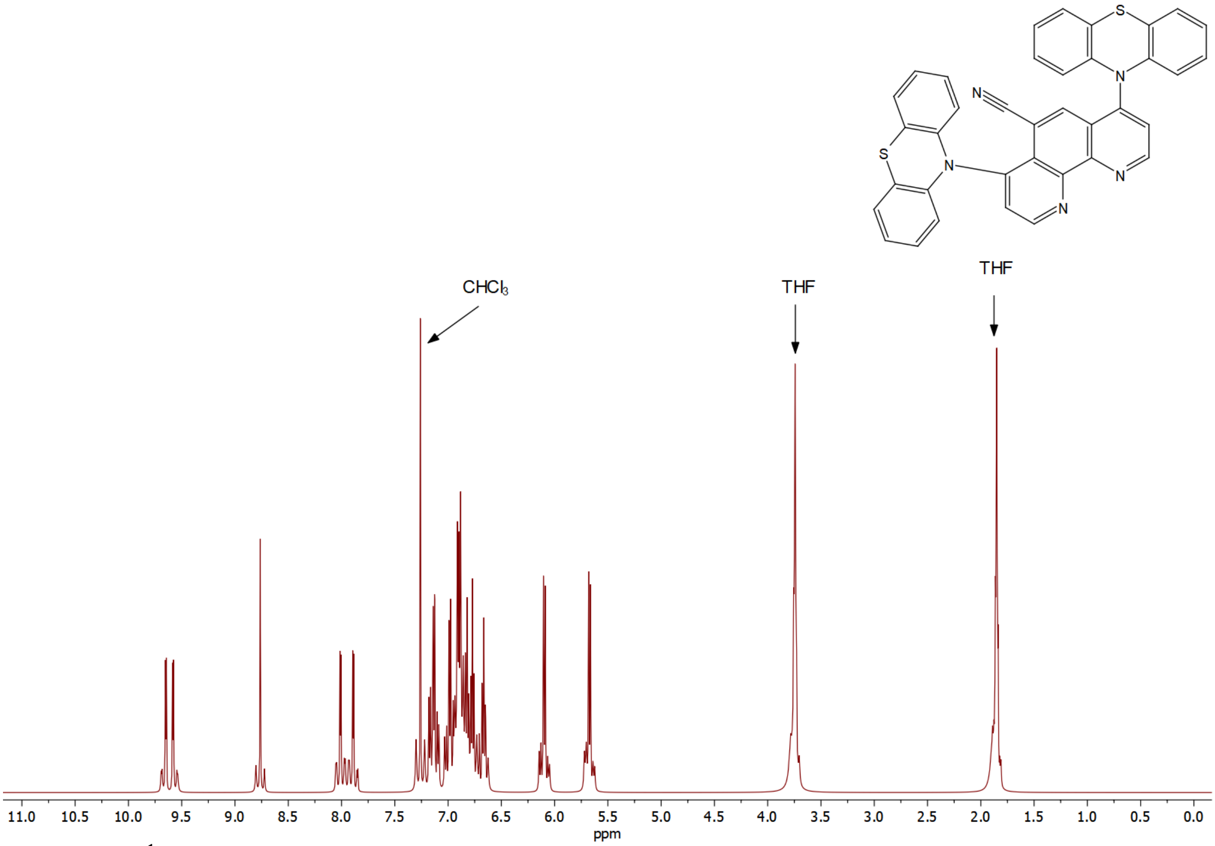

Fig. S25a. ^{1}H NMR ($CDCl_3$; 500.2 MHz) spectrum of **5n**.

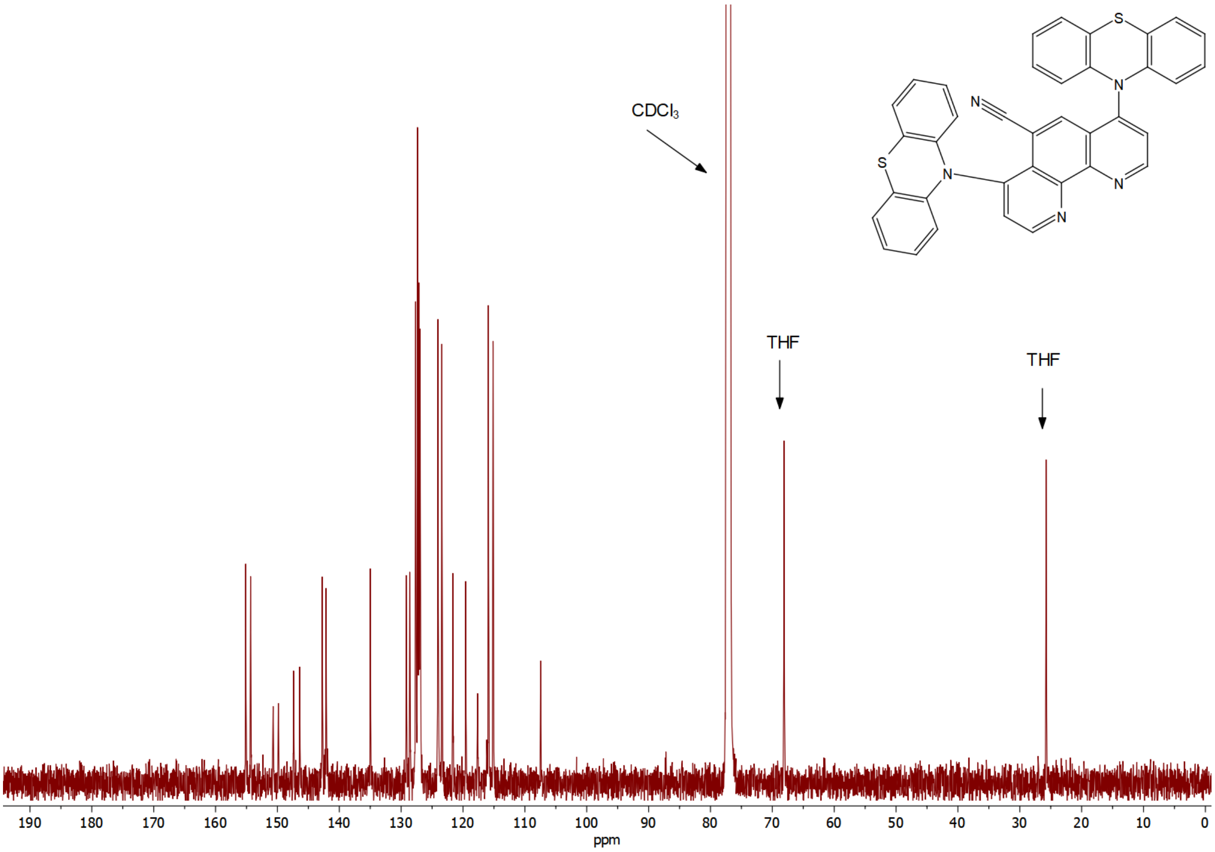

Fig. S25b. $^{13}C\{^1H\}$ NMR ($CDCl_3$; 125.8 MHz) spectrum of **5n**.

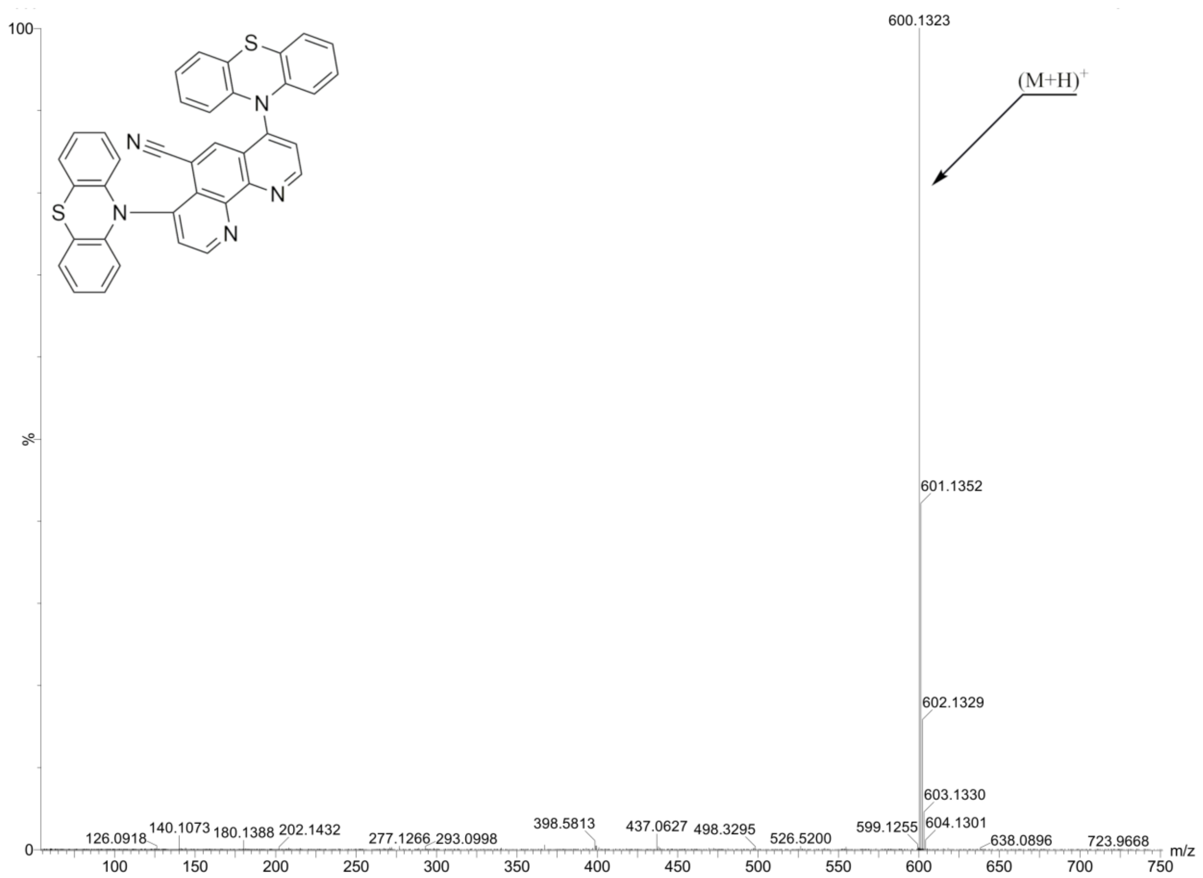

Fig. S25c. MS spectrum of **5n**.

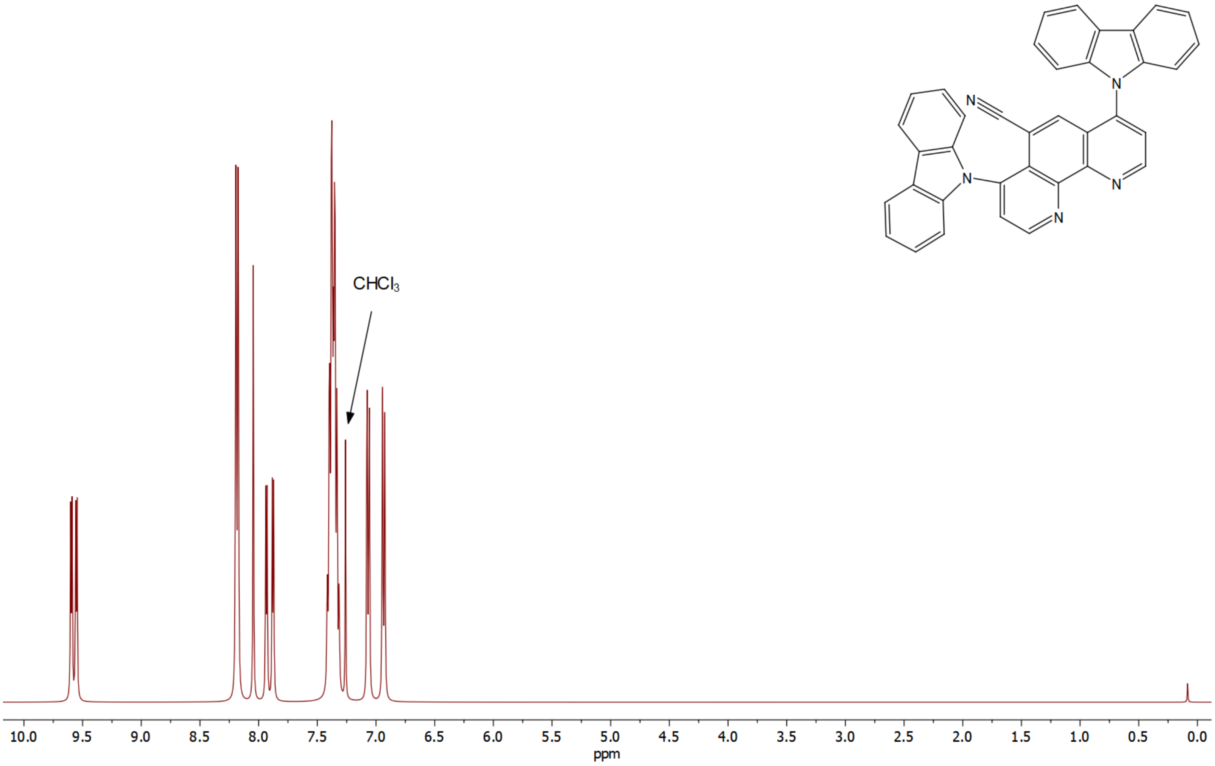

Fig. S26a. ^{1}H NMR ($CDCl_3$; 400.2 MHz) spectrum of **5m**

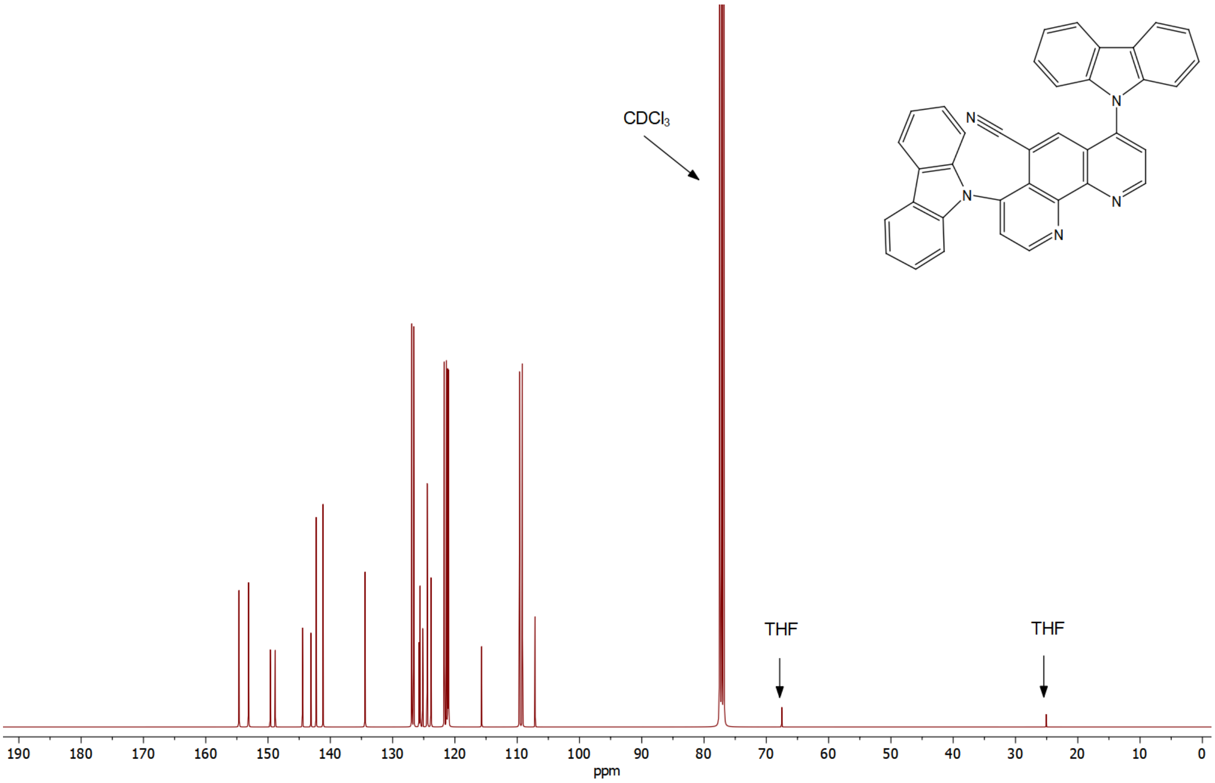

Fig. S26b. $^{13}C\{^{1}H\}$ NMR ($CDCl_3$; 100.6 MHz) spectrum of **5m**.

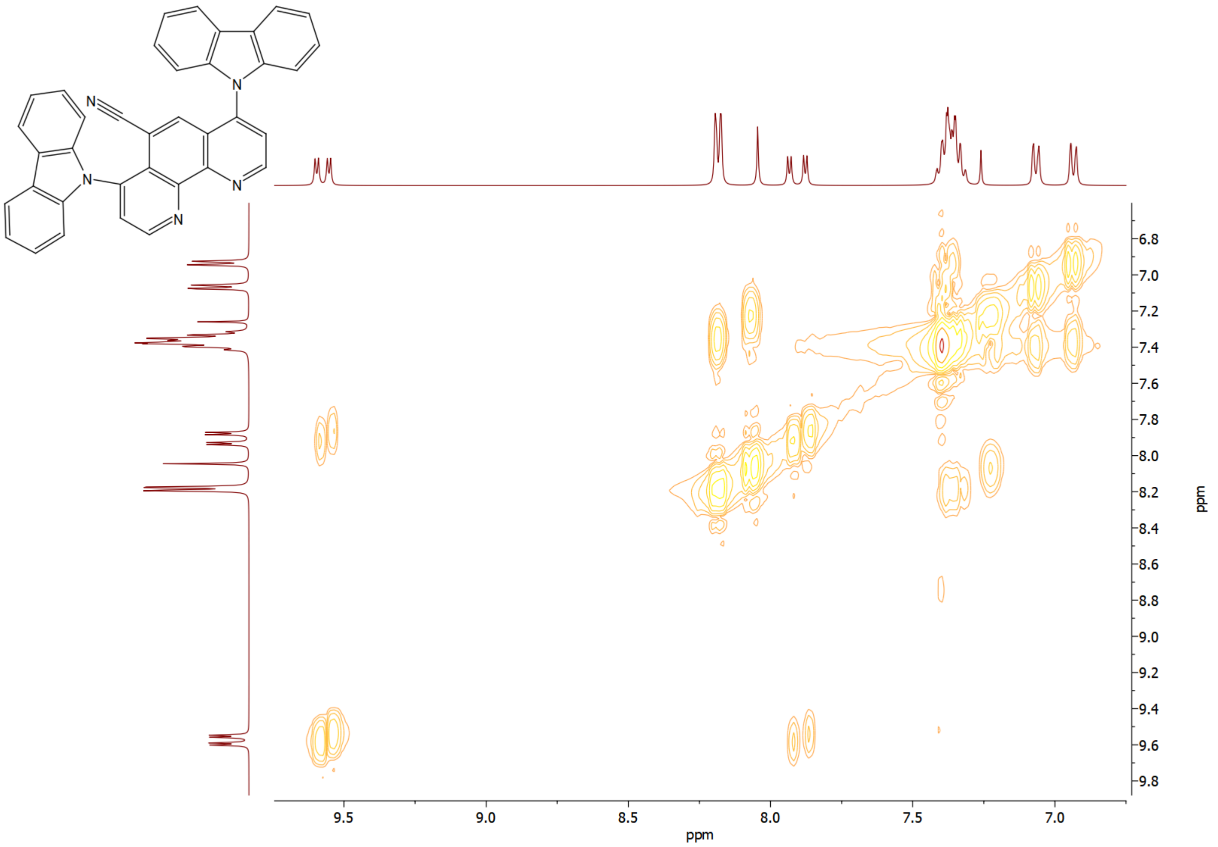

Fig. S26c. 2D-COSY NMR in $CDCl_3$ spectrum of **5m**

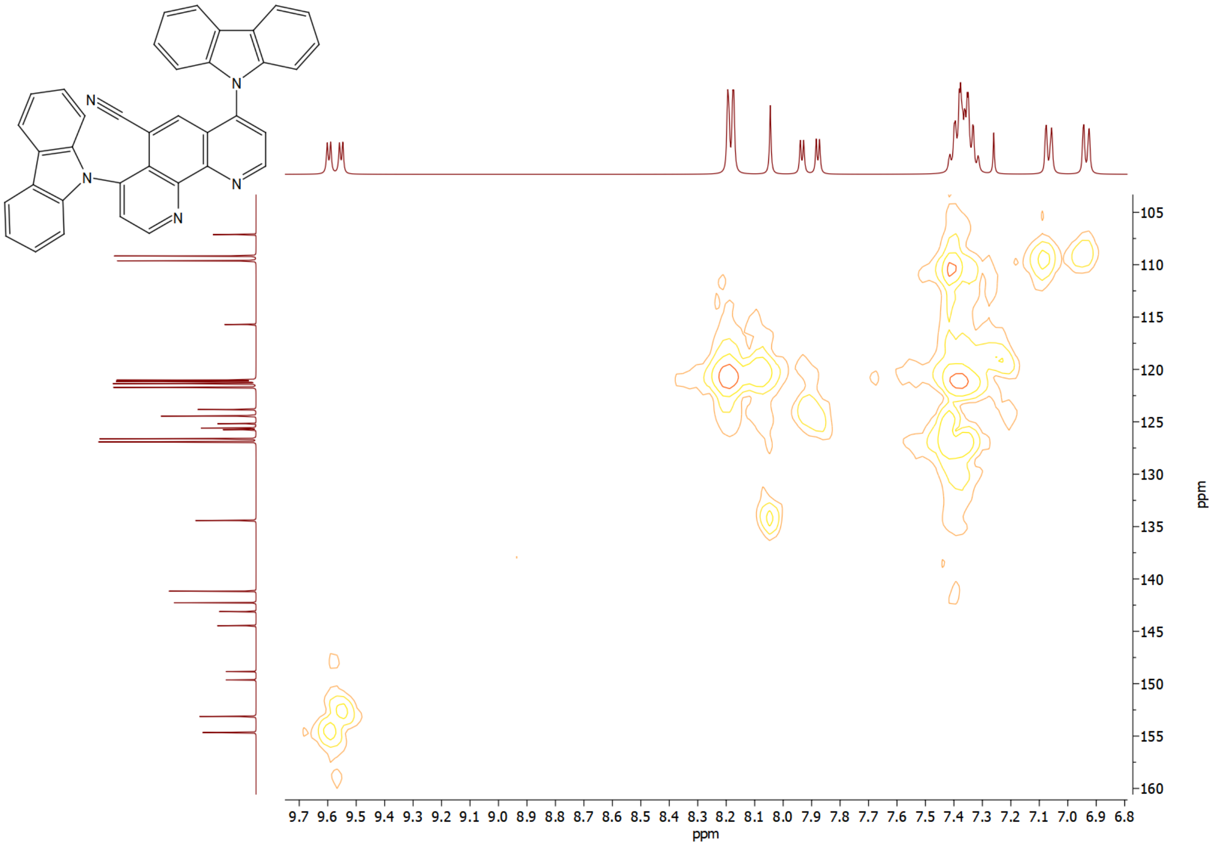

Fig. S26c. ^{1}H, ^{13}C NMR HMQC in $CDCl_3$ spectrum of **5m**.

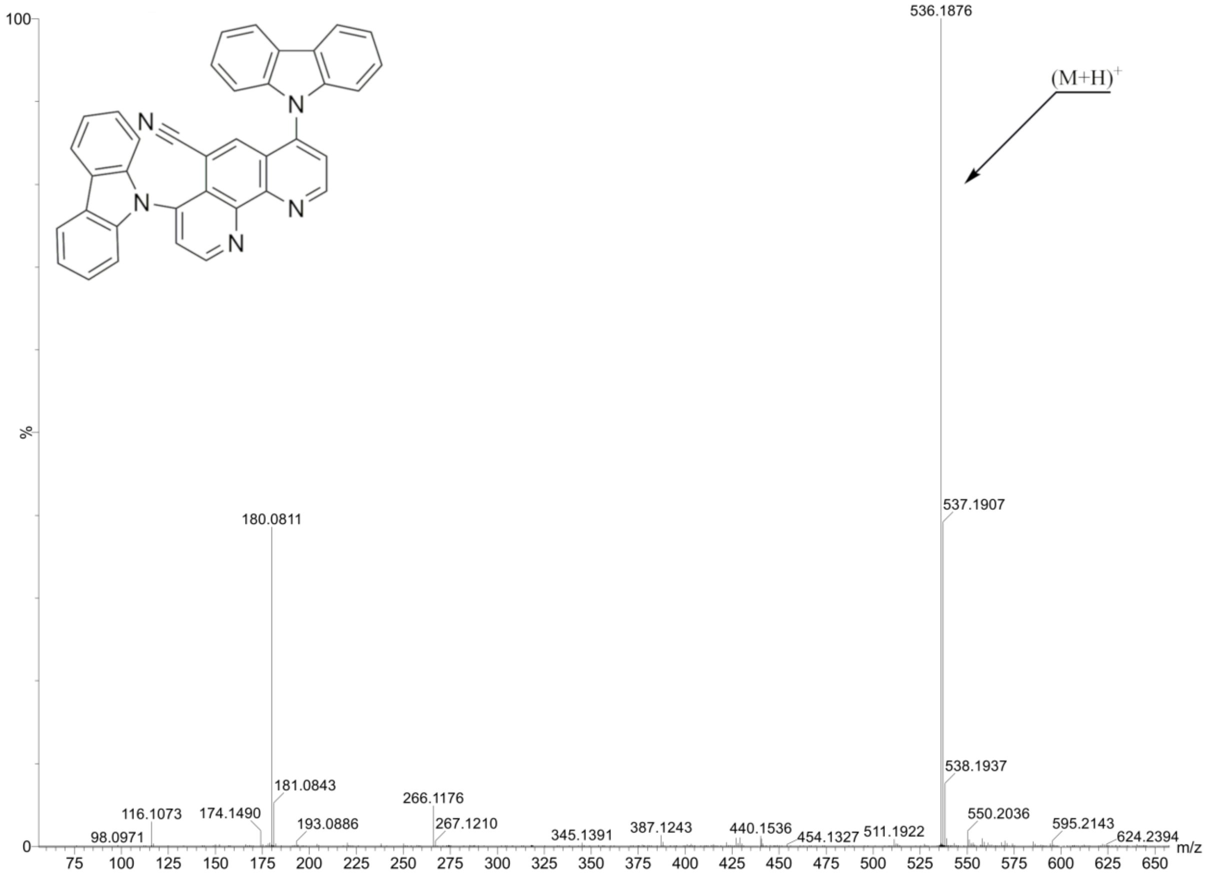

Fig. S26d. MS spectrum of **5m**.

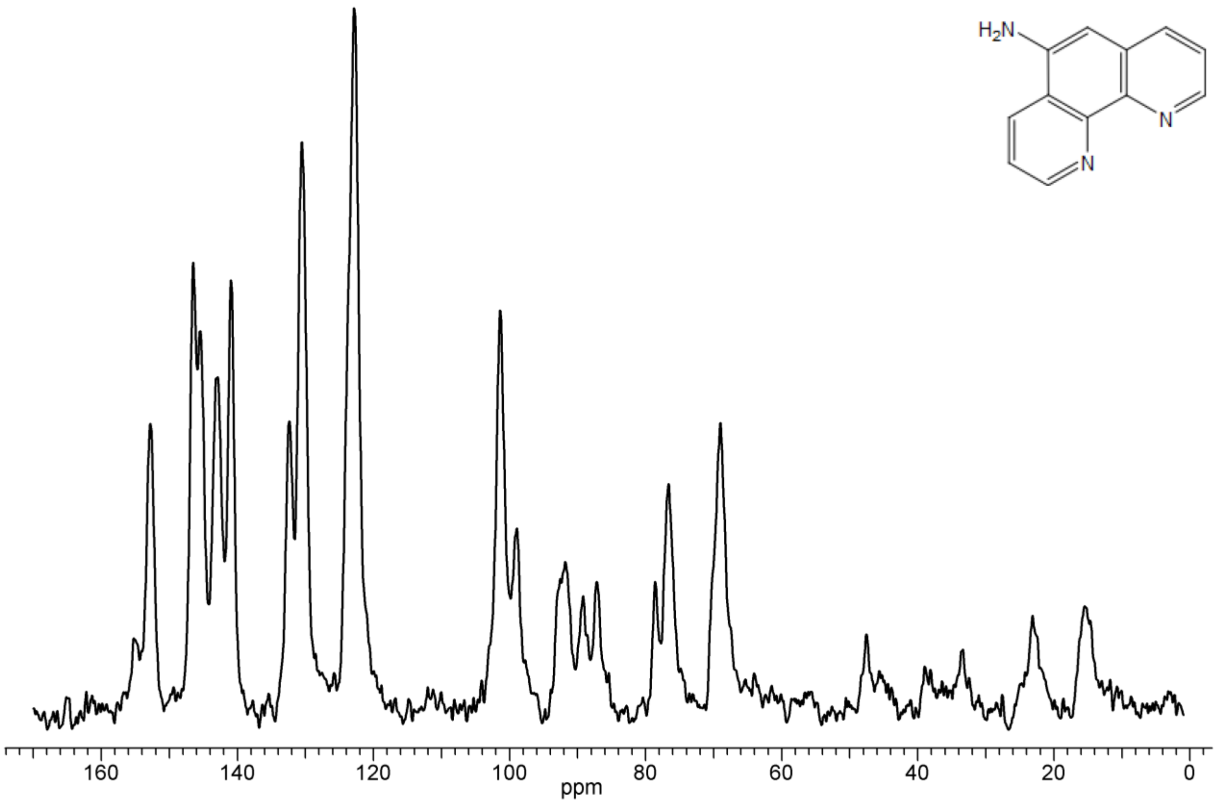

Fig. S27a. ^{13}C CP/MAS NMR spectrum of **5l**.

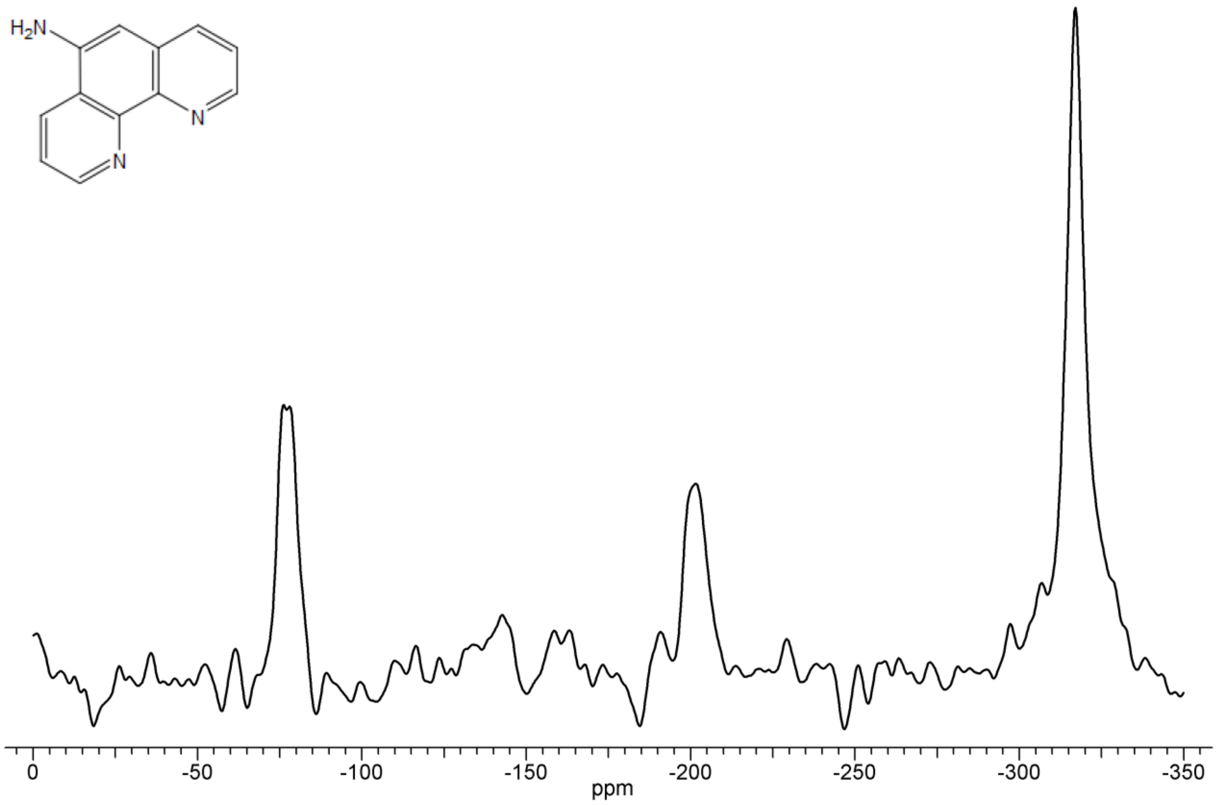

Fig. S27b. ^{15}N CP/MAS NMR spectrum of **5l**.

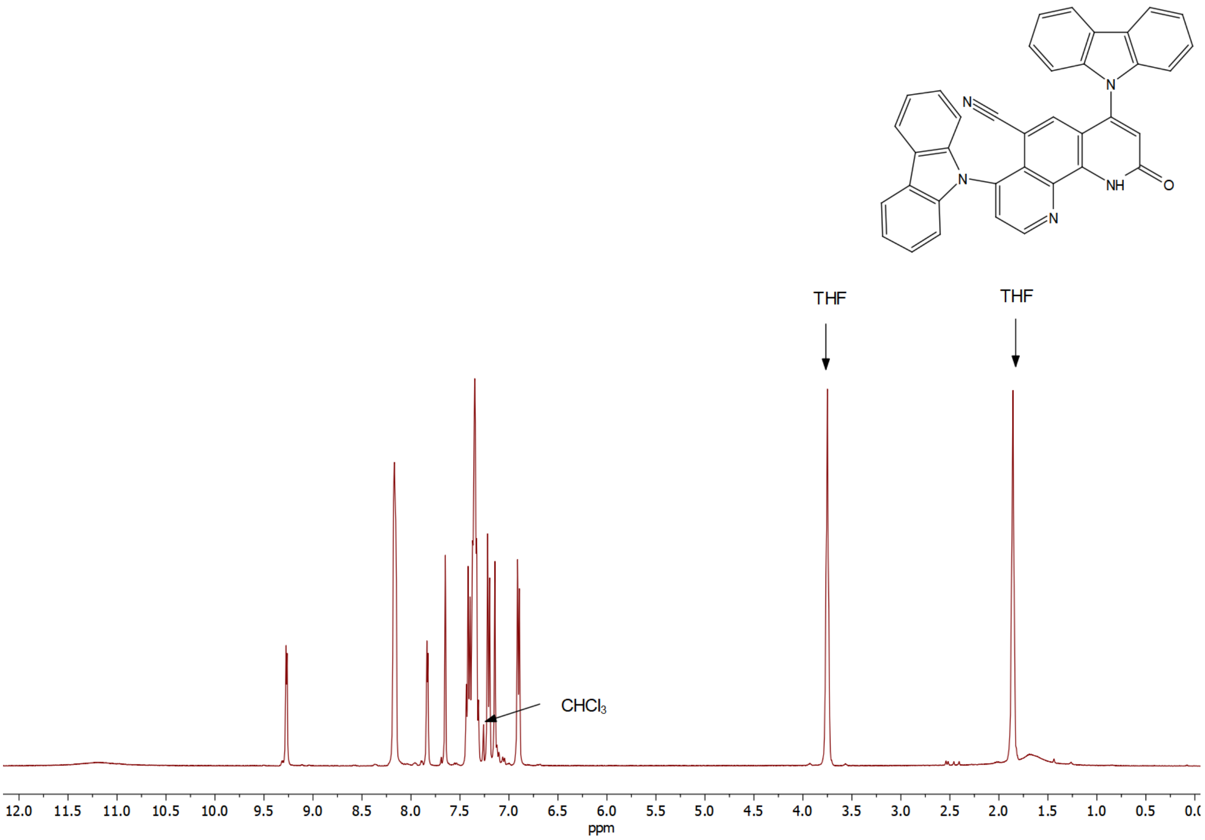

Fig. S28a. ^{1}H NMR ($CDCl_3$; 400.2 MHz) spectrum of **6a.**

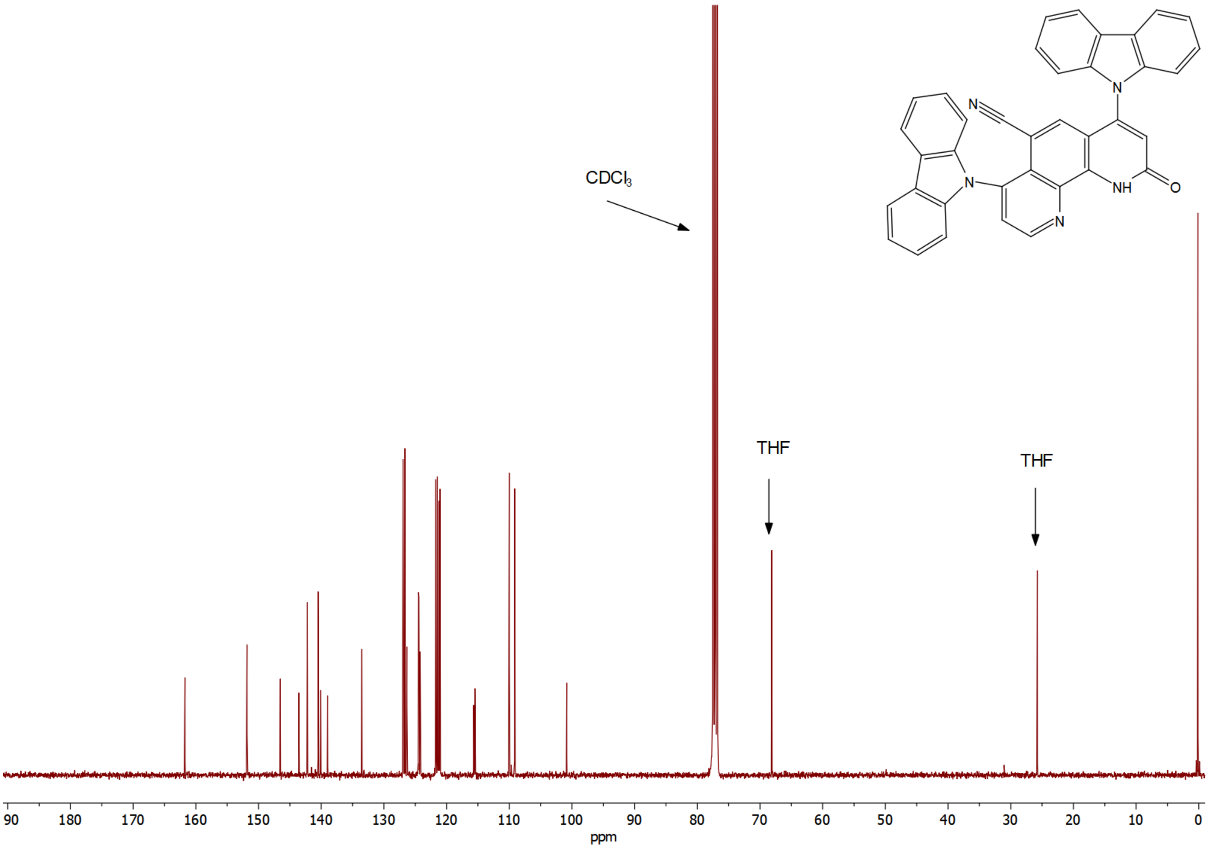

Fig. S28b. ^{13}C{^{1}H} NMR ($CDCl_3$; 100.6 MHz) spectrum of **6a**.

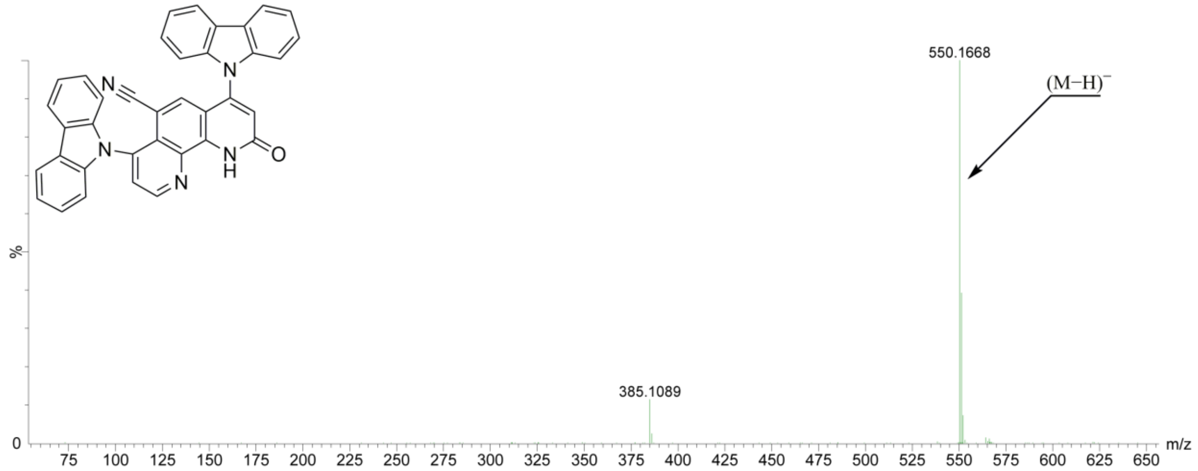

Fig. S28c. MS spectrum of **6a**.

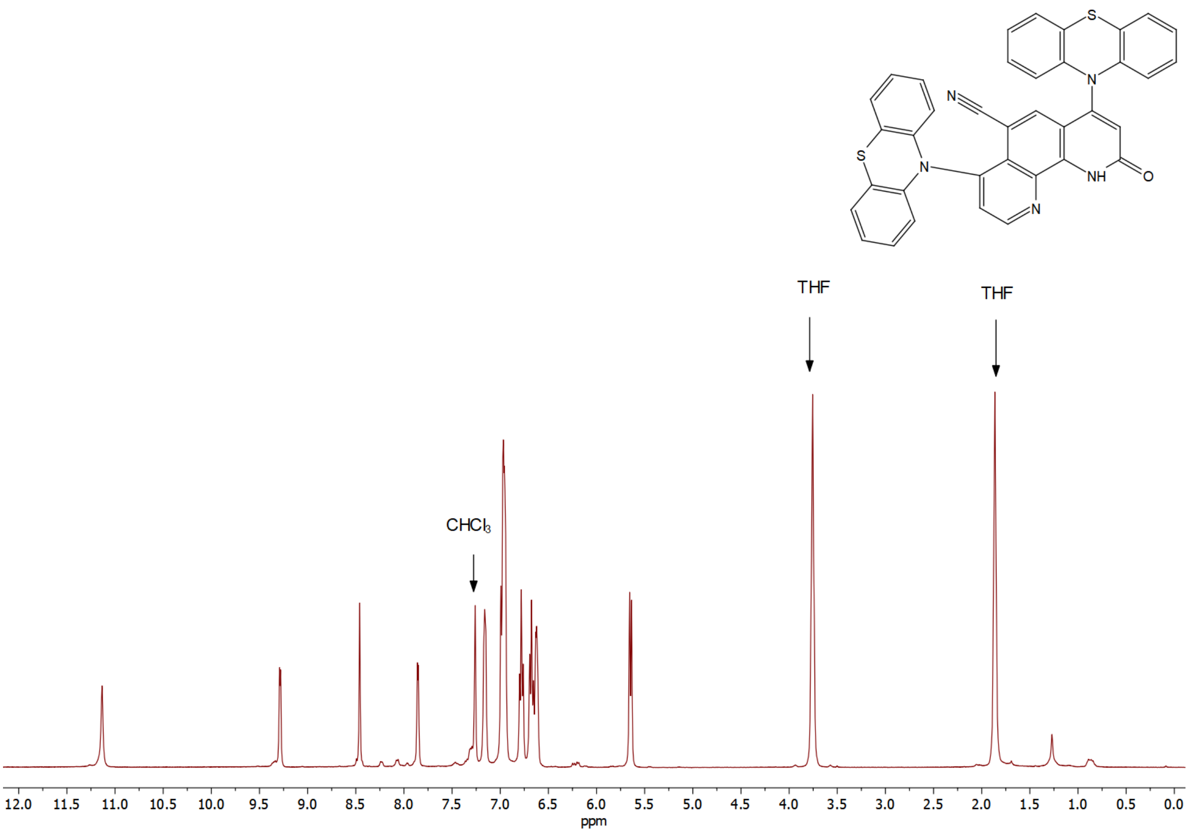

Fig. S29a. ^{1}H NMR ($CDCl_3$; 400.2 MHz) spectrum of **6b**.

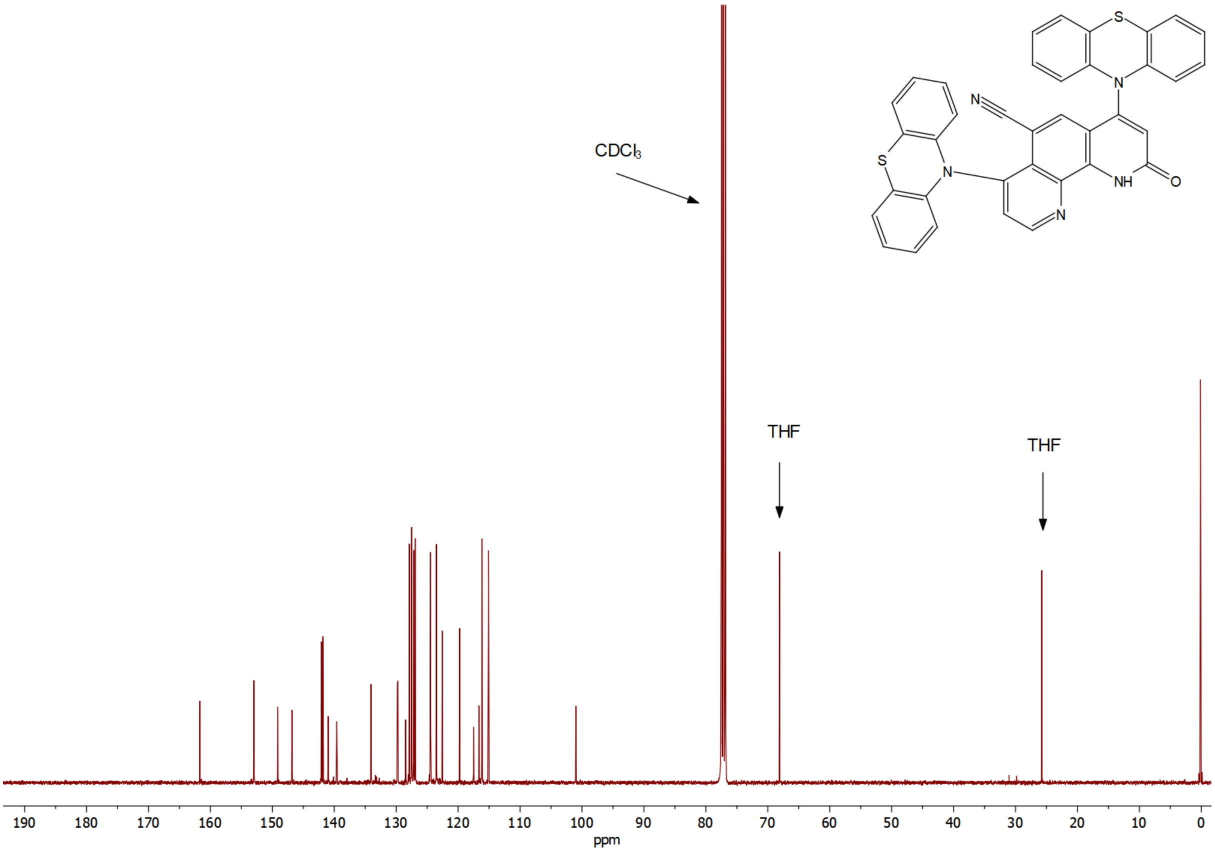

Fig. S29b. $^{13}C\{^{1}H\}$ NMR ($CDCl_3$; 100.6 MHz) spectrum of **6b**.

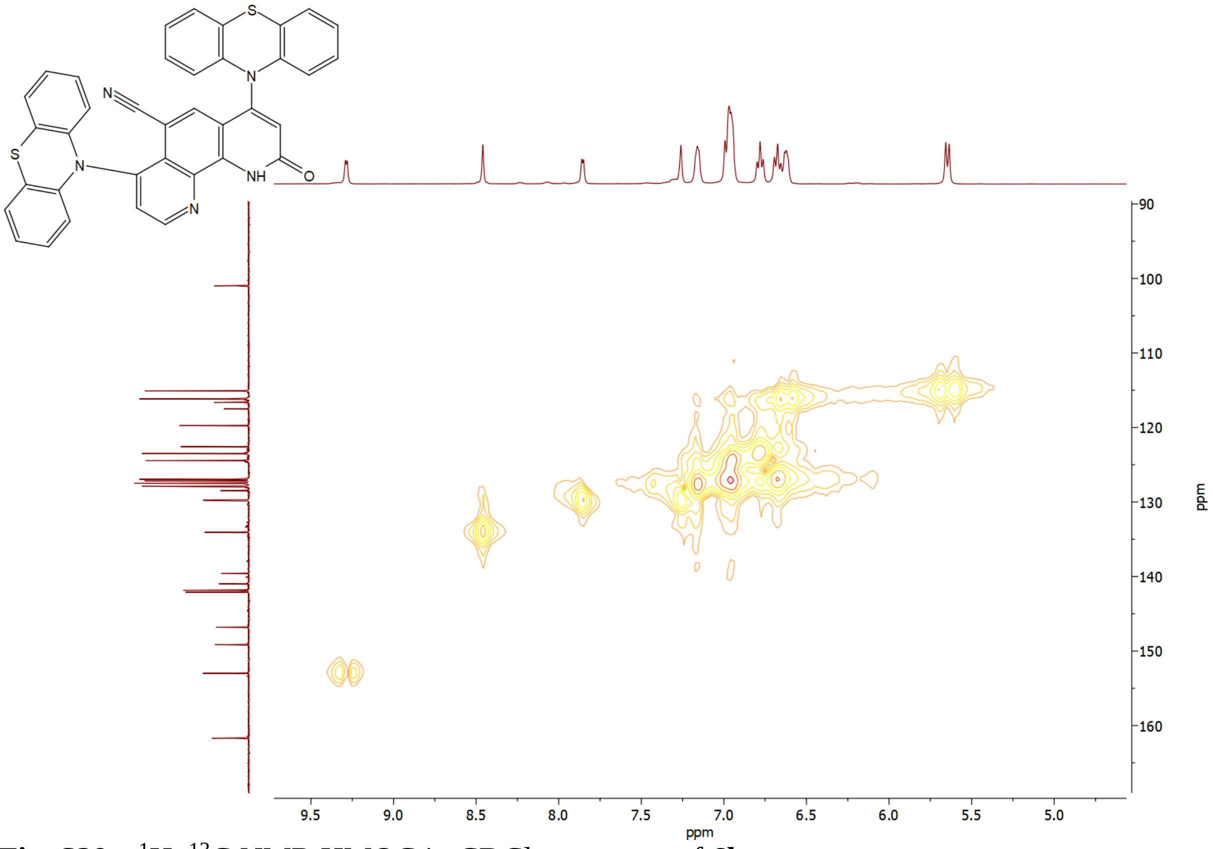

Fig. S29c. ^{1}H, ^{13}C NMR HMQC in $CDCl_3$ spectrum of **6b**.

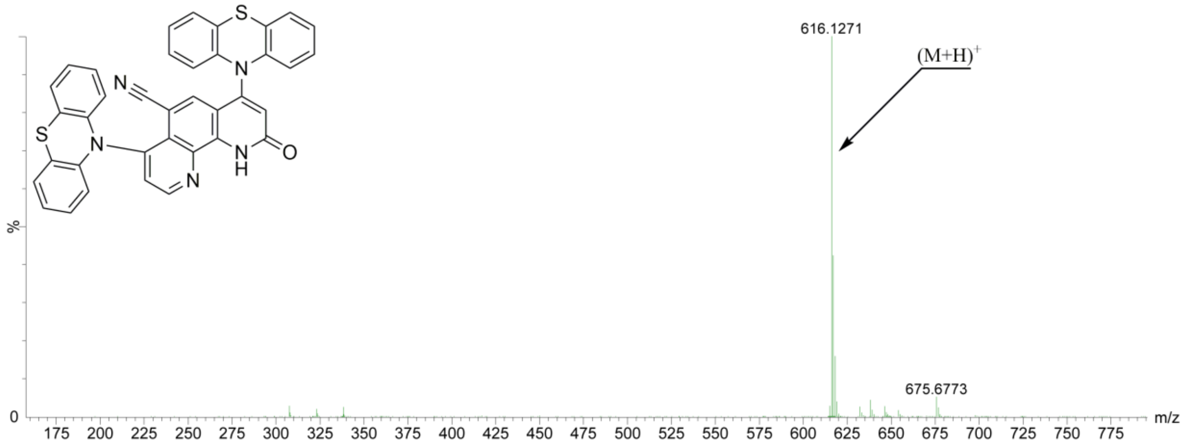

Fig. S29d. MS spectrum of **6b**.
